# Multi-Target Anti-Endometriosis Mechanisms of *Camellia sinensis* Polyphenols: An Integrated Computational and Transcriptomic Study

**DOI:** 10.3390/life16101613

**Published:** 2026-09-26

**Authors:** Rahni Hossain, Rifat Jafrin, Jitbanjong Tangpong, Md. Atiar Rahman, Mehmet Sütyemez

**Affiliations:** 1 Department of Medical Technology International, School of Allied Health Sciences, Walailak University, Nakhon Si Thammarat 80160, Thailand; rjitbanj@wu.ac.th; 2 Research Excellence Center for Innovation and Health Product (RECIHP), Walailak University, Nakhon Si Thammarat 80160, Thailand; 3 Institute of Biochemistry and Biology, Faculty of Science, University of Potsdam, 14476 Potsdam, Germany; jafrin@uni-potsdam.de; 4 Department of Biochemistry and Molecular Biology, University of Chittagong, Chittagong 4331, Bangladesh; atiar@cu.ac.bd; 5 Department of Horticulture, Faculty of Agriculture, Kahramanmaraş Sütçü İmam University, Kahramanmaraş 46050, Turkey; sutyemez@ksu.edu.tr

**Keywords:** *Camellia sinensis*, endometriosis, network pharmacology, molecular docking, EGCG, AGE-RAGE signaling, estrogen receptor, green tea catechins

## Abstract

Endometriosis is a chronic inflammatory disorder linked to estrogen, affecting about 10% of women of childbearing age. Current treatments, including hormonal and surgical options, have limitations related to side effects and recurrence. Green tea (*Camellia sinensis*) is rich in polyphenols and has known anti-estrogenic, anti-angiogenic, and anti-inflammatory effects, but its mechanism against endometriosis is unclear. Using phytochemical profiling, network pharmacology, and molecular docking, we identified twenty significant metabolites. From 619 predicted compound targets and 3065 endometriosis-related genes, we identified 292 common targets that formed a connected PPI network (*p* < 1.0 × 10^−16^) centered on key proteins such as TP53 and ESR1, with components linked to estrogenic and inflammatory processes. Functional enrichment revealed key processes such as oxidative stress and cell proliferation. Transcriptomic analysis confirmed consistent downregulation of ESR1 in ectopic endometrial tissue. Molecular docking confirmed that ECG has a strong affinity for ESR1 (−7.84 kcal/mol), marmesin binds notably to PGR, and a unique EGCG–EP300/CREBBP epigenetic pathway exists. These findings outline a dual endocrine–inflammatory mechanism in which ESR1 emerges as the most strongly supported target—validated by network centrality, reproducible transcriptomic down-regulation, and docking—while CYP19A1 is nominated as a plausible complementary node, and suggest that *C. sinensis* polyphenols merit experimental evaluation as complementary anti-endometriotic agents.

## 1. Introduction

Endometriosis is a chronic gynecological condition that relies on estrogen, characterized by the presence of endometrial-like tissue, both glandular and stromal, outside the uterine cavity, most frequently found on the ovaries, pelvic peritoneum, and rectovaginal septum [1,2]. It is estimated to affect 6–10% of women of childbearing age and nearly half of those who experience infertility or chronic pelvic pain, creating a significant impact on quality of life, productivity, and healthcare systems [3]. Despite its high prevalence, this condition is often diagnosed after several years of delay, and the molecular mechanisms behind it are still not completely understood [1,4]. Current models suggest a self-perpetuating interaction involving localized excess estrogen, progesterone resistance, chronic inflammation, oxidative stress, abnormal blood vessel formation, and increased signaling for growth and resistance to cell death, all of which contribute to the survival and growth of ectopic lesions [5]. A key aspect of this pathophysiology is an imbalanced endocrine microenvironment [6]. Ectopic lesions exhibit abnormal local estradiol production due to overactivity of aromatase (CYP19A1) and other steroidogenic enzymes, along with an altered estrogen-receptor ratio in which estrogen receptor β is more dominant than estrogen receptor α (ESR1), while signaling from the progesterone receptor (PGR) is diminished [7]. Estrogen promotes prostaglandin synthesis mediated by cyclooxygenase-2 (PTGS2) and activates NF-κB-dependent inflammatory processes, creating a positive feedback loop between estrogen signaling and inflammation that is further intensified by reactive oxygen species in the peritoneal cavity [8]. This combined endocrine and inflammatory nature strongly justifies treatment approaches that target estrogen production, receptor signaling, and inflammatory substances concurrently, rather than focusing on individual components in isolation.

The current treatment methods focus on hormonal suppression through combined oral contraceptives, progestins, gonadotropin-releasing hormone agonists and antagonists, and aromatase inhibitors, as well as surgical removal [9]. While these strategies often effectively manage symptoms, they are hindered by hypoestrogenic side effects, high rates of recurrence following treatment or surgery, and notably, their incompatibility with simultaneous conception for patients wanting to conceive [10,11]. Among non-hormonal options, dietary polyphenols are especially promising for endometriosis for four main reasons. First, since endometriosis involves estrogen dependence, inflammation, angiogenesis, and oxidative stress, polyphenols uniquely target all four areas by modulating estrogen receptors, reducing inflammation via NF-κB/PTGS2, inhibiting VEGF-driven blood vessel formation, and scavenging ROS. Second, unlike experimental kinase inhibitors, polyphenols from food have extensive human use data, are well tolerated, and are suitable for a condition requiring long-term management and fertility considerations. Third, green tea (*Camellia sinensis*) catechins specifically show preclinical evidence of reversing lesions and preventing angiogenesis in animal models, offering a disease-specific basis that generic antioxidants lack. Fourth, their chemical diversity within standardized extracts allows for a true multi-target approach. We thus focus on *C. sinensis* polyphenols [12,13].

Green tea, the minimally processed leaf of *C. sinensis* (L.) Kuntze, is one of the most popular beverages globally and a rich source of flavan-3-ols in the diet. Its main components—(−)-epigallocatechin-3-gallate (EGCG), (−)-epicatechin-3-gallate (ECG), epigallocatechin (EGC), epicatechin, and catechin—along with flavonol glycosides, phenolic acids, and methylxanthines, provide well-established antioxidant, anti-angiogenic, anti-proliferative, and anti-estrogenic effects [14,15]. Particularly, EGCG has demonstrated potential in preclinical models of endometriosis, as it reduces lesion angiogenesis, growth, and inflammatory signaling [15]. However, most mechanistic research has focused on individual catechins targeting specific pathways, leaving the multi-targeted effects of the whole extract—how its chemically varied metabolites collectively interact with the endometriotic targets—largely unexplored [16].

Network pharmacology has become an effective framework for unraveling complex multi-component and multi-target interactions; however, its predictive capabilities are frequently constrained by two common limitations: dependence on compound lists derived from databases that may not accurately represent the true composition of a specific preparation, and the lack of validation against expression data relevant to disease [17,18]. To remedy these issues, we established an integrated workflow grounded in experimental evidence, where UHPLC-QTOF-MS-based phytochemical analysis of 20 compounds from *C. sinensis* extract (CSE) with network pharmacology; unlike most similar studies, we validate predicted disease-related targets against actual differential gene expression data from two separate cohorts of endometriosis patients. We also confirm our molecular docking grid parameters using CASTp-based pocket detection and cross-reference them with experimentally determined ligand coordinates. Through this integrated and expression-validated methodology, we distinguish a specific mechanism whereby EGCG uniquely interacts with the tumor suppressor TP53 and the transcriptional coactivators EP300 and CREBBP among the twenty tested compounds, while the broader catechin/flavonoid group focuses on the ESR1, CYP19A1, PTGS2, and AKT1. This study aimed to (i) characterize the phytochemical profile of a standardized *C. sinensis* extract using UHPLC–QTOF–MS; (ii) identify potential human protein targets of the compounds and compare them with curated genes linked to endometriosis; (iii) analyze the shared-target network, including its topology, hub genes, and functional enrichment; (iv) validate the predicted targets with independent patient transcriptomes; and (v) explore compound–target interactions through molecular docking. Collectively, these results offer a systems-level, partially expression-validated framework for comprehending the multi-target therapeutic potential of green tea catechins in endometriosis and propose specific, testable hypotheses—especially regarding EGCG’s interaction with coactivators—for future experimental validation.

## 2. Materials and Methods

### 2.1. Phytochemical Profiling and Compound Selection

*C. sinensis* (L.) Kuntze leaves were purchased from Chiang Rai Province, northern Thailand. The CSE analyzed here is the same green tea pressurized hot-water extract (GPHWE) prepared and characterized in our previous study [19]. Briefly, we extracted air-dried, ground leaves (100 g) with distilled water (800 mL; a plant material-to-solvent ratio of 1:8, *w*/*v*) by pressurized hot-water extraction at 100 °C and 15 psi for 15 min. After cooling to room temperature, the extract was filtered (Whatman No. 1) (Sigma-Aldrich, St. Louis, MO, USA) and centrifuged (1500 rpm, 10 min) (Rotina 420 R; Hettich, Tuttlingen, Germany). The supernatant was freeze-dried (EYELA FDU-1200, Tokyo, Japan) and the powder stored in an airtight container at −20 °C until analysis. The extract was standardized based on the antioxidant marker content determined for the same batch [19]: total phenolic content 258.61 ± 32.48 mg gallic acid equivalents/g dry weight (Folin–Ciocalteu) and total flavonoid content 98.91 ± 22.19 mg quercetin equivalents/g dry weight (aluminum chloride method); radical-scavenging IC_50_ values were 72.84 ± 4.90 (DPPH), 94.87 ± 8.27 (hydroxyl), and 77.82 ± 8.04 µg/mL (nitric oxide). *C. sinensis* leaves were obtained as a commercially sourced material; accordingly, a specific cultivar, geographic coordinates, batch/lot number, and a deposited voucher specimen were not available, and formal taxonomic authentication was not performed.

The composition of CSE was analyzed by UHPLC–QTOF–MS (Agilent TOF/Q-TOF Mass Spectrometer, 6200-series TOF/6500-series Q-TOF; Agilent Technologies, Santa Clara, CA, USA) with a Dual Agilent Jet Stream (AJS) electrospray-ionization (ESI) source, operated in Auto-MS/MS mode in both positive and negative polarities. Separation used a Zorbax Eclipse Plus C18 Rapid Resolution HD column (150 mm × 2.1 mm, 1.8 µm; Agilent) with 0.1% formic acid in water (A) and 0.1% formic acid in acetonitrile (B) (UHPLC grade; Sigma-Aldrich, St. Louis, MO, USA); gradient 0 min 100% A, 35 min 10% A/90% B, 37 min 100% A, then a 3-min post-run at 100% A; flow 0.200 mL/min; injection 2 µL; column temperature 25 °C. MS source and scan parameters were: drying-gas temperature 350 °C at 10 L/min; nebuliser 60 psig; sheath-gas temperature 275 °C at 12 L/min; capillary voltage (VCap) 4000 V; nozzle voltage 2000 V; fragmentor 175 V; skimmer 65 V; octopole RF peak 750 V. Fixed collision energies of 10, 20 and 40 V were applied. Spectra were acquired over *m*/*z* 100–1500 (MS) and *m*/*z* 50–1500 (MS/MS). Continuous mass calibration used reference ions at *m*/*z* 121.0509 and 922.0098 (positive mode) and *m*/*z* 112.9856 and 1033.9881 (negative mode). The extract was reconstituted at 10 mg/mL, filtered through a 0.2-µm nylon membrane, and injected directly. Data acquisition used MassHunter Workstation LC/MS Data Acquisition (B.08.00); compound identification used MassHunter Qualitative Analysis Workflows (B.08.00) with the METLIN Metabolite Personal Compound Database and Library (PCDL) and PCDL Manager (version 8) [20].

Compound selection relied on analytical confidence, unrelated to disease. Of 159 features detected in positive (84) and negative (75) modes, compounds matched METLIN PCDL by accurate mass [21] (error ≤ 5 ppm, median ≈ 1 ppm), isotopic pattern, and confirmed by MS/MS spectral-library matching (score ≥ 85; library-match score ≥ 80; MSI Level 2) [21]. Eighteen constituents met these criteria. Two major green-tea catechins, ECG and EGC, were retained based on accurate mass and molecular formula matching (≤1 ppm), despite lacking confirmatory MS/MS library hits (MSI Level 3) [21]. Caffeine was confirmed by MS/MS library match (score 96.7), with larger mass deviation due to detector saturation. No abundance threshold or pre-defined disease list was used; relevance is reported post hoc via literature, ensuring unbiased network analysis. Identification metrics are in Table S1 (20 compounds) and Table S2 (all features).

### 2.2. ADMET Profiling

The physicochemical and ADMET characteristics of all 20 compounds were assessed using SwissADME [22] and RDKit [23] online platforms (http://www.swissadme.ch/index.php, http://www.rdkit.org) accessed on 20 August 2026. Key properties evaluated included Lipinski rule compliance, quantitative estimate of drug-likeness (QED), PAINS alerts, oral bioavailability, hERG inhibition risk, drug-induced liver injury (DILI) risk, Ames mutagenicity, and nuclear receptor (NR-ER, NR-Aromatase) activity.

### 2.3. Target Prediction and Disease Gene Collection

Canonical SMILES for the twenty compounds were uploaded to Swiss Target Prediction [24] (http://swisstargetprediction.ch/), accessed on 20 August 2026, selecting *Homo sapiens* to create a combined set of 619 predicted protein targets, which were linked to gene symbols through UniProt. Genes associated with endometriosis (*n* = 3065) were collected from GeneCards/DisGeNET/OMIM [25,26,27] (https://www.genecards.org/, https://www.disgenet.org/, https://omim.org/ accessed on 22 August 2026). We employed a Venn diagram tool [28] (https://www.interactivenn.net/), accessed on 22 August 2026, to visualize the overlap between compound targets and disease genes, identifying 292 shared targets for further network analysis.

### 2.4. PPI Network Construction and Hub Gene Identification

The 292 shared genes were uploaded to the STRING 11.0 database [29] (https://string-db.org/), accessed on 22 August 2026 (*Homo sapiens*, confidence score ≥ 0.7), to create a protein–protein interaction network, which was then imported into Cytoscape 3.4.0 [30] (http://chianti.ucsd.edu/cytoscape-3.4.0/), also accessed on 22 August 2026. We pinpointed topological hub genes using CytoHubba (Degree, MCC, MNC algorithms) and validated them in R (igraph) through degree and betweenness centrality; genes that ranked in the top decile across two or more methods were identified as consensus hub genes [31]. Additionally, we utilized betweenness centrality to find bridging nodes that connect the estrogen-signaling and NF-κB-inflammatory modules within the network.

### 2.5. Functional Enrichment Analysis

We conducted enrichment analyses for Gene Ontology (biological process, molecular function, cellular component) and KEGG pathways on the 292 shared genes using R with the clusterProfiler and org.Hs.eg.db packages [32] (adjusted *p* < 0.05, Benjamini–Hochberg), and we visualized the outcomes with enrichplot. Analyses for Gene Ontology (Biological Process, Cellular Component, Molecular Function) and KEGG pathways on the set of 292 common target genes were also executed using ShinyGO 0.80 [33] (http://bioinformatics.sdstate.edu/), accessed on 22 August 2026, and simultaneously performed in R with the clusterProfiler package (Benjamini–Hochberg adjusted *p* < 0.05) to validate the enrichment findings across different tools.

### 2.6. Gene Expression Validation

Publicly accessible transcriptomic data on endometriosis (GSE7305; GPL570 Affymetrix Human Genome U133 Plus 2.0 Array; 10 ectopic ovarian endometrioma samples versus 10 normal endometrium samples) [34] were obtained from the Gene Expression Omnibus and analyzed in R utilizing the GEOquery and limma packages [35]. After conducting a log2 transformation, the differential expression was evaluated through linear modeling and empirical Bayes moderation (with |log2 fold-change| > 1 and Benjamini–Hochberg adjusted *p* < 0.05) [36]. The menstrual cycle phase was considered as a potential confounding variable; the phase distribution was balanced between the disease and normal groups (8/8 follicular, 2/2 luteal), and a phase-adjusted differential expression model resulted in a completely concordant differentially expressed gene (DEG) set (100% gene overlap) compared to the unadjusted model, confirming that disease status, rather than menstrual phase, was the main factor causing the observed transcriptional differences. The DEG list obtained was intersected with the 292 common targets to create a set of expression-validated targets for further prioritization.

### 2.7. Cross-Dataset Validation

An additional, separate transcriptomic dataset related to endometriosis (GSE25628; GPL571 platform; 8 ectopic, 8 eutopic, and 6 normal endometrium samples) was examined using the same differential expression methodology (comparison of ectopic versus normal endometrium) to evaluate the consistency of significant hub gene expression findings across different patient groups and microarray platforms.

### 2.8. Molecular Docking

The three-dimensional configurations of the active compounds (formatted as SDF) were sourced from the PubChem database [37] (https://pubchem.ncbi.nlm.nih.gov/), accessed on 23 August 2026, while the crystal structures of the selected target proteins (provided in PDB format) were obtained from the Protein Data Bank (PDB) [38] (https://www.rcsb.org) accessed on 23 August 2026. Eight targets were chosen for docking based on factors such as network centrality (hub-gene status), expression-validation status, and/or network bridging-node status: tumor protein p53 (TP53), E1A-binding protein p300 (EP300), CREB-binding protein (CREBBP), progesterone receptor (PGR), estrogen receptor alpha (ESR1), aromatase (CYP19A1), cyclooxygenase-2 (PTGS2/COX-2), and AKT1. We performed structural refinement of the target proteins using ChimeraX (version 1.8) [39] (https://www.cgl.ucsf.edu/chimerax/) accessed on 23 August 2026, and then optimized and prepared the receptors for docking in Discovery Studio Visualizer 2020 by eliminating crystallographic water molecules and co-crystallized heteroatoms, adding polar hydrogens, and assigning charges. Molecular docking simulations were conducted using AutoDock Vina 1.2.0. Candidate binding pockets for each target were identified using the Computed Atlas of Surface Topography of proteins (CASTp) server [40] (http://sts.bioe.uic.edu/castp/) accessed on 23 August 2026. Docking used an exhaustiveness of 8, generating 9 poses per ligand across 10 independent runs, with an energy range of 3 kcal/mol. The top-ranked pose by predicted binding free energy (ΔG) was retained. Predicted dissociation constants were derived from ΔG using Kd = exp(ΔG/RT) at T = 298.15 K (R = 1.987 × 10^−3^ kcal·mol^−1^·K^−1^).

## 3. Results

### 3.1. Phytochemical Composition of CSE

The UHPLC–QTOF-MS profiling in both ionization modes produced a detailed phytochemical fingerprint, mainly featuring flavan-3-ols, flavonol glycosides, phenolic acids, and purine alkaloids. Accurate-mass database searching annotated 159 features—84 in ESI+ and 75 in ESI−—with mass errors typically ≤ 5 ppm (median ≈ 1 ppm), indicating high-confidence formula assignment (the sole exception, caffeine, was confirmed by MS/MS spectral-library matching despite a larger accurate-mass deviation on its dominant peak; Table 1). From these features, twenty compounds met the identification criteria and were carried forward for analysis (Table 1). The base peak chromatograms highlighted the distinctive composition of *C. sinensis*. In positive mode (Figure 1A), the profile was dominated by caffeine (*m*/*z* 195.0902, [M + H]^+^, RT 9.47 min) and theophylline, along with flavonoid aglycones such as kaempferol and (−)-naringenin. In negative mode (Figure 1B), key signals included epicatechin (*m*/*z* 289.0731, [M − H]^−^, RT 10.65 min) and its derivative ECG (*m*/*z* 441.0825, RT 12.84 min), together with other constituents such as catechin, EGC, and flavonol glycosides. The reported panel reflects the actual measured metabolome of the *C. sinensis* extract rather than a curated flavonoid list. It spans the compound classes characteristic of green tea: flavan-3-ols/catechins (EGCG, ECG, EGC, epicatechin, catechin, procyanidin B2), flavonols and flavonoid glycosides (kaempferol, herbacetin, rutin, quercetin 3-galactoside), a flavanone (naringenin), phenolic acids (gallic, caffeic, chlorogenic), the signature purine methylxanthines (caffeine, theophylline), the characteristic tea amino acid (L-theanine), and minor coumarins (marmesin, aesculin, 4-hydroxycoumarin). Because compounds were selected purely on identification confidence, we retained all confidently identified constituents regardless of chemical class, ensuring the network analysis represents the whole administered extract—a deliberate strength of this study over analyses built on database-derived compound lists that may not reflect the true composition of a specific preparation. Notably, several metabolites were detected in both ionization modes, further supporting their assignment. The twenty selected compounds accounted for approximately 37.8% of the total base-peak signal, with epicatechin, caffeine, and ECG being the most abundant.

### 3.2. Strategic Bioavailability Screening and ADMET Profiling

To establish the relevance of an orally administered anti-endometriotic phytotherapeutic, twenty *C. sinensis* metabolites were evaluated for drug-likeness, pharmacokinetics, toxicity, and nuclear-receptor engagement (Table 2). All metabolites met Lipinski’s Rule of Five, with fifteen fully compliant and five with one violation due to high hydrogen-bond-donor counts. Lipophilicity was low (cLogP −1.93 to 1.87), and molecular weights spanned from 162.14 g/mol to 610.5 g/mol. Quantitative drug-likeness scores were highest for marmesin (0.78) and (−)-naringenin (0.74), while large glycosides had lower scores. Most metabolites had favorable human intestinal absorption, but flavonol glycosides were predicted to be poorly absorbed. Predicted distribution indicated that flavonoid aglycones were highly protein-bound, while caffeine, theophylline, and theanine were mostly unbound, suggesting better availability at target tissues. Toxicity screening revealed low cardiotoxicity risks, but galloylated catechins showed elevated liver injury probabilities. Mutagenicity was highest for quercetin 3-galactoside, while caffeic and chlorogenic acids had lower risks. The galloylated and glycosylated polyphenols were retained with caution, given their dose-dependent safety profile (Supplementary Table S3).

### 3.3. Compound-Disease Target Intersection

The twenty CSE metabolites were analyzed using SwissTargetPrediction (*Homo sapiens*), and predicted proteins were standardized to official gene symbols through UniProt, resulting in 619 unique targets after removing duplicates. Targets related to endometriosis were collected from GeneCards, DisGeNET, and OMIM (search term “endometriosis”; Supplementary Table S4), totaling 3065 disease-associated genes. The intersection of these two sets (using Venny 2.1; Figure 2) identified 292 shared targets (47.2% of the 619 compound targets), along with 327 CSE-specific and 2773 disease-specific genes. These 292 common targets suggest a potential mechanistic connection between green tea metabolites and endometriosis. To determine whether specific compounds drove this overlap, we compared each compound’s targets individually to the disease gene set (Supplementary Table S5). The overlap was distributed broadly: each compound contributed between 47 and 84 of the 292 targets, with EGCG leading at 84 (28.8%), followed by naringenin (65), caffeic acid (62), and herbacetin (61). Even the least-connected compounds (caffeine and theophylline) mapped to 47–48 targets. Overall, 175 targets (60%) were shared by two or more compounds, 36 targets (12%) were shared by 10 or more compounds (including one target shared by 18/20), while 117 targets were specific to individual compounds. This suggests a true polypharmacological interaction, involving a shared core of targets plus compound-specific ones, rather than dominance by just a few constituents.

### 3.4. Protein–Protein Interaction Network and Hub Gene Identification

The 292 shared targets created a protein–protein interaction network consisting of 919 edges among 252 interconnected nodes (40 genes were not connected at the specified confidence level and were therefore excluded from the topological analysis) (Supplementary Figure S1, Table S6). PPI enrichment analysis revealed that the network exhibited significantly more interactions than would be anticipated by chance (325 edges expected; PPI enrichment *p* < 1.0 × 10^−16^), suggesting that the 292 shared targets constitute a biologically meaningful, organized functional network (Figure 3) instead of a collection of unrelated genes.

The degree-centrality ranking identified TP53 (degree = 56) as the most interconnected node, followed by SRC (42), STAT3 (37), HSP90AA1 (36), AKT1 (34) and ESR1 (32) (Figure 4; Supplementary Table S7). Betweenness-centrality analysis highlighted these core hubs together with prostaglandin-endoperoxide synthase 2 (PTGS2) and aromatase (CYP19A1) as key bridging nodes: although these two enzymes had comparatively modest degree, they ranked 4th (PTGS2, betweenness = 3246) and 7th (CYP19A1, betweenness = 2242) of all 252 network nodes in betweenness, consistent with a role in linking the estrogen-signaling and inflammatory modules of the network. To confirm that hub identification did not depend on the chosen metric, we compared three cytoHubba algorithms (Degree, MCC and MNC), which produced overlapping but non-identical rankings reflecting their different emphases (global connectivity, dense-clique membership and neighbourhood size, respectively). Of the top 10 nodes, four (SRC, STAT3, PIK3CA and PIK3R1) ranked highly by all three methods, and ten appeared in the top 10 of at least two (TP53, SRC, STAT3, HSP90AA1, AKT1, ESR1, CTNNB1, HSP90AB1, PIK3CA and PIK3R1). Genes ranked highly by ≥2 of the three algorithms were retained as consensus hubs (Supplementary Table S8), ensuring that hub identification did not rely on any single centrality measure.

### 3.5. GO and KEGG Pathway Enrichment

The combined analysis of GO (Biological Process, Cellular Component, Molecular Function) and KEGG pathways for the 292 shared targets is presented in Figure 5. The enrichment bar chart (Figure 5A) indicated a significant enrichment for biological processes related to hormone and xenobiotic response (such as response to xenobiotic stimulus, oxidative stress response, and cellular response to peptide and peptide hormone), cellular components linked to receptor complexes and membrane rafts (including membrane raft, membrane microdomain, and focal adhesion), as well as molecular functions associated with transcription factors and kinases (like DNA-binding transcription factor binding, transcription coregulator binding, nuclear receptor activity, and protein tyrosine kinase activity). When ranked by gene count, the largest KEGG categories included PI3K-Akt signaling, chemical carcinogenesis—reactive oxygen species, and HIF-1 signaling, while the AGE-RAGE signaling pathway in diabetic complications emerged as the most prominently enriched category when assessed by fold enrichment. Mapping the key network hubs onto the enriched categories (Figure 5B) revealed that the central targets—TP53, SRC, STAT3, HSP90AA1, AKT1, and ESR1—were distributed throughout all four ontology branches, suggesting that these top hub genes simultaneously influence multiple enriched processes instead of being confined to a single functional category. To identify which targets are fundamental to the most significant biological processes, the leading GO Biological Process terms were linked to the respective genes and colored according to their differential expression in GSE7305 (Figure 5C). This gene-term chord visualization indicated that a core group of nuclear receptors and hormone-metabolism enzymes—including ESR1, PGR, NR3C1, PPARG, NR1H4, AHR, and the AKR1C1/HSD11B2/ALDH1A2 steroid-processing enzymes—were shared among hormone metabolic processes, intracellular receptor signaling pathways, and responses to peptide hormones, highlighting endocrine regulation as the primary enriched axis. Another module that included TP53, CDK1, JAK3, INSR, and AGTR1 linked the cellular response to peptide and the response to xenobiotic stimuli with the regulation of inflammatory responses, while the immediate-early nuclear receptors NR4A1/NR4A2/NR4A3 also contributed to the rhythmic process category. The log_2_ fold-change overlay revealed that most of these BP-driving genes were differentially expressed in ectopic endometrium, establishing a direct connection between the enriched processes and the disease transcriptome rather than just predictive assessment.

Due to its recognition as the top-ranked enriched pathway and its specific relevance to the pathophysiology of endometriosis—regardless of its originally identified role in diabetic complications—the AGE-RAGE signaling pathway was chosen for further mechanistic analysis. When the actual GSE7305 differential expression values (log2 fold-change) were overlaid onto the AGE-RAGE pathway topology (Figure 6), it displayed a directionally mixed outcome: TGFβRI/RII, AT1R, components of NADPH oxidase, JNK, AP-1, NF-κB, MCP-1, VCAM-1, AKT, and STAT1/3/5 showed increased expression in ectopic endometrium, while ERK1/2, p38 (at two positions within the pathway), p21 RAS, and COL1 exhibited decreased expression, which is in line with a biologically plausible activation pattern rather than a blanket dysregulation across the pathway.

To connect the individually enriched pathways into a unified mechanistic framework, we categorized significant KEGG pathways by the downstream pathophysiological processes they lead to and visualized them concerning the disease outcome (Figure 7). This synthesis of pathway themes revealed that the enriched pathways direct towards four fundamental processes involved in the formation of endometriotic lesions: oxidative stress (chemical carcinogenesis involving reactive oxygen species, AGE-RAGE, HIF-1 signaling), cell migration (focal adhesion, proteoglycans in cancer), cell proliferation (PI3K-Akt and p53 signaling), and estrogen signaling (estrogen signaling pathway, endocrine resistance, steroid hormone biosynthesis), all of which culminate in endometriosis. The convergence of otherwise distinct pathways on estrogen signaling and oxidative stress highlights the dual endocrine–inflammatory mechanism suggested throughout the enrichment analysis.

### 3.6. Expression-Validated Targets in GSE7305

The differential expression analysis of GSE7305, which included 10 ectopic ovarian endometrioma samples and 10 normal endometrium samples, revealed 2791 significant differentially expressed genes (DEGs) (|log2FC| > 1, adjusted *p* < 0.05) at the probe level, corresponding to 1778 distinct genes (Figure 8). By intersecting this DEG set with the 292 common targets, we identified 65 expression-validated targets, representing compound targets with confirmed transcriptional dysregulation in patient tissue.

Hierarchical clustering of the 65 expression-validated targets across all 20 GSE7305 samples clearly separated disease and normal samples into two clusters (Figure 9), visually confirming that this gene set differentiates disease status. Among the 34 hub genes with a degree of 15 or greater, only TP53 and the estrogen receptor alpha (ESR1) were also validated through expression; the majority of the hub genes within the network (such as SRC, STAT3, HSP90AA1, AKT1, EP300, and CREBBP) were topologically prominent but not identified as significant differentially expressed genes (DEGs), aligning with the established understanding of their roles as signaling kinases, chaperones, and transcriptional coactivators that are primarily regulated through post-translational modifications and protein–protein interactions rather than through changes in transcriptional levels.

### 3.7. Reproducibility in GSE25628

To evaluate reproducibility across independent patient groups, the expression of TP53, ESR1, and PGR was examined in a second dataset (GSE25628) and compared with GSE7305 (Figure 10). ESR1 showed a statistically significant reduction in ectopic endometrium in both GSE7305 (log2FC = −2.917, adjusted *p* = 3.3 × 10^−10^) and GSE25628 (log2FC = −0.794, adjusted *p* = 0.0012), indicating a consistent, reproducible finding across two platforms and patient populations. TP53 was significantly dysregulated in both datasets but in opposing directions (GSE7305: log2FC = −1.330, downregulated; GSE25628: log2FC = +0.972, upregulated). PGR was markedly downregulated in GSE7305 (log2FC = −2.436, adjusted *p* = 9.2 × 10^−8^) but did not reach significance in GSE25628 (adjusted *p* = 0.294). Extending the analysis to all three GSE25628 groups (Supplementary Figure S2; pairwise two-sample *t*-tests) revealed two distinct patterns. AKT1 and TP53 were up-regulated in eutopic endometrium relative to normal controls (*p* = 3.4 × 10^−6^ and 6.8 × 10^−4^) with no further change in ectopic tissue (eutopic vs. ectopic *p* = 0.94 and 0.89), consistent with a systemic “field effect” already present in the eutopic endometrium of affected women. In contrast, ESR1 decreased progressively and significantly at each step (normal vs. eutopic *p* = 0.0029; eutopic vs. ectopic *p* = 0.019; normal vs. ectopic *p* = 3 × 10^−5^), indicating a graded, lesion-associated reduction in estrogen-receptor expression. PGR showed a non-significant downward trend (normal vs. ectopic *p* = 0.21). These three-group results reproduce the direction and significance shown in the corresponding Figure 10 panels.

### 3.8. Compound-Target-Pathway Network

A three-tier compound-target-pathway network (Figure 11) was created to link a 20-compound panel to 8 prioritized docking targets and 10 curated pathways based on KEGG enrichment results. This analysis revealed that EGCG uniquely connected to TP53, EP300, and CREBBP, with EP300 and CREBBP independently converging on the HIF-1 signaling pathway. In contrast, the broader catechin/flavonoid panel targeted ESR1, CYP19A1, PTGS2, and AKT1, showcasing a wider range of pathway connections.

### 3.9. Docking Interactions with Key Targets

Molecular docking was restricted to predicted compound–target pairs rather than all possible combinations. For each of the eight prioritized targets, the compound(s) predicted to bind it in SwissTargetPrediction were docked; for the four targets predicted for a single compound, that compound was docked (EGCG for TP53, EP300 and CREBBP; marmesin for PGR), and for the four targets predicted for multiple compounds (ESR1, CYP19A1, PTGS2, AKT1), the principal predicted constituents—the extract’s major catechins and abundant flavonoids/phenolic acids—were docked. This gave 26 compound–target pairs in total (Table 3); non-predicted pairs were not evaluated. Among the twenty constituents, EGCG was the only compound predicted to target TP53, EP300, and CREBBP, with docking scores of TP53 (−4.67 kcal/mol, Kd = 379.10 μM), EP300 (−7.05 kcal/mol, Kd = 6.83 μM), and CREBBP (−5.18 kcal/mol, Kd = 159.71 μM). Marmesin targeted PGR (−7.25 kcal/mol, Kd = 4.84 μM), while ECG exhibited the strongest binding affinity against ESR1 (−7.84 kcal/mol, Kd = 1.80 μM). AKT1 displayed strong binding across five compounds (−6.29 to −7.31 kcal/mol, Kd: 4.36–24.34 μM). Rutin and gallic acid showed weaker binding against PTGS2 (−2.38 kcal/mol, Kd = 17.92 mM and −3.97 kcal/mol, Kd = 1.23 mM, respectively). The strongest docking complexes were depicted in Figure 12A–C: EGCG–EP300, ECG–ESR1, and Marmesin–PGR.

## 4. Discussion

In this research, we combined phytochemical profiling, network pharmacology, transcriptomic validation, and molecular docking to model how *C. sinensis* polyphenols may combat endometriosis. Our analysis revealed that a catechin-rich extract interacts with a target network focused on estrogen signaling, inflammation, oxidative stress, and cellular proliferation, reflecting the condition’s pathophysiology. Notably, ESR1 was identified as a key target, while CYP19A1 (aromatase) played a lesser role, with modest binding energies (−6.0 to −6.5 kcal/mol) and lacking validation in transcriptomic data. Therefore, aromatase modulation remains a hypothesis to be tested through direct enzymatic inhibition assays.

The UHPLC–QTOF–MS analysis revealed that the extract mainly contains flavan-3-ols (epicatechin, ECG, EGCG, catechin, EGC), flavonol glycosides (rutin, quercetin 3-galactoside), phenolic acids (chlorogenic, gallic, caffeic), and methylxanthines (caffeine, theophylline). This typical composition highlights complementary bioactivities: catechins provide antioxidant properties, flavonols offer kinase-modulating activities, and methylxanthines influence signaling pathways [41]. By focusing on the measured metabolite profile, we enhance the biological relevance of predictions. Predicted ADMET profiling indicates that while many metabolites have drug-like properties, galloylated catechins present hepatotoxicity concerns and larger glycosides may have poor absorption, aligning with known pharmacokinetic challenges [42]. The pharmacologically relevant receptor engaged is ERα/ESR1, which differs from ERβ, the receptor linked to estrogen-dependent lesions. Green tea catechins preferentially bind ERβ, so down-regulation of ESR1 in ectopic tissue indicates its disease relevance rather than a directive to lower ERα. Thus, we view ESR1 as a modifiable component of the estrogen module, where catechins’ effects (agonist, antagonist, or SERM-like) present a specific hypothesis for further investigation.

Our most significant finding from the integrated analysis was the prominence of the estrogen axis. ESR1 was among the leading network hubs, was one of only two hub genes validated independently at the transcriptomic level, and—crucially—exhibited consistent downregulation in ectopic endometrium across two distinct datasets and platforms (GSE7305, log_2_FC = −2.917; GSE25628, log_2_FC = −0.794). ESR1 (ERα) was consistently downregulated in ectopic tissue across both datasets. Although endometriotic lesions are reported to show a shift toward ERβ dominance with reduced ERα [8], ESR2 was not measured in this study; we therefore interpret ESR1 as a reproducible disease-associated target rather than evidence of a coordinated receptor switch or a causal driver of lesion formation. Molecular docking further supported this axis, as epicatechin gallate exhibited the highest binding affinity to ESR1 in the entire screening (−7.84 kcal/mol; Kd = 1.80 μM), while several other catechins, along with chlorogenic acid, occupied the same ligand-binding pocket through conserved interactions with Glu353, Arg394, and His524. Concurrently, multiple catechins docked favorably with aromatase (CYP19A1), which serves as a bridging node connecting the estrogenic and inflammatory modules and whose pharmacological inhibition is already used clinically for endometriosis [43]. Collectively, these findings indicate that green tea polyphenols may target the estrogen axis at two complementary levels—reducing local estradiol production through aromatase modulation while acting as phytoestrogens or partial modulators at the ESR1 receptor level [44].

A recurring theme was the involvement of inflammatory and oxidative-stress signaling. Betweenness-centrality analysis identified PTGS2 (COX-2) as a crucial bridging node, and enrichment analysis highlighted the AGE–RAGE, HIF-1, and chemical carcinogenesis–reactive oxygen species pathways, with the AGE–RAGE cascade showing the greatest fold-change enrichment. The AGE–RAGE axis links the oxidative stress and inflammation pathways prominent in our findings. In endometriosis, advanced glycation end-products (AGEs) form in the oxidative, iron-rich peritoneal environment and bind to the RAGE receptor on endometrial and immune cells. This binding activates NADPH oxidase and NF-κB, increasing reactive oxygen species and pro-inflammatory cytokines such as IL-6, TNF-α, and MCP-1, while also upregulating VEGF. This creates a self-perpetuating cycle that promotes lesion survival, angiogenesis, and adhesion. Several key genes, such as NF-κB, AKT, STAT, VCAM-1, and MMPs, link to the KEGG AGE–RAGE pathway, even though it is annotated for diabetic complications—because its downstream effects are common. Evidence specific to endometriosis shows elevated AGEs and altered soluble RAGE (sRAGE) levels in serum, peritoneal, and follicular fluids, with a RAGE–EN-RAGE axis implicated in lesion inflammation [45,46,47,48]. We propose AGE–RAGE as a unified oxidative-inflammatory hypothesis worth further experimental testing.

Overlaying patient expression data onto this pathway supported the model: rather than random dysregulation, the cascade was activated in a directionally coherent manner—upregulation of NADPH oxidase components and the downstream NF-κB, AKT and STAT effectors, together with adhesion and chemotactic mediators—mechanistically connecting oxidative stress to the inflammatory and proliferative behaviour of ectopic tissue [49]. Both chlorogenic acid and EGCG showed favorable docking to PTGS2 (−7.46 and −7.23 kcal/mol, respectively), aligning with the known ability of green tea polyphenols to inhibit prostaglandin synthesis and redox-sensitive inflammatory transcription. The convergence of various distinct enriched pathways on oxidative stress and estrogen signaling reinforces the dual endocrine–inflammatory mechanism observed throughout our analysis.

The proliferative and survival aspects of the network were represented by the PI3K–Akt pathway and its effector AKT1, which were targeted by five compounds with consistently favorable binding energies (−6.29 to −7.31 kcal/mol), alongside the master regulator TP53, the most interconnected hub in the entire network. TP53 exhibited transcriptional dysregulation in both validation datasets but in opposite directions (downregulated in GSE7305, upregulated in GSE25628), highlighting the context-dependence of p53 signaling across different lesion types, disease stages, and patient populations, and urging caution against over-interpreting any single dataset’s direction. Nonetheless, the broad engagement of AKT1 by the catechin panel is significant, given the pathway’s pivotal role in the anti-apoptotic, pro-survival phenotype, which allows ectopic tissue to survive [50].

Analyzing the network at the compound level revealed a notable division of labor. EGCG uniquely connected to the transcriptional coactivators EP300 and CREBBP as well as to TP53, with these acetyltransferases converging on HIF-1 signaling, while the broader catechin and flavonoid panel engaged ESR1, CYP19A1, PTGS2, and AKT1. Docking results confirmed this distinction: EGCG bound EP300 with the highest affinity among its three unique targets (−7.05 kcal/mol; Kd = 6.83 μM), suggesting an epigenetic and hypoxia-related mechanism of action that complements, rather than duplicates, the receptor- and enzyme-targeted activities of the other catechins. This complementarity demonstrates the polypharmacological nature of botanical therapeutics, where structurally similar metabolites distribute their effects across parallel disease modules, resulting in a network-level impact that no single constituent can achieve alone [51].

The phenomenon of progesterone resistance, another characteristic of endometriosis, was evidenced by the significant downregulation of PGR in GSE7305 (log_2_FC = −2.436) and the favorable docking of the coumarin marmesin to the progesterone receptor (−7.25 kcal/mol; Kd = 4.84 μM). However, PGR downregulation was not statistically significant in the second dataset, making this axis a plausible but less strongly supported element of the proposed mechanism that warrants focused experimental investigation.

Several considerations qualify the translational interpretation of these findings. The oral bioavailability of green tea catechins is typically low due to limited absorption and extensive phase-II metabolism [52]. Consequently, achieving lesion-relevant concentrations in vivo may require formulation strategies or prodrug approaches. Additionally, the predicted hepatotoxicity associated with galloylated catechins warrants careful evaluation of dosing, as high doses of EGCG have been linked to hepatic injury [53]. The transcriptomic validation is based on modestly sized, publicly archived microarray datasets [54]. Although control for menstrual-cycle phase was implemented, and reproducibility across platforms was confirmed, larger RNA-sequencing cohorts stratified by lesion subtype would provide stronger inferences. The two validation datasets differ in lesion context: GSE7305 comprises ovarian endometrioma versus normal endometrium, whereas GSE25628 comprises ectopic endometriotic lesions [55] with paired eutopic and normal endometrium. Both share an ectopic-lesion-versus-normal-endometrium design, making them appropriate cross-validation sets; rather than a limitation, the use of two independent datasets differing in precise lesion subtype strengthens the analysis, since targets reproducible across platforms and lesion contexts—most notably ESR1—are more likely to reflect robust, subtype-independent features of endometriosis. Nonetheless, lesion subtype and anatomical site were not uniformly annotated across datasets, and larger RNA-sequencing cohorts stratified by lesion subtype would provide stronger inferences.

Despite these limitations, a principal strength of this study resides in its convergent design. By anchoring the network in an experimentally measured phytochemical profile and cross-validating predicted targets against independent patient transcriptomes prior to docking, we significantly reduce the speculative distance that typically separates conventional network-pharmacology reports from disease biology. The resulting hypotheses are concrete and testable. Future research priorities should include in vitro validation of ESR1 modulation and aromatase inhibition by ECG and EGCG in endometriotic stromal cells, functional interrogation of the EGCG–EP300/CREBBP–HIF-1 epigenetic axis, and assessment of standardized *C. sinensis* extracts—both independently and as adjuncts to existing hormonal therapies—in preclinical models, with a particular emphasis on enhancing bioavailability through formulation strategies.

## 5. Conclusions

Integrating UHPLC–QTOF–MS profiling, network pharmacology, transcriptomic cross-validation, and docking, this study models how *C. sinensis* polyphenols may act against endometriosis through a dual endocrine–inflammatory mechanism. The 20 measured constituents converged on 292 endometriosis targets, poly-pharmacologically distributed across the panel rather than driven by any single compound. TP53 emerged as the most interconnected network hub, with SRC, STAT3, AKT1 and ESR1 forming the central cluster, while PTGS2 and CYP19A1 acted as bridges between the estrogenic and inflammatory modules. ESR1 emerged as a central, consistently down-regulated node, with transcriptomic down-regulation in ectopic tissue across two independent datasets; because ERβ (ESR2) was not measured here, we consider ESR1 a disease-associated, potentially modifiable target rather than a demonstrated driver. At the compound level, EGCG was the only constituent computationally predicted through target prediction and molecular docking to interact with the coactivators EP300/CREBBP and TP53. This suggests a hypothetical epigenetic mode of action that complements the receptor/enzyme targeting (ESR1, CYP19A1, PTGS2, AKT1) predicted across the broader catechin–flavonoid panel. These interactions are predicted rather than experimentally validated, and the docking results were consistent with them (e.g., ECG–ESR1, marmesin–PGR, EGCG–EP300). These hypothesis-generating findings nominate specific, testable targets, particularly ESR1, CYP19A1, and the EGCG–EP300/CREBBP epigenetic axis, for green-tea-based anti-endometriotic strategies.

## Figures and Tables

**Figure 1:**
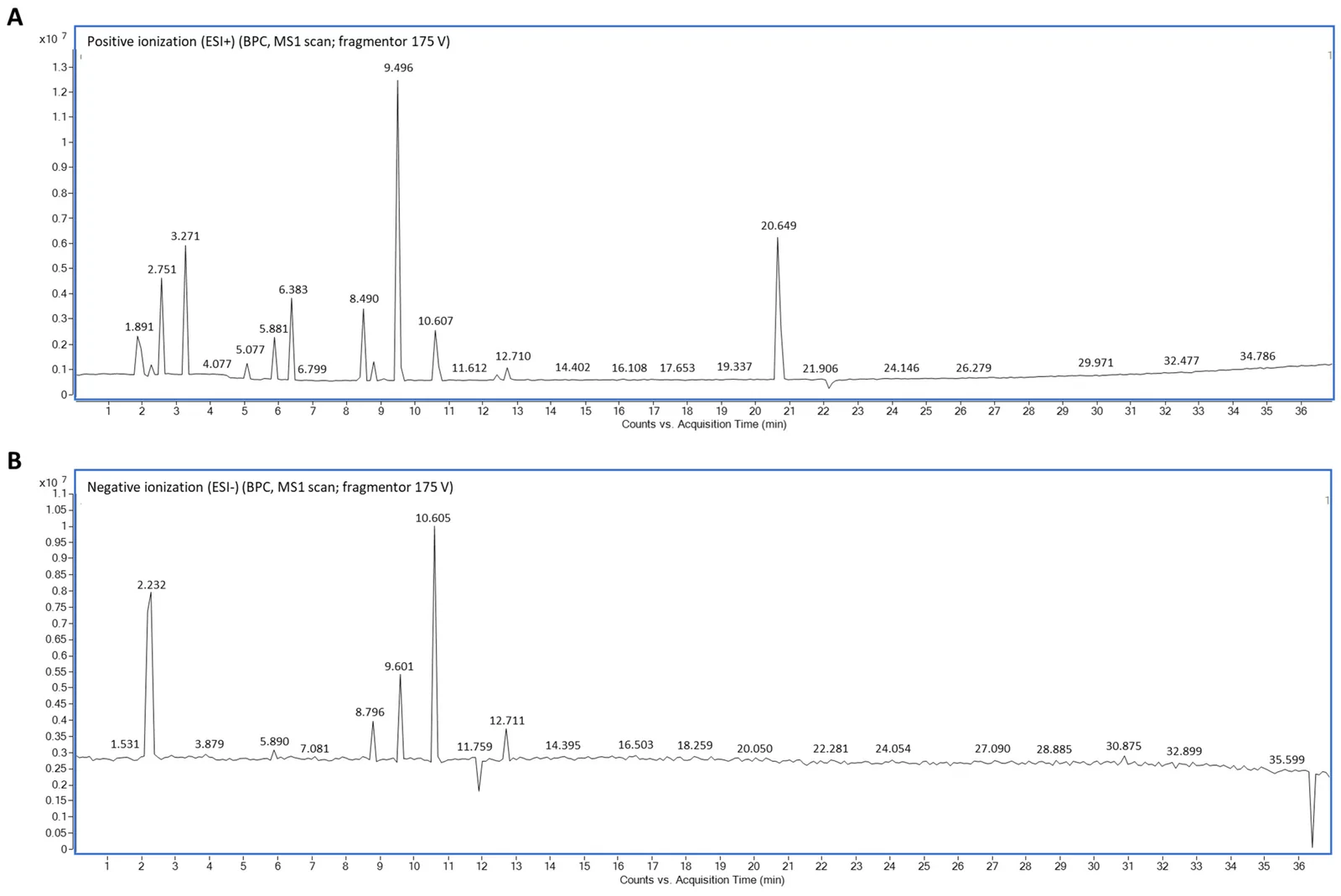
Base peak chromatograms (BPC, MS1 scan; fragmentor 175 V) of the *C. sinensis* extract acquired by UHPLC–QTOF-MS in (**A**) positive (ESI+) and (**B**) negative (ESI−) ionization modes.

**Figure 2:**
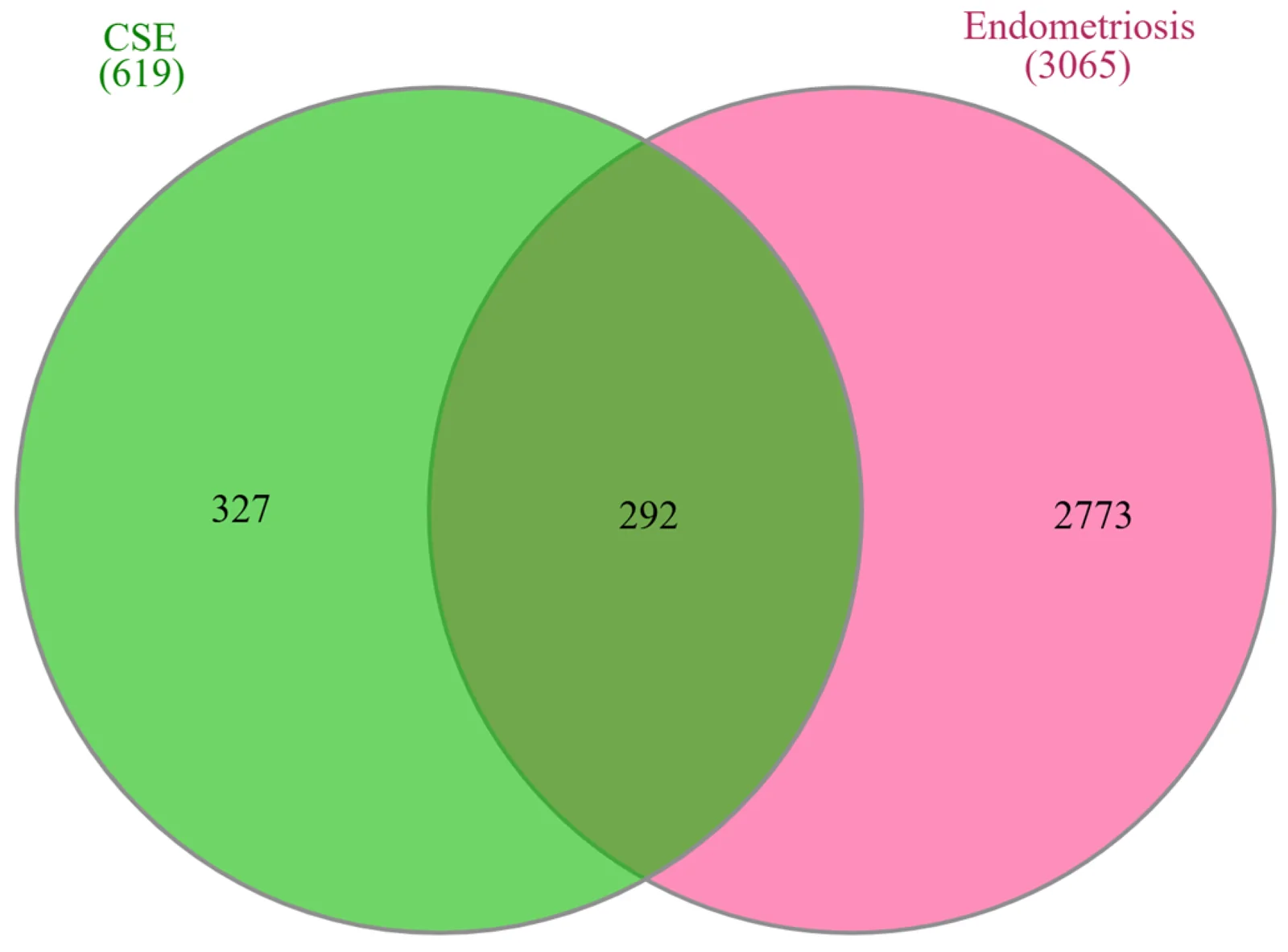
Venn diagram of the intersection between predicted CSE compound targets (n = 619) and curated endometriosis-associated genes (n = 3065), yielding 292 common targets.

**Figure 3:**
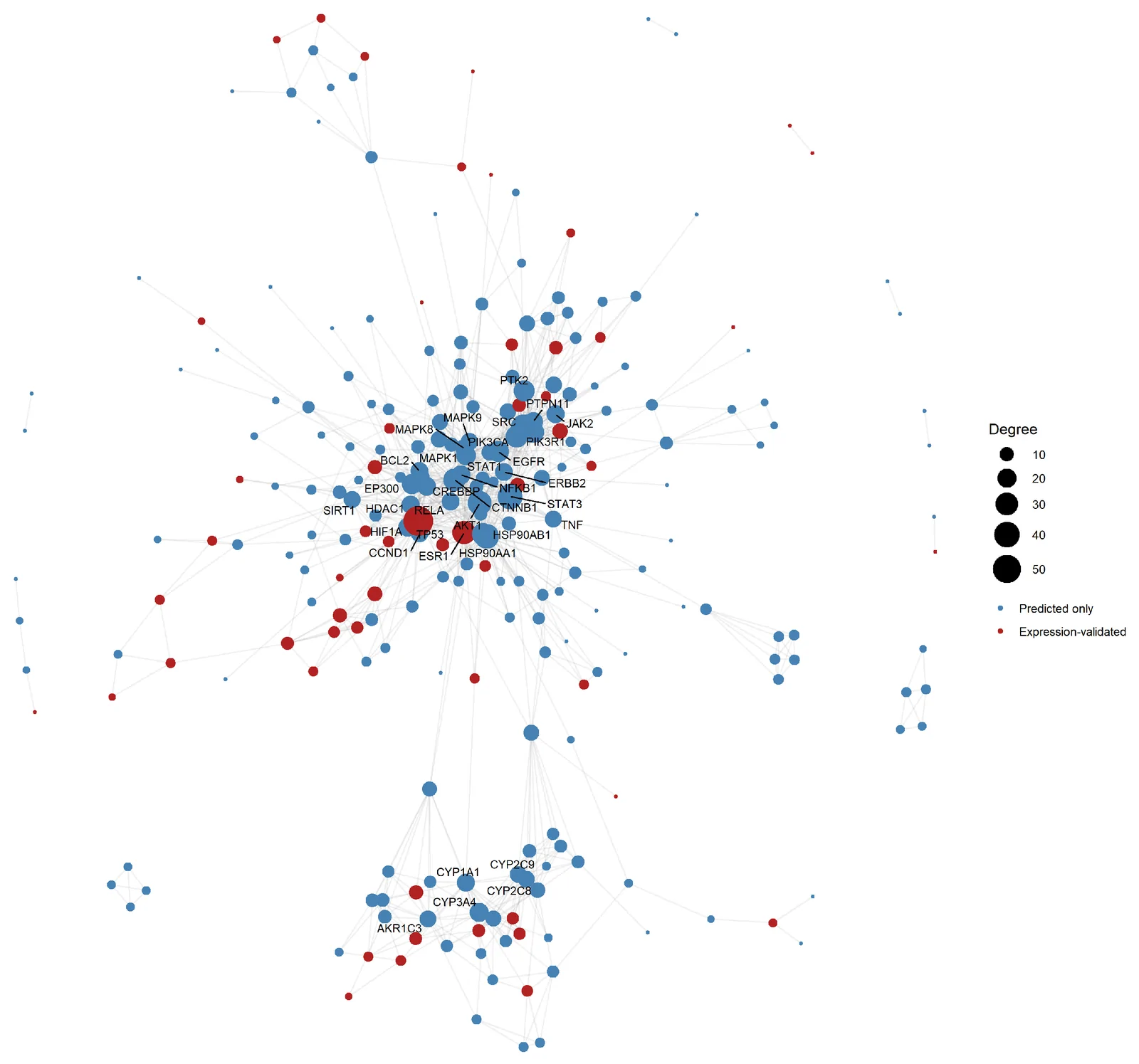
Protein–protein interaction network of the 292 common targets. Node size is proportional to degree centrality; red nodes indicate expression-validated targets, blue nodes indicate predicted-only targets. TP53, SRC, STAT3, HSP90AA1, AKT1, and ESR1 constitute the dominant central hub cluster; a peripheral cytochrome P450 (CYP)-enriched cluster is visible in the lower portion of the network.

**Figure 4:**
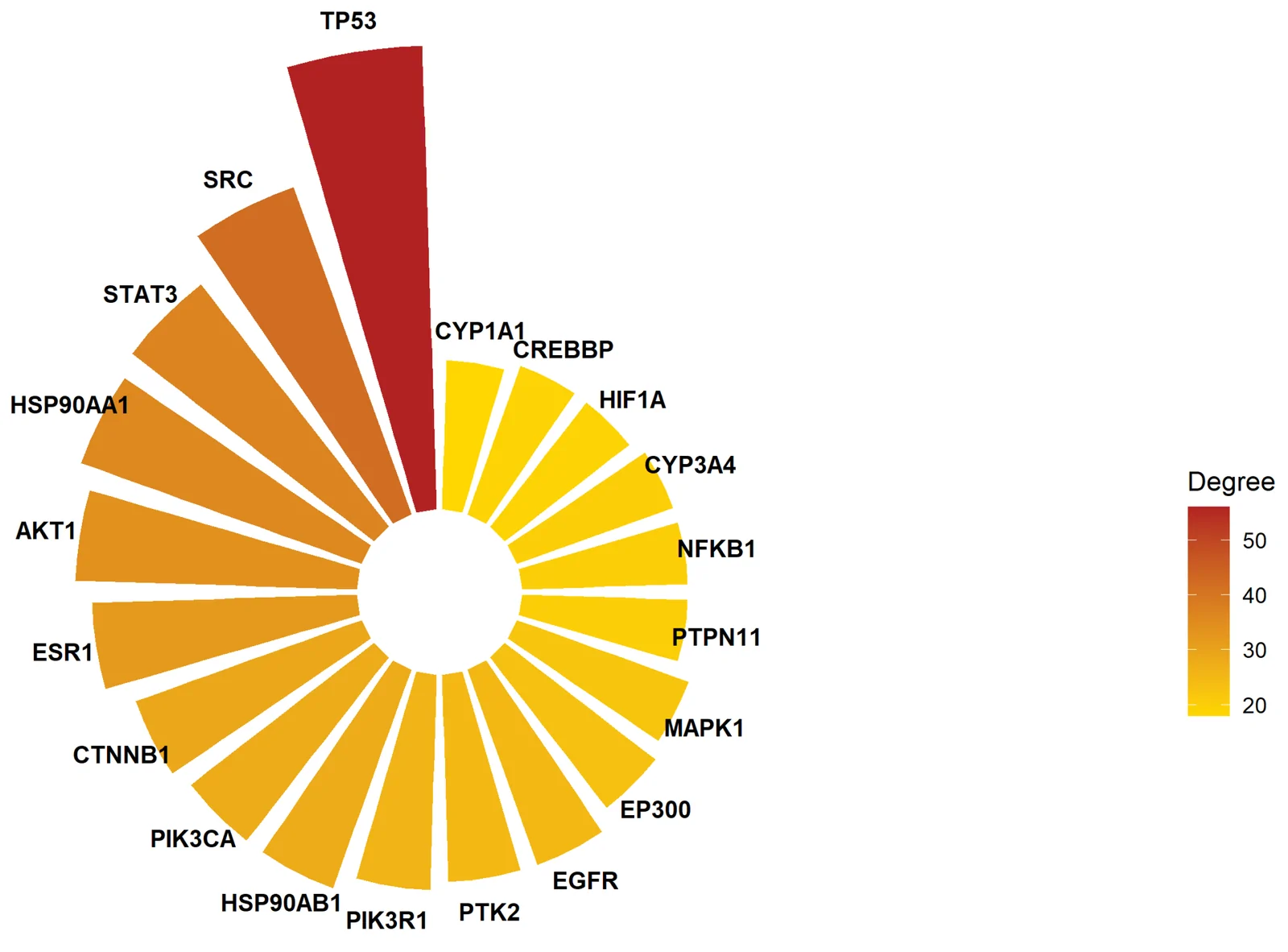
Top 20 hub genes of the 292-gene protein–protein interaction network, ranked by degree centrality.

**Figure 5:**
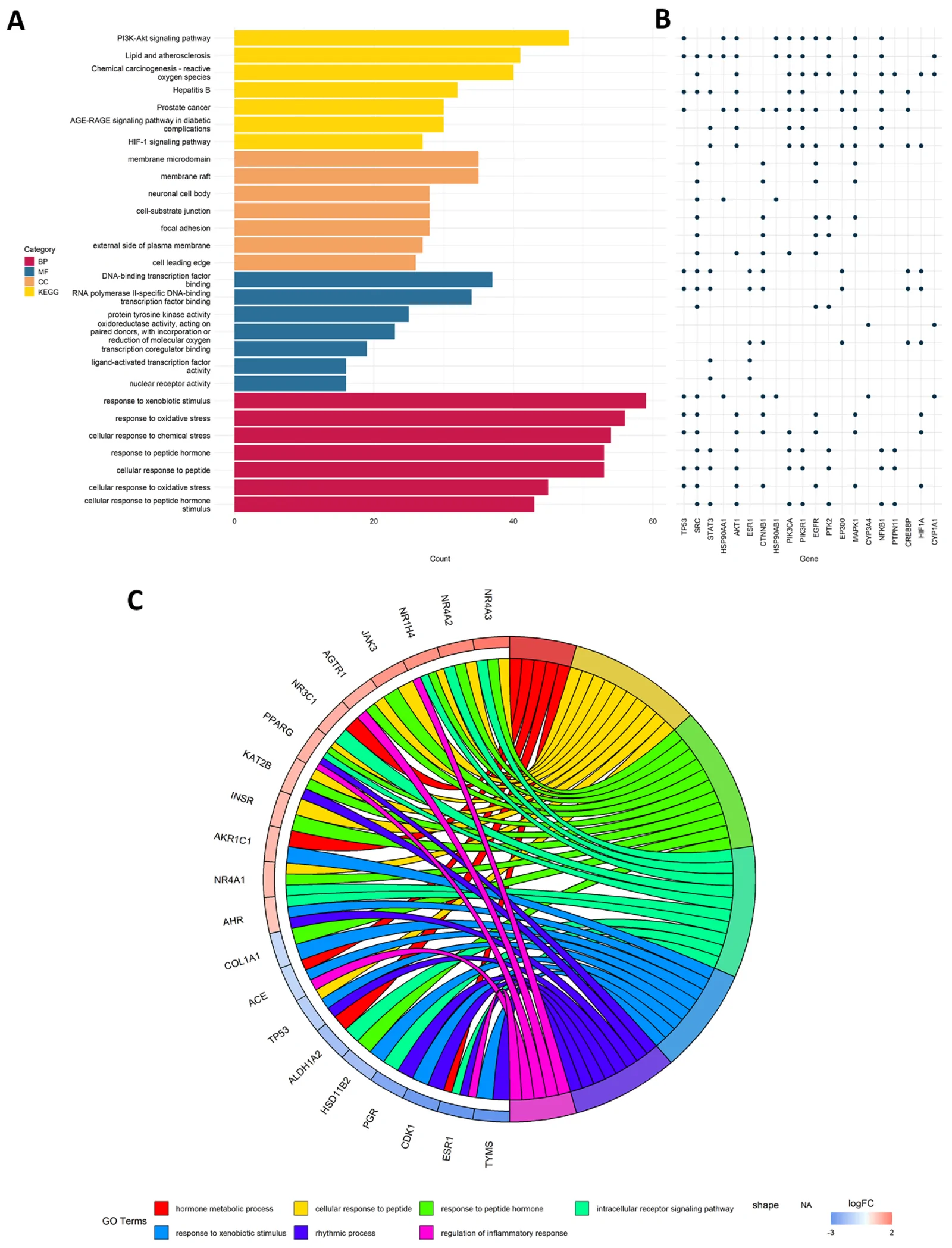
Functional enrichment of the 292 common targets. (**A**) Top enriched GO (BP, CC, MF) and KEGG terms, coloured by category and ranked by gene count. (**B**) Membership matrix of the leading hub genes across the enriched GO/KEGG categories. (**C**) Gene–GO Biological Process chord diagram; left arcs are genes coloured by GSE7305 log_2_ fold-change (blue = down-, red = upregulated in ectopic vs. normal endometrium), right arcs are the leading enriched BP terms. Enrichment statistics (gene ratio, fold enrichment, *p*-value, and Benjamini–Hochberg adjusted *p*-value) for all terms shown are provided in Supplementary Table S9.

**Figure 6:**
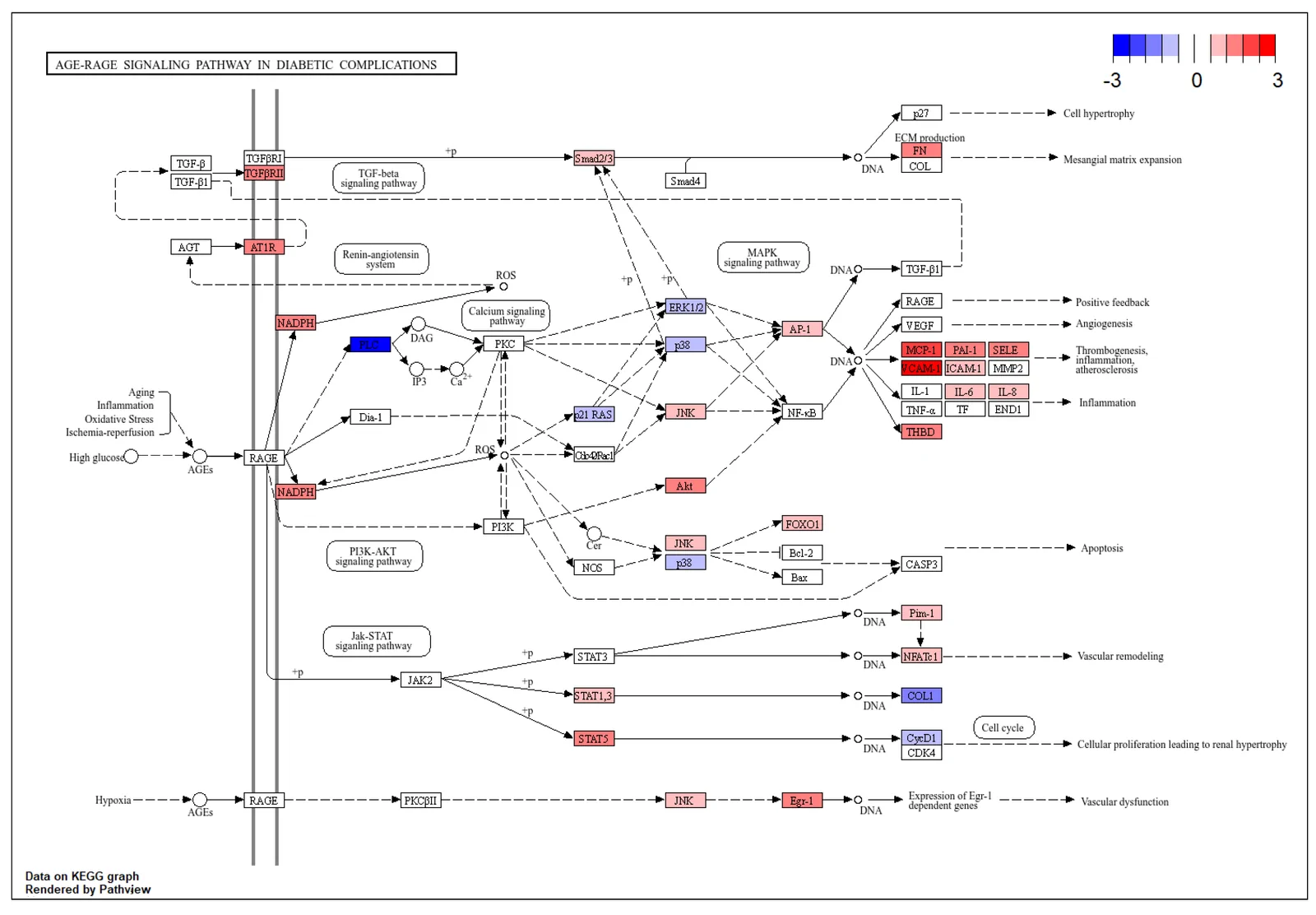
AGE-RAGE signaling pathway in diabetic complications (KEGG hsa04933), with pathway components colored by real log2 fold-change values from GSE7305 differential expression analysis (red = upregulated, blue = downregulated in ectopic vs. normal endometrium).

**Figure 7:**
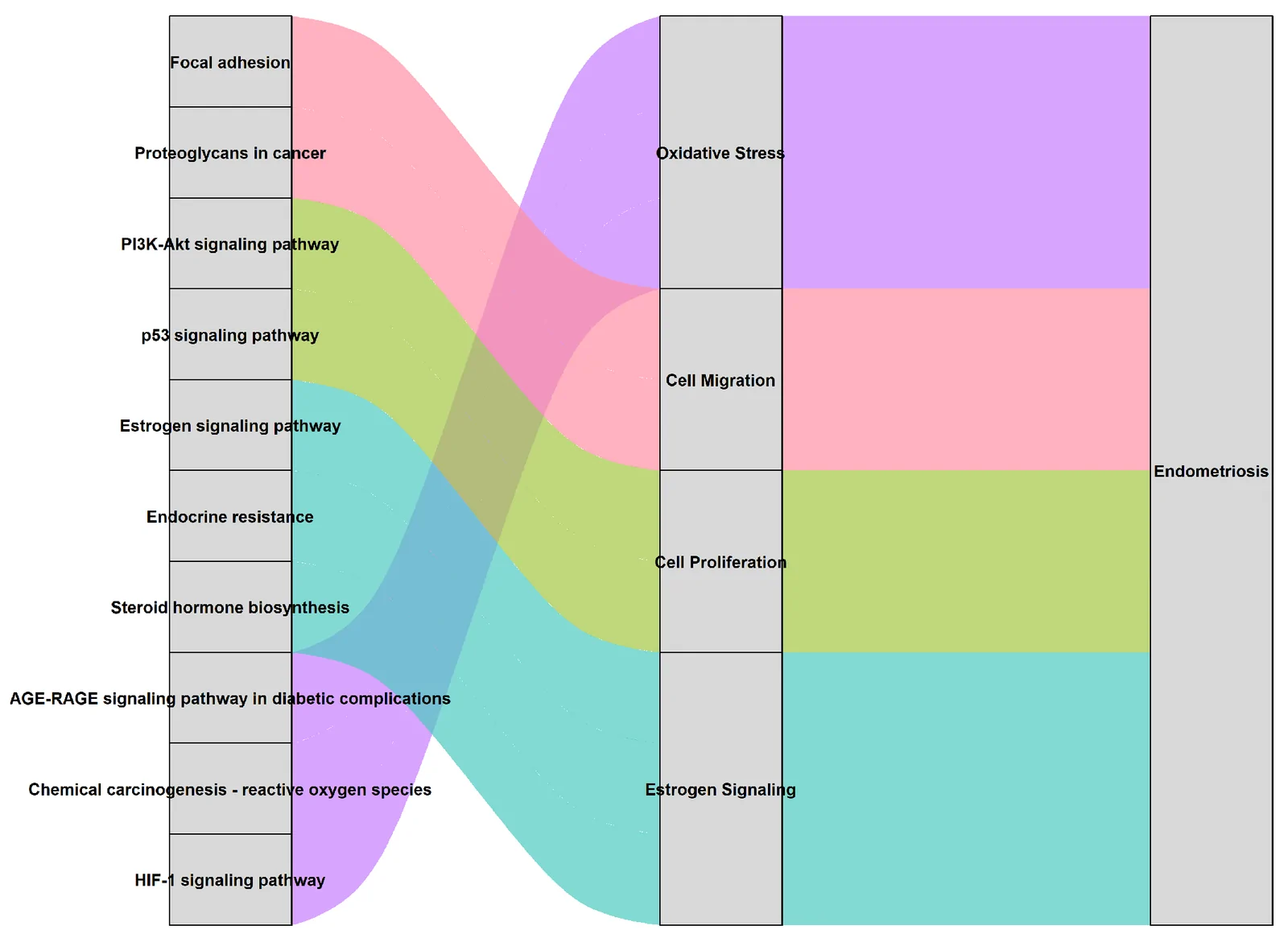
Sankey diagram integrating the enriched KEGG pathways (**left**) into four mechanistic themes—oxidative stress, cell migration, cell proliferation, and estrogen signaling (**center**)—that converge on endometriosis (**right**).

**Figure 8:**
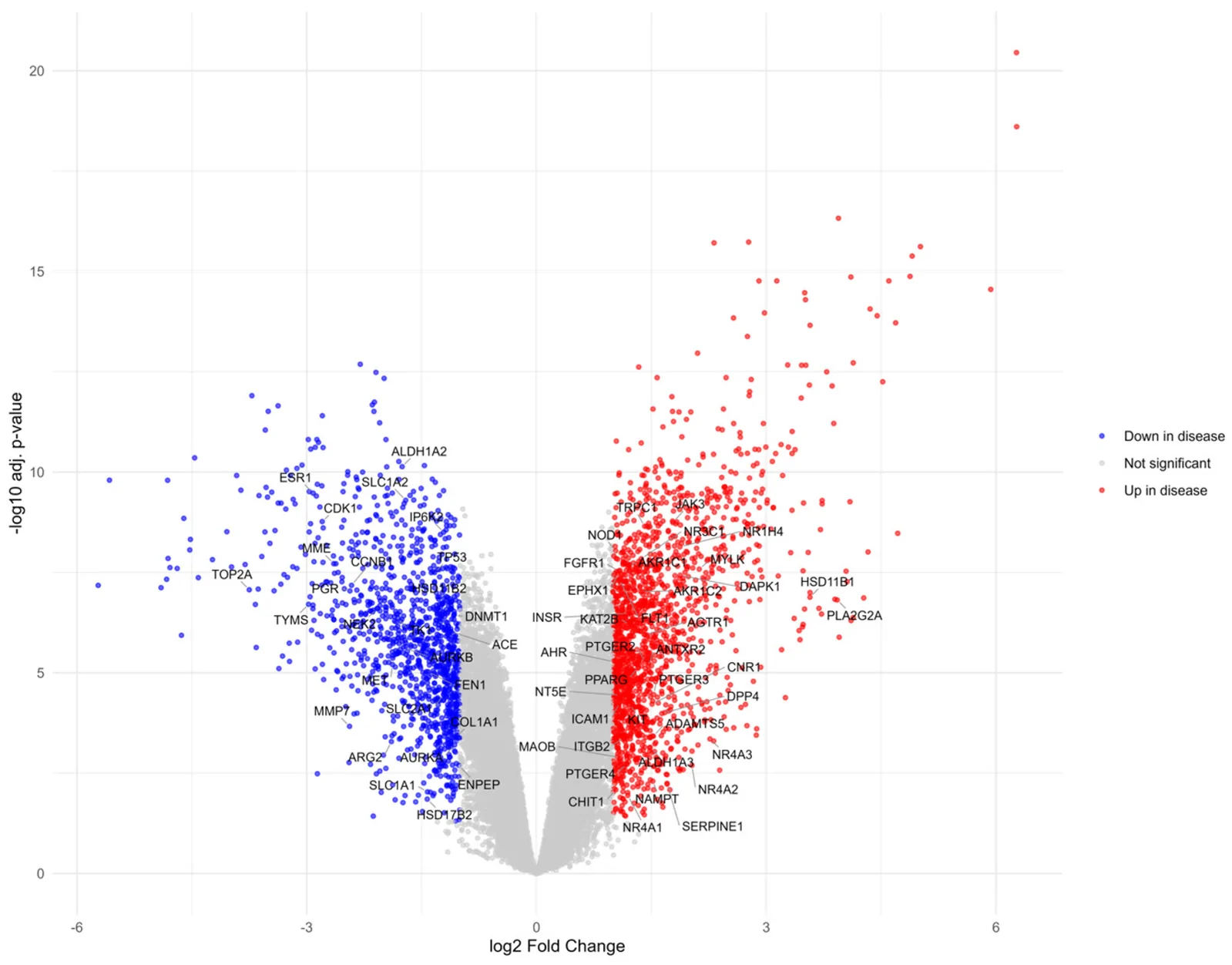
Volcano plot of differential gene expression in GSE7305 (ectopic vs. normal endometrium). Red points indicate significantly upregulated genes, blue points indicate significantly downregulated genes (|log2FC| > 1, adjusted *p* < 0.05).

**Figure 9:**
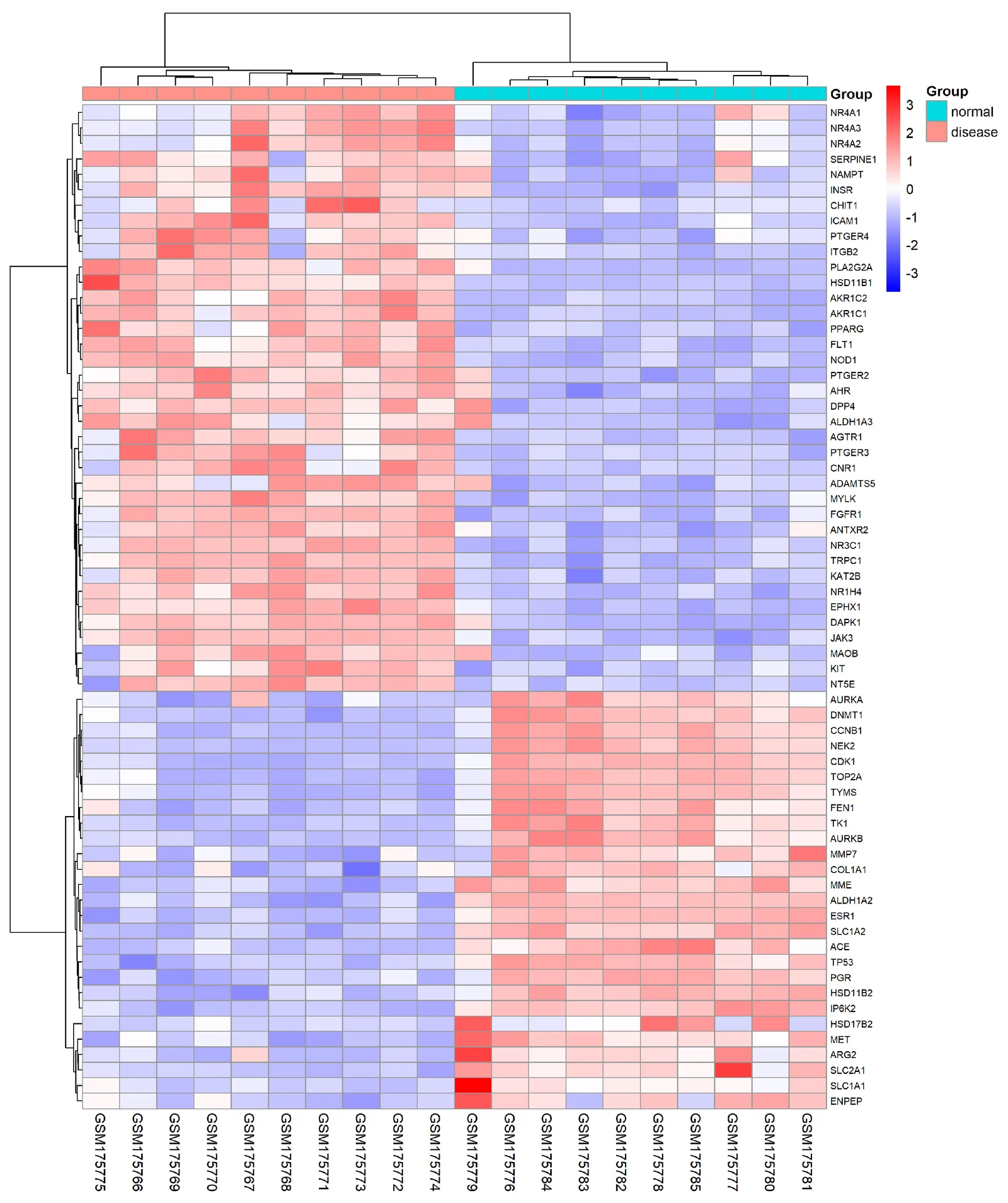
Heatmap of the 65 expression-validated targets across all 20 GSE7305 samples (10 disease, 10 normal), demonstrating complete separation of disease and normal samples by unsupervised hierarchical clustering.

**Figure 10:**
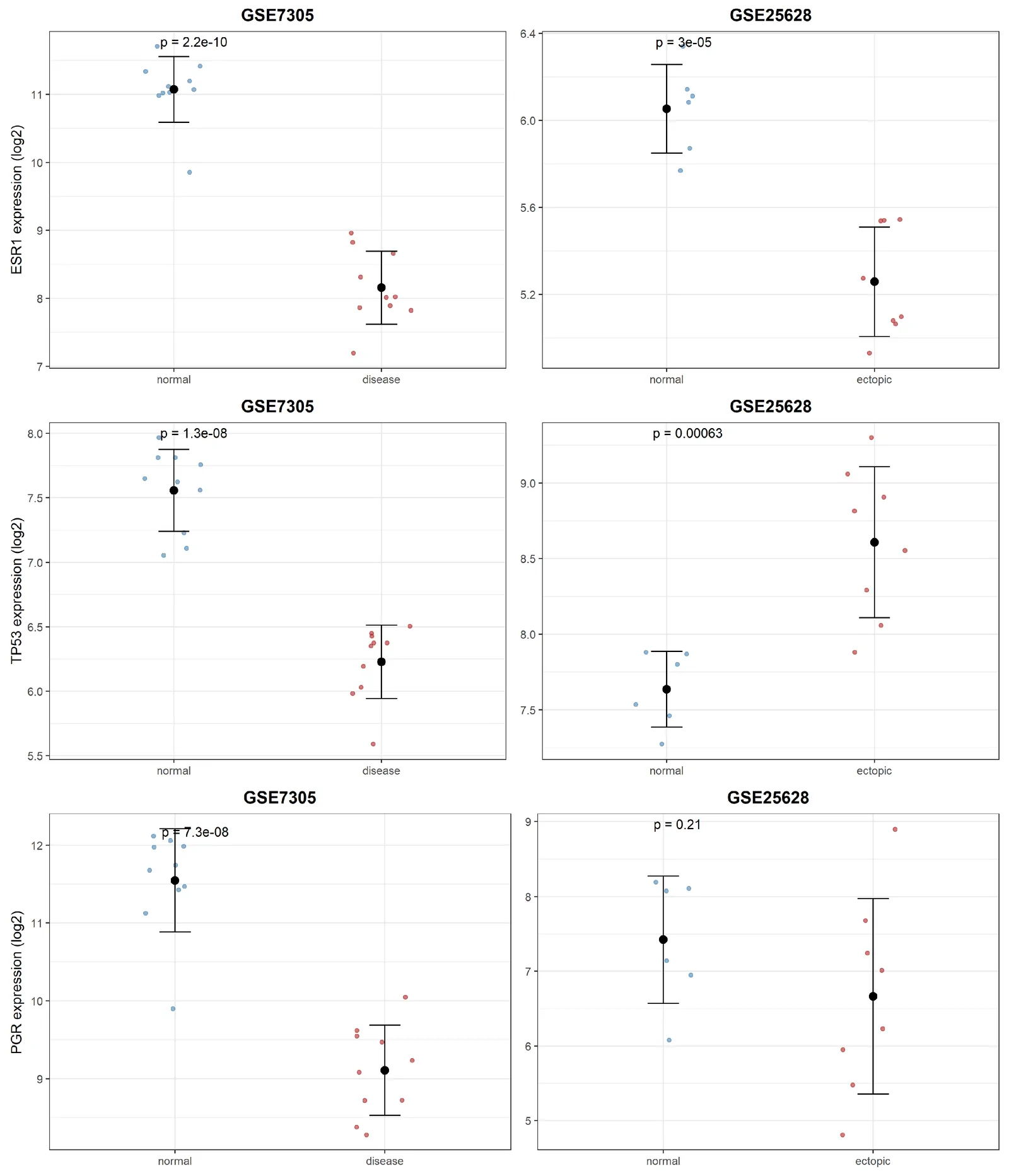
Cross-dataset expression validation of TP53, ESR1, and PGR across two independent endometriosis datasets (GSE7305, (**left**); GSE25628, (**right**)). Points represent individual samples; error bars represent mean ± SD.

**Figure 11:**
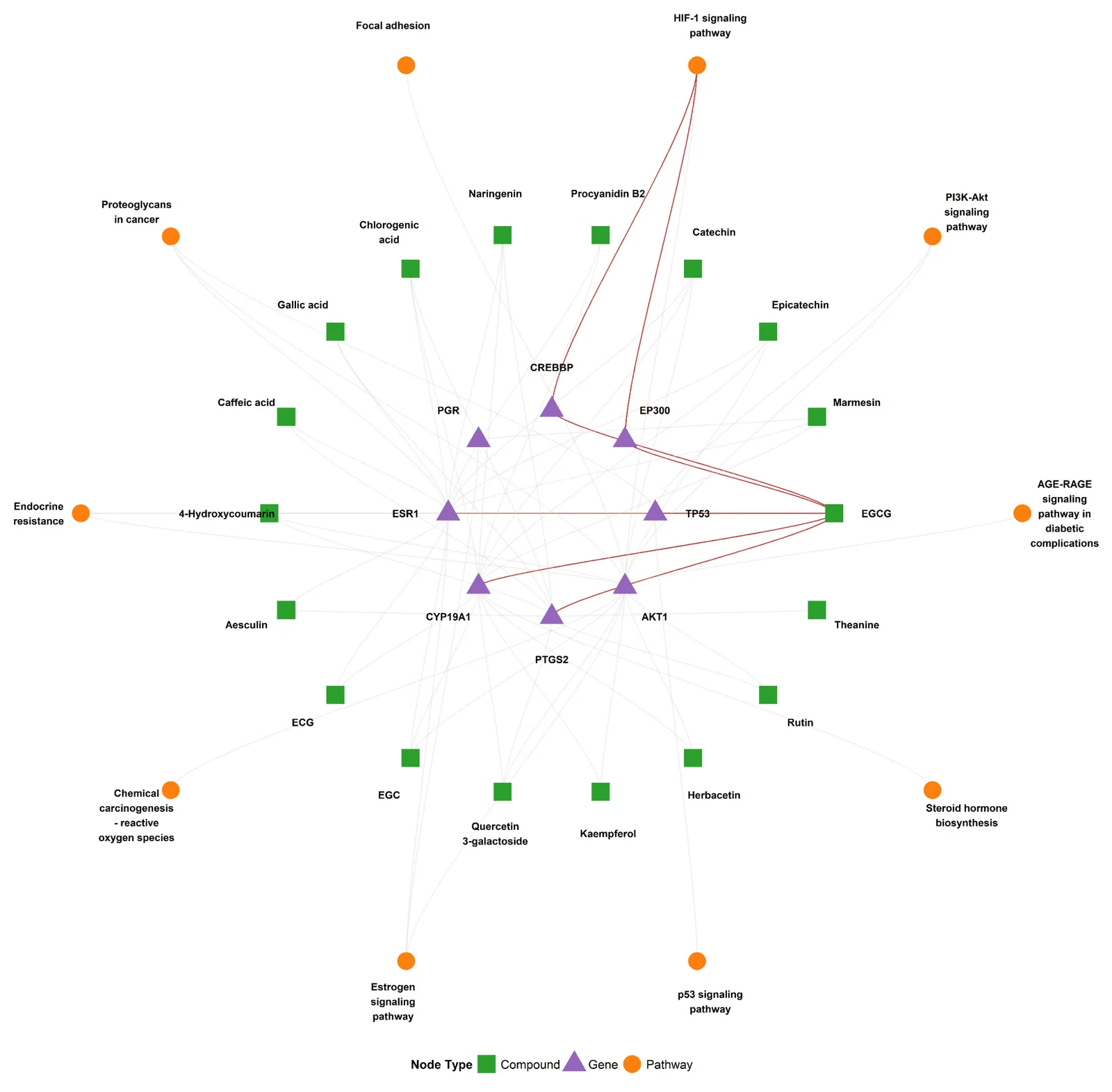
Compound-target-pathway network. Green squares represent the 20 compounds, purple triangles represent the 8 prioritized docking targets, orange circles represent curated KEGG pathways. Red-highlighted edges indicate connections unique to EGCG and its targets EP300/CREBBP.

**Figure 12:**
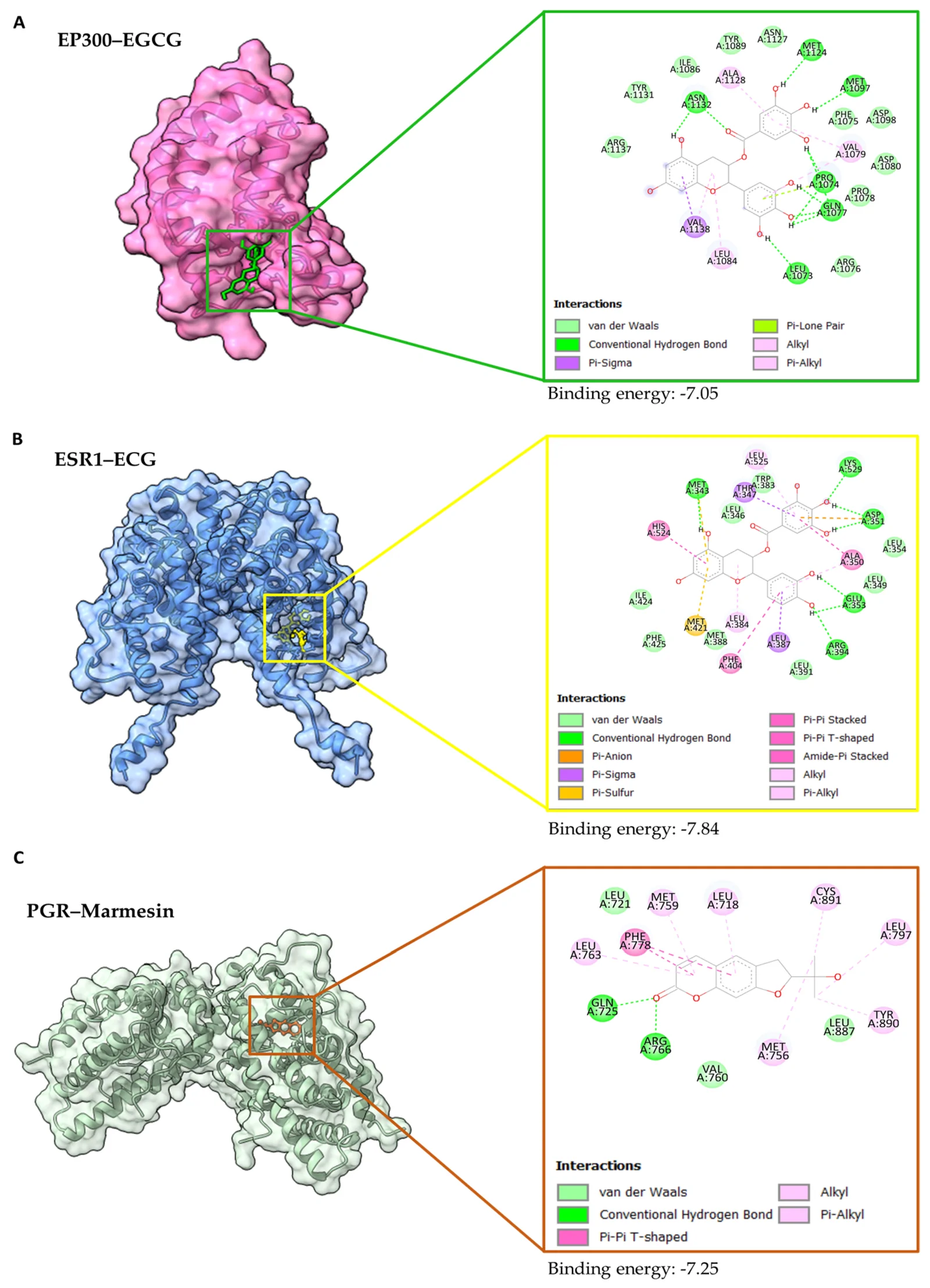
The docking complexes of the top-ranked *C. sinensis* compound (3D, 2D). (**A**) EGCG–EP300 (−7.05 kcal/mol); (**B**) ECG–ESR1 (−7.84 kcal/mol); (**C**) Marmesin–PGR (−7.25 kcal/mol).

**Table 1:** Putative identification of the twenty selected phytochemicals in the *C. sinensis* extract by UHPLC–QTOF-MS in positive and negative ionization modes.

	Compound	Formula	RT (min)	Observed *m*/*z*	Theoretical *m*/*z*	Mass Error (ppm)	AreaSum (%)	ESI+ (Adduct, *m*/*z*)	ESI− (Adduct, *m*/*z*)
1	Epigallocatechin Gallate (EGCG)	C_22_H_18_O_11_	10.930	457.0773	457.0776	0.75	5.99	n.d.	[M − H]^−^ 457.0773
2	Epicatechin	C_15_H_14_O_6_	10.654	289.0731	289.0720	−3.69	25.91	[M + H]^+^ 291.0868	[M − H]^−^ 289.0731
3	(±)-Catechin	C_15_H_14_O_6_	9.675	289.0721	289.0717	−1.18	9.20	[M + H]^+^ 291.0871	[M − H]^−^ 289.0721
4	Rutin	C_27_H_30_O_16_	11.984	609.1457	609.1462	0.80	7.08	n.d.	[M − H]^−^ 609.1457
5	Quercetin 3-galactoside	C_21_H_20_O_12_	12.537	463.0880	463.0883	0.62	7.49	[M + Na]^+^ 487.0852	[M − H]^−^ 463.0880
6	Procyanidin B2	C_30_H_26_O_12_	9.926	577.1351	577.1353	0.31	3.06	[M + H]^+^ 579.1492	[M − H]^−^ 577.1351
7	(−)-Naringenin	C_15_H_12_O_5_	12.807	273.0761	273.0758	−1.21	0.88	[M + H]^+^ 273.0761	n.d.
8	Kaempferol	C_15_H_10_O_6_	13.460	287.0554	287.0549	−1.46	0.18	[M + H]^+^ 287.0554	n.d.
9	Chlorogenic acid	C_16_H_18_O_9_	8.069	353.0875	353.0879	1.01	1.47	n.d.	[M − H]^−^ 353.0875
10	Gallic acid	C_7_H_6_O_5_	12.887	169.0142	169.0142	0.24	0.70	n.d.	[M − H]^−^ 169.0142
11	Caffeic acid	C_9_H_8_O_4_	9.574	179.0349	179.0350	0.40	0.40	n.d.	[M − H]^−^ 179.0349
12	Herbacetin	C_15_H_10_O_7_	12.506	303.0503	303.0500	−1.25	0.70	[M + H]^+^ 303.0503	n.d.
13	Caffeine	C_8_H_10_N_4_O_2_	9.470	195.0902	195.0877	−13.26	16.87	[M + H]^+^ 195.0902	n.d.
14	Theanine	C_7_H_14_N2O_3_	3.351	173.0932	173.0931	−0.22	2.37	n.d.	[M − H]^−^ 173.0932
15	Theophylline	C_7_H_8_N_4_O_2_	7.110	181.0723	181.0719	−1.89	0.23	[M + H]^+^ 181.0723	n.d.
16	Marmesin	C_14_H_14_O_4_	10.654	245.0821	245.0820	−0.40	1.17	n.d.	[M − H]^−^ 245.0821
17	4-Hydroxycoumarin	C_9_H_6_O_3_	8.064	163.0392	163.0388	−2.34	0.89	[M + H]^+^ 163.0392	n.d.
18	Aesculin	C_15_H_16_O_9_	8.546	339.0718	339.0722	1.28	0.12	n.d.	[M − H]^−^ 339.0718
19	Epicatechin Gallate (ECG)	C_22_H_18_O_10_	12.838	441.0825	441.0827	0.36	10.35	[M + H]^+^ 443.0974	[M − H]^−^ 441.0825
20	Epigallocatechin (EGC)	C_15_H_14_O_7_	7.567	305.0670	305.0668	−0.95	4.94	[M + H]^+^ 307.0812	[M − H]^−^ 305.0670

Note: n.d. not detected.

**Table 2:** ADMET and drug-likeness profiling of the twenty *C. sinensis* metabolites.

	Compound	MW (g/mol)	cLogP	HBD	HBA	TPSA (Å^2^)	Lipinski Viol.
1	(−)-Epigallocatechin gallate (EGCG)	458.4	0.8	8	11	197.37	1
2	Epicatechin	290.27	0.7	5	6	110.38	0
3	(±)-Catechin	290.27	0.7	5	6	110.38	0
4	Rutin	610.5	−2.74	10	16	269.43	1
5	Quercetin 3-galactoside	464.4	−1.34	8	12	210.51	1
6	Procyanidin B2	578.5	1.4	10	12	220.76	1
7	(−)-Naringenin	272.25	1.87	3	5	86.99	0
8	Kaempferol	286.24	1.2	4	6	111.13	0
9	Chlorogenic acid	354.31	−0.71	6	9	164.75	0
10	Gallic acid	170.12	0.21	4	5	97.99	0
11	Caffeic acid	180.16	0.92	3	4	77.76	0
12	Herbacetin	302.23	0.79	5	7	131.36	0
13	Caffeine	194.19	−1.71	0	3	61.82	0
14	Theanine	174.20	−1.93	3	4	92.42	0
15	Theophylline	180.16	−1.42	1	3	72.68	0
16	Marmesin	246.26	1.28	1	4	59.67	0
17	4-Hydroxycoumarin	162.14	0.85	1	3	50.44	0
18	Aesculin	340.28	−1.8	5	9	149.82	0
19	Epicatechin Gallate (ECG)	442.4	1.21	7	10	177.14	0
20	Epigallocatechin (EGC)	306.27	0.28	6	7	130.61	0

Note: MW molecular weight (g/mol), HBD hydrogen bond donors, HBA hydrogen bond acceptors.

**Table 3:** Molecular docking investigations of the phytocompounds of *C. sinensis* with target enzymes of TP53, EP300, CREBBP, PGR, ESR1, CYP19A1, PTGS2 and AKT1.

Target [PDB]	Compound	Binding Energy (kcal/mol)	Kd	Hydrogen Bond (Length Å)
Tumor protein p53 (TP53) [2OCJ]	Epigallocatechin Gallate (EGCG)	−4.67	379.10 µM	GLU-221 (4.46), GLU-224 (3.91), CYS-229 (4.11, 3.91), THR-231 (3.80, 3.82, 4.05), GLY-199 (3.75)
E1A-binding protein p300 (EP300) [5BT3]	Epigallocatechin Gallate (EGCG)	−7.05	6.83 µM	ASN-1132 (4.24, 4.87), LEU-1073 (5.16), MET-1124 (4.19), MET-1097 (5.77), PRO-1074 (3.74, 5.18), GLN-1077 (2.92, 3.27, 5.59, 5.78)
CREB-binding protein (CREBBP) [3DWY]	Epigallocatechin Gallate (EGCG)	−5.18	159.71 µM	ASP-1134 (3.45), ASP-1116 (4.05, 4.84), MET-1133 (5.30)
Progesterone receptor (PGR) [1A28]	Marmesin	−7.25	4.84 µM	ARG-766 (5.90), GLN-725 (5.45)
Estrogen receptor alpha (ESR1) [1A52]	Epigallocatechin Gallate (EGCG)	−7.08	6.49 µM	GLU-353 (5.03), LYS-529 (6.07), ASP-351 (4.02, 4.50), GLY-521 (3.22), HIS-524 (2.99)
	Epicatechin	−6.95	7.98 µM	GLU-353 (4.47, 4.61), GLY-521 (3.37)
	Catechin	−6.62	14.03 µM	HIS-524 (3.91), GLY-521 (3.67), GLU-353 (4.64, 4.68)
	Chlorogenic acid	−7.28	4.60 µM	GLU-353 (4.60, 4.80), GLY-521 (3.37)
	Epicatechin Gallate (ECG)	−7.84	1.80 µM	MET-343 (4.20), ARG-394 (7.03), GLU-353 (4.62, 4.90), ASP-351 (3.96, 4.54), LYS-529 (6.33)
	Epigallocatechin (EGC)	−5.93	45.29 µM	LEU-387 (3.26), GLU-353 (4.21), GLY-521 (3.00, 3.55), HIS-524 (3.22)
Aromatase (CYP19A1) [3EQM]	Epigallocatechin Gallate (EGCG)	−6.47	18.21 µM	PHE-430 (5.82), PRO-429 (5.14), GLU-302 (4.31), ASP-309 (3.33, 3.68), LEU-477 (3.69, 3.97), LEU-372 (3.76), MET-374 (3.79)
	Epicatechin	−6.01	39.64 µM	ASP-309 (3.32), ARG-115 (3.85), MET-374 (3.00), LEU-477 (3.58)
	Catechin	−6.11	32.96 µM	ASP-309 (3.24, 3.33), LEU-477 (3.31)
	Quercetin 3-galactoside	−6.19	28.86 µM	PRO-429 (4.70), LEU-477 (3.97), LEU-372 (4.77), MET-374 (3.90), ASP-309 (3.65)
	Epicatechin Gallate (ECG)	−6.24	26.85 µM	ALA-438 (4.87), ASP-309 (3.34, 3.74), PRO-429 (5.11, 5.11), LEU-477 (3.70)
	Epigallocatechin (EGC)	−4.71	352.76 µM	ARG-115 (3.76), LEU-477 (3.74), MET-374 (2.83), ALA-306 (3.19)
Prostaglandin-endoperoxide synthase 2 (PTGS2) [5KIR]	Epigallocatechin Gallate (EGCG)	−7.23	5.00 µM	ARG-120 (4.13), ARG-513 (3.67), GLN-192 (5.23), SER-530 (3.52, 3.74)
	Rutin	−2.38	17.92 mM	ARG-120 (4.25), TYR-355 (4.47), SER-353 (3.24), GLN-192 (4.92), SER-530 (3.93), VAL-349 (3.22)
	Quercetin 3-galactoside	−6.26	25.67 µM	PHE-518 (2.96), LEU-352 (3.65), SER-530 (3.80), TYR-385 (6.02)
	Chlorogenic acid	−7.46	3.39 µM	GLN-192 (4.94, 5.07), PHE-518 (3.55), ALA-527 (3.57), SER-530 (3.34, 3.68), TYR-385 (6.16)
	Gallic acid	−3.97	1.23 mM	PHE-518 (3.80), GLN-192 (5.31), ARG-513 (5.76), HIS-90 (4.12)
RAC-alpha serine/threonine-protein kinase (AKT1) [4EJN]	Epicatechin	−7.31	4.36 µM	ILE-290 (4.53), THR-211 (3.65, 4.00), ASP-292 (3.24), SER-205 (3.71), GLN-203 (3.99, 4.42)
	Catechin	−7.30	4.49 µM	ASP-292 (3.70), THR-211 (2.66, 3.07), LYS-268 (4.34), GLN-203 (3.55)
	Quercetin 3-galactoside	−7.24	4.91 µM	SER-205 (3.30, 3.33, 4.57), TRP-80 (5.54), THR-211 (3.76), ASP-292 (4.54), THR-82 (4.14)
	Epigallocatechin (EGC)	−6.29	24.34 µM	ILE-290 (4.40), THR-211 (3.66), ASP-292 (3.22), SER-205 (3.80), GLN-203 (4.13)
	Kaempferol	−6.67	13.01 µM	ASP-292 (4.00), THR-290 (4.59), LYS-268 (4.03), GLN-203 (3.89)

## Data Availability

All data generated or analyzed during this study are included in this published article.

## Supplementary Materials

The following supporting information can be downloaded at: https://www.mdpi.com/article/10.3390/life16101613/s1, Table S1: Identification of the 20 *C. sinensis* compounds selected for network analysis; Table S2: Complete list of 159 accurate-mass features detected in *C. sinensis* extract; Table S3: Predicted Pharmacokinetic and Toxicological Properties of *C. sinensis* Bioactive Compounds; Table S4: Genes from DisGenet, Gene Cards, OMIM; Table S5: Per-compound contribution to the 292 shared targets; Table S6: 292 putative therapeutic target genes of CSE against Endometriosis; Table S7: 20 hub genes according to degree and betweenness; Table S8: Top 10 hub genes of the 292-target PPI network ranked independently by three cytoHubba centrality algorithms; Table S9: Enrichment statistics (gene ratio, fold enrichment, *p*-value, and Benjamini–Hochberg adjusted *p*); Figure S1: A protein interaction network diagram of active components and disease targets (292) of CSE was constructed; Figure S2: Expression of key hub genes across the three GSE25628 groups.

## Abbreviations

The following abbreviations are used in this manuscript:

ADMET	Absorption, distribution, metabolism, excretion, and toxicity
AGE–RAGE	Advanced glycation end products–receptor for advanced glycation end products
AHR	Aryl hydrocarbon receptor
AJS-ESI	Agilent Jet Stream electrospray ionization
AKT1	AKT serine/threonine kinase 1 (RAC-alpha serine/threonine-protein kinase)
Ames	Ames bacterial reverse-mutation (mutagenicity) assay
AP-1	Activator protein 1
AT1R/AGTR1	Angiotensin II type 1 receptor
BP	Biological process (Gene Ontology)
CASTp	Computed Atlas of Surface Topography of proteins
CC	Cellular component (Gene Ontology)
cLogP	Calculated logarithm of the octanol–water partition coefficient
COX-2	Cyclooxygenase-2
CREBBP	CREB-binding protein
CSE	*Camellia sinensis* extract
CYP	Cytochrome P450
CYP19A1	Cytochrome P450 family 19 subfamily A member 1
DEG	Differentially expressed gene
DILI	Drug-induced liver injury
EP300	E1A-binding protein p300
ERα/ERβ	estrogen receptor alpha/beta
ERK1/2	extracellular signal-regulated kinase 1/2
ESI (+/−)	electrospray ionization (positive/negative mode)
ESR1	estrogen receptor 1 (estrogen receptor alpha)
GEO	Gene Expression Omnibus
GnRH	gonadotropin-releasing hormone
GO	Gene Ontology
HBA	Hydrogen bond acceptor
HBD	Hydrogen bond donor
hERG	human ether-à-go-go-related gene
HIF-1	Hypoxia-inducible factor 1
HSP90AA1	Heat shock protein 90 alpha family class A member 1
JNK	c-Jun N-terminal kinase
KEGG	Kyoto Encyclopedia of Genes and Genomes
log_2_FC	log_2_ fold-change
MCC	Maximal Clique Centrality
MCP-1	Monocyte chemoattractant protein-1
MF	Molecular function
MNC	Maximum Neighborhood Component
MS/MS	Tandem mass spectrometry
MW	Molecular weight
*m*/*z*	mass-to-charge ratio
NADPH	Reduced nicotinamide adenine dinucleotide phosphate
NF-κB	Nuclear factor kappa-light-chain-enhancer of activated B cells
NR	Nuclear receptor
NR3C1	Nuclear receptor subfamily 3 group C member 1
OMIM	Online Mendelian Inheritance in Man
PAINS	Pan-assay interference compounds
PCDL	Personal compound database and library
PDB	Protein Data Bank
PGR	Progesterone receptor
PI3K–Akt	Phosphoinositide 3-kinase–protein kinase B
PPARG	Peroxisome proliferator-activated receptor gamma
PPI	Protein–protein interaction
PTGS2	Prostaglandin-endoperoxide synthase 2 (cyclooxygenase-2)
QED	Quantitative estimate of drug-likeness
(U)HPLC–QTOF–MS	Ultra-high-performance liquid chromatography–quadrupole time-of-flight mass spectrometry
RNA-seq	RNA sequencing
ROS	Reactive oxygen species
RT	Retention time
SD	Standard deviation
SDF	Structure-data file
SMILES	Simplified Molecular-Input Line-Entry System
SRC	Proto-oncogene tyrosine-protein kinase Src
STAT3	Signal transducer and activator of transcription 3
STRING	Search Tool for the Retrieval of Interacting Genes/Proteins
TGF-βRI/RII	Transforming growth factor-β receptor I/II
TP53	Tumor protein p53
TPSA	Topological polar surface area
VCAM-1	Vascular cell adhesion molecule-1
