# Supplementary material for: Multi-Target Anti-Endometriosis Mechanisms of Camellia sinensis Polyphenols: An Integrated Computational and Transcriptomic Study
Source: Life (Basel). 2026 Sep 26;16(10):1613. doi: 10.3390/life16101613 (PMC13705937; doi:10.3390/life16101613)
Supplement: Supplementary file 1 [file life-16-01613-s001.zip › Table S4.pdf]

Table S4

Genes from DisGenet			Genes from Gene Cards					OMIM (Genes)
Gene	Gene Full N	score	Symbol	Name	Type	Relevance	Knowle	UQCRC2
ESR1	estrogen re	1.15	ESR1	Estrogen R	Protein Co	98.63384	100	PER2
CYP19A1	cytochrom	1.0	CYP19A1	Cytochrom	Protein Co	89.82405	100	PTGER3
PGR	progesterc	1.0	ESR2	Estrogen R	Protein Co	89.47487	100	MEIS2
IGF1	insulin like	1.0	TP53	Tumor Pro	Protein Co	84.76966	100	PIEZO1
ESR2	estrogen re	0.9	PTEN	Phosphata	Protein Co	81.85256	100	CHIT1
KRAS	KRAS protc	0.9	CDKN2A	Cyclin Dep	Protein Co	75.20026	100	FOSB
GREB1	growth reg	0.9	ARID1A	AT-Rich Int	Protein Co	72.12413	100	INTU
TNF	tumor nec	0.9	MMP2	Matrix Me	Protein Co	69.6808	100	LRP8
PRL	prolactin	0.85	KRAS	KRAS Protc	Protein Co	68.94509	100	SLC33A1
IL10	interleukin	0.85	CYP17A1	Cytochrom	Protein Co	67.93502	100	HK2
IL1B	interleukin	0.85	AR	Androgen	Protein Co	62.81653	100	EPHX1
PTGS2	prostaglan	0.8	TGFB1	Transformi	Protein Co	58.25068	100	IGFBP7
TGFB2	transformi	0.8	CYP1B1	Cytochrom	Protein Co	56.70023	100	AIMP2
EGFR	epidermal	0.8	BDNF	Brain Deriv	Protein Co	54.50999	100	CANX
PTGER2	prostaglan	0.75	MTOR	Mechanist	Protein Co	54.32129	100	DNAJB11
CCL11	C-C motif c	0.75	IL10	Interleukin	Protein Co	52.49207	100	GALE
IGFBP1	insulin like	0.75	VDR	Vitamin D	Protein Co	51.50295	100	HNRNPR
NR5A1	nuclear rec	0.75	PIK3CA	Phosphatic	Protein Co	51.09501	100	NARS1
ABI3BP	ABI family	0.7	BRCA2	BRCA2 DN	Protein Co	47.23581	100	RPL17
ITGB1	integrin su	0.7	ATM	ATM Serin	Protein Co	47.03674	100	RPS13
MIR21	microRNA	0.7	NFKB1	Nuclear Fa	Protein Co	44.57857	100	SEC31A
IL15	interleukin	0.7	AHR	Aryl Hydro	Protein Co	43.94213	100	U2AF2
IDO1	indoleamir	0.7	HGF	Hepatocyt	Protein Co	43.53347	100	UPF1
HSD17B1	hydroxyste	0.7	MME	Membrane	Protein Co	43.32149	100	PEPD
SERPINF1	serpin fam	0.7	ABCB1	ATP Bindin	Protein Co	43.21853	100	C3AR1
IL1R1	interleukin	0.65	FASLG	Fas Ligand	Protein Co	43.12502	100	DHX30
CNR1	cannabinoi	0.65	CTNNB1	Catenin Be	Protein Co	41.85921	100	LYZ
ERRFI1	ERBB recep	0.65	PLG	Plasminog	Protein Co	41.08489	100	CALCRL
KLF9	KLF transcr	0.65	HMOX1	Heme Oxyg	Protein Co	40.66486	100	COP1
NR4A1	nuclear rec	0.65	CXCR4	C-X-C Moti	Protein Co	40.57735	100	PCBP2
NCOA1	nuclear rec	0.65	BRCA1	BRCA1 DN	Protein Co	40.32147	100	RBFOX3
TNFRSF1B	TNF recept	0.65	FN1	Fibronectir	Protein Co	39.93019	100	TRPS1
PTPN22	protein tyr	0.65	STAT3	Signal Tran	Protein Co	39.37959	100	SOX4
HLA-DRB1	major histc	0.65	ALB	Albumin	Protein Co	39.05849	100	CD74
HSD17B2	hydroxyste	0.6	PPARG	Peroxisom	Protein Co	38.95742	100	NR0B2
AKR1C3	aldo-keto r	0.6	ERBB2	Erb-B2 Rec	Protein Co	38.59028	100	AGTR2
OLFM4	olfactomeo	0.6	FGFR1	Fibroblast	Protein Co	37.97618	100	SNRPN
CD226	CD226 mol	0.6	TERT	Telomeras	Protein Co	37.89852	100	PTHLH
BMP7	bone morp	0.6	NR3C1	Nuclear Re	Protein Co	37.42786	100	PPP2R1B
CXCL14	C-X-C moti	0.6	SMAD3	SMAD Fam	Protein Co	36.50623	100	GSTM1
DKK1	dickkopf W	0.6	JAK3	Janus Kina	Protein Co	36.00764	100	MMP7
HDAC2	histone de	0.6	NGF	Nerve Grov	Protein Co	33.32643	100	AHSG
CXCL9	C-X-C moti	0.6	AKT1	AKT Serine	Protein Co	33.23853	100	RUVBL2
DICER1	dicer 1, rib	0.6	DICER1	Dicer 1, Rib	Protein Co	31.97897	100	DNAH5
FOS	Fos proto-c	0.6	IGF1	Insulin Like	Protein Co	31.64343	100	ADIPOQ
NR2F2	nuclear rec	0.6	WT1	WT1 Trans	Protein Co	30.18322	100	TRAF1
PLA2G2A	phospholip	0.6	BRAF	B-Raf Protc	Protein Co	29.87773	100	MST1

ELAVL1	ELAV like R	0.6	NFE2L2	NFE2 Like I	Protein Co	29.43937	100	CTSH
HDAC1	histone de	0.6	FBN1	Fibrillin 1	Protein Co	29.10746	100	CYP1A2
HLA-DPB1	major histo	0.6	F7	Coagulatio	Protein Co	28.47612	100	IL18
TNC	tenascin C	0.6	MET	MET Proto	Protein Co	28.05415	100	LGALS3
NR3C2	nuclear rec	0.6	SLCO1B1	Solute Carr	Protein Co	27.96739	100	IL16
PTGER4	prostaglan	0.6	RORC	RAR Relate	Protein Co	27.05423	100	STRA6
AKR1C1	aldo-keto r	0.6	MMP9	Matrix Me	Protein Co	26.64196	100	CXCR3
MYLIP	myosin reg	0.6	VWF	Von Willeb	Protein Co	26.52782	100	ATP5F1B
IL7R	interleukin	0.6	ITGB3	Integrin Su	Protein Co	25.91097	100	PFKFB3
ARNT	aryl hydroc	0.6	AGT	Angiotensi	Protein Co	25.6005	100	SIRPA
LTF	lactotransf	0.6	RAD51	RAD51 Rec	Protein Co	24.02078	100	S100A9
PAX2	paired box	0.6	MLH1	MutL Hom	Protein Co	23.79132	100	BAK1
NRP1	neuropilin	0.6	CDK4	Cyclin Dep	Protein Co	23.574	100	ATAD3A
PRLR	prolactin r	0.55	MTHFR	Methylene	Protein Co	23.41839	100	SFRP4
KLF13	KLF transcr	0.55	IGF1R	Insulin Like	Protein Co	23.36233	100	HMGA1
PAPPA	pappalysin	0.55	NLRP3	NLR Family	Protein Co	23.28467	100	CADM1
MTA1	metastasis	0.55	POLE	DNA Polym	Protein Co	23.1314	100	S100A8
SRD5A1	steroid 5 a	0.5	MDM2	MDM2 Pro	Protein Co	23.10196	100	PSMD2
GPX3	glutathion	0.5	FAS	Fas Cell Su	Protein Co	23.09554	100	RAN
SST	somatosta	0.5	NRP1	Neuropilin	Protein Co	23.04397	100	SFPQ
MTA2	metastasis	0.5	C5	Compleme	Protein Co	22.96186	100	SOCS3
CD55	CD55 mole	0.5	HNRNPA2E	Heterogen	Protein Co	22.85959	100	KRT18
FBLN1	fibulin 1	0.5	IL6ST	Interleukin	Protein Co	22.78804	100	BAD
AKR1B1	aldo-keto r	0.5	RUNX1	RUNX Fam	Protein Co	22.28822	100	CTSG
IGFBP6	insulin like	0.5	MSH2	MutS Hom	Protein Co	21.91672	100	AKR1C4
NTRK3	neurotropl	0.5	APOE	Apolipopro	Protein Co	21.65494	100	DMBT1
ACTA2	actin alpha	0.5	PLCE1	Phospholip	Protein Co	20.82728	100	SBDS
ENPP1	ectonucleo	0.5	CFTR	CF Transm	Protein Co	20.76194	100	DOK1
TAGLN	transgelin	0.5	ERCC2	ERCC Excis	Protein Co	20.73622	100	EPO
NCOR1	nuclear rec	0.5	NCOR2	Nuclear Re	Protein Co	20.64163	100	TRIM24
VCAN	versican	0.5	GJA1	Gap Juncti	Protein Co	20.55601	100	CNGB3
CFD	compleme	0.5	ITGA6	Integrin Su	Protein Co	18.53834	100	TFDP1
HBEGF	heparin bir	0.5	POMC	Proopiome	Protein Co	18.53834	100	TK1
MED1	mediator c	0.5	MSH6	MutS Hom	Protein Co	17.53948	100	CSPG4
TRH	thyrotropi	0.5	GATA4	GATA Bind	Protein Co	17.21746	100	IL12A
TOB1	transducer	0.5	SMARCA4	SWI/SNF R	Protein Co	16.85069	100	MAP2
ABCC4	ATP bindin	0.5	C3	Compleme	Protein Co	15.88272	100	NTN1
RARB	retinoic aci	0.45	CDH1	Cadherin 1	Protein Co	15.69688	100	SELL
ITGB8	integrin su	0.45	COMT	Catechol-C	Protein Co	15.66089	100	ADD1
NR2C1	nuclear rec	0.45	RELA	RELA Proto	Protein Co	15.65307	100	AFDN
SULF2	sulfatase 2	0.4	EZH2	Enhancer (	Protein Co	15.63559	100	ASH2L
RASGRP1	RAS guany	0.4	EGFR	Epidermal	Protein Co	15.62084	100	CCT3
PLXNC1	plexin C1	0.4	SMAD2	SMAD Fam	Protein Co	15.58269	100	EEF1D
CPM	carboxype	0.4	NOTCH1	Notch Rece	Protein Co	15.51163	100	ENO2
DCSTAMP	dendrocyt	0.4	CDKN1B	Cyclin Dep	Protein Co	15.49643	100	FASTKD2
SLC16A6	solute carr	0.4	DNMT1	DNA Meth	Protein Co	15.49011	100	GOLGA2
COPS2	COP9 signa	0.4	EGF	Epidermal	Protein Co	15.40247	100	H1-4
HS3ST3B1	heparan su	0.4	NFKBIA	NFKB Inhib	Protein Co	15.37691	100	HDLBP
NR1D2	nuclear rec	0.4	HSPD1	Heat Shock	Protein Co	15.36903	100	NOP2
CYB5A	cytochrom	0.4	ACE	Angiotensi	Protein Co	15.329	100	PIP4K2A

DUSP1	dual specif	0.4	NOS3	Nitric Oxid	Protein Co	15.31207	100	PSMC4
FBN1	fibrillin 1	0.4	NCOA1	Nuclear Re	Protein Co	15.30781	100	RAB5C
ITGB3BP	integrin su	0.4	TNFRSF1A	TNF Recep	Protein Co	15.29396	100	RPS11
ANKRD1	ankyrin rep	0.4	SMAD4	SMAD Fam	Protein Co	15.28601	100	SSRP1
LAMB1	laminin sul	0.4	JAK2	Janus Kina:	Protein Co	15.28297	100	TAF10
CCL1	C-C motif c	0.4	CTLA4	Cytotoxic T	Protein Co	15.24874	100	TCP1
SELENOP	selenoprot	0.4	ACTA2	Actin Alpha	Protein Co	15.22603	100	TMEM43
SLC1A1	solute carr	0.4	DNMT3B	DNA Meth	Protein Co	15.19637	100	NDUFA13
MED16	mediator c	0.4	NTRK2	Neurotrop	Protein Co	15.18917	100	TBX6
CXCL13	C-X-C moti	0.4	STAT4	Signal Tran	Protein Co	15.17297	100	KCNAB2
ANO4	anoctamin	0.4	DNMT3A	DNA Meth	Protein Co	15.1288	100	LDHB
NCOA6	nuclear rec	0.4	CDH2	Cadherin 2	Protein Co	15.11575	100	RPS18
NEDD4L	NEDD4 like	0.4	IL12B	Interleukin	Protein Co	15.06244	100	SLC25A1
IHH	Indian hed	0.4	SOD1	Superoxide	Protein Co	15.06064	100	MUC5B
IMPA2	inositol mo	0.4	IL6R	Interleukin	Protein Co	15.05963	100	HTR2B
AREG	amphiregu	0.4	CSF1R	Colony Stir	Protein Co	15.05136	100	ADGRG6
TACSTD2	tumor asso	0.4	MMP14	Matrix Me	Protein Co	14.99747	100	CC2D2A
MAOA	monoamin	0.4	GATA2	GATA Bind	Protein Co	14.96148	100	DPP6
CCL22	C-C motif c	0.4	LDHA	Lactate De	Protein Co	14.94919	100	FKRP
THRA	thyroid ho	0.4	FGFR2	Fibroblast	Protein Co	14.92438	100	PDE5A
ARHGAP28	Rho GTPas	0.4	ADAM17	ADAM Met	Protein Co	14.86436	100	PTPN14
SPARCL1	SPARC like	0.4	ADA	Adenosine	Protein Co	14.85781	100	APOA4
SUCLG2	succinate-(	0.4	SERPINA1	Serpin Fam	Protein Co	14.85206	100	ATL1
NDNF	neuron der	0.4	CTSD	Cathepsin	Protein Co	14.83142	100	MMP8
STC2	stanniocalc	0.4	TGFB2	Transformi	Protein Co	14.75392	100	TP53BP2
MED14	mediator c	0.4	DRD2	Dopamine	Protein Co	14.75154	100	CCL5
MED17	mediator c	0.4	SHH	Sonic Hedg	Protein Co	14.74577	100	IL33
DDX5	DEAD-box	0.4	CTNNA1	Catenin Alp	Protein Co	14.7275	100	F3
FMO2	flavin cont	0.4	USP7	Ubiquitin S	Protein Co	14.72048	100	EGR1
RBPJ	recombina	0.4	KMT2A	Lysine Met	Protein Co	14.589	100	RUNX3
RASL11A	RAS like fa	0.4	JUP	Junction Pl	Protein Co	14.56539	100	NR5A2
LRRK2	leucine rich	0.4	IL2RB	Interleukin	Protein Co	14.54405	100	DDX39B
CYP26A1	cytochrom	0.4	GATA3	GATA Bind	Protein Co	14.54318	100	ANXA5
NR2F6	nuclear rec	0.4	AXL	AXL Recep	Protein Co	14.53703	100	FKBP4
LMOD1	leiomodin	0.4	ZEB2	Zinc Finger	Protein Co	14.43824	100	CXCL10
TMT1A	thiol meth	0.4	SLC2A1	Solute Carr	Protein Co	14.38662	100	INHBA
FAM180A	family with	0.4	STAT1	Signal Tran	Protein Co	14.37731	100	HMGA2
MAOB	monoamin	0.4	AGTR1	Angiotensi	Protein Co	14.3732	100	PHB1
NEFM	neurofilam	0.4	FGA	Fibrinogen	Protein Co	14.36836	100	DDIT3
UST	uronyl 2-su	0.4	TGFBR1	Transformi	Protein Co	14.36836	100	JUNB
TXNIP	thioredoxi	0.4	FBXW7	F-Box And	Protein Co	14.33575	100	LY96
BRD8	bromodom	0.4	APOA1	Apolipopro	Protein Co	14.32113	100	SEMA3F
DEPP1	DEPP auto	0.4	CTSB	Cathepsin	Protein Co	14.30099	100	EIF3B
ANKH	ANKH inor	0.4	PTPN11	Protein Tyr	Protein Co	14.20317	100	RPL7
RXFP1	relaxin fam	0.4	HRAS	HRas Proto	Protein Co	14.20047	100	RPL7A
RGS4	regulator c	0.4	MTR	5-Methylte	Protein Co	14.17913	100	RPS16
RORB	RAR relate	0.4	RAF1	Raf-1 Proto	Protein Co	14.17913	100	SLC25A5
SLC20A1	solute carr	0.4	VCAN	Versican	Protein Co	14.17913	100	SDC1
SRD5A2	steroid 5 a	0.4	ATP2A2	ATPase Sar	Protein Co	14.17211	100	IL13
C1R	compleme	0.4	BMP4	Bone Morp	Protein Co	14.17211	100	SSTR5

CLDN1	claudin 1	0.4	CASP8	Caspase 8	Protein Co	14.17211	100	IL18R1
CCNE2	cyclin E2	0.4	MYLK	Myosin Lig	Protein Co	14.13768	100	EIF3A
CNIH3	cornichon	0.4	KMT2D	Lysine Met	Protein Co	14.08802	100	ERG
AKR1C2	aldo-keto r	0.4	BRIP1	BRCA1 Inte	Protein Co	13.73497	100	GABRA3
DIO2	iodothyror	0.4	PDGFRB	Platelet De	Protein Co	13.73189	100	GABRA6
FKBP5	FKBP proly	0.4	SMO	Smoothen	Protein Co	13.72425	100	MYOD1
SLC7A8	solute carr	0.4	ACTB	Actin Beta	Protein Co	13.70661	100	NEIL1
MED4	mediator c	0.4	ATP1A1	ATPase Na	Protein Co	13.70661	100	KPNB1
NR3C1	nuclear rec	0.4	CLTC	Clathrin He	Protein Co	13.70661	100	IL7
SLC40A1	solute carr	0.4	DSP	Desmoplak	Protein Co	13.70661	100	CAPN5
IFNGR1	interferon	0.4	EEF2	Eukaryotic	Protein Co	13.70661	100	GSTA1
ITGA2	integrin su	0.4	FUS	FUS RNA B	Protein Co	13.70661	100	HSD11B1
ABCC9	ATP bindin	0.4	RANBP2	RAN Bindir	Protein Co	13.70661	100	MSTN
SMPDL3A	sphingomy	0.4	RPSA	Ribosomal	Protein Co	13.70661	100	EML4
OSR2	odd-skippe	0.4	TUBB	Tubulin Be	Protein Co	13.70661	100	ACSL3
PTGFR	prostaglan	0.4	TYK2	Tyrosine Ki	Protein Co	13.70661	100	ARF4
NR2C2	nuclear rec	0.4	VCP	Valosin Co	Protein Co	13.70661	100	ECM1
SCGB3A1	secretoglo	0.4	NOTCH3	Notch Rece	Protein Co	13.65203	100	EIF5A
FAM163A	family with	0.4	PIK3R1	Phosphoin	Protein Co	13.65203	100	ESRRG
			CD40	CD40 Mole	Protein Co	13.48653	100	FXR1
			EIF2AK3	Eukaryotic	Protein Co	13.45849	100	HNRNPL
			EPHA2	EPH Recep	Protein Co	13.45542	100	HNRNPM
			FLT4	Fms Relate	Protein Co	13.45542	100	HSPA1B
			TLR3	Toll Like Re	Protein Co	13.44293	100	KRT2
			NTRK1	Neurotrop	Protein Co	13.39679	100	LMO7
			ADAM10	ADAM Met	Protein Co	13.27504	100	MYO18A
			KIT	KIT Proto-C	Protein Co	13.27197	100	PARP14
			IL2RA	Interleukin	Protein Co	13.26843	100	PSMC2
			TCF3	Transcripti	Protein Co	13.26432	100	PSMC6
			TCF7L2	Transcripti	Protein Co	13.26432	100	RAB1A
			TP63	Tumor Pro	Protein Co	13.26432	100	RING1
			CD55	CD55 Mole	Protein Co	13.24668	100	RPL23A
			COL1A1	Collagen T	Protein Co	13.23797	100	RPS23
			GLB1	Galactosid	Protein Co	13.22687	100	RPS5
			IDH1	Isocitrate D	Protein Co	13.0266	100	SEC13
			ABCB11	ATP Bindin	Protein Co	12.85376	100	SF3B2
			IFNAR2	Interferon	Protein Co	12.85376	100	SLC27A2
			RB1	RB Transcr	Protein Co	12.79881	100	SRP72
			ARG1	Arginase 1	Protein Co	12.79398	100	LITAF
			HNF4A	Hepatocyt	Protein Co	12.79398	100	PPP2CB
			EPHB4	EPH Recep	Protein Co	12.7882	100	SRSF2
			TEK	TEK Recept	Protein Co	12.7882	100	UBA52
			CHUK	Componen	Protein Co	12.78118	100	HGD
			ABL1	ABL Proto-	Protein Co	12.77246	100	PER1
			NOD2	Nucleotide	Protein Co	12.77246	100	AGAP1
			NOTCH2	Notch Rece	Protein Co	12.77246	100	COL13A1
			TH	Tyrosine H	Protein Co	12.77246	100	DDHD2
			BTK	Bruton Tyr	Protein Co	12.76136	100	EIF4B
			CD36	CD36 Mole	Protein Co	12.76136	100	GPNMB
			CREBBP	CREB Bindi	Protein Co	12.76136	100	GTF2H1

KCNQ2	Potassium	Protein Co	12.76136	100	GPC6
CACNA1D	Calcium Vc	Protein Co	12.74674	100	CSF2
CFB	Compleme	Protein Co	12.74674	100	LNPEP
PDGFB	Platelet De	Protein Co	12.74674	100	GPER1
RAD50	RAD50 Dou	Protein Co	12.74674	100	NLRC5
TAF1	TATA-Box I	Protein Co	12.74674	100	CDKN2B
CHEK2	Checkpoin	Protein Co	12.7266	100	NBAS
LAMB1	Laminin Su	Protein Co	12.7266	100	METAP2
NQO1	NAD(P)H C	Protein Co	12.7266	100	FABP4
TNC	Tenascin C	Protein Co	12.7266	100	MKI67
TRPC6	Transient F	Protein Co	12.7266	100	FZD7
VHL	Von Hippel	Protein Co	12.7266	100	MAP1LC3B
ACTN1	Actinin Alp	Protein Co	12.69709	100	TNFSF13B
AKT3	AKT Serine	Protein Co	12.69709	100	SLC22A6
F2	Coagulatio	Protein Co	12.69709	100	KLF11
JAG1	Jagged Car	Protein Co	12.69709	100	NAA15
LYN	LYN Proto-	Protein Co	12.69709	100	KLK2
VAV1	Vav Guanir	Protein Co	12.69709	100	MBD2
SLC1A2	Solute Carı	Protein Co	12.64969	100	ILF3
SLC6A4	Solute Carı	Protein Co	12.64969	100	RPL10A
TF	Transferrir	Protein Co	12.64969	100	RPL12
ALDH3A2	Aldehyde D	Protein Co	12.56109	100	RPL4
EP300	EP300 Lysi	Protein Co	12.56109	100	RPS4X
ACAT1	Acetyl-CoA	Protein Co	12.3362	100	RPS8
ACTG1	Actin Gamı	Protein Co	12.3362	100	U2AF1
AKT2	AKT Serine	Protein Co	12.3362	100	SORBS1
CHD4	Chromodo	Protein Co	12.3362	100	NECTIN4
CSNK2A1	Casein Kina	Protein Co	12.3362	100	CRHR2
CTNND1	Catenin De	Protein Co	12.3362	100	SLIT3
DIAPH1	Diaphanou	Protein Co	12.3362	100	CDA
DNM2	Dynamin 2	Protein Co	12.3362	100	CPT1B
DYNC1H1	Dynein Cyt	Protein Co	12.3362	100	GABRA4
EIF4G1	Eukaryotic	Protein Co	12.3362	100	C4BPA
FASN	Fatty Acid :	Protein Co	12.3362	100	YWHAQ
FLNA	Filamin A	Protein Co	12.3362	100	PIAS3
GPI	Glucose-6-	Protein Co	12.3362	100	RBP1
HNRNPK	Heterogen	Protein Co	12.3362	100	NUP210
HSD17B4	Hydroxyste	Protein Co	12.3362	100	CCR6
ITPR1	Inositol 1,4	Protein Co	12.3362	100	BCAT1
ITPR3	Inositol 1,4	Protein Co	12.3362	100	CEL
KMT2C	Lysine Met	Protein Co	12.3362	100	SDC4
MAP2K2	Mitogen-A	Protein Co	12.3362	100	ACVR2A
P4HB	Prolyl 4-Hy	Protein Co	12.3362	100	FAAH
PHGDH	Phosphogl	Protein Co	12.3362	100	GAB2
PPP1CB	Protein Ph	Protein Co	12.3362	100	CHL1
PRKDC	Protein Kir	Protein Co	12.3362	100	DDX21
PRKN	Parkin RBR	Protein Co	12.3362	100	EIF2S1
PTCH1	Patched 1	Protein Co	12.3362	100	FLII
RPL5	Ribosomal	Protein Co	12.3362	100	GCLM
RPS19	Ribosomal	Protein Co	12.3362	100	GFM2

SDHA	Succinate I	Protein Co	12.3362	100	MAN2A1
SPTBN1	Spectrin Be	Protein Co	12.3362	100	MAPRE1
TJP2	Tight Junct	Protein Co	12.3362	100	MSR1
TUBB3	Tubulin Be	Protein Co	12.3362	100	MYBBP1A
TUBB4A	Tubulin Be	Protein Co	12.3362	100	PSMD11
CSF2RB	Colony Stir	Protein Co	10.61539	100	RPL24
DCTN1	Dynactin S	Protein Co	10.61539	100	HSPA4
LIG1	DNA Ligase	Protein Co	10.61539	100	PAK5
TNFAIP3	TNF Alpha	Protein Co	10.61279	100	RPS3A
PTPRC	Protein Tyı	Protein Co	10.02884	100	NTF3
ACTA1	Actin Alpha	Protein Co	9.212296	100	ANAPC1
ALDOA	Aldolase, F	Protein Co	9.212296	100	ASTN2
MTHFD1	Methylene	Protein Co	9.212296	100	CBX5
PIK3C2A	Phosphatic	Protein Co	9.212296	100	NRG3
RET	Ret Proto-(	Protein Co	9.212296	100	PSMD13
PAX6	Paired Box	Protein Co	9.201195	100	RAB4A
G6PD	Glucose-6-	Protein Co	9.136921	100	SYNJ2
RUNX2	RUNX Fam	Protein Co	9.000925	100	STAG3
ITCH	Itchy E3 Ub	Protein Co	8.77603	100	SDCBP
LAMA5	Laminin Su	Protein Co	8.77603	100	AVPR1A
SMARCB1	SWI/SNF R	Protein Co	8.18842	100	ADAM12
ATIC	5-Aminoim	Protein Co	8.080542	100	SMC5
AXIN2	Axin 2	Protein Co	8.080542	100	KCNK9
CACNA1A	Calcium Vc	Protein Co	8.080542	100	LTC4S
DAG1	Dystroglyc	Protein Co	8.080542	100	NPAS2
ERBB4	Erb-B2 Rec	Protein Co	8.080542	100	TIMP1
HSPG2	Heparan Sı	Protein Co	8.080542	100	IL17A
MAP3K1	Mitogen-A	Protein Co	8.080542	100	GC
PLCB1	Phospholip	Protein Co	8.080542	100	CPB2
POLR2A	RNA Polym	Protein Co	8.080542	100	CNR2
ZAP70	Zeta Chain	Protein Co	8.080542	100	CXCL1
ERCC1	ERCC Excis	Protein Co	7.98948	100	SF1
INSR	Insulin Rec	Protein Co	7.98948	100	CGA
SMARCA2	SWI/SNF R	Protein Co	7.832011	100	LILRB1
COL1A2	Collagen T	Protein Co	6.139478	100	HOXA13
MAP3K7	Mitogen-A	Protein Co	6.062575	100	CX3CL1
PRKCD	Protein Kir	Protein Co	5.708739	100	LCN2
ITGAM	Integrin Su	Protein Co	5.694116	100	ANG
ITGB2	Integrin Su	Protein Co	5.694116	100	IGF2BP1
EPAS1	Endothelia	Protein Co	5.673974	100	NODAL
FGFR3	Fibroblast	Protein Co	5.508469	100	IGF2BP2
KCNH2	Potassium	Protein Co	5.508469	100	PHB2
KDM1A	Lysine Den	Protein Co	5.508469	100	EIF3E
PCSK9	Proprotein	Protein Co	5.508469	100	MT-ND5
XIAP	X-Linked Ir	Protein Co	5.508469	100	USP10
GNB1	G Protein S	Protein Co	5.283573	100	TNIP2
ACTN4	Actinin Alp	Protein Co	3.560168	100	SLC25A3
IKBKB	Inhibitor O	Protein Co	2.084297	100	UGCG
NR1H4	Nuclear Re	Protein Co	2.084297	100	MGMT
OPRM1	Opioid Rec	Protein Co	2.036903	100	LEFTY2

F10	Coagulatio	Protein Co	1.948301	100	SPINK1
F12	Coagulatio	Protein Co	1.948301	100	RPLP1
F9	Coagulatio	Protein Co	1.948301	100	SLIT1
MCM2	Minichrom	Protein Co	1.948301	100	GABRG3
ALK	ALK Recep	Protein Co	1.723406	100	PTGDR
ATR	ATR Check	Protein Co	1.723406	100	SLCO1A2
ITGA2	Integrin Su	Protein Co	1.723406	100	BMP6
KCNQ1	Potassium	Protein Co	1.723406	100	CCR7
MEN1	Menin 1	Protein Co	1.723406	100	P2RX1
PRKCG	Protein Kir	Protein Co	1.723406	100	FGF7
RASA1	RAS P21 Pr	Protein Co	1.723406	100	ITIH4
SQSTM1	Sequestos	Protein Co	1.723406	100	MFGE8
KDR	Kinase Inse	Protein Co	76.16293	99	BDKRB1
PLAU	Plasminog	Protein Co	69.40487	99	HMOX2
SERPINE1	Serpin Fam	Protein Co	58.55598	99	TRPC1
CDC42	Cell Divisio	Protein Co	46.67595	99	DNASE1
TYMP	Thymidine	Protein Co	39.00436	99	NPY
F8	Coagulatio	Protein Co	28.47612	99	NR4A3
LEPR	Leptin Rec	Protein Co	23.33435	99	USF1
HNRNPA1	Heterogen	Protein Co	21.78715	99	ADAMTS5
C2	Compleme	Protein Co	20.86874	99	WWTR1
RARB	Retinoic Ac	Protein Co	18.25804	99	COPG1
CYP2D6	Cytochrom	Protein Co	17.31343	99	DDRGK1
PMS2	PMS1 Hom	Protein Co	16.29507	99	G3BP1
ZEB1	Zinc Finger	Protein Co	15.60888	99	GTPBP1
MUC1	Mucin 1, C	Protein Co	15.52808	99	NACA
CEBPA	CCAAT Enh	Protein Co	14.7275	99	PRPF19
STING1	Stimulator	Protein Co	14.36259	99	RAB8A
STAT6	Signal Tran	Protein Co	14.17211	99	RFC3
HNRNPU	Heterogen	Protein Co	13.70661	99	RPL14
ILK	Integrin Lir	Protein Co	13.62686	99	RPL22
PAK1	P21 (RAC1)	Protein Co	13.37555	99	SQLE
CFH	Compleme	Protein Co	13.2537	99	WNT9B
UGT1A1	UDP Glucu	Protein Co	13.1921	99	TRIB3
GABRG2	Gamma-Ar	Protein Co	12.85376	99	DDX1
CASR	Calcium Se	Protein Co	12.77246	99	SCO1
TGFB3	Transformi	Protein Co	12.77246	99	SLC25A10
CETP	Cholestery	Protein Co	12.74674	99	TRA2B
NEK2	NIMA Rela	Protein Co	12.74674	99	APLNR
COL18A1	Collagen T	Protein Co	12.7266	99	HES1
GLI3	GLI Family	Protein Co	12.7266	99	PPP1R15A
PLCB4	Phospholip	Protein Co	12.7266	99	GRIK3
FPR1	Formyl Pep	Protein Co	12.69709	99	NME7
VCL	Vinculin	Protein Co	12.69709	99	SLC24A4
ATP6V1A	ATPase H+	Protein Co	12.64969	99	TTK
IDH2	Isocitrate D	Protein Co	12.3362	99	LPAR2
LBR	Lamin B Re	Protein Co	12.3362	99	MTPAP
SPTAN1	Spectrin Al	Protein Co	12.3362	99	AP2A2
TUBA4A	Tubulin Alp	Protein Co	12.3362	99	CD163
F5	Coagulatio	Protein Co	10.02884	99	WASL

NTRK3	Neurotrop	Protein Co	9.712886	99	FADS1
PSMB8	Proteasom	Protein Co	9.16643	99	HOXA10
BLK	BLK Proto-	Protein Co	8.080542	99	GNRH1
BMPR2	Bone Morp	Protein Co	8.080542	99	CCN1
GRIK2	Glutamate	Protein Co	8.080542	99	GSTM2
NOTCH4	Notch Rece	Protein Co	8.080542	99	ACKR3
TOP2B	DNA Topoi	Protein Co	8.080542	99	MCAM
ATRX	ATRX Chro	Protein Co	7.98948	99	MICA
NOS1	Nitric Oxid	Protein Co	7.98948	99	CBX3
CARD11	Caspase Re	Protein Co	6.927439	99	HAMP
EGLN1	Egl-9 Famil	Protein Co	3.560168	99	CCNA1
TNFRSF11A	TNF Recep	Protein Co	3.560168	99	KRT19
MYD88	MYD88 Inr	Protein Co	1.723406	99	NAT2
NR5A1	Nuclear Re	Protein Co	71.8718	98	NCR1
FSHR	Follicle Stir	Protein Co	43.98532	98	PTGIS
ENG	Endoglin	Protein Co	38.47647	98	MAP1LC3A
LHCGR	Luteinizing	Protein Co	36.47297	98	H2AX
F11	Coagulatio	Protein Co	28.47612	98	HPSE
GSR	Glutathion	Protein Co	27.03867	98	GHRL
ESRRB	Estrogen R	Protein Co	26.94178	98	CBR1
ARID1B	AT-Rich Int	Protein Co	25.51184	98	PDLIM3
IGF2	Insulin Like	Protein Co	15.05463	98	FABP5
LMNB1	Lamin B1	Protein Co	14.85936	98	ABCF1
VIM	Vimentin	Protein Co	14.85609	98	SRSF7
CXCR2	C-X-C Moti	Protein Co	14.75605	98	VDAC2
SOX9	SRY-Box Tr	Protein Co	14.39772	98	SRF
ELANE	Elastase, N	Protein Co	14.38392	98	F2RL1
MAT2A	Methionin	Protein Co	14.20317	98	SSTR2
HDAC6	Histone De	Protein Co	14.20047	98	NAT10
CYP11A1	Cytochrom	Protein Co	14.18975	98	STOM
LIMK1	LIM Domai	Protein Co	14.18491	98	SYNCRIP
ATP5F1A	ATP Syntha	Protein Co	13.70661	98	TSHB
EPRS1	Glutamyl-F	Protein Co	13.70661	98	MUC2
ITGA7	Integrin Su	Protein Co	13.6979	98	SMAD5
GABRA1	Gamma-Ar	Protein Co	12.85376	98	HYAL2
GABRB2	Gamma-Ar	Protein Co	12.85376	98	GRHL2
GABRB3	Gamma-Ar	Protein Co	12.85376	98	KLK4
ADAR	Adenosine	Protein Co	12.76136	98	SIRT7
SLIT2	Slit Guidan	Protein Co	12.76136	98	TFEB
CD40LG	CD40 Ligar	Protein Co	12.74674	98	CCR9
PRNP	Prion Prote	Protein Co	12.74674	98	CXADR
SERPING1	Serpin Fam	Protein Co	12.74674	98	IL3RA
SMPD1	Sphingomy	Protein Co	12.74674	98	KLF10
SPARC	Secreted P	Protein Co	12.74674	98	SDC2
PCK1	Phosphoer	Protein Co	12.7266	98	CCT7
PGK1	Phosphogl	Protein Co	12.7266	98	COPB1
PIK3CG	Phosphatic	Protein Co	12.7266	98	KHSRP
PPP1R12A	Protein Ph	Protein Co	12.7266	98	KRT9
CHEK1	Checkpoin	Protein Co	12.69709	98	MACROH2A1
MTRR	5-Methylte	Protein Co	12.64969	98	MAP4

SETD2	SET Domai	Protein Co	12.64969	98	NEDD8
IL12RB1	Interleukin	Protein Co	12.56109	98	RAB14
ABCC1	ATP Bindin	Protein Co	12.3362	98	RCC1
ADSL	Adenylosu	Protein Co	12.3362	98	RPL18A
ALDH7A1	Aldehyde D	Protein Co	12.3362	98	RPL27A
COPA	Coat Prote	Protein Co	12.3362	98	RPLP2
GFPT1	Glutamine-	Protein Co	12.3362	98	RPN1
HADHB	Hydroxyac	Protein Co	12.3362	98	SARS1
MYH14	Myosin He	Protein Co	12.3362	98	SNRPE
MYO1C	Myosin IC	Protein Co	12.3362	98	VDAC3
PPIB	Peptidylpr	Protein Co	12.3362	98	ZNFX1
RDX	Radixin	Protein Co	12.3362	98	CUX2
NDUFS2	NADH:Ubi	Protein Co	9.212296	98	NME4
UBE3A	Ubiquitin P	Protein Co	9.212296	98	RALGAPA1
BMP2	Bone Morp	Protein Co	9.089527	98	SF3B3
TOP3A	DNA Topoi	Protein Co	8.77603	98	SRSF6
TTN	Titin	Protein Co	8.77603	98	TARS1
GRM7	Glutamate	Protein Co	8.080542	98	PYCARD
IRF4	Interferon	Protein Co	8.080542	98	APOD
MAP3K8	Mitogen-A	Protein Co	5.673974	98	MPZ
HMGCR	3-Hydroxy-	Protein Co	5.644465	98	ADAMTS18
EPHA4	EPH Recep	Protein Co	5.597071	98	EXOC2
CYP11B1	Cytochrom	Protein Co	5.283573	98	HOXA2
HNF1B	HNF1 Hom	Protein Co	83.34821	97	MAP3K12
FLT1	Fms Relate	Protein Co	44.04592	97	QRICH1
VEGFA	Vascular E	Protein Co	42.02627	97	TARBP2
PPP2R1A	Protein Ph	Protein Co	41.74331	97	CATSPER1
TWIST1	Twist Fami	Protein Co	39.49542	97	DNAH1
IL1RN	Interleukin	Protein Co	37.98754	97	ODF2
NDRG1	N-Myc Dov	Protein Co	31.81146	97	RXFP2
ADAMTS13	ADAM Met	Protein Co	26.52782	97	TRADD
TARDBP	TAR DNA B	Protein Co	25.18996	97	PTGER1
GPX4	Glutathion	Protein Co	22.17923	97	TDO2
BMPR1B	Bone Morp	Protein Co	22.10701	97	CCR4
EFTUD2	Elongation	Protein Co	17.29284	97	NBR1
SIRT1	Sirtuin 1	Protein Co	15.73043	97	HEY1
CD44	CD44 Mole	Protein Co	15.70279	97	LSR
MMP13	Matrix Me	Protein Co	15.32976	97	MUC5AC
STUB1	STIP1 Hom	Protein Co	14.78242	97	PER3
TPM3	Tropomyo	Protein Co	14.76723	97	REV1
NOS2	Nitric Oxid	Protein Co	14.7275	97	STX5
SLCO1B3	Solute Car	Protein Co	14.57716	97	LIF
SRC	SRC Proto-	Protein Co	14.49586	97	CRH
STK4	Serine/Thr	Protein Co	14.17913	97	THOC6
GPC3	Glypican 3	Protein Co	14.1634	97	NPSR1
MAOA	Monoamir	Protein Co	14.11921	97	FSHB
ATF6	Activating	Protein Co	13.72425	97	SRY
HSPA9	Heat Shock	Protein Co	13.70661	97	SNRPD2
NPM1	Nucleopho	Protein Co	13.70661	97	AKR1A1
ANGPT1	Angiopoiet	Protein Co	13.25948	97	ABO

CPT2	Carnitine P	Protein Co	12.85376	97	PDLIM5
GABRA2	Gamma-Ar	Protein Co	12.85376	97	HSD3B1
IL12RB2	Interleukin	Protein Co	12.78118	97	MDK
SLC12A2	Solute Carr	Protein Co	12.77246	97	LGALS1
NT5E	5'-Nucleoti	Protein Co	12.76136	97	SLC22A8
RAC1	Rac Family	Protein Co	12.76136	97	STMN1
VLDLR	Very Low D	Protein Co	12.76136	97	F11R
LAMC1	Laminin Su	Protein Co	12.7266	97	PF4
DNA2	DNA Replic	Protein Co	12.69709	97	TNFRSF6B
BAP1	BRCA1 Ass	Protein Co	12.56109	97	LGR5
AARS1	Alanyl-TRN	Protein Co	12.3362	97	CITED2
ACO2	Aconitase 2	Protein Co	12.3362	97	TNS1
C1R	Compleme	Protein Co	12.3362	97	BNC2
DKC1	Dyskerin P	Protein Co	12.3362	97	DHX15
FLNB	Filamin B	Protein Co	12.3362	97	SLC25A11
GARS1	Glycyl-TRN	Protein Co	12.3362	97	HAND2
NDUFS1	NADH:Ubic	Protein Co	12.3362	97	LILRB2
PLEC	Plectin	Protein Co	12.3362	97	CCL20
KDM6A	Lysine Den	Protein Co	10.61539	97	SSTR1
NRAS	NRAS Proto	Protein Co	9.221008	97	FIP1L1
AHCY	Adenosylh	Protein Co	9.212296	97	CBX1
FECH	Ferrochela	Protein Co	9.212296	97	ICAM3
GRIN2D	Glutamate	Protein Co	8.080542	97	PPM1A
ROBO2	Roundabou	Protein Co	8.080542	97	IL27RA
POLG	DNA Polym	Protein Co	7.98948	97	ALOX15B
SMARCC1	SWI/SNF R	Protein Co	7.832011	97	PLAGL1
MSN	Moesin	Protein Co	3.560168	97	SFN
COL4A1	Collagen Ty	Protein Co	2.133948	97	THY1
CCND2	Cyclin D2	Protein Co	1.948301	97	TONSL
KAT2B	Lysine Ace	Protein Co	1.948301	97	GPBAR1
CD46	CD46 Mole	Protein Co	1.723406	97	MBD1
TLR4	Toll Like Re	Protein Co	42.88711	96	SYP
HIF1A	Hypoxia In	Protein Co	42.13037	96	HMCN1
OXTR	Oxytocin R	Protein Co	32.05559	96	ENPEP
PAX8	Paired Box	Protein Co	29.23402	96	NACC1
HSD3B2	Hydroxy-D	Protein Co	28.93792	96	SPRY2
GSK3B	Glycogen S	Protein Co	21.42579	96	ABCE1
PTK2	Protein Tyr	Protein Co	15.31717	96	ACIN1
PARP1	Poly(ADP-F	Protein Co	15.25041	96	ACSL5
TLR2	Toll Like Re	Protein Co	15.12175	96	BGLAP
HLA-B	Major Hist	Protein Co	15.05734	96	CCT4
DCN	Decorin	Protein Co	14.36259	96	CNBP
PARK7	Parkinsonis	Protein Co	14.18975	96	CPSF6
PRPF8	Pre-MRNA	Protein Co	14.17913	96	DEK
ENPP1	Ectonuclec	Protein Co	14.1634	96	EIF3I
DDX3X	DEAD-Box	Protein Co	13.70661	96	ELP3
DHCR7	7-Dehydro	Protein Co	13.70661	96	NMT1
FTL	Ferritin Lig	Protein Co	13.6979	96	PDLIM7
AIFM1	Apoptosis	Protein Co	13.65203	96	RBBP5
DDB2	Damage Sp	Protein Co	13.62252	96	RPS12

AXIN1	Axin 1	Protein Co	13.26432	96	SEPTIN2
LAMC2	Laminin Su	Protein Co	13.24668	96	SRRT
NRP2	Neuropilin	Protein Co	13.24668	96	TKFC
CP	Ceruloplas	Protein Co	12.85376	96	TMED7
L1CAM	L1 Cell Adh	Protein Co	12.80293	96	VAPA
SCN9A	Sodium Vo	Protein Co	12.79398	96	BAG5
TERF2IP	TERF2 Inte	Protein Co	12.78118	96	CACNA2D3
DNM1L	Dynamin 1	Protein Co	12.76136	96	GTF2I
EPOR	Erythropoi	Protein Co	12.74674	96	PRPF40A
FDFT1	Farnesyl-D	Protein Co	12.64969	96	CHRDL1
FGFR4	Fibroblast	Protein Co	12.64969	96	CYP2C18
TJP1	Tight Junct	Protein Co	12.64969	96	ATG12
GRN	Granulin P	Protein Co	12.56109	96	DMXL2
IHH	Indian Hed	Protein Co	12.56109	96	CAMK1D
ASPH	Aspartate	Protein Co	12.3362	96	EIF3H
CDC6	Cell Divisio	Protein Co	12.3362	96	FAF1
FHL2	Four And A	Protein Co	12.3362	96	LRP1B
LAMB3	Laminin Su	Protein Co	12.3362	96	SNX9
LONP1	Lon Peptid	Protein Co	12.3362	96	TFPI
NDUFV1	NADH:Ubi	Protein Co	12.3362	96	VPS13B
NR4A2	Nuclear Re	Protein Co	12.3362	96	MSH5
PDHB	Pyruvate D	Protein Co	12.3362	96	PDCD4
RPL15	Ribosomal	Protein Co	12.3362	96	AFP
SLC25A13	Solute Car	Protein Co	12.3362	96	ARX
SLC25A4	Solute Car	Protein Co	12.3362	96	ADAM8
STT3A	STT3 Oligo	Protein Co	12.3362	96	CHD6
FOXP2	Forkhead B	Protein Co	8.080542	96	PMEL
PIKFYVE	Phosphoin	Protein Co	8.080542	96	AKT1S1
TYR	Tyrosinase	Protein Co	8.080542	96	MEGF10
TCF12	Transcripti	Protein Co	5.694116	96	TCF7L1
PGR	Progesterc	Protein Co	97.36697	95	CA1
LEP	Leptin	Protein Co	47.77759	95	FADS2
PAX2	Paired Box	Protein Co	44.00511	95	PDK2
NR2F2	Nuclear Re	Protein Co	43.62262	95	WNT7B
CTCF	CCCTC-Bin	Protein Co	36.83056	95	EMX2
UCHL1	Ubiquitin C	Protein Co	26.53599	95	SAA1
SOX2	SRY-Box Tr	Protein Co	21.9788	95	HOXA11
SH2B3	SH2B Adap	Protein Co	17.40387	95	LGALS9
NRIP1	Nuclear Re	Protein Co	17.15591	95	SNAI1
G6PC1	Glucose-6-	Protein Co	16.39101	95	LHB
RAD51B	RAD51 Par	Protein Co	16.0313	95	MICB
ICAM1	Intercellula	Protein Co	15.73885	95	MAPK8IP1
THBS1	Thrombos	Protein Co	15.73161	95	TIMP2
ITGAV	Integrin Su	Protein Co	15.57951	95	CHI3L1
MYC	MYC Proto	Protein Co	15.52129	95	CCL21
HLA-DRB1	Major Hist	Protein Co	15.51243	95	ACP1
XRCC1	X-Ray Repa	Protein Co	15.37514	95	ID1
GATA6	GATA Bind	Protein Co	15.28688	95	ADM
HLA-A	Major Hist	Protein Co	15.05308	95	MRC1
GLI1	GLI Family	Protein Co	15.03918	95	CEBPD

MTAP	Methylthic	Protein Co	15.00798	95	SERPINA6
WNT5A	Wnt Family	Protein Co	14.85206	95	CSMD1
XRCC4	X-Ray Repa	Protein Co	14.74884	95	LARP1
CYP27A1	Cytochrom	Protein Co	14.19386	95	FSCN1
ROCK2	Rho Associ	Protein Co	14.18975	95	CYP3A7
CA2	Carbonic A	Protein Co	14.1523	95	COPS5
FZD4	Frizzled Cla	Protein Co	14.04063	95	PMS1
BUB3	BUB3 Mito	Protein Co	13.70661	95	PRMT2
SNRNP200	Small Nucl	Protein Co	13.70661	95	PGF
LIFR	LIF Recept	Protein Co	13.26432	95	MMP12
GABRB1	Gamma-Ar	Protein Co	12.85376	95	CXXC1
DES	Desmin	Protein Co	12.77246	95	CCR8
KIF22	Kinesin Far	Protein Co	12.77246	95	DCLK1
HTRA2	HtrA Serin	Protein Co	12.76136	95	AHCTF1
RBP4	Retinol Bin	Protein Co	12.76136	95	ATP5PO
ERCC5	ERCC Excis	Protein Co	12.74674	95	BAG6
LIPC	Lipase C, H	Protein Co	12.74674	95	CCT8
MAF	MAF BZIP T	Protein Co	12.74674	95	CNP
B2M	Beta-2-Mic	Protein Co	12.7266	95	DDX17
CDH3	Cadherin 3	Protein Co	12.7266	95	EIF2S2
EDNRA	Endothelin	Protein Co	12.7266	95	FARSA
HPGD	15-Hydrox	Protein Co	12.7266	95	G3BP2
LAMA1	Laminin Su	Protein Co	12.7266	95	GTF2F1
CFI	Compleme	Protein Co	12.69709	95	KHDRBS1
NEDD4	NEDD4 E3	Protein Co	12.64969	95	NPEPPS
PRDM16	PR/SET Do	Protein Co	12.64969	95	NXF1
AGO2	Argonaute	Protein Co	12.56109	95	SNRPD3
PINK1	PTEN Indu	Protein Co	12.56109	95	SNU13
ARCN1	Archain 1 (	Protein Co	12.3362	95	SRSF10
HYOU1	Hypoxia U	Protein Co	12.3362	95	TOMM70
KARS1	Lysyl-TRNA	Protein Co	12.3362	95	WDR36
KMT2B	Lysine Met	Protein Co	12.3362	95	ZFR
KRT1	Keratin 1	Protein Co	12.3362	95	LHX1
RPS10	Ribosomal	Protein Co	12.3362	95	CDX2
RPS7	Ribosomal	Protein Co	12.3362	95	MGP
SNRPB	Small Nucl	Protein Co	12.3362	95	TOMM40
TAF15	TATA-Box I	Protein Co	12.3362	95	ABCA13
TMPO	Thymopoie	Protein Co	12.3362	95	AFF3
DMPK	DM1 Prote	Protein Co	9.803947	95	CHST14
ETV6	ETS Varian	Protein Co	9.803947	95	LGMN
PML	PML Nucle	Protein Co	9.212296	95	OASL
FTH1	Ferritin He	Protein Co	9.000925	95	PHLPP1
AAAS	Aladin WD	Protein Co	8.080542	95	PLXNB2
EIF2AK4	Eukaryotic	Protein Co	8.080542	95	SH3BP4
ITPR2	Inositol 1,4	Protein Co	8.080542	95	SIX5
PTPRO	Protein Tyr	Protein Co	8.080542	95	USP4
GHRHR	Growth Hc	Protein Co	5.597071	95	BRDT
CCT2	Chaperonin	Protein Co	5.283573	95	MSH4
WNK1	WNK Lysin	Protein Co	3.560168	95	SPAG1
CTH	Cystathion	Protein Co	1.948301	95	NECTIN3

SRD5A2	Steroid 5 A	Protein Co	25.41485	94	DFFA
MAPK3	Mitogen-A	Protein Co	22.54587	94	ADIPOR1
FTO	FTO Alpha-	Protein Co	21.96308	94	PHF1
ITGB1	Integrin Su	Protein Co	21.77844	94	TET1
RUVBL1	RuvB Like /	Protein Co	20.41674	94	AHRR
MAPK1	Mitogen-A	Protein Co	15.76575	94	DMC1
GSTP1	Glutathion	Protein Co	15.67806	94	INHA
HMGB1	High Mobil	Protein Co	15.6048	94	CMKLR1
CHD7	Chromodo	Protein Co	15.34098	94	ID2
EPCAM	Epithelial (	Protein Co	15.12713	94	ALYREF
PPARGC1A	PPARG Coa	Protein Co	15.05308	94	PRDX6
FOLR1	Folate Rec	Protein Co	14.9643	94	HSD17B7
C7	Compleme	Protein Co	14.95322	94	AQP5
ABCA4	ATP Bindin	Protein Co	14.57622	94	PIP5K1B
CASP9	Caspase 9	Protein Co	14.50259	94	FBL
AGER	Advanced (	Protein Co	14.1634	94	H4C3
RPL18	Ribosomal	Protein Co	13.70661	94	WNT2
TRAP1	TNF Recep	Protein Co	13.70661	94	APOC1
TUFM	Tu Translat	Protein Co	13.70661	94	CYP2J2
NCOA3	Nuclear Re	Protein Co	13.22687	94	PRMT6
NUP62	Nucleopori	Protein Co	13.21224	94	PRX
GABRD	Gamma-Ar	Protein Co	12.85376	94	FHIT
GGT1	Gamma-Gl	Protein Co	12.85376	94	MAS1
UGT2B7	UDP Glucu	Protein Co	12.85376	94	AQP9
PTK2B	Protein Tyı	Protein Co	12.81865	94	IGFBP5
GCLC	Glutamate	Protein Co	12.78118	94	MYH8
SLC12A1	Solute Carı	Protein Co	12.77246	94	SERPINE2
CIITA	Class II Ma	Protein Co	12.74674	94	RAMP1
CREB3L1	CAMP Resp	Protein Co	12.74674	94	ZNF217
SLC11A2	Solute Carı	Protein Co	12.74674	94	ACKR1
KNG1	Kininogen	Protein Co	12.7266	94	ACTR1A
RPL19	Ribosomal	Protein Co	12.56109	94	BCL9
TEAD1	TEA Domai	Protein Co	12.56109	94	CDIPT
APRT	Adenine Pl	Protein Co	12.3362	94	CPNE1
DHCR24	24-Dehydr	Protein Co	12.3362	94	DYNC1LI2
HDAC9	Histone De	Protein Co	12.3362	94	EIF3C
IARS1	Isoleucyl-T	Protein Co	12.3362	94	EIF5
MAPK8	Mitogen-A	Protein Co	12.3362	94	GTPBP4
OAT	Ornithine /	Protein Co	12.3362	94	HIP1R
SMAD9	SMAD Fam	Protein Co	12.3362	94	KTN1
TUBB4B	Tubulin Be	Protein Co	12.3362	94	MYL6
UTRN	Utrophin	Protein Co	12.3362	94	NXN
XPO1	Exportin 1	Protein Co	12.3362	94	PAM
C1QBP	Compleme	Protein Co	9.212296	94	PDIA6
PAH	Phenylalan	Protein Co	9.201195	94	PDS5B
AHI1	Abelson He	Protein Co	8.77603	94	PLRG1
CDC73	Cell Divisio	Protein Co	8.080542	94	PSME2
EYA1	EYA Transc	Protein Co	8.080542	94	RAB1B
GRM5	Glutamate	Protein Co	8.080542	94	RBM14
SPOP	Speckle Ty	Protein Co	5.597071	94	RPL38

GNB4	G Protein S	Protein Co	5.283573	94	SNRPA1
KRT5	Keratin 5	Protein Co	5.283573	94	SNRPD1
MED12	Mediator (	Protein Co	5.283573	94	SNRPF
EEF1A2	Eukaryotic	Protein Co	3.560168	94	HPS5
HCK	HCK Proto-	Protein Co	1.948301	94	DLX6
PRKCB	Protein Kir	Protein Co	1.723406	94	CEP131
BCL2	BCL2 Apop	Protein Co	66.34084	93	SLC25A6
IL6	Interleukin	Protein Co	52.93576	93	TECR
FOXP3	Forkhead E	Protein Co	42.35668	93	XRN2
AMHR2	Anti-Mulle	Protein Co	32.99388	93	ZFYVE9
ABCG2	ATP Bindin	Protein Co	29.13328	93	NUCB1
MBL2	Mannose E	Protein Co	28.07212	93	AP1M2
CYP2C9	Cytochrom	Protein Co	25.21592	93	CEMIP
FMR1	Fragile X M	Protein Co	25.05386	93	GJC1
IL1R1	Interleukin	Protein Co	23.63401	93	HEY2
HLA-C	Major Hist	Protein Co	22.84021	93	HIVEP2
NCOA2	Nuclear Re	Protein Co	22.45599	93	LPP
SYNE1	Spectrin Re	Protein Co	22.19281	93	MTRFR
CHRNA7	Cholinergic	Protein Co	21.11235	93	PPP1R9A
TLR1	Toll Like Re	Protein Co	20.853	93	TLR10
GDNF	Glial Cell D	Protein Co	19.67418	93	TXNL4A
KEAP1	Kelch Like	Protein Co	19.57713	93	BCORL1
BIRC5	Baculoviral	Protein Co	15.81536	93	UBE2B
YAP1	Yes1 Assoc	Protein Co	15.58939	93	ZP3
VCAM1	Vascular C	Protein Co	15.43302	93	FOXO4
ARNT	Aryl Hydro	Protein Co	15.41084	93	F2RL3
RHOA	Ras Homol	Protein Co	15.40639	93	DEPTOR
SERPINF1	Serpin Fam	Protein Co	15.37514	93	EIF6
FOS	Fos Proto-(	Protein Co	15.34875	93	TREM1
CREB1	CAMP Resp	Protein Co	14.96219	93	IL18BP
ANGPT2	Angiopoiet	Protein Co	14.86208	93	KLK6
CYP24A1	Cytochrom	Protein Co	14.84391	93	ARG2
ABCC4	ATP Bindin	Protein Co	14.83142	93	CRY2
FOXA2	Forkhead E	Protein Co	14.76723	93	IL4I1
SMAD7	SMAD Fam	Protein Co	14.76122	93	MARVELD2
CAT	Catalase	Protein Co	14.75795	93	P2RX3
CCR5	C-C Motif (	Protein Co	14.75605	93	MEIS1
PRKCE	Protein Kir	Protein Co	14.74884	93	IL11
PAK4	P21 (RAC1)	Protein Co	14.36836	93	BSN
PPARA	Peroxisom	Protein Co	14.34685	93	TGFB1I1
DUSP6	Dual Specif	Protein Co	14.30099	93	CREM
TNFRSF10E	TNF Recep	Protein Co	14.21427	93	IGSF1
CDK6	Cyclin Dep	Protein Co	14.20047	93	CD200
TOP2A	DNA Topoi	Protein Co	14.11071	93	CRIPTO
HSPA8	Heat Shock	Protein Co	13.70661	93	PIM2
PKM	Pyruvate K	Protein Co	13.70661	93	EIF4EBP1
HDAC3	Histone De	Protein Co	13.44293	93	BCLAF1
BCOR	BCL6 Corep	Protein Co	13.27305	93	EEF1G
MAPK14	Mitogen-A	Protein Co	13.16259	93	HSD17B12
UGT1A3	UDP Glucu	Protein Co	12.85376	93	IPO5

SCN11A	Sodium Vo	Protein Co	12.79398	93	PCBP1
BMP7	Bone Morp	Protein Co	12.76136	93	AFF2
SOD2	Superoxide	Protein Co	12.76136	93	SFRP1
C6	Compleme	Protein Co	12.74674	93	S100A12
MPO	Myeloperc	Protein Co	12.74674	93	BIRC7
AURKB	Aurora Kin	Protein Co	12.7266	93	NCR3
MDM4	MDM4 Reg	Protein Co	12.7266	93	PIEZO2
REV3L	REV3 Like,	Protein Co	12.7266	93	SMOC2
TFG	Trafficking	Protein Co	12.7266	93	WIF1
IRF6	Interferon	Protein Co	12.69709	93	HRH4
DIABLO	Diablo IAP-	Protein Co	12.56109	93	AOC3
CCT5	Chaperonii	Protein Co	12.3362	93	ARL1
DARS1	Aspartyl-TI	Protein Co	12.3362	93	DCXR
DLST	Dihydrolipo	Protein Co	12.3362	93	EIF3D
GBF1	Golgi Brefe	Protein Co	12.3362	93	EXOSC10
GNAI2	G Protein S	Protein Co	12.3362	93	FXR2
IMPDH2	Inosine Mc	Protein Co	12.3362	93	H2AZ1
PRKRA	Protein Act	Protein Co	12.3362	93	HEATR1
TPP1	Tripeptidyl	Protein Co	12.3362	93	HNRNPF
SUZ12	SUZ12 Poly	Protein Co	10.02638	93	MRPS7
SLC40A1	Solute Carr	Protein Co	9.186571	93	PLIN3
GSK3A	Glycogen S	Protein Co	9.16643	93	POLR2H
SYNE2	Spectrin Re	Protein Co	8.77603	93	RRBP1
NECTIN1	Nectin Cell	Protein Co	5.756911	93	SAP18
IRS1	Insulin Rec	Protein Co	5.283573	93	SSB
NR1H3	Nuclear Re	Protein Co	5.283573	93	BRSK1
RARA	Retinoic Ac	Protein Co	2.036903	93	HSPH1
MMP1	Matrix Me	Protein Co	82.40524	92	IRS4
ENO1	Enolase 1	Protein Co	74.4071	92	EBF1
CYP1A1	Cytochrom	Protein Co	68.38834	92	ERI1
CYP2C19	Cytochrom	Protein Co	55.03755	92	ETV1
MMP3	Matrix Me	Protein Co	44.13439	92	EYS
CXCL12	C-X-C Moti	Protein Co	39.4768	92	NSD3
DROSHA	Drosha Rib	Protein Co	30.14911	92	USP48
CYP3A4	Cytochrom	Protein Co	29.64213	92	GAP43
BAX	BCL2 Assoc	Protein Co	28.45698	92	AGFG1
NR4A1	Nuclear Re	Protein Co	22.41384	92	MPRIP
KSR2	Kinase Sup	Protein Co	20.86874	92	NFYA
PDCD1	Programm	Protein Co	15.54082	92	PELP1
BCL2L1	BCL2 Like 1	Protein Co	15.28178	92	NCOA4
YWHAZ	Tyrosine 3-	Protein Co	15.07952	92	TIMELESS
HDAC2	Histone De	Protein Co	15.06064	92	ZP1
EZR	Ezrin	Protein Co	14.85936	92	PGRMC1
CASP1	Caspase 1	Protein Co	14.74884	92	ELAVL2
PLAT	Plasminoge	Protein Co	14.57716	92	POSTN
DHX9	DExH-Box I	Protein Co	14.54983	92	MLLT10
DAPK1	Death Assc	Protein Co	14.19386	92	DDX4
CLU	Clusterin	Protein Co	14.18975	92	MAPK8IP2
MST1R	Macrophag	Protein Co	14.18491	92	KLRK1
JARID2	Jumonji Ar	Protein Co	14.1634	92	PTGES

PRDX1	Peroxiredc	Protein Co	14.1523	92	SULT1E1
C9	Compleme	Protein Co	14.11753	92	NANOG
CSNK1A1	Casein Kin	Protein Co	14.08802	92	GDF15
MATR3	Matrin 3	Protein Co	13.71363	92	VIP
GAPDH	Glyceralde	Protein Co	13.70661	92	STIP1
HSP90AB1	Heat Shock	Protein Co	13.70661	92	USF2
HSP90B1	Heat Shock	Protein Co	13.70661	92	HOXD10
LRPPRC	Leucine Ric	Protein Co	13.70661	92	ENPP3
MOGS	Mannosyl-	Protein Co	13.70661	92	CCDC22
SHC1	SHC Adapt	Protein Co	13.70661	92	ALDH1B1
ACSL4	Acyl-CoA S	Protein Co	13.65203	92	CGAS
CCNE1	Cyclin E1	Protein Co	13.27504	92	HNRNPH2
CYP27B1	Cytochrom	Protein Co	13.23797	92	RND3
UGT1A7	UDP Glucu	Protein Co	12.85376	92	TFAP2C
UGT1A9	UDP Glucu	Protein Co	12.85376	92	LGALS3BP
SMAD1	SMAD Fam	Protein Co	12.8017	92	ICAM2
KITLG	KIT Ligand	Protein Co	12.77246	92	TEAD3
AKAP13	A-Kinase A	Protein Co	12.76136	92	IL23A
ALDH2	Aldehyde D	Protein Co	12.76136	92	LUM
CRYAB	Crystallin A	Protein Co	12.76136	92	SLC15A2
ALOX5	Arachidona	Protein Co	12.74674	92	HDC
ALDH1A3	Aldehyde D	Protein Co	12.7266	92	AZGP1
ATF2	Activating	Protein Co	12.7266	92	H3C1
COMP	Cartilage C	Protein Co	12.69709	92	GREB1L
GH1	Growth Hc	Protein Co	12.64969	92	ARFGAP1
ROR1	Receptor T	Protein Co	12.64969	92	CBX8
PCK2	Phosphoer	Protein Co	12.56109	92	CPSF7
BIRC2	Baculoviral	Protein Co	12.3362	92	EEF1B2
COASY	Coenzyme	Protein Co	12.3362	92	EPPK1
DDOST	Dolichyl-Di	Protein Co	12.3362	92	ESYT1
EIF2B5	Eukaryotic	Protein Co	12.3362	92	GNL3
HSD11B2	Hydroxyste	Protein Co	12.3362	92	H2BC11
IDH3A	Isocitrate D	Protein Co	12.3362	92	HM13
NUP98	Nucleopor	Protein Co	12.3362	92	KRT6B
PABPN1	Poly(A) Bin	Protein Co	12.3362	92	NOP58
PDPK1	3-Phospho	Protein Co	12.3362	92	RAB27B
RARS1	Arginyl-TRI	Protein Co	12.3362	92	SEC22B
SETD1A	SET Domai	Protein Co	12.3362	92	SRPRA
SUPT16H	SPT16 Hon	Protein Co	12.3362	92	TBL3
TKT	Transketol	Protein Co	12.3362	92	TMTC3
UBR4	Ubiquitin F	Protein Co	12.3362	92	TXNDC5
HSP90AA1	Heat Shock	Protein Co	10.18201	92	CAND1
DSG1	Desmoglei	Protein Co	9.212296	92	CPLANE1
MCCC2	Methylcrot	Protein Co	9.212296	92	MSTO1
AMT	Aminomet	Protein Co	8.080542	92	OTUB1
MAP2K5	Mitogen-A	Protein Co	8.080542	92	PIGR
MAPK9	Mitogen-A	Protein Co	8.080542	92	CLINT1
NUP133	Nucleopor	Protein Co	8.080542	92	HOXA3
PLCB2	Phospholip	Protein Co	8.080542	92	RIN3
UBTF	Upstream	Protein Co	8.080542	92	NLRP5

TIA1	TIA1 Cytot	Protein Co	6.174243	92	PIWIL1
MAP3K5	Mitogen-A	Protein Co	6.139478	92	PRDM9
SMARCD1	SWI/SNF R	Protein Co	5.708739	92	CXCR6
ADRB2	Adrenocep	Protein Co	5.694116	92	IL19
CASP7	Caspase 7	Protein Co	5.673974	92	E2F6
GHSR	Growth Hc	Protein Co	5.597071	92	KLK1
TYMS	Thymidylat	Protein Co	4.025672	92	KRT7
SLC2A4	Solute Carı	Protein Co	3.560168	92	IL37
IKBKE	Inhibitor O	Protein Co	2.036903	92	METTL3
FBLN5	Fibulin 5	Protein Co	1.723406	92	ITPK1
ITGA5	Integrin Su	Protein Co	1.723406	92	LINC00473
TNNI3	Troponin I3	Protein Co	1.723406	92	CALD1
PTGS2	Prostaglan	Protein Co	85.77502	91	CCL19
IL1B	Interleukin	Protein Co	83.40623	91	CXCL11
TNF	Tumor Nec	Protein Co	67.84652	91	PFKFB4
WNT4	Wnt Family	Protein Co	61.81068	91	DNMT3L
BCL6	BCL6 Trans	Protein Co	39.38439	91	ATP5F1C
HDAC1	Histone De	Protein Co	39.21866	91	H1-2
TET2	Tet Methyl	Protein Co	38.89457	91	TNFRSF21
PPARD	Peroxisom	Protein Co	23.73092	91	ANXA4
CCND1	Cyclin D1	Protein Co	23.59951	91	IGFBP4
POU5F1	POU Class	Protein Co	22.37338	91	GABRP
FOXP1	Forkhead E	Protein Co	22.19807	91	BAIAP2L1
ETS1	ETS Proto-(	Protein Co	22.16857	91	EN1
XRCC5	X-Ray Repa	Protein Co	21.78715	91	BLMH
PDE1C	Phosphodi	Protein Co	20.86874	91	LIPH
CASP3	Caspase 3	Protein Co	15.86755	91	SGMS1
TNFRSF1B	TNF Recep	Protein Co	15.53629	91	SMPD3
ROCK1	Rho Associ	Protein Co	15.15591	91	MMP11
TNFRSF11E	TNF Recep	Protein Co	14.86537	91	PHLPP2
SIRT3	Sirtuin 3	Protein Co	14.86078	91	SESN2
AURKA	Aurora Kin	Protein Co	14.85419	91	TRPV2
HTRA1	HtrA Serine	Protein Co	14.72048	91	CALB1
TLN1	Talin 1	Protein Co	14.35556	91	BOP1
IRS2	Insulin Rec	Protein Co	14.20047	91	CAPRIN1
PROM1	Prominin 1	Protein Co	14.20047	91	CREB5
RXRA	Retinoid X	Protein Co	14.1974	91	CYP2S1
PRDM1	PR/SET Do	Protein Co	14.18491	91	DDX54
NUCLEOLII	Nucleolin N	Protein Co	13.70661	91	DNAJA3
CDH23	Cadherin R	Protein Co	13.6979	91	DRG1
NME1	NME/NM2	Protein Co	13.46119	91	EIF3M
FGF9	Fibroblast (	Protein Co	13.43715	91	GOLGB1
SGK1	Serum/Glu	Protein Co	13.43013	91	HNRNPA3
GRB2	Growth Fa	Protein Co	13.25948	91	MRPL23
BIRC3	Baculoviral	Protein Co	13.1921	91	PDCD11
GABRA5	Gamma-Ar	Protein Co	12.85376	91	PLIN2
UGT1A6	UDP Glucu	Protein Co	12.85376	91	PSME1
AQP1	Aquaporin	Protein Co	12.79881	91	RBM15
CDH13	Cadherin 1	Protein Co	12.79398	91	SART3
TGFBR3	Transformi	Protein Co	12.78118	91	SOX18

TLR9	Toll Like Re	Protein Co	12.77246	91	TACO1
YES1	YES Proto-(	Protein Co	12.77246	91	TIMM44
RNF213	Ring Finger	Protein Co	12.76136	91	VIL1
LEF1	Lymphoid	Protein Co	12.74674	91	BAG2
ATG7	Autophagy	Protein Co	12.7266	91	SLC30A2
CTSL	Cathepsin	Protein Co	12.7266	91	EIF5B
NR2F1	Nuclear Re	Protein Co	12.7266	91	H2BC21
RRM1	Ribonuclec	Protein Co	12.7266	91	FGD3
SEMA3A	Semaphori	Protein Co	12.7266	91	CDKN2D
TRPM4	Transient F	Protein Co	12.7266	91	CD109
NOD1	Nucleotide	Protein Co	12.64969	91	MMP25
MCL1	MCL1 Apo	Protein Co	12.56109	91	SERAC1
AGK	Acylglycero	Protein Co	12.3362	91	SYMPK
DDX5	DEAD-Box	Protein Co	12.3362	91	WDR6
FBLN1	Fibulin 1	Protein Co	12.3362	91	CATSPER2
IL11RA	Interleukin	Protein Co	12.3362	91	ACKR2
IL17F	Interleukin	Protein Co	12.3362	91	LPAR4
MACF1	Microtubu	Protein Co	12.3362	91	CORO1C
MYO1E	Myosin IE	Protein Co	12.3362	91	IGFBP2
NUP205	Nucleopori	Protein Co	12.3362	91	SLC6A11
PABPC1	Poly(A) Bin	Protein Co	12.3362	91	DHRS4
PTPN1	Protein Tyi	Protein Co	12.3362	91	CCDC39
PUF60	Poly(U) Bir	Protein Co	12.3362	91	IFNA2
RPL10	Ribosomal	Protein Co	12.3362	91	TSLP
VARS1	Valyl-TRNA	Protein Co	12.3362	91	KIR2DL4
PCNA	Proliferatir	Protein Co	9.221008	91	S100A6
DPM1	Dolichyl-Pł	Protein Co	9.212296	91	E4F1
EIF4A1	Eukaryotic	Protein Co	9.212296	91	HOXA9
HSD17B3	Hydroxyste	Protein Co	9.136921	91	EEFSEC
ASXL1	ASXL Trans	Protein Co	8.080542	91	SPIDR
ATP8B1	ATPase Ph	Protein Co	8.080542	91	S100A4
MARK3	Microtubu	Protein Co	8.080542	91	CCL25
SLC19A2	Solute Cari	Protein Co	8.080542	91	CSE1L
TUBB8	Tubulin Be	Protein Co	7.98948	91	APCS
NR0B1	Nuclear Re	Protein Co	5.973973	91	IGF2BP3
CSK	C-Terminal	Protein Co	5.694116	91	PDCD6
S1PR2	Sphingosin	Protein Co	5.694116	91	ID3
ARRB2	Arrestin Be	Protein Co	5.597071	91	IMMT
KIF20A	Kinesin Far	Protein Co	5.597071	91	OLA1
SALL4	Spalt Like 1	Protein Co	5.597071	91	THRAP3
GNB2	G Protein S	Protein Co	5.283573	91	CDH12
PRKAA1	Protein Kir	Protein Co	5.283573	91	AGR2
PRKAR2A	Protein Kir	Protein Co	5.283573	91	MMP10
TMPRSS2	Transmem	Protein Co	3.560168	91	CYP3A43
STS	Steroid Sul	Protein Co	84.35877	90	GABRE
STAR	Steroidoge	Protein Co	43.57452	90	ORM1
CDKN1A	Cyclin Dep	Protein Co	40.6808	90	CKS1B
KISS1R	KISS1 Rece	Protein Co	38.49483	90	NTN4
PTGS1	Prostaglan	Protein Co	27.24489	90	NUCB2
ANXA2	Annexin A2	Protein Co	25.62975	90	AQP8

FOXO1	Forkhead E	Protein Co	23.84846	90	MSLN
LTF	Lactotrans	Protein Co	23.32515	90	TNFRSF17
BSG	Basigin (Ok	Protein Co	22.98556	90	MARCKS
SEPTIN9	Septin 9	Protein Co	22.28371	90	SPAG8
SP1	Sp1 Transc	Protein Co	21.49086	90	KLF6
FOXO3	Forkhead E	Protein Co	21.30064	90	KLRC1
LAMC3	Laminin Su	Protein Co	20.8419	90	TRDMT1
PTPRD	Protein Tyr	Protein Co	20.77763	90	H3C2
TRAF2	TNF Recep	Protein Co	20.36459	90	IFITM1
CYP3A5	Cytochrom	Protein Co	16.9532	90	NAT1
VEGFC	Vascular En	Protein Co	15.05853	90	ATAD3B
KLF4	KLF Transc	Protein Co	14.89737	90	EHD1
NCAM1	Neural Cell	Protein Co	14.85781	90	FNBP1L
APEX1	Apurinic/A	Protein Co	14.85206	90	FSTL1
ANXA1	Annexin A1	Protein Co	14.76264	90	GABPA
CDK1	Cyclin Dep	Protein Co	14.75154	90	HNRNPAB
RACK1	Receptor F	Protein Co	14.56231	90	LSM2
AKR1C2	Aldo-Keto	Protein Co	14.36259	90	LSM4
GZMB	Granzyme	Protein Co	14.21556	90	NDUFA5
GALT	Galactose-	Protein Co	14.18975	90	PABPC4
HNRNPC	Heterogen	Protein Co	14.17913	90	PRRC2A
ATF4	Activating	Protein Co	14.17211	90	QSOX1
MAP2K4	Mitogen-A	Protein Co	14.1634	90	RCC2
EDN1	Endothelin	Protein Co	14.1523	90	UBAP2L
FDXR	Ferredoxin	Protein Co	14.08802	90	UBE2O
PTBP1	Polypyrimi	Protein Co	13.95203	90	HNRNPUL1
CD86	CD86 Mole	Protein Co	13.70661	90	IGHG1
DDB1	Damage Sp	Protein Co	13.70661	90	PDZRN3
FHL1	Four And A	Protein Co	13.70661	90	PGAM5
HNRNPH1	Heterogen	Protein Co	13.70661	90	SEMA4F
LOX	Lysyl Oxida	Protein Co	13.70661	90	TRMT10C
RPS3	Ribosomal	Protein Co	13.70661	90	CRTC2
FOXM1	Forkhead E	Protein Co	13.41032	90	BNIP3
CCR2	C-C Motif (	Protein Co	13.26432	90	GPC5
PDK1	Pyruvate D	Protein Co	13.25948	90	SEPTIN7
KRT8	Keratin 8	Protein Co	13.0266	90	SMYD2
FOXL2	Forkhead E	Protein Co	12.7882	90	DAZAP1
ITGB7	Integrin Su	Protein Co	12.7882	90	RSF1
BGN	Biglycan	Protein Co	12.74674	90	KIR2DL3
FCGR3A	Fc Gamma	Protein Co	12.7266	90	FCHSD2
MTA1	Metastasis	Protein Co	12.69709	90	GDF9
ALDH4A1	Aldehyde D	Protein Co	12.3362	90	CXCL5
C1QA	Compleme	Protein Co	12.3362	90	HPS6
EIF4E	Eukaryotic	Protein Co	12.3362	90	FBXO5
GNAI3	G Protein S	Protein Co	12.3362	90	RHOJ
IQGAP1	IQ Motif Co	Protein Co	12.3362	90	AIF1
PDIA3	Protein Dis	Protein Co	12.3362	90	STOML2
PRPF4	Pre-MRNA	Protein Co	12.3362	90	CXCL6
PRPF6	Pre-MRNA	Protein Co	12.3362	90	DDIT4
PSMC3	Proteasom	Protein Co	12.3362	90	SPRY1

PSMD12	Proteasom	Protein Co	12.3362	90	H4C1
YARS1	Tyrosyl-TR	Protein Co	12.3362	90	ILF2
C4B	Compleme	Protein Co	11.64071	90	MRPS27
CLN3	CLN3 Lysos	Protein Co	10.61539	90	PTP4A3
CD33	CD33 Mole	Protein Co	10.61279	90	KLRD1
TUBA1B	Tubulin Alp	Protein Co	9.212296	90	GCN1
FCGR2A	Fc Gamma	Protein Co	9.201195	90	GABRG1
PPP1R13L	Protein Ph	Protein Co	9.201195	90	PPP1R21
PALLD	Palladin, C	Protein Co	9.136921	90	BCAR3
B4GALNT1	Beta-1,4-N	Protein Co	9.089527	90	TSPAN1
CRB1	Crumbs Ce	Protein Co	8.080542	90	CD83
FZD5	Frizzled Cla	Protein Co	8.080542	90	VEGFD
MAPK11	Mitogen-A	Protein Co	8.080542	90	EBI3
SMARCAL1	SNF2 Relat	Protein Co	8.080542	90	IFITM3
TRDN	Triadin	Protein Co	8.080542	90	KIR2DL1
PRLR	Prolactin R	Protein Co	7.98948	90	MMP24
ARRB1	Arrestin Be	Protein Co	6.174243	90	PEMT
PPIA	Peptidylpr	Protein Co	5.508469	90	PENK
LTA4H	Leukotrien	Protein Co	3.560168	90	MSI2
E2F1	E2F Transc	Protein Co	2.084297	90	CDC42EP1
CSNK1E	Casein Kina	Protein Co	1.723406	90	CHGA
WEE1	WEE1 G2 C	Protein Co	1.723406	90	NMI
AMH	Anti-Mulle	Protein Co	53.43	89	SPRY4
PTPN22	Protein Tyr	Protein Co	41.55924	89	ATG2B
CCR1	C-C Motif (	Protein Co	40.23506	89	AZU1
ALDH1A2	Aldehyde D	Protein Co	29.84968	89	CIRBP
HLA-G	Major Hist	Protein Co	29.60081	89	COPE
IDS	Iduronate 2	Protein Co	28.43023	89	CPSF2
WNT7A	Wnt Family	Protein Co	23.86909	89	DDX46
CYP2B6	Cytochrom	Protein Co	16.28047	89	DPY30
XRCC2	X-Ray Repa	Protein Co	16.0313	89	ECI2
IFNG	Interferon	Protein Co	15.67693	89	H2AC4
TRPV1	Transient F	Protein Co	15.35082	89	H2BC3
CD274	CD274 Mo	Protein Co	15.29309	89	IPO4
TIMP3	TIMP Meta	Protein Co	15.12584	89	NMU
CRHR1	Corticotrop	Protein Co	15.06472	89	PKP3
ERN1	Endoplasm	Protein Co	14.86537	89	RALY
GREM1	Gremlin 1,	Protein Co	14.57047	89	RPL30
JUN	Jun Proto-O	Protein Co	14.48207	89	RSL1D1
CCNB1	Cyclin B1	Protein Co	14.37731	89	S100A7
HLA-DQB1	Major Hist	Protein Co	14.18975	89	SNRPC
EEF1A1	Eukaryotic	Protein Co	14.17913	89	SRPRB
FEN1	Flap Struct	Protein Co	14.17913	89	STAU1
ROBO3	Roundabou	Protein Co	14.1523	89	USP36
RPLP0	Ribosomal	Protein Co	13.70661	89	SHOX
PLAUR	Plasminoge	Protein Co	13.55663	89	ZNF407
GJB2	Gap Juncti	Protein Co	13.45849	89	CDH24
S1PR1	Sphingosin	Protein Co	13.26843	89	AGPAT1
AKR1D1	Aldo-Keto	Protein Co	12.85376	89	EIF4ENIF1
UGT1A8	UDP Glucu	Protein Co	12.85376	89	HELZ

HYAL1	Hyaluronid	Protein Co	12.7882	89	MRPS34
TNFRSF10A	TNF Recep	Protein Co	12.7882	89	RNF17
AQP2	Aquaporin	Protein Co	12.76136	89	ZC3H11A
AVP	Arginine Va	Protein Co	12.76136	89	ZNF598
EXTL3	Exostosin L	Protein Co	12.76136	89	ATF7
FGF10	Fibroblast	Protein Co	12.76136	89	DNAAF19
PARP2	Poly(ADP-R	Protein Co	12.76136	89	FOXA3
CAPN2	Calpain 2	Protein Co	12.74674	89	IP6K2
LTBP2	Latent Tran	Protein Co	12.74674	89	MKRN1
BDKRB2	Bradykinin	Protein Co	12.7266	89	TEX11
CD63	CD63 Mole	Protein Co	12.7266	89	ZP2
CLCN3	Cl-/H+ Anti	Protein Co	12.7266	89	KCNQ1OT1
PLK1	Polo Like K	Protein Co	12.7266	89	CLDN7
APOA2	Apolipopro	Protein Co	12.69709	89	CENPS
CYP26B1	Cytochrom	Protein Co	12.69709	89	LASP1
TLR5	Toll Like Re	Protein Co	12.69709	89	HGFAC
WNT1	Wnt Family	Protein Co	12.69709	89	HLX
RRM2	Ribonucleo	Protein Co	12.64969	89	MIR145
EFEMP1	EGF-Like Fi	Protein Co	12.56109	89	SHBG
CFL2	Cofilin 2	Protein Co	12.3362	89	FCRL3
COPB2	Coat Prote	Protein Co	12.3362	89	KISS1
EIF2B4	Eukaryotic	Protein Co	12.3362	89	HOXA5
EIF2S3	Eukaryotic	Protein Co	12.3362	89	PHF19
ETFA	Electron Tr	Protein Co	12.3362	89	PTMA
KRT14	Keratin 14	Protein Co	12.3362	89	SKAP1
LMNB2	Lamin B2	Protein Co	12.3362	89	RSPO3
NSUN2	NOP2/Sun	Protein Co	12.3362	89	TMOD3
NUP155	Nucleopori	Protein Co	12.3362	89	DUSP2
PSMC5	Proteasom	Protein Co	12.3362	89	MSI1
RPL9	Ribosomal	Protein Co	12.3362	89	SFRP2
RPS27A	Ribosomal	Protein Co	12.3362	89	TMSB4X
SF3B1	Splicing Fa	Protein Co	12.3362	89	CD82
SLC1A5	Solute Carr	Protein Co	12.3362	89	CCL17
SLC25A20	Solute Carr	Protein Co	12.3362	89	SRA1
SLC3A2	Solute Carr	Protein Co	12.3362	89	MOK
TUBB6	Tubulin Be	Protein Co	12.3362	89	CD1A
WDR5	WD Repeat	Protein Co	12.3362	89	INHBB
SLX4	SLX4 Struct	Protein Co	10.61539	89	MUC17
GAS6	Growth Arr	Protein Co	10.61279	89	MIR1-1
BMAL1	Basic Helix	Protein Co	9.712886	89	TRERF1
UBC	Ubiquitin C	Protein Co	9.228029	89	TRIB1
ACLY	ATP Citrate	Protein Co	9.212296	89	HOOK1
CPOX	Coproporp	Protein Co	9.212296	89	NFIL3
MKNK1	MAPK Inte	Protein Co	8.77603	89	PLPP3
COL12A1	Collagen Ty	Protein Co	8.080542	89	CNTF
ERLIN2	ER Lipid Ra	Protein Co	8.080542	89	KLRC2
SBF2	SET Binding	Protein Co	8.080542	89	PPP1R9B
TNKS	Tankyrase	Protein Co	8.080542	89	BYSL
MLH3	MutL Hom	Protein Co	7.98948	89	DDX18
ACTL6A	Actin Like 6	Protein Co	6.927439	89	DDX47

CARM1	Coactivato	Protein Co	6.927439	89	EIF3L
TGM2	Transgluta	Protein Co	5.973973	89	HES5
FOXC1	Forkhead E	Protein Co	5.283573	89	MTDH
GJB1	Gap Juncti	Protein Co	5.283573	89	MTX1
LPAR1	Lysophosp	Protein Co	3.560168	89	PTRH2
FDPS	Farnesyl Di	Protein Co	1.948301	89	RAB21
GNRHR	Gonadotro	Protein Co	125.1018	88	RASAL1
CXCL8	C-X-C Moti	Protein Co	83.77554	88	SRP68
MUC16	Mucin 16,	Protein Co	72.43794	88	SURF4
NPHP4	Nephrocys	Protein Co	43.36673	88	TRMT112
HSPA5	Heat Shock	Protein Co	28.03271	88	YAF2
LTA	Lymphoto›	Protein Co	27.91338	88	PRDX4
SRD5A3	Steroid 5 A	Protein Co	23.46915	88	EIF4E2
CYP2C8	Cytochrom	Protein Co	15.81446	88	GTF3C2
CFL1	Cofilin 1	Protein Co	15.46905	88	PECR
NGFR	Nerve Grov	Protein Co	15.16608	88	PRPS2
TGFBI	Transformi	Protein Co	15.06472	88	RBM25
ALOX15	Arachidona	Protein Co	14.95494	88	SERPINB3
TNFSF10	TNF Superf	Protein Co	14.86874	88	ARFIP2
CEBPB	CCAAT Enh	Protein Co	14.83626	88	CLEC10A
CSF1	Colony Stir	Protein Co	14.76512	88	CSTA
NOX4	NADPH Ox	Protein Co	14.75795	88	NMNAT3
FGF2	Fibroblast	Protein Co	14.70067	88	ATP6V1G2
HNRNPD	Heterogen	Protein Co	14.57047	88	BNIP1
NUMB	NUMB End	Protein Co	14.57047	88	CCN4
IRF5	Interferon	Protein Co	14.54405	88	CKAP2L
HP	Haptoglobi	Protein Co	14.39772	88	KCNG2
HAVCR2	Hepatitis A	Protein Co	14.20047	88	KIF18B
HLA-DQA1	Major Hist	Protein Co	14.19386	88	KLHL9
HLA-DPB1	Major Hist	Protein Co	14.1634	88	MACROD2
CNR1	Cannabino	Protein Co	14.1523	88	MFHAS1
TFAM	Transcripti	Protein Co	14.1523	88	NEGR1
HSPA1A	Heat Shock	Protein Co	13.71941	88	NTSR2
RBMX	RNA Bindir	Protein Co	13.70661	88	RMND1
RPL13	Ribosomal	Protein Co	13.70661	88	INSL3
SRSF1	Serine And	Protein Co	13.70661	88	PACRG
ATG5	Autophagy	Protein Co	13.62252	88	SEPTIN12
PDGFA	Platelet De	Protein Co	13.43013	88	TEX15
DPP4	Dipeptidyl	Protein Co	13.1152	88	USP26
ESRRA	Estrogen R	Protein Co	12.85376	88	CXCL16
PNLIP	Pancreatic	Protein Co	12.85376	88	IL22
UGT1A10	UDP Glucu	Protein Co	12.85376	88	TFPI2
TXN	Thioredoxi	Protein Co	12.80954	88	CDX1
JMJD1C	Jumonji Dc	Protein Co	12.79881	88	SELENBP1
TRPA1	Transient F	Protein Co	12.79398	88	TRIM65
AKR1B1	Aldo-Keto	Protein Co	12.78118	88	
DYSF	Dysferlin	Protein Co	12.77246	88	
CX3CR1	C-X3-C Mo	Protein Co	12.76136	88	
ALOX12	Arachidona	Protein Co	12.74674	88	
NAMPT	Nicotinami	Protein Co	12.7266	88	

SELE	Selectin E	Protein Co	12.7266	88
SLC2A3	Solute Carı	Protein Co	12.7266	88
TRPM3	Transient F	Protein Co	12.7266	88
SCRIB	Scribble Pl	Protein Co	12.69709	88
SLCO2A1	Solute Carı	Protein Co	12.69709	88
CYP2E1	Cytochrom	Protein Co	12.64969	88
EIF3F	Eukaryotic	Protein Co	12.56109	88
KIF5B	Kinesin Far	Protein Co	12.56109	88
AIMP1	Aminoacyl	Protein Co	12.3362	88
C5AR1	Compleme	Protein Co	12.3362	88
DPYSL2	Dihydropyı	Protein Co	12.3362	88
GPX1	Glutathion	Protein Co	12.3362	88
NONO	Non-POU [	Protein Co	12.3362	88
PFKL	Phosphofrı	Protein Co	12.3362	88
RPL13A	Ribosomal	Protein Co	12.3362	88
SETD1B	SET Domai	Protein Co	12.3362	88
TUBA1C	Tubulin Alp	Protein Co	12.3362	88
XRCC6	X-Ray Repa	Protein Co	12.3362	88
TYRO3	TYRO3 Pro	Protein Co	10.61279	88
RARG	Retinoic Ac	Protein Co	10.11745	88
DLX5	Distal-Less	Protein Co	9.233808	88
PKP1	Plakophilin	Protein Co	9.212296	88
PSMD1	Proteasom	Protein Co	9.212296	88
SETX	Senataxin	Protein Co	9.212296	88
TOP1	DNA Topoi	Protein Co	9.212296	88
RAB3GAP1	RAB3 GTPa	Protein Co	9.16643	88
PKP4	Plakophilin	Protein Co	9.136921	88
TRPM6	Transient F	Protein Co	9.089527	88
NEB	Nebulin	Protein Co	8.77603	88
ARHGAP26	Rho GTPas	Protein Co	8.080542	88
ASCC1	Activating	Protein Co	8.080542	88
PRKD2	Protein Kir	Protein Co	8.080542	88
SEC61A1	SEC61 Trar	Protein Co	8.080542	88
TRIP11	Thyroid Hc	Protein Co	8.080542	88
S100B	S100 Calciı	Protein Co	5.597071	88
SKP2	S-Phase Kiı	Protein Co	5.283573	88
AMACR	Alpha-Met	Protein Co	3.560168	88
SOX17	SRY-Box Tr	Protein Co	3.560168	88
THBS2	Thrombos	Protein Co	2.133948	88
CCL2	C-C Motif (	Protein Co	80.95971	87
IL1A	Interleukin	Protein Co	51.90048	87
CRP	C-Reactive	Protein Co	42.49559	87
ALDH1A1	Aldehyde [	Protein Co	42.28304	87
AKR1C3	Aldo-Keto	Protein Co	39.33312	87
IL4	Interleukin	Protein Co	39.24521	87
IL1RAP	Interleukin	Protein Co	21.69008	87
GDAP1	Gangliosid	Protein Co	20.853	87
ATP1B1	ATPase Na	Protein Co	20.80714	87
IL2	Interleukin	Protein Co	15.17107	87
CCN2	Cellular Co	Protein Co	15.06157	87

YBX1	Y-Box Bind	Protein Co	14.94411	87
IL1RL1	Interleukin	Protein Co	14.85609	87
OGG1	8-Oxoguan	Protein Co	14.76393	87
FGF1	Fibroblast	Protein Co	14.70067	87
ACVR1B	Activin A R	Protein Co	14.48476	87
TACR1	Tachykinin	Protein Co	14.38901	87
SLC7A11	Solute Carı	Protein Co	14.30099	87
ATF3	Activating	Protein Co	14.20958	87
CD14	CD14 Mole	Protein Co	14.20555	87
ANTXR2	ANTXR Cel	Protein Co	14.18975	87
SNAI2	Snail Famil	Protein Co	14.13768	87
HSF1	Heat Shock	Protein Co	13.74005	87
RPL6	Ribosomal	Protein Co	13.70661	87
RPS2	Ribosomal	Protein Co	13.70661	87
TIMM50	Translocas	Protein Co	13.70661	87
CRK	CRK Proto-	Protein Co	13.26163	87
BMI1	BMI1 Proto	Protein Co	13.22687	87
ELAVL1	ELAV Like I	Protein Co	13.22687	87
KRT17	Keratin 17	Protein Co	13.1921	87
ADAMTS1(	ADAM Met	Protein Co	12.85376	87
CYP2A6	Cytochrom	Protein Co	12.85376	87
HTR1A	5-Hydroxyt	Protein Co	12.85376	87
PTGIR	Prostaglan	Protein Co	12.85376	87
CCNA2	Cyclin A2	Protein Co	12.81223	87
GCKR	Glucokinas	Protein Co	12.79881	87
CHD5	Chromodo	Protein Co	12.76136	87
LATS1	Large Tum	Protein Co	12.74674	87
E2F3	E2F Transc	Protein Co	12.56109	87
F2R	Coagulatio	Protein Co	12.56109	87
ACOX2	Acyl-CoA C	Protein Co	12.3362	87
EIF4A3	Eukaryotic	Protein Co	12.3362	87
EPS8L2	EPS8 Signa	Protein Co	12.3362	87
ETFB	Electron Tr	Protein Co	12.3362	87
H3-3A	H3.3 Histo	Protein Co	12.3362	87
HNRNPDL	Heterogen	Protein Co	12.3362	87
NCKAP1	NCK Associ	Protein Co	12.3362	87
NHP2	NHP2 Ribo	Protein Co	12.3362	87
PSMA1	Proteasom	Protein Co	12.3362	87
PSMD14	Proteasom	Protein Co	12.3362	87
RBBP4	RB Binding	Protein Co	12.3362	87
RPS14	Ribosomal	Protein Co	12.3362	87
SMCHD1	Structural	Protein Co	12.3362	87
TPR	Translocat	Protein Co	12.3362	87
TRPM2	Transient F	Protein Co	12.3362	87
UGDH	UDP-Gluco	Protein Co	12.3362	87
PCSK5	Proprotein	Protein Co	10.61279	87
GIGYF2	GRB10 Inte	Protein Co	9.212296	87
MCM3	Minichrom	Protein Co	9.212296	87
RPS6	Ribosomal	Protein Co	9.212296	87
FARS2	Phenylalan	Protein Co	8.080542	87

PAK6	P21 (RAC1)	Protein Co	8.080542	87
TLR6	Toll Like Re	Protein Co	8.080542	87
TRIP13	Thyroid Ho	Protein Co	7.98948	87
ARF6	ARF GTPas	Protein Co	5.673974	87
TCF7	Transcripti	Protein Co	5.644465	87
TYROBP	Transmem	Protein Co	5.508469	87
TOR1A	Torsin Fam	Protein Co	3.560168	87
BACE1	Beta-Secre	Protein Co	1.723406	87
CCR3	C-C Motif (	Protein Co	1.723406	87
IL1R2	Interleukin	Protein Co	52.0888	86
MIF	Macrophag	Protein Co	43.90184	86
TET3	Tet Methyl	Protein Co	39.04911	86
XRCC3	X-Ray Repa	Protein Co	28.77804	86
PON1	Paraoxona	Protein Co	22.84557	86
DNM3	Dynamin 3	Protein Co	20.87452	86
DDX6	DEAD-Box	Protein Co	20.41674	86
HOXA1	Homeobox	Protein Co	17.26711	86
MAP3K4	Mitogen-A	Protein Co	16.85657	86
BECN1	Beclin 1	Protein Co	15.63195	86
SPP1	Secreted P	Protein Co	15.62249	86
IGFBP3	Insulin Like	Protein Co	15.55766	86
CD47	CD47 Mole	Protein Co	15.04946	86
FST	Follistatin	Protein Co	14.86717	86
CXCR1	C-X-C Moti	Protein Co	14.60422	86
DKK1	Dickkopf W	Protein Co	14.57047	86
MUC4	Mucin 4, C	Protein Co	14.57047	86
RHOC	Ras Homol	Protein Co	14.38392	86
AEBP1	AE Binding	Protein Co	14.20317	86
FPR2	Formyl Pep	Protein Co	14.19386	86
CLOCK	Clock Circa	Protein Co	14.18975	86
RPL8	Ribosomal	Protein Co	14.17913	86
SEMA3C	Semaphori	Protein Co	14.1634	86
EWSR1	EWS RNA B	Protein Co	13.70661	86
RPL3	Ribosomal	Protein Co	13.70661	86
UQCRC2	Ubiquinol-	Protein Co	13.70661	86
PER2	Period Circ	Protein Co	13.65203	86
PTGER3	Prostaglan	Protein Co	13.24668	86
PLA2G2A	Phospholip	Protein Co	13.21224	86
MEIS2	Meis Home	Protein Co	12.77246	86
PIEZO1	Piezo Type	Protein Co	12.77246	86
CHIT1	Chitinase 1	Protein Co	12.76136	86
FOSB	FosB Proto	Protein Co	12.76136	86
INTU	Inturned P	Protein Co	12.76136	86
LRP8	LDL Recept	Protein Co	12.76136	86
SLC33A1	Solute Carr	Protein Co	12.76136	86
FKBP5	FKBP Proly	Protein Co	12.7266	86
HBEGF	Heparin Bi	Protein Co	12.7266	86
HK2	Hexokinase	Protein Co	12.69709	86
EPHX1	Epoxide Hy	Protein Co	12.64969	86
IGFBP7	Insulin Like	Protein Co	12.64969	86

AIMP2	Aminoacyl	Protein Co	12.3362	86
CANX	Calnexin	Protein Co	12.3362	86
DNAJB11	DnaJ Heat	Protein Co	12.3362	86
GALE	UDP-Galac	Protein Co	12.3362	86
HNRNPR	Heterogen	Protein Co	12.3362	86
NARS1	Asparaginy	Protein Co	12.3362	86
RPL17	Ribosomal	Protein Co	12.3362	86
RPS13	Ribosomal	Protein Co	12.3362	86
SEC31A	SEC31 Hon	Protein Co	12.3362	86
U2AF2	U2 Small N	Protein Co	12.3362	86
UPF1	UPF1 RNA	Protein Co	12.3362	86
PEPD	Peptidase	Protein Co	10.61539	86
C3AR1	Compleme	Protein Co	9.212296	86
DHX30	DExH-Box	Protein Co	9.212296	86
LYZ	Lysozyme	Protein Co	9.212296	86
CALCRL	Calcitonin	Protein Co	8.080542	86
COP1	COP1 E3 U	Protein Co	8.080542	86
PCBP2	Poly(RC) Bi	Protein Co	8.080542	86
RBFOX3	RNA Bindir	Protein Co	8.080542	86
TRPS1	Transcripti	Protein Co	8.080542	86
SOX4	SRY-Box Tr	Protein Co	6.927439	86
PTGER4	Prostaglan	Protein Co	6.614045	86
CD74	CD74 Mole	Protein Co	5.694116	86
NR0B2	Nuclear Re	Protein Co	5.597071	86
AGTR2	Angiotensi	Protein Co	5.283573	86
SNRPN	Small Nucl	Protein Co	5.283573	86
PTHLH	Parathyroi	Protein Co	3.560168	86
PPP2R1B	Protein Ph	Protein Co	1.723406	86
GSTM1	Glutathion	Protein Co	68.36314	85
MMP7	Matrix Me	Protein Co	68.24209	85
AHSG	Alpha 2-HS	Protein Co	67.83094	85
RUVBL2	RuvB Like	Protein Co	55.70293	85
DNAH5	Dynein Axc	Protein Co	43.36673	85
ADIPOQ	Adiponecti	Protein Co	39.20465	85
TRAF1	TNF Recep	Protein Co	22.58313	85
MST1	Macropha	Protein Co	22.24394	85
PTGFR	Prostaglan	Protein Co	21.07974	85
CTSH	Cathepsin	Protein Co	19.35217	85
CYP1A2	Cytochrom	Protein Co	15.81446	85
IL18	Interleukin	Protein Co	15.63805	85
LGALS3	Galectin 3	Protein Co	15.47031	85
IL16	Interleukin	Protein Co	15.33392	85
STRA6	Signaling R	Protein Co	15.09264	85
CXCR3	C-X-C Moti	Protein Co	15.06401	85
ATP5F1B	ATP Syntha	Protein Co	14.94919	85
PFKFB3	6-Phospho	Protein Co	14.76393	85
SIRPA	Signal Regu	Protein Co	14.74577	85
S100A9	S100 Calciu	Protein Co	14.57047	85
BAK1	BCL2 Antag	Protein Co	14.3732	85
ATAD3A	ATPase Far	Protein Co	14.20317	85

SFRP4	Secreted F	Protein Co	14.1523	85
HMGA1	High Mobil	Protein Co	14.08802	85
CADM1	Cell Adhesi	Protein Co	13.7581	85
S100A8	S100 Calciu	Protein Co	13.71941	85
PSMD2	Proteasom	Protein Co	13.70661	85
RAN	RAN, Mem	Protein Co	13.70661	85
SFPQ	Splicing Fa	Protein Co	13.70661	85
SOCS3	Suppressor	Protein Co	13.44293	85
KRT18	Keratin 18	Protein Co	13.27774	85
BAD	BCL2 Assoc	Protein Co	13.24668	85
CTSG	Cathepsin	Protein Co	13.21224	85
AKR1C4	Aldo-Keto	Protein Co	12.85376	85
DMBT1	Deleted In	Protein Co	12.7882	85
SBDS	SBDS Ribos	Protein Co	12.77246	85
DOK1	Docking Pr	Protein Co	12.76136	85
EPO	Erythropoi	Protein Co	12.74674	85
TRIM24	Tripartite M	Protein Co	12.74674	85
CNGB3	Cyclic Nucl	Protein Co	12.7266	85
TFDP1	Transcripti	Protein Co	12.7266	85
TK1	Thymidine	Protein Co	12.7266	85
CSPG4	Chondroiti	Protein Co	12.69709	85
IL12A	Interleukin	Protein Co	12.69709	85
MAP2	Microtubu	Protein Co	12.69709	85
NTN1	Netrin 1	Protein Co	12.69709	85
SELL	Selectin L	Protein Co	12.69709	85
CLDN1	Claudin 1	Protein Co	12.56109	85
ADD1	Adducin 1	Protein Co	12.3362	85
AFDN	Afadin, Adl	Protein Co	12.3362	85
ASH2L	ASH2 Like,	Protein Co	12.3362	85
CCT3	Chaperonin	Protein Co	12.3362	85
EEF1D	Eukaryotic	Protein Co	12.3362	85
ENO2	Enolase 2	Protein Co	12.3362	85
FASTKD2	FAST Kinas	Protein Co	12.3362	85
GOLGA2	Golgin A2	Protein Co	12.3362	85
H1-4	H1.4 Linker	Protein Co	12.3362	85
HDLBP	High Densi	Protein Co	12.3362	85
NOP2	NOP2 Nucl	Protein Co	12.3362	85
PIP4K2A	Phosphatid	Protein Co	12.3362	85
PSMC4	Proteasom	Protein Co	12.3362	85
RAB5C	RAB5C, Me	Protein Co	12.3362	85
RPS11	Ribosomal	Protein Co	12.3362	85
SSRP1	Structure S	Protein Co	12.3362	85
TAF10	TATA-Box I	Protein Co	12.3362	85
TCP1	T-Complex	Protein Co	12.3362	85
TMEM43	Transmem	Protein Co	12.3362	85
NDUFA13	NADH:Ubiq	Protein Co	10.62589	85
TBX6	T-Box Tran	Protein Co	10.61539	85
KCNAB2	Potassium	Protein Co	9.212296	85
LDHB	Lactate De	Protein Co	9.212296	85
RPS18	Ribosomal	Protein Co	9.212296	85

SLC25A1	Solute Carı	Protein Co	9.212296	85
MUC5B	Mucin 5B,	Protein Co	9.186571	85
HTR2B	5-Hydroxyt	Protein Co	9.16643	85
ADGRG6	Adhesion (	Protein Co	8.080542	85
CC2D2A	Coiled-Coil	Protein Co	8.080542	85
DPP6	Dipeptidyl	Protein Co	8.080542	85
FKRP	Fukutin Re	Protein Co	8.080542	85
ITGB8	Integrin Su	Protein Co	8.080542	85
PDE5A	Phosphodi	Protein Co	8.080542	85
PTPN14	Protein Tyı	Protein Co	8.080542	85
DUSP1	Dual Specit	Protein Co	5.508469	85
APOA4	Apolipoprc	Protein Co	5.283573	85
ATL1	Atlastin GT	Protein Co	5.283573	85
NCOA6	Nuclear Re	Protein Co	5.283573	85
MMP8	Matrix Me	Protein Co	3.560168	85
TP53BP2	Tumor Pro	Protein Co	1.723406	85
CCL5	C-C Motif (	Protein Co	67.85857	84
IL33	Interleukin	Protein Co	39.60176	84
F3	Coagulatio	Protein Co	39.30028	84
EGR1	Early Grow	Protein Co	23.11304	84
RUNX3	RUNX Fam	Protein Co	22.45374	84
NR5A2	Nuclear Re	Protein Co	20.72121	84
DDX39B	DExD-Box I	Protein Co	20.41674	84
ANXA5	Annexin A!	Protein Co	15.11716	84
FKBP4	FKBP Proly	Protein Co	15.06157	84
CXCL10	C-X-C Moti	Protein Co	15.05853	84
INHBA	Inhibin Sub	Protein Co	14.84968	84
HMGA2	High Mobil	Protein Co	14.76122	84
PHB1	Prohibitin :	Protein Co	14.75392	84
DDIT3	DNA Dama	Protein Co	14.74884	84
JUNB	JunB Proto	Protein Co	14.20768	84
LY96	Lymphocyt	Protein Co	14.17211	84
SEMA3F	Semaphori	Protein Co	14.1634	84
EIF3B	Eukaryotic	Protein Co	13.70661	84
RPL7	Ribosomal	Protein Co	13.70661	84
RPL7A	Ribosomal	Protein Co	13.70661	84
RPS16	Ribosomal	Protein Co	13.70661	84
SLC25A5	Solute Carı	Protein Co	13.70661	84
SDC1	Syndecan :	Protein Co	13.44293	84
IL13	Interleukin	Protein Co	13.27504	84
SSTR5	Somatosta	Protein Co	13.26843	84
IL18R1	Interleukin	Protein Co	13.24668	84
EIF3A	Eukaryotic	Protein Co	13.0266	84
ERG	ETS Transc	Protein Co	12.85376	84
GABRA3	Gamma-Ar	Protein Co	12.85376	84
GABRA6	Gamma-Ar	Protein Co	12.85376	84
MYOD1	Myogenic I	Protein Co	12.85376	84
NEIL1	Nei Like DI	Protein Co	12.80293	84
KPNB1	Karyopheri	Protein Co	12.78118	84
IL7	Interleukin	Protein Co	12.77246	84

CAPN5	Calpain 5	Protein Co	12.74674	84
GSTA1	Glutathion	Protein Co	12.7266	84
HSD11B1	Hydroxyste	Protein Co	12.69709	84
MSTN	Myostatin	Protein Co	12.69709	84
EML4	EMAP Like	Protein Co	12.56109	84
ACSL3	Acyl-CoA S	Protein Co	12.3362	84
ARF4	ARF GTPas	Protein Co	12.3362	84
ECM1	Extracellul	Protein Co	12.3362	84
EIF5A	Eukaryotic	Protein Co	12.3362	84
ESRRG	Estrogen R	Protein Co	12.3362	84
FXR1	FMR1 Auto	Protein Co	12.3362	84
HNRNPL	Heterogen	Protein Co	12.3362	84
HNRNPM	Heterogen	Protein Co	12.3362	84
HSPA1B	Heat Shock	Protein Co	12.3362	84
KRT2	Keratin 2	Protein Co	12.3362	84
LMO7	LIM Domai	Protein Co	12.3362	84
MYO18A	Myosin XV	Protein Co	12.3362	84
PARP14	Poly(ADP-F	Protein Co	12.3362	84
PSMC2	Proteasom	Protein Co	12.3362	84
PSMC6	Proteasom	Protein Co	12.3362	84
RAB1A	RAB1A, Me	Protein Co	12.3362	84
RING1	Ring Finger	Protein Co	12.3362	84
RPL23A	Ribosomal	Protein Co	12.3362	84
RPS23	Ribosomal	Protein Co	12.3362	84
RPS5	Ribosomal	Protein Co	12.3362	84
SEC13	SEC13 Hon	Protein Co	12.3362	84
SF3B2	Splicing Fa	Protein Co	12.3362	84
SLC27A2	Solute Carr	Protein Co	12.3362	84
SRP72	Signal Reco	Protein Co	12.3362	84
LITAF	Lipopolysa	Protein Co	9.212296	84
PPP2CB	Protein Ph	Protein Co	9.212296	84
SRSF2	Serine And	Protein Co	9.212296	84
UBA52	Ubiquitin A	Protein Co	9.212296	84
HGD	Homogent	Protein Co	9.136921	84
PER1	Period Circ	Protein Co	9.136921	84
AGAP1	ArfGAP Wi	Protein Co	8.080542	84
COL13A1	Collagen T	Protein Co	8.080542	84
DDHD2	DDHD Don	Protein Co	8.080542	84
EIF4B	Eukaryotic	Protein Co	8.080542	84
GPNMB	Glycoprote	Protein Co	8.080542	84
GTF2H1	General Tr	Protein Co	5.673974	84
GPC6	Glypican 6	Protein Co	5.283573	84
CSF2	Colony Stir	Protein Co	3.560168	84
LNPEP	Leucyl And	Protein Co	3.560168	84
GPER1	G Protein-(	Protein Co	43.93745	83
NLRC5	NLR Family	Protein Co	36.8588	83
CDKN2B	Cyclin Dep	Protein Co	29.18711	83
NBAS	NBAS Subu	Protein Co	23.26509	83
METAP2	Methionyl	Protein Co	20.41674	83
FABP4	Fatty Acid	Protein Co	19.95404	83

IDO1	Indoleamir	Protein Co	15.45077	83
MKI67	Marker Of	Protein Co	15.42231	83
FZD7	Frizzled Cla	Protein Co	15.11057	83
MAP1LC3B	Microtubu	Protein Co	14.91721	83
TNFSF13B	TNF Superf	Protein Co	14.74884	83
SLC22A6	Solute Carı	Protein Co	14.55707	83
KLF11	KLF Transc	Protein Co	14.39303	83
AKR1C1	Aldo-Keto	Protein Co	14.38085	83
NAA15	N-Alpha-Ac	Protein Co	14.20317	83
KLK2	Kallikrein F	Protein Co	14.19386	83
MBD2	Methyl-Cp	Protein Co	14.17913	83
ILF3	Interleukin	Protein Co	13.70661	83
RPL10A	Ribosomal	Protein Co	13.70661	83
RPL12	Ribosomal	Protein Co	13.70661	83
RPL4	Ribosomal	Protein Co	13.70661	83
RPS4X	Ribosomal	Protein Co	13.70661	83
RPS8	Ribosomal	Protein Co	13.70661	83
U2AF1	U2 Small N	Protein Co	13.70661	83
SORBS1	Sorbin And	Protein Co	13.6979	83
NECTIN4	Nectin Cell	Protein Co	13.29132	83
CRHR2	Corticotro	Protein Co	13.24668	83
SLIT3	Slit Guidan	Protein Co	13.21224	83
CDA	Cytidine De	Protein Co	13.1921	83
CPT1B	Carnitine P	Protein Co	12.85376	83
GABRA4	Gamma-Ar	Protein Co	12.85376	83
C4BPA	Compleme	Protein Co	12.81462	83
YWHAQ	Tyrosine 3-	Protein Co	12.7882	83
PIAS3	Protein Inh	Protein Co	12.77246	83
RBP1	Retinol Bin	Protein Co	12.77246	83
NUP210	Nucleopori	Protein Co	12.76136	83
CCR6	C-C Motif (	Protein Co	12.74674	83
BCAT1	Branched (	Protein Co	12.7266	83
CEL	Carboxyl E:	Protein Co	12.7266	83
SDC4	Syndecan 4	Protein Co	12.7266	83
ACVR2A	Activin A R	Protein Co	12.69709	83
FAAH	Fatty Acid .	Protein Co	12.64969	83
GAB2	GRB2 Asso	Protein Co	12.56109	83
CHL1	Cell Adhesi	Protein Co	12.3362	83
DDX21	DExD-Box I	Protein Co	12.3362	83
EIF2S1	Eukaryotic	Protein Co	12.3362	83
FLII	FLII Actin R	Protein Co	12.3362	83
GCLM	Glutamate	Protein Co	12.3362	83
GFM2	GTP Deper	Protein Co	12.3362	83
MAN2A1	Mannosida	Protein Co	12.3362	83
MAPRE1	Microtubu	Protein Co	12.3362	83
MSR1	Macropha	Protein Co	12.3362	83
MYBBP1A	MYB Bindi	Protein Co	12.3362	83
PSMD11	Proteasom	Protein Co	12.3362	83
RPL24	Ribosomal	Protein Co	12.3362	83
HSPA4	Heat Shock	Protein Co	9.212296	83

PAK5	P21 (RAC1)	Protein Co	9.212296	83
RPS3A	Ribosomal	Protein Co	9.212296	83
NTF3	Neurotrop	Protein Co	8.87574	83
ANAPC1	Anaphase	Protein Co	8.080542	83
ASTN2	Astrotactir	Protein Co	8.080542	83
CBX5	Chromobo	Protein Co	8.080542	83
NRG3	Neuregulir	Protein Co	8.080542	83
PSMD13	Proteasom	Protein Co	8.080542	83
RAB4A	RAB4A, Me	Protein Co	8.080542	83
SYNJ2	Synaptojar	Protein Co	8.080542	83
STAG3	STAG3 Coh	Protein Co	7.98948	83
SDCBP	Syndecan E	Protein Co	5.644465	83
AVPR1A	Arginine Va	Protein Co	5.508469	83
ADAM12	ADAM Met	Protein Co	5.283573	83
SMC5	Structural	Protein Co	5.283573	83
KCNK9	Potassium	Protein Co	3.560168	83
LTC4S	Leukotrien	Protein Co	3.560168	83
NPAS2	Neuronal F	Protein Co	1.723406	83
PRL	Prolactin	Protein Co	46.71787	82
TIMP1	TIMP Meta	Protein Co	43.93631	82
IL17A	Interleukin	Protein Co	39.05092	82
GC	GC Vitamir	Protein Co	31.05041	82
CPB2	Carboxype	Protein Co	28.64196	82
CNR2	Cannabino	Protein Co	27.04351	82
CXCL1	C-X-C Moti	Protein Co	22.42074	82
SF1	Splicing Fa	Protein Co	21.34886	82
CGA	Glycoprote	Protein Co	20.84324	82
LILRB1	Leukocyte	Protein Co	19.62453	82
HOXA13	Homeobox	Protein Co	18.69593	82
CX3CL1	C-X3-C Mo	Protein Co	15.17051	82
LCN2	Lipocalin 2	Protein Co	15.15888	82
ANG	Angiogenir	Protein Co	15.04733	82
IGF2BP1	Insulin Like	Protein Co	14.82564	82
NODAL	Nodal Grov	Protein Co	14.74223	82
IGF2BP2	Insulin Like	Protein Co	14.7275	82
PHB2	Prohibitin 2	Protein Co	14.6659	82
EIF3E	Eukaryotic	Protein Co	14.53703	82
MT-ND5	Mitochond	Protein Co	14.17211	82
USP10	Ubiquitin S	Protein Co	14.13768	82
TNIP2	TNFAIP3 In	Protein Co	13.73766	82
SLC25A3	Solute Carr	Protein Co	13.70661	82
UGCG	UDP-Gluco	Protein Co	13.68679	82
MGMT	O-6-Methy	Protein Co	13.65203	82
LEFTY2	Left-Right	Protein Co	13.27305	82
SPINK1	Serine Pep	Protein Co	13.24668	82
RPLP1	Ribosomal	Protein Co	13.23797	82
SLIT1	Slit Guidan	Protein Co	13.16259	82
GABRG3	Gamma-Ar	Protein Co	12.85376	82
PTGDR	Prostaglan	Protein Co	12.85376	82
SLCO1A2	Solute Carr	Protein Co	12.85376	82

TXNIP	Thioredoxi	Protein Co	12.7882	82
BMP6	Bone Morp	Protein Co	12.77246	82
CCR7	C-C Motif (	Protein Co	12.77246	82
P2RX1	Purinergic	Protein Co	12.77246	82
FGF7	Fibroblast	Protein Co	12.76136	82
ITIH4	Inter-Alpha	Protein Co	12.74674	82
MFGE8	Milk Fat Gl	Protein Co	12.74674	82
BDKRB1	Bradykinin	Protein Co	12.7266	82
HMOX2	Heme Oxyg	Protein Co	12.7266	82
TRPC1	Transient F	Protein Co	12.7266	82
DNASE1	Deoxyribo	Protein Co	12.64969	82
NPY	Neuropept	Protein Co	12.64969	82
NR4A3	Nuclear Re	Protein Co	12.64969	82
USF1	Upstream	Protein Co	12.64969	82
ADAMTS5	ADAM Met	Protein Co	12.56109	82
WWTR1	WW Doma	Protein Co	12.56109	82
COPG1	Coat Prote	Protein Co	12.3362	82
DDRGK1	DDRGK Do	Protein Co	12.3362	82
G3BP1	G3BP Stres	Protein Co	12.3362	82
GTPBP1	GTP Bindin	Protein Co	12.3362	82
NACA	Nascent Po	Protein Co	12.3362	82
PRPF19	Pre-MRNA	Protein Co	12.3362	82
RAB8A	RAB8A, Me	Protein Co	12.3362	82
RFC3	Replicatior	Protein Co	12.3362	82
RPL14	Ribosomal	Protein Co	12.3362	82
RPL22	Ribosomal	Protein Co	12.3362	82
SQLE	Squalene E	Protein Co	12.3362	82
WNT9B	Wnt Family	Protein Co	10.61539	82
TRIB3	Tribbles Ps	Protein Co	10.61279	82
DDX1	DEAD-Box	Protein Co	9.212296	82
SCO1	Synthesis (	Protein Co	9.212296	82
SLC25A10	Solute Carr	Protein Co	9.212296	82
TRA2B	Transform	Protein Co	9.212296	82
APLNR	Apelin Rec	Protein Co	9.201195	82
HES1	Hes Family	Protein Co	9.136921	82
PPP1R15A	Protein Ph	Protein Co	9.089527	82
GRIK3	Glutamate	Protein Co	8.080542	82
NME7	NME/NM2	Protein Co	8.080542	82
SLC24A4	Solute Carr	Protein Co	8.080542	82
TTK	TTK Protei	Protein Co	6.139478	82
LPAR2	Lysophosp	Protein Co	6.109969	82
MTPAP	Mitochonc	Protein Co	5.508469	82
AP2A2	Adaptor Re	Protein Co	5.283573	82
CD163	CD163 Mo	Protein Co	5.283573	82
WASL	WASP Like	Protein Co	1.948301	82
FADS1	Fatty Acid	Protein Co	1.723406	82
HOXA10	Homeobox	Protein Co	92.32381	81
HSD17B1	Hydroxyste	Protein Co	68.10403	81
GNRH1	Gonadotro	Protein Co	45.01583	81
CCN1	Cellular Co	Protein Co	41.57014	81

GSTM2	Glutathion	Protein Co	40.05973	81
ACKR3	Atypical Ch	Protein Co	29.53073	81
MCAM	Melanoma	Protein Co	28.61554	81
MICA	MHC Class	Protein Co	20.88347	81
CBX3	Chromobo	Protein Co	20.41674	81
HAMP	Hepcidin A	Protein Co	20.19176	81
CCNA1	Cyclin A1	Protein Co	17.21751	81
KRT19	Keratin 19	Protein Co	15.59665	81
NAT2	N-Acetyltra	Protein Co	15.57221	81
NCR1	Natural Cy	Protein Co	15.05605	81
CCL11	C-C Motif (	Protein Co	15.05136	81
PTGIS	Prostaglan	Protein Co	15.04733	81
IL15	Interleukin	Protein Co	14.94919	81
MAP1LC3A	Microtubu	Protein Co	14.75392	81
H2AX	H2A.X Vari	Protein Co	14.74223	81
HPSE	Heparanas	Protein Co	14.74223	81
GHRL	Ghrelin An	Protein Co	14.49289	81
CBR1	Carbonyl R	Protein Co	14.34685	81
PDLIM3	PDZ And LI	Protein Co	14.19386	81
FABP5	Fatty Acid	Protein Co	14.1634	81
ABCF1	ATP Bindin	Protein Co	13.70661	81
SRSF7	Serine And	Protein Co	13.70661	81
VDAC2	Voltage De	Protein Co	13.70661	81
SRF	Serum Res	Protein Co	13.45849	81
F2RL1	F2R Like Tr	Protein Co	13.43715	81
SSTR2	Somatosta	Protein Co	13.26843	81
NAT10	N-Acetyltra	Protein Co	13.0266	81
STOM	Stomatin	Protein Co	13.0266	81
SYNCRIP	Synaptotag	Protein Co	13.0266	81
TSHB	Thyroid Sti	Protein Co	12.85376	81
MUC2	Mucin 2, O	Protein Co	12.80646	81
SMAD5	SMAD Fam	Protein Co	12.8017	81
HYAL2	Hyaluronid	Protein Co	12.7882	81
GRHL2	Grainyhead	Protein Co	12.76136	81
KLK4	Kallikrein F	Protein Co	12.76136	81
SIRT7	Sirtuin 7	Protein Co	12.76136	81
TFEB	Transcripti	Protein Co	12.76136	81
CCR9	C-C Motif (	Protein Co	12.7266	81
CXADR	CXADR Cel	Protein Co	12.7266	81
IL3RA	Interleukin	Protein Co	12.64969	81
KLF10	KLF Transc	Protein Co	12.56109	81
SDC2	Syndecan 2	Protein Co	12.56109	81
CCT7	Chaperonin	Protein Co	12.3362	81
COPB1	Coat Prote	Protein Co	12.3362	81
KHSRP	KH-Type Sp	Protein Co	12.3362	81
KRT9	Keratin 9	Protein Co	12.3362	81
MACROH2	MacroH2A	Protein Co	12.3362	81
MAP4	Microtubu	Protein Co	12.3362	81
NEDD8	NEDD8 Ub	Protein Co	12.3362	81
RAB14	RAB14, Me	Protein Co	12.3362	81

RCC1	Regulator (	Protein Co	12.3362	81
RPL18A	Ribosomal	Protein Co	12.3362	81
RPL27A	Ribosomal	Protein Co	12.3362	81
RPLP2	Ribosomal	Protein Co	12.3362	81
RPN1	Ribophorir	Protein Co	12.3362	81
SARS1	Seryl-TRNA	Protein Co	12.3362	81
SNRPE	Small Nucl	Protein Co	12.3362	81
VDAC3	Voltage De	Protein Co	12.3362	81
ZNFX1	Zinc Finger	Protein Co	12.3362	81
CUX2	Cut Like Hc	Protein Co	9.228029	81
NME4	NME/NM2	Protein Co	9.212296	81
RALGAPA1	Ral GTPase	Protein Co	9.212296	81
SF3B3	Splicing Fa	Protein Co	9.212296	81
SRSF6	Serine And	Protein Co	9.212296	81
TARS1	Threonyl-T	Protein Co	9.212296	81
PYCARD	PYD And C.	Protein Co	9.201195	81
APOD	Apolipoprc	Protein Co	9.136921	81
MPZ	Myelin Pro	Protein Co	9.136921	81
ADAMTS18	ADAM Met	Protein Co	9.089527	81
EXOC2	Exocyst Co	Protein Co	8.080542	81
HOXA2	Homeobox	Protein Co	8.080542	81
MAP3K12	Mitogen-A	Protein Co	8.080542	81
QRICH1	Glutamine	Protein Co	8.080542	81
TARBP2	TARBP2 Su	Protein Co	8.080542	81
CATSPER1	Cation Cha	Protein Co	7.98948	81
DNAH1	Dynein Axc	Protein Co	7.98948	81
ODF2	Outer Den:	Protein Co	7.98948	81
RXFP2	Relaxin Far	Protein Co	7.98948	81
TRADD	TNFRSF1A	Protein Co	6.927439	81
PTGER1	Prostaglan	Protein Co	6.211694	81
TDO2	Tryptophai	Protein Co	5.644465	81
CCR4	C-C Motif (	Protein Co	5.597071	81
NBR1	NBR1 Auto	Protein Co	5.508469	81
HEY1	Hes Relate	Protein Co	1.948301	81
LSR	Lipolysis St	Protein Co	1.948301	81
MUC5AC	Mucin 5AC	Protein Co	1.723406	81
PER3	Period Circ	Protein Co	1.723406	81
REV1	REV1 DNA	Protein Co	1.723406	81
RXFP1	Relaxin Far	Protein Co	85.04151	80
STX5	Syntaxin 5	Protein Co	83.08887	80
IGFBP1	Insulin Like	Protein Co	81.26948	80
LIF	LIF Interleu	Protein Co	44.31711	80
CRH	Corticotro	Protein Co	28.58765	80
THOC6	THO Comp	Protein Co	27.81995	80
PTGER2	Prostaglan	Protein Co	27.53098	80
NPSR1	Neuropept	Protein Co	25.6005	80
FSHB	Follicle Stir	Protein Co	23.32553	80
SRY	Sex Detern	Protein Co	20.73622	80
SNRPD2	Small Nucl	Protein Co	20.41674	80
AKR1A1	Aldo-Keto	Protein Co	19.71564	80

ABO	ABO, Alpha	Protein Co	17.28174	80
PDLIM5	PDZ And LI	Protein Co	17.28174	80
HSD3B1	Hydroxy-D	Protein Co	15.12175	80
MDK	Midkine	Protein Co	15.06157	80
LGALS1	Galectin 1	Protein Co	14.74884	80
SLC22A8	Solute Carı	Protein Co	14.55707	80
STMN1	Stathmin 1	Protein Co	14.20047	80
F11R	F11 Recept	Protein Co	14.1974	80
PF4	Platelet Fa	Protein Co	14.19386	80
TNFRSF6B	TNF Recep	Protein Co	14.19386	80
LGR5	Leucine Ric	Protein Co	14.18975	80
CITED2	Cbp/P300	Protein Co	14.17211	80
TNS1	Tensin 1	Protein Co	14.17211	80
BNC2	Basonuclin	Protein Co	14.1634	80
DHX15	DEAH-Box	Protein Co	13.70661	80
SLC25A11	Solute Carı	Protein Co	13.70661	80
HAND2	Heart And	Protein Co	13.65203	80
LILRB2	Leukocyte	Protein Co	13.65203	80
CCL20	C-C Motif (	Protein Co	13.45188	80
SSTR1	Somatosta	Protein Co	13.26843	80
FIP1L1	Factor Inte	Protein Co	13.0266	80
CBX1	Chromobo	Protein Co	12.85376	80
ICAM3	Intercellula	Protein Co	12.77246	80
PPM1A	Protein Ph	Protein Co	12.76136	80
IL27RA	Interleukin	Protein Co	12.74674	80
ALOX15B	Arachidona	Protein Co	12.7266	80
PLAGL1	PLAG1 Like	Protein Co	12.7266	80
SFN	Stratifin	Protein Co	12.7266	80
THY1	Thy-1 Cell	Protein Co	12.7266	80
TONSL	Tonsoku Li	Protein Co	12.7266	80
GPBAR1	G Protein-(	Protein Co	12.69709	80
MBD1	Methyl-Cp	Protein Co	12.69709	80
SYP	Synaptoph	Protein Co	12.69709	80
HMCN1	Hemicentiı	Protein Co	12.64969	80
ENPEP	Glutamyl A	Protein Co	12.56109	80
NACC1	Nucleus Ac	Protein Co	12.56109	80
SPRY2	Sprouty RT	Protein Co	12.56109	80
ABCE1	ATP Bindin	Protein Co	12.3362	80
ACIN1	Apoptotic	Protein Co	12.3362	80
ACSL5	Acyl-CoA S	Protein Co	12.3362	80
BGLAP	Bone Gamı	Protein Co	12.3362	80
CCT4	Chaperoniı	Protein Co	12.3362	80
CNBP	CCHC-Type	Protein Co	12.3362	80
CPSF6	Cleavage A	Protein Co	12.3362	80
DEK	DEK Proto-	Protein Co	12.3362	80
EIF3I	Eukaryotic	Protein Co	12.3362	80
ELP3	Elongator	Protein Co	12.3362	80
NMT1	N-Myristoy	Protein Co	12.3362	80
PDLIM7	PDZ And LI	Protein Co	12.3362	80
RBBP5	RB Binding	Protein Co	12.3362	80

RPS12	Ribosomal	Protein Co	12.3362	80
SEPTIN2	Septin 2	Protein Co	12.3362	80
SRRT	Serrate, RN	Protein Co	12.3362	80
TKFC	Triokinase	Protein Co	12.3362	80
TMED7	Transmem	Protein Co	12.3362	80
VAPA	VAMP Asso	Protein Co	12.3362	80
BAG5	BAG Cocha	Protein Co	10.16408	80
CACNA2D3	Calcium Vo	Protein Co	9.233808	80
GTF2I	General Tr	Protein Co	9.212296	80
PRPF40A	Pre-MRNA	Protein Co	9.212296	80
CHRDL1	Chordin Lik	Protein Co	9.136921	80
CYP2C18	Cytochrom	Protein Co	9.136921	80
ATG12	Autophagy	Protein Co	8.77603	80
DMXL2	Dmx Like 2	Protein Co	8.77603	80
CAMK1D	Calcium/Ca	Protein Co	8.080542	80
EIF3H	Eukaryotic	Protein Co	8.080542	80
FAF1	Fas Associa	Protein Co	8.080542	80
LRP1B	LDL Recept	Protein Co	8.080542	80
SNX9	Sorting Ne	Protein Co	8.080542	80
TFPI	Tissue Fact	Protein Co	8.080542	80
VPS13B	Vacuolar P	Protein Co	8.080542	80
MSH5	MutS Hom	Protein Co	7.98948	80
PDCD4	Programm	Protein Co	6.109969	80
AFP	Alpha Feto	Protein Co	5.973973	80
ARX	Aristaless F	Protein Co	5.708739	80
ADAM8	ADAM Met	Protein Co	5.694116	80
CHD6	Chromodo	Protein Co	5.283573	80
PMEL	Premelano	Protein Co	5.283573	80
AKT1S1	AKT1 Subs	Protein Co	3.560168	80
MEGF10	Multiple E(	Protein Co	3.560168	80
TCF7L1	Transcripti	Protein Co	2.133948	80
CA1	Carbonic A	Protein Co	1.723406	80
FADS2	Fatty Acid	Protein Co	1.723406	80
PDK2	Pyruvate D	Protein Co	1.723406	80
WNT7B	Wnt Family	Protein Co	1.723406	80
EMX2	Empty Spir	Protein Co	61.22858	79
SAA1	Serum Amy	Protein Co	51.82412	79
HOXA11	Homeobox	Protein Co	49.81292	79
LGALS9	Galectin 9	Protein Co	37.92066	79
SRD5A1	Steroid 5 A	Protein Co	28.44203	79
SNAI1	Snail Famil	Protein Co	26.19835	79
LHB	Luteinizing	Protein Co	23.31488	79
MICB	MHC Class	Protein Co	21.82059	79
MAPK8IP1	Mitogen-A	Protein Co	18.9856	79
TIMP2	TIMP Meta	Protein Co	15.41688	79
CHI3L1	Chitinase 3	Protein Co	15.33677	79
CCL21	C-C Motif (	Protein Co	15.07655	79
ACP1	Acid Phosp	Protein Co	14.75605	79
ID1	Inhibitor O	Protein Co	14.74884	79
ADM	Adrenome	Protein Co	14.38901	79

MRC1	Mannose F	Protein Co	14.20555	79
CEBPD	CCAAT Enh	Protein Co	14.1974	79
SERPINA6	Serpin Fam	Protein Co	14.1634	79
CSMD1	CUB And S	Protein Co	13.71941	79
LARP1	La Ribonuc	Protein Co	13.70661	79
FSCN1	Fascin Acti	Protein Co	13.1921	79
CYP3A7	Cytochrom	Protein Co	12.85376	79
COPS5	COP9 Signa	Protein Co	12.79398	79
PMS1	PMS1 Hom	Protein Co	12.79398	79
PRMT2	Protein Arg	Protein Co	12.76136	79
PGF	Placental G	Protein Co	12.74674	79
MMP12	Matrix Me	Protein Co	12.7266	79
CXXC1	CXXC Finge	Protein Co	12.69709	79
CCR8	C-C Motif (	Protein Co	12.64969	79
DCLK1	Doublecort	Protein Co	12.64969	79
AHCTF1	AT-Hook C	Protein Co	12.3362	79
ATP5PO	ATP Syntha	Protein Co	12.3362	79
BAG6	BAG Cocha	Protein Co	12.3362	79
CCT8	Chaperonin	Protein Co	12.3362	79
CNP	2',3'-Cyclic	Protein Co	12.3362	79
DDX17	DEAD-Box	Protein Co	12.3362	79
EIF2S2	Eukaryotic	Protein Co	12.3362	79
FARSA	Phenylalan	Protein Co	12.3362	79
G3BP2	G3BP Stres	Protein Co	12.3362	79
GTF2F1	General Tr	Protein Co	12.3362	79
KHDRBS1	KH RNA Bin	Protein Co	12.3362	79
NPEPPS	Aminopept	Protein Co	12.3362	79
NXF1	Nuclear RN	Protein Co	12.3362	79
SNRPD3	Small Nucle	Protein Co	12.3362	79
SNU13	Small Nucle	Protein Co	12.3362	79
SRSF10	Serine And	Protein Co	12.3362	79
TOMM70	Translocas	Protein Co	12.3362	79
WDR36	WD Repeat	Protein Co	12.3362	79
ZFR	Zinc Finger	Protein Co	12.3362	79
LHX1	LIM Homeo	Protein Co	10.61539	79
CDX2	Caudal Typ	Protein Co	10.61279	79
MGP	Matrix Gla	Protein Co	10.61279	79
TOMM40	Translocas	Protein Co	9.212296	79
ABCA13	ATP Bindin	Protein Co	8.77603	79
AFF3	ALF Transc	Protein Co	8.080542	79
CHST14	Carbohydra	Protein Co	8.080542	79
LGMN	Legumain	Protein Co	8.080542	79
OASL	2'-5'-Oligoa	Protein Co	8.080542	79
PHLPP1	PH Domain	Protein Co	8.080542	79
PLXNB2	Plexin B2	Protein Co	8.080542	79
SH3BP4	SH3 Domai	Protein Co	8.080542	79
SIX5	SIX Homeo	Protein Co	8.080542	79
USP4	Ubiquitin S	Protein Co	8.080542	79
BRDT	Bromodom	Protein Co	7.98948	79
MSH4	MutS Hom	Protein Co	7.98948	79

SPAG1	Sperm Ass	Protein Co	7.98948	79
NECTIN3	Nectin Cell	Protein Co	5.756911	79
DFFA	DNA Fragm	Protein Co	5.708739	79
ADIPOR1	Adiponecti	Protein Co	5.673974	79
PHF1	PHD Finger	Protein Co	1.723406	79
TET1	Tet Methyl	Protein Co	38.90925	78
AHRR	Aryl Hydro	Protein Co	25.84181	78
DMC1	DNA Meiot	Protein Co	24.02078	78
INHA	Inhibin Sub	Protein Co	20.77768	78
CMKLR1	Chemerin (	Protein Co	14.75795	78
ID2	Inhibitor O	Protein Co	14.5745	78
ALYREF	Aly/REF Ex	Protein Co	14.17913	78
CYP26A1	Cytochrom	Protein Co	14.17211	78
PRDX6	Peroxiredo	Protein Co	14.1634	78
HSD17B7	Hydroxyste	Protein Co	14.1523	78
AQP5	Aquaporin	Protein Co	13.95203	78
PIP5K1B	Phosphatic	Protein Co	13.73497	78
FBL	Fibrillarin F	Protein Co	13.70661	78
H4C3	H4 Cluster	Protein Co	13.70661	78
WNT2	Wnt Family	Protein Co	13.62252	78
APOC1	Apolipopro	Protein Co	12.85376	78
CYP2J2	Cytochrom	Protein Co	12.85376	78
PRMT6	Protein Arg	Protein Co	12.79881	78
PRX	Periaxin	Protein Co	12.78118	78
CXCL9	C-X-C Moti	Protein Co	12.77246	78
FHIT	Fragile Hist	Protein Co	12.76136	78
MAS1	MAS1 Prot	Protein Co	12.76136	78
AQP9	Aquaporin	Protein Co	12.74674	78
IGFBP5	Insulin Like	Protein Co	12.74674	78
MYH8	Myosin He	Protein Co	12.74674	78
CD226	CD226 Mo	Protein Co	12.7266	78
SERPINE2	Serpin Fam	Protein Co	12.7266	78
RAMP1	Receptor A	Protein Co	12.69709	78
ZNF217	Zinc Finger	Protein Co	12.64969	78
ACKR1	Atypical Ch	Protein Co	12.3362	78
ACTR1A	Actin Relat	Protein Co	12.3362	78
BCL9	BCL9 Trans	Protein Co	12.3362	78
CDIPT	CDP-Diacyl	Protein Co	12.3362	78
CPNE1	Copine 1	Protein Co	12.3362	78
DYNC1LI2	Dynein Cyt	Protein Co	12.3362	78
EIF3C	Eukaryotic	Protein Co	12.3362	78
EIF5	Eukaryotic	Protein Co	12.3362	78
GTPBP4	GTP Bindin	Protein Co	12.3362	78
HIP1R	Huntingtin	Protein Co	12.3362	78
KTN1	Kinectin 1	Protein Co	12.3362	78
MYL6	Myosin Lig	Protein Co	12.3362	78
NXN	Nucleored	Protein Co	12.3362	78
PAM	Peptidylgly	Protein Co	12.3362	78
PDIA6	Protein Dis	Protein Co	12.3362	78
PDS5B	PDS5 Cohe	Protein Co	12.3362	78

PLRG1	Pleiotropic	Protein Co	12.3362	78
PSME2	Proteasom	Protein Co	12.3362	78
RAB1B	RAB1B, Me	Protein Co	12.3362	78
RBM14	RNA Bindir	Protein Co	12.3362	78
RPL38	Ribosomal	Protein Co	12.3362	78
SNRPA1	Small Nucl	Protein Co	12.3362	78
SNRPD1	Small Nucl	Protein Co	12.3362	78
SNRPF	Small Nucl	Protein Co	12.3362	78
HPS5	HPS5 Bioge	Protein Co	10.61539	78
DLX6	Distal-Less	Protein Co	9.233808	78
CEP131	Centrosom	Protein Co	9.228029	78
SLC25A6	Solute Carr	Protein Co	9.212296	78
TECR	Trans-2,3-E	Protein Co	9.212296	78
XRN2	5'-3' Exorib	Protein Co	9.212296	78
ZFYVE9	Zinc Finger	Protein Co	9.212296	78
NUCB1	Nucleobinc	Protein Co	8.77603	78
AP1M2	Adaptor Re	Protein Co	8.080542	78
CEMIP	Cell Migrat	Protein Co	8.080542	78
GJC1	Gap Juncti	Protein Co	8.080542	78
HEY2	Hes Relate	Protein Co	8.080542	78
HIVEP2	HIVEP Zinc	Protein Co	8.080542	78
LPP	LIM Domai	Protein Co	8.080542	78
MTRFR	Mitochond	Protein Co	8.080542	78
PPP1R9A	Protein Ph	Protein Co	8.080542	78
TLR10	Toll Like Re	Protein Co	8.080542	78
TXNL4A	Thioredoxi	Protein Co	8.080542	78
BCORL1	BCL6 Corep	Protein Co	7.98948	78
UBE2B	Ubiquitin C	Protein Co	7.98948	78
ZP3	Zona Pellu	Protein Co	7.98948	78
FOXO4	Forkhead E	Protein Co	6.927439	78
F2RL3	F2R Like Th	Protein Co	5.673974	78
DEPTOR	DEP Doma	Protein Co	5.283573	78
EIF6	Eukaryotic	Protein Co	5.283573	78
TREM1	Triggering	Protein Co	5.283573	78
IL18BP	Interleukin	Protein Co	3.560168	78
KLK6	Kallikrein F	Protein Co	1.948301	78
ARG2	Arginase 2	Protein Co	1.723406	78
CRY2	Cryptochrc	Protein Co	1.723406	78
IL4I1	Interleukin	Protein Co	1.723406	78
MARVELD2	MARVEL D	Protein Co	1.723406	78
P2RX3	Purinergic	Protein Co	28.76024	77
MEIS1	Meis Home	Protein Co	21.33424	77
IL11	Interleukin	Protein Co	21.23616	77
BSN	Bassoon Pr	Protein Co	20.853	77
TGFB1I1	Transformi	Protein Co	19.6888	77
CREM	CAMP Resp	Protein Co	18.14872	77
IGSF1	Immunoglo	Protein Co	15.35184	77
GPX3	Glutathion	Protein Co	14.94411	77
CD200	CD200 Mo	Protein Co	14.48245	77
CRIPTO	Cripto, EGF	Protein Co	14.38662	77

PIM2	Pim-2 Prot	Protein Co	14.18491	77
EIF4EBP1	Eukaryotic	Protein Co	14.17211	77
BCLAF1	BCL2 Assoc	Protein Co	13.70661	77
EEF1G	Eukaryotic	Protein Co	13.70661	77
HSD17B12	Hydroxyste	Protein Co	13.70661	77
IPO5	Importin 5	Protein Co	13.70661	77
PCBP1	Poly(RC) Bi	Protein Co	13.70661	77
AFF2	ALF Transc	Protein Co	13.62252	77
SFRP1	Secreted F	Protein Co	13.24668	77
S100A12	S100 Calciu	Protein Co	13.16259	77
BIRC7	Baculoviral	Protein Co	12.7882	77
NCR3	Natural Cy	Protein Co	12.7882	77
PIEZO2	Piezo Type	Protein Co	12.76136	77
SMOC2	SPARC Rela	Protein Co	12.74674	77
WIF1	Wnt Inhibi	Protein Co	12.7266	77
HRH4	Histamine	Protein Co	12.69709	77
AOC3	Amine Oxi	Protein Co	12.64969	77
ARL1	ARF Like G	Protein Co	12.3362	77
DCXR	Dicarbonyl	Protein Co	12.3362	77
EIF3D	Eukaryotic	Protein Co	12.3362	77
EXOSC10	Exosome C	Protein Co	12.3362	77
FXR2	FMR1 Auto	Protein Co	12.3362	77
H2AZ1	H2A.Z Vari	Protein Co	12.3362	77
HEATR1	HEAT Repe	Protein Co	12.3362	77
HNRNPF	Heterogen	Protein Co	12.3362	77
MRPS7	Mitochonc	Protein Co	12.3362	77
PLIN3	Perilipin 3	Protein Co	12.3362	77
POLR2H	RNA Polym	Protein Co	12.3362	77
RRBP1	Ribosome	Protein Co	12.3362	77
SAP18	Sin3A Asso	Protein Co	12.3362	77
SSB	Small RNA	Protein Co	12.3362	77
BRSK1	BR Serine/	Protein Co	9.212296	77
HSPH1	Heat Shock	Protein Co	9.212296	77
IRS4	Insulin Rec	Protein Co	9.212296	77
EBF1	EBF Transc	Protein Co	8.080542	77
ERI1	Exoribonu	Protein Co	8.080542	77
ETV1	ETS Varian	Protein Co	8.080542	77
EYS	EGF-Like P	Protein Co	8.080542	77
NSD3	Nuclear Re	Protein Co	8.080542	77
USP48	Ubiquitin S	Protein Co	8.080542	77
GAP43	Growth As	Protein Co	5.728552	77
AGFG1	ArfGAP Wi	Protein Co	5.283573	77
MPRIP	Myosin Ph	Protein Co	5.283573	77
NFYA	Nuclear Tr	Protein Co	5.283573	77
PELP1	Proline, Gl	Protein Co	3.560168	77
NCOA4	Nuclear Re	Protein Co	1.723406	77
TIMELESS	Timeless C	Protein Co	1.723406	77
HSD17B2	Hydroxyste	Protein Co	68.4773	76
ZP1	Zona Pellu	Protein Co	43.36673	76
PGRMC1	Progesterc	Protein Co	30.62147	76

ELAVL2	ELAV Like I	Protein Co	24.09744	76
POSTN	Periostin	Protein Co	21.49375	76
MLLT10	MLLT10 Hi	Protein Co	20.853	76
DDX4	DEAD-Box	Protein Co	20.73622	76
MAPK8IP2	Mitogen-A	Protein Co	18.9856	76
KLRK1	Killer Cell L	Protein Co	15.49385	76
PTGES	Prostaglan	Protein Co	15.32338	76
SULT1E1	Sulfotransf	Protein Co	15.05308	76
NANOG	Nanog Hor	Protein Co	14.9625	76
GDF15	Growth Dif	Protein Co	14.84698	76
VIP	Vasoactive	Protein Co	14.2113	76
STIP1	Stress Indu	Protein Co	14.19386	76
USF2	Upstream T	Protein Co	14.19386	76
HOXD10	Homeobox	Protein Co	14.13768	76
ENPP3	Ectonucleo	Protein Co	14.08802	76
CCDC22	CCC Compl	Protein Co	13.6979	76
ALDH1B1	Aldehyde D	Protein Co	13.68679	76
CGAS	Cyclic GMP	Protein Co	13.1152	76
HNRNPH2	Heterogen	Protein Co	13.0266	76
RND3	Rho Family	Protein Co	12.7882	76
TFAP2C	Transcripti	Protein Co	12.7882	76
LGALS3BP	Galectin 3	Protein Co	12.78118	76
ICAM2	Intercellula	Protein Co	12.77246	76
TEAD3	TEA Domai	Protein Co	12.77246	76
IL23A	Interleukin	Protein Co	12.74674	76
LUM	Lumican	Protein Co	12.74674	76
SLC15A2	Solute Carr	Protein Co	12.7266	76
HDC	Histidine D	Protein Co	12.69709	76
AZGP1	Alpha-2-Gl	Protein Co	12.64969	76
H3C1	H3 Cluster	Protein Co	12.64969	76
GREB1L	GREB1 Like	Protein Co	12.64662	76
ARFGAP1	ARF GTPas	Protein Co	12.3362	76
CBX8	Chromobo	Protein Co	12.3362	76
CPSF7	Cleavage A	Protein Co	12.3362	76
EEF1B2	Eukaryotic	Protein Co	12.3362	76
EPPK1	Epiplakin 1	Protein Co	12.3362	76
ESYT1	Extended S	Protein Co	12.3362	76
GNL3	G Protein N	Protein Co	12.3362	76
H2BC11	H2B Cluste	Protein Co	12.3362	76
HM13	Histocomp	Protein Co	12.3362	76
KRT6B	Keratin 6B	Protein Co	12.3362	76
NOP58	NOP58 Rib	Protein Co	12.3362	76
RAB27B	RAB27B, M	Protein Co	12.3362	76
SEC22B	SEC22 Hon	Protein Co	12.3362	76
SRPRA	SRP Recep	Protein Co	12.3362	76
TBL3	Transducin	Protein Co	12.3362	76
TMTC3	Transmem	Protein Co	12.3362	76
TXNDC5	Thioredoxi	Protein Co	12.3362	76
CAND1	Cullin Asso	Protein Co	9.212296	76
CPLANE1	Ciliogenesi	Protein Co	9.212296	76

MSTO1	Misato Mit	Protein Co	9.212296	76
OTUB1	OTU Deubi	Protein Co	9.212296	76
PIGR	Polymeric	Protein Co	9.212296	76
CLINT1	Clathrin Int	Protein Co	8.080542	76
HOXA3	Homeobox	Protein Co	8.080542	76
RIN3	Ras And Ra	Protein Co	8.080542	76
NLRP5	NLR Family	Protein Co	7.98948	76
PIWIL1	Piwi Like R	Protein Co	7.98948	76
PRDM9	PR/SET Do	Protein Co	7.98948	76
CXCR6	C-X-C Moti	Protein Co	6.174243	76
IL19	Interleukin	Protein Co	5.708739	76
E2F6	E2F Transc	Protein Co	5.283573	76
KLK1	Kallikrein 1	Protein Co	1.948301	76
KRT7	Keratin 7	Protein Co	50.31124	75
IL37	Interleukin	Protein Co	23.62015	75
METTL3	Methyltrar	Protein Co	22.70694	75
ITPK1	Inositol-Te	Protein Co	20.86874	75
LINC00473	Long Interg	RNA Gene	19.65404	75
CALD1	Caldesmor	Protein Co	14.20768	75
CCL19	C-C Motif (	Protein Co	14.20317	75
CXCL11	C-X-C Moti	Protein Co	14.20317	75
PFKFB4	6-Phospho	Protein Co	14.1523	75
DNMT3L	DNA Meth	Protein Co	14.13768	75
ATP5F1C	ATP Syntha	Protein Co	13.70661	75
H1-2	H1.2 Linke	Protein Co	13.70661	75
TNFRSF21	TNF Recep	Protein Co	13.62252	75
ANXA4	Annexin A4	Protein Co	13.2537	75
CCL22	C-C Motif (	Protein Co	13.2537	75
IGFBP4	Insulin Like	Protein Co	13.16259	75
GABRP	Gamma-Ar	Protein Co	12.85376	75
BAIAP2L1	BAR/IMD D	Protein Co	12.79881	75
EN1	Engrailed H	Protein Co	12.76136	75
BLMH	Bleomycin	Protein Co	12.74674	75
LIPH	Lipase H	Protein Co	12.74674	75
SGMS1	Sphingomy	Protein Co	12.74674	75
SMPD3	Sphingomy	Protein Co	12.74674	75
MMP11	Matrix Me	Protein Co	12.7266	75
PHLPP2	PH Domain	Protein Co	12.7266	75
SESN2	Sestrin 2	Protein Co	12.7266	75
TRPV2	Transient F	Protein Co	12.7266	75
CALB1	Calbindin 1	Protein Co	12.64969	75
BOP1	BOP1 Ribo	Protein Co	12.3362	75
CAPRIN1	Cell Cycle A	Protein Co	12.3362	75
CREB5	CAMP Resp	Protein Co	12.3362	75
CYP2S1	Cytochrom	Protein Co	12.3362	75
DDX54	DEAD-Box	Protein Co	12.3362	75
DNAJA3	DnaJ Heat	Protein Co	12.3362	75
DRG1	Developme	Protein Co	12.3362	75
EIF3M	Eukaryotic	Protein Co	12.3362	75
GOLGB1	Golgin B1	Protein Co	12.3362	75

HNRNPA3	Heterogen	Protein Co	12.3362	75
MRPL23	Mitochonc	Protein Co	12.3362	75
PDCD11	Programm	Protein Co	12.3362	75
PLIN2	Perilipin 2	Protein Co	12.3362	75
PSME1	Proteasom	Protein Co	12.3362	75
RBM15	RNA Bindir	Protein Co	12.3362	75
SART3	Spliceoson	Protein Co	12.3362	75
SOX18	SRY-Box Tr	Protein Co	12.3362	75
TACO1	Translatior	Protein Co	12.3362	75
TIMM44	Translocas	Protein Co	12.3362	75
VIL1	Villin 1	Protein Co	12.3362	75
BAG2	BAG Cocha	Protein Co	9.233808	75
SLC30A2	Solute Carr	Protein Co	9.228029	75
EIF5B	Eukaryotic	Protein Co	9.212296	75
H2BC21	H2B Cluste	Protein Co	9.212296	75
FGD3	FYVE, Rho(	Protein Co	9.089527	75
CDKN2D	Cyclin Dep	Protein Co	8.77603	75
CD109	CD109 Mo	Protein Co	8.080542	75
MMP25	Matrix Me	Protein Co	8.080542	75
SERAC1	Serine Acti	Protein Co	8.080542	75
SYMPK	Symplekin	Protein Co	8.080542	75
WDR6	WD Repea	Protein Co	8.080542	75
CATSPER2	Cation Cha	Protein Co	7.98948	75
ACKR2	Atypical Ch	Protein Co	6.927439	75
LPAR4	Lysophosp	Protein Co	5.644465	75
CORO1C	Coronin 1C	Protein Co	5.283573	75
IGFBP2	Insulin Like	Protein Co	3.560168	75
SLC6A11	Solute Carr	Protein Co	1.723406	75
DHRS4	Dehydroge	Protein Co	149.1267	74
CCDC39	Coiled-Coil	Protein Co	43.36673	74
IFNA2	Interferon	Protein Co	39.23323	74
TSLP	Thymic Str	Protein Co	28.92567	74
KIR2DL4	Killer Cell I	Protein Co	27.6901	74
S100A6	S100 Calciu	Protein Co	22.02419	74
E4F1	E4F Transc	Protein Co	20.73024	74
HOXA9	Homeobox	Protein Co	20.64163	74
EEFSEC	Eukaryotic	Protein Co	20.41674	74
SPIDR	Scaffold Pr	Protein Co	16.0313	74
S100A4	S100 Calciu	Protein Co	15.24674	74
CCL25	C-C Motif (	Protein Co	14.84037	74
CSE1L	Chromosoı	Protein Co	14.55467	74
APCS	Amyloid P	Protein Co	14.43389	74
IGF2BP3	Insulin Like	Protein Co	14.35556	74
PDCD6	Programm	Protein Co	14.18491	74
ID3	Inhibitor O	Protein Co	13.72836	74
IMMT	Inner Mem	Protein Co	13.70661	74
OLA1	Obg Like A	Protein Co	13.70661	74
THRAP3	Thyroid Ho	Protein Co	13.70661	74
CDH12	Cadherin 1	Protein Co	13.6979	74
AGR2	Anterior G	Protein Co	13.67217	74

MMP10	Matrix Me	Protein Co	13.22687	74
CYP3A43	Cytochrom	Protein Co	12.85376	74
GABRE	Gamma-Ar	Protein Co	12.85376	74
ORM1	Orosomuc	Protein Co	12.85376	74
CKS1B	CDC28 Pro	Protein Co	12.79398	74
NTN4	Netrin 4	Protein Co	12.78118	74
NUCB2	Nucleobin	Protein Co	12.76136	74
AQP8	Aquaporin	Protein Co	12.74674	74
MSLN	Mesothelir	Protein Co	12.74674	74
TNFRSF17	TNF Recep	Protein Co	12.74674	74
MARCKS	Myristoyla	Protein Co	12.7266	74
SPAG8	Sperm Ass	Protein Co	12.7266	74
CXCL13	C-X-C Moti	Protein Co	12.69709	74
KLF6	KLF Transc	Protein Co	12.69709	74
KLRC1	Killer Cell L	Protein Co	12.69709	74
TRDMT1	TRNA Aspa	Protein Co	12.69709	74
H3C2	H3 Cluster	Protein Co	12.64969	74
IFITM1	Interferon	Protein Co	12.64969	74
NAT1	N-Acetyltra	Protein Co	12.64969	74
ATAD3B	ATPase Far	Protein Co	12.3362	74
EHD1	EH Domair	Protein Co	12.3362	74
FNBP1L	Formin Bin	Protein Co	12.3362	74
FSTL1	Follistatin I	Protein Co	12.3362	74
GABPA	GA Binding	Protein Co	12.3362	74
HNRNPAB	Heterogen	Protein Co	12.3362	74
LSM2	LSM2 Hom	Protein Co	12.3362	74
LSM4	LSM4 Hom	Protein Co	12.3362	74
NDUFA5	NADH:Ubi	Protein Co	12.3362	74
PABPC4	Poly(A) Bin	Protein Co	12.3362	74
PRRC2A	Proline Ric	Protein Co	12.3362	74
QSOX1	Quiescin S	Protein Co	12.3362	74
RCC2	Regulator (	Protein Co	12.3362	74
UBAP2L	Ubiquitin A	Protein Co	12.3362	74
UBE2O	Ubiquitin C	Protein Co	12.3362	74
HNRNPUL1	Heterogen	Protein Co	9.212296	74
IGHG1	Immunogl	Protein Co	9.212296	74
PDZRN3	PDZ Doma	Protein Co	9.212296	74
PGAM5	PGAM Fan	Protein Co	9.212296	74
SEMA4F	Ssemaphoi	Protein Co	9.212296	74
TRMT10C	TRNA Metl	Protein Co	9.212296	74
CRTC2	CREB Regu	Protein Co	9.186571	74
BNIP3	BCL2 Inter	Protein Co	9.16643	74
DIO2	Iodothyror	Protein Co	9.136921	74
GPC5	Glypican 5	Protein Co	8.080542	74
SEPTIN7	Septin 7	Protein Co	8.080542	74
SMYD2	SET And M	Protein Co	8.080542	74
DAZAP1	DAZ Associ	Protein Co	7.98948	74
RSF1	Remodelin	Protein Co	6.927439	74
KIR2DL3	Killer Cell I	Protein Co	5.283573	74
FCHSD2	FCH And D	Protein Co	1.723406	74

GDF9	Growth Dif	Protein Co	46.96469	73
CXCL5	C-X-C Moti	Protein Co	43.84458	73
MIR21	MicroRNA	RNA Gene	43.69521	73
HPS6	HPS6 Bioge	Protein Co	25.51141	73
FBXO5	F-Box Prot	Protein Co	25.13215	73
RHOJ	Ras Homol	Protein Co	21.79995	73
AIF1	Allograft Ir	Protein Co	21.10657	73
STOML2	Stomatin L	Protein Co	14.76122	73
CXCL6	C-X-C Moti	Protein Co	14.74577	73
DDIT4	DNA Dama	Protein Co	14.1523	73
SPRY1	Sprouty RT	Protein Co	14.08802	73
H4C1	H4 Cluster	Protein Co	13.70661	73
ILF2	Interleukin	Protein Co	13.70661	73
MRPS27	Mitochonc	Protein Co	13.70661	73
PTP4A3	Protein Tyr	Protein Co	13.43013	73
KLRD1	Killer Cell L	Protein Co	13.1921	73
GCN1	GCN1 Activ	Protein Co	13.0266	73
GABRG1	Gamma-Ar	Protein Co	12.85376	73
PPP1R21	Protein Ph	Protein Co	12.79881	73
BCAR3	BCAR3 Ada	Protein Co	12.7882	73
TSPAN1	Tetraspani	Protein Co	12.7882	73
CD83	CD83 Mole	Protein Co	12.77246	73
VEGFD	Vascular Er	Protein Co	12.76136	73
EBI3	Epstein-Ba	Protein Co	12.74674	73
IFITM3	Interferon	Protein Co	12.74674	73
KIR2DL1	Killer Cell I	Protein Co	12.7266	73
MMP24	Matrix Me	Protein Co	12.7266	73
PEMT	Phosphatic	Protein Co	12.7266	73
PENK	Proenkeph	Protein Co	12.7266	73
SPARCL1	SPARC Like	Protein Co	12.7266	73
MSI2	Musashi RI	Protein Co	12.69709	73
CDC42EP1	CDC42 Effe	Protein Co	12.64969	73
CHGA	Chromogra	Protein Co	12.64969	73
NMI	N-Myc Anc	Protein Co	12.64969	73
SPRY4	Sprouty RT	Protein Co	12.56109	73
ATG2B	Autophagy	Protein Co	12.3362	73
AZU1	Azurocidin	Protein Co	12.3362	73
CIRBP	Cold Induc	Protein Co	12.3362	73
COPE	Coat Prote	Protein Co	12.3362	73
CPSF2	Cleavage A	Protein Co	12.3362	73
DDX46	DEAD-Box	Protein Co	12.3362	73
DPY30	Dpy-30 His	Protein Co	12.3362	73
ECI2	Enoyl-CoA	Protein Co	12.3362	73
H2AC4	H2A Cluste	Protein Co	12.3362	73
H2BC3	H2B Cluste	Protein Co	12.3362	73
IPO4	Importin 4	Protein Co	12.3362	73
NMU	Neuromed	Protein Co	12.3362	73
PKP3	Plakophilin	Protein Co	12.3362	73
RALY	RALY Heter	Protein Co	12.3362	73
RPL30	Ribosomal	Protein Co	12.3362	73

RSL1D1	Ribosomal	Protein Co	12.3362	73
S100A7	S100 Calciu	Protein Co	12.3362	73
SNRPC	Small Nucl	Protein Co	12.3362	73
SRPRB	SRP Recep	Protein Co	12.3362	73
STAU1	Staufen Dc	Protein Co	12.3362	73
TAGLN	Transgelin	Protein Co	12.3362	73
USP36	Ubiquitin S	Protein Co	12.3362	73
SHOX	SHOX Hom	Protein Co	10.61539	73
ZNF407	Zinc Finger	Protein Co	10.61539	73
CDH24	Cadherin 2	Protein Co	9.228029	73
AGPAT1	1-Acylglyce	Protein Co	9.212296	73
EIF4ENIF1	Eukaryotic	Protein Co	9.212296	73
HELZ	Helicase W	Protein Co	9.212296	73
MRPS34	Mitochonc	Protein Co	9.212296	73
RNF17	Ring Finger	Protein Co	9.212296	73
ZC3H11A	Zinc Finger	Protein Co	9.212296	73
ZNF598	Zinc Finger	Protein Co	9.212296	73
CPM	Carboxype	Protein Co	9.136921	73
ATF7	Activating	Protein Co	8.080542	73
DNAAF19	Dynein Axc	Protein Co	8.080542	73
FOXA3	Forkhead E	Protein Co	8.080542	73
IGFBP6	Insulin Like	Protein Co	8.080542	73
IP6K2	Inositol He	Protein Co	8.080542	73
MKRN1	Makorin Ri	Protein Co	8.080542	73
TEX11	Testis Expr	Protein Co	8.080542	73
ZP2	Zona Pellu	Protein Co	7.98948	73
KCNQ1OT1	KCNQ1 Op	RNA Gene	6.927439	73
CLDN7	Claudin 7	Protein Co	5.644465	73
CENPS	Centromer	Protein Co	5.283573	73
LASP1	LIM And Sh	Protein Co	2.036903	73
HGFAC	HGF Activa	Protein Co	1.723406	73
HLX	H2.0 Like H	Protein Co	1.723406	73
MIR145	MicroRNA	RNA Gene	177.8634	72
SHBG	Sex Hormo	Protein Co	46.23233	72
FCRL3	Fc Recepto	Protein Co	39.5095	72
KISS1	KiSS-1 Met	Protein Co	39.43723	72
HOXA5	Homeobox	Protein Co	20.82728	72
PHF19	PHD Finger	Protein Co	20.80714	72
PTMA	Prothymos	Protein Co	17.29284	72
SKAP1	Src Kinase	Protein Co	17.24697	72
RSPO3	R-Spondin	Protein Co	17.21746	72
TMOD3	Tropomod	Protein Co	14.49586	72
DUSP2	Dual Speci	Protein Co	14.46651	72
MSI1	Musashi RI	Protein Co	14.38085	72
SFRP2	Secreted F	Protein Co	14.21886	72
TMSB4X	Thymosin I	Protein Co	13.70661	72
CD82	CD82 Mole	Protein Co	13.46932	72
CCL17	C-C Motif (	Protein Co	13.4657	72
SRA1	Steroid Re	Protein Co	13.1921	72
MOK	MOK Prote	Protein Co	12.79881	72

CD1A	CD1a Mole	Protein Co	12.77246	72
INHBB	Inhibin Sub	Protein Co	12.77246	72
MUC17	Mucin 17,	Protein Co	12.76136	72
MIR1-1	MicroRNA	RNA Gene	12.74674	72
TRERF1	Transcripti	Protein Co	12.74674	72
TRIB1	Tribbles Ps	Protein Co	12.74674	72
HOOK1	Hook Micr	Protein Co	12.7266	72
NFIL3	Nuclear Fa	Protein Co	12.7266	72
PLPP3	Phospholip	Protein Co	12.7266	72
CNTF	Ciliary Neu	Protein Co	12.69709	72
KLRC2	Killer Cell L	Protein Co	12.69709	72
PPP1R9B	Protein Ph	Protein Co	12.64969	72
BYSL	Bystin Like	Protein Co	12.3362	72
DDX18	DEAD-Box	Protein Co	12.3362	72
DDX47	DEAD-Box	Protein Co	12.3362	72
EIF3L	Eukaryotic	Protein Co	12.3362	72
HES5	Hes Family	Protein Co	12.3362	72
MTDH	Metadheri	Protein Co	12.3362	72
MTX1	Metaxin 1	Protein Co	12.3362	72
PTRH2	Peptidyl-TI	Protein Co	12.3362	72
RAB21	RAB21, Me	Protein Co	12.3362	72
RASAL1	RAS Protei	Protein Co	12.3362	72
SRP68	Signal Recc	Protein Co	12.3362	72
SURF4	Surfeit 4	Protein Co	12.3362	72
TRMT112	TRNA Metl	Protein Co	12.3362	72
YAF2	YY1 Associ	Protein Co	12.3362	72
PRDX4	Peroxiredc	Protein Co	10.61279	72
EIF4E2	Eukaryotic	Protein Co	9.212296	72
GTF3C2	General Tr	Protein Co	9.212296	72
PECR	Peroxisom	Protein Co	9.212296	72
PRPS2	Phosphorib	Protein Co	9.212296	72
RBM25	RNA Bindir	Protein Co	9.212296	72
SERPINB3	Serpin Fam	Protein Co	9.212296	72
ARFIP2	ARF Intera	Protein Co	9.16643	72
CLEC10A	C-Type Lec	Protein Co	9.16643	72
CSTA	Cystatin A	Protein Co	9.136921	72
NMNAT3	Nicotinami	Protein Co	8.77603	72
ATP6V1G2	ATPase H+	Protein Co	8.080542	72
BNIP1	BCL2 Intera	Protein Co	8.080542	72
CCN4	Cellular Co	Protein Co	8.080542	72
CKAP2L	Cytoskelet	Protein Co	8.080542	72
KCNG2	Potassium	Protein Co	8.080542	72
KIF18B	Kinesin Far	Protein Co	8.080542	72
KLHL9	Kelch Like	Protein Co	8.080542	72
MACROD2	Mono-ADF	Protein Co	8.080542	72
MFHAS1	Multifunct	Protein Co	8.080542	72
NEGR1	Neuronal C	Protein Co	8.080542	72
NTSR2	Neurotens	Protein Co	8.080542	72
RMND1	Required F	Protein Co	8.080542	72
INSL3	Insulin Like	Protein Co	7.98948	72

PACRG	Parkin Cor	Protein Co	7.98948	72
SEPTIN12	Septin 12	Protein Co	7.98948	72
TEX15	Testis Expr	Protein Co	7.98948	72
USP26	Ubiquitin S	Protein Co	7.98948	72
CXCL16	C-X-C Moti	Protein Co	6.174243	72
IL22	Interleukin	Protein Co	6.139478	72
TFPI2	Tissue Fact	Protein Co	5.708739	72
CDX1	Caudal Typ	Protein Co	5.597071	72
SELENBP1	Selenium E	Protein Co	5.508469	72
TRIM65	Tripartite M	Protein Co	5.283573	72
GPR18	G Protein-(	Protein Co	1.948301	72
EPC1	Enhancer C	Protein Co	1.723406	72
MIR29A	MicroRNA	RNA Gene	68.73734	71
FBXW2	F-Box And	Protein Co	20.86172	71
DEFA1	Defensin A	Protein Co	20.853	71
MIR29B1	MicroRNA	RNA Gene	20.78644	71
RHOU	Ras Homol	Protein Co	20.77763	71
PGRMC2	Progesterc	Protein Co	20.16541	71
BST2	Bone Marr	Protein Co	14.94919	71
CLDN4	Claudin 4	Protein Co	14.75795	71
KLF9	KLF Transc	Protein Co	14.74884	71
ZFP36	ZFP36 Zinc	Protein Co	14.56539	71
METTL14	Methyltrar	Protein Co	14.45371	71
CCNL2	Cyclin L2	Protein Co	14.35556	71
SLPI	Secretory L	Protein Co	14.18975	71
LYVE1	Lymphatic	Protein Co	14.08802	71
PFDN5	Prefoldin S	Protein Co	13.72501	71
SERBP1	SERPINE1 I	Protein Co	13.70661	71
GTF3C5	General Tr	Protein Co	13.62252	71
GABRQ	Gamma-Ar	Protein Co	12.85376	71
PZP	PZP Alpha-	Protein Co	12.85376	71
IL9	Interleukin	Protein Co	12.78118	71
GAS2L2	Growth Ar	Protein Co	12.77246	71
SPN	Sialophorir	Protein Co	12.77246	71
RNH1	Ribonuclea	Protein Co	12.76136	71
CHCHD2	Coiled-Coil	Protein Co	12.7266	71
ERRFI1	ERBB Rece	Protein Co	12.7266	71
FSTL3	Follistatin I	Protein Co	12.7266	71
ZNF473	Zinc Finger	Protein Co	12.7266	71
RECK	Reversion	Protein Co	12.64969	71
DNAJA2	DnaJ Heat	Protein Co	12.3362	71
GLG1	Golgi Glycc	Protein Co	12.3362	71
HSPE1	Heat Shock	Protein Co	12.3362	71
SART1	Spliceosom	Protein Co	12.3362	71
SF3B6	Splicing Fa	Protein Co	12.3362	71
SGF29	SAGA Com	Protein Co	12.3362	71
ATXN2L	Ataxin 2 Lil	Protein Co	9.212296	71
CNTNAP4	Contactin /	Protein Co	9.212296	71
HOXD3	Homeobox	Protein Co	9.186571	71
S100A11	S100 Calciu	Protein Co	9.16643	71

NAV3	Neuron Na	Protein Co	9.089527	71
ADGRA2	Adhesion (	Protein Co	8.080542	71
AGBL4	AGBL Carb	Protein Co	8.080542	71
COX6C	Cytochrom	Protein Co	8.080542	71
CSAD	Cysteine Su	Protein Co	8.080542	71
DNAJC17	DnaJ Heat	Protein Co	8.080542	71
KCNU1	Potassium	Protein Co	8.080542	71
PITPNM2	Phosphatic	Protein Co	8.080542	71
RABGAP1L	RAB GTPas	Protein Co	8.080542	71
SLC20A1	Solute Carı	Protein Co	8.080542	71
ZNF263	Zinc Finger	Protein Co	8.080542	71
DAZL	Deleted In	Protein Co	7.98948	71
MYBL1	MYB Proto	Protein Co	7.98948	71
REC8	REC8 Meio	Protein Co	7.98948	71
SYCP3	Synaptone	Protein Co	7.98948	71
CAPN7	Calpain 7	Protein Co	6.139478	71
ADIPOR2	Adiponecti	Protein Co	5.673974	71
KIR3DL1	Killer Cell I	Protein Co	5.283573	71
PDPN	Podoplanir	Protein Co	1.948301	71
SENP3	SUMO Spe	Protein Co	1.723406	71
ID4	Inhibitor O	Protein Co	20.86874	70
BMF	Bcl2 Modif	Protein Co	20.853	70
MIR16-1	MicroRNA	RNA Gene	19.87657	70
HOXC4	Homeobox	Protein Co	17.26711	70
CKAP4	Cytoskelet	Protein Co	15.03564	70
APOBEC3B	Apolipopro	Protein Co	14.84698	70
IL27	Interleukin	Protein Co	14.76122	70
TCF21	Transcripti	Protein Co	14.39631	70
NAPEPLD	N-Acyl Pho	Protein Co	14.18975	70
PSMD10	Proteasom	Protein Co	14.17913	70
IGHM	Immunoglo	Protein Co	13.6979	70
GHRH	Growth Ho	Protein Co	13.23797	70
NTS	Neurotens	Protein Co	12.85376	70
MAP7	Microtubu	Protein Co	12.79398	70
RNF144B	Ring Finger	Protein Co	12.7882	70
ICAM4	Intercellula	Protein Co	12.77246	70
IGSF8	Immunoglo	Protein Co	12.77246	70
RAP2A	RAP2A, Me	Protein Co	12.77246	70
RSAD2	Radical S-A	Protein Co	12.76136	70
SHOX2	SHOX Hom	Protein Co	12.76136	70
CCL16	C-C Motif (	Protein Co	12.74674	70
PRELP	Proline And	Protein Co	12.74674	70
RNASE1	Ribonuclea	Protein Co	12.7266	70
LOXL1	Lysyl Oxida	Protein Co	12.69709	70
CD24	CD24 Mole	Protein Co	12.64969	70
CPEB3	Cytoplasm	Protein Co	12.64969	70
AHSA1	Activator (	Protein Co	12.3362	70
ATP5PD	ATP Syntha	Protein Co	12.3362	70
BCAS2	BCAS2 Pre-	Protein Co	12.3362	70
CCDC47	Coiled-Coil	Protein Co	12.3362	70

CCL28	C-C Motif (	Protein Co	12.3362	70
ECPAS	Ecm29 Pro	Protein Co	12.3362	70
EDC4	Enhancer (	Protein Co	12.3362	70
HDGFL2	HDGF Like	Protein Co	12.3362	70
KLF15	KLF Transc	Protein Co	12.3362	70
PCID2	PCI Domai	Protein Co	12.3362	70
POLDIP2	DNA Polym	Protein Co	12.3362	70
SLC30A1	Solute Car	Protein Co	12.3362	70
TSR1	TSR1 Ribos	Protein Co	12.3362	70
VTA1	Vesicle Tra	Protein Co	12.3362	70
DECR2	2,4-Dienoy	Protein Co	9.212296	70
DSC1	Desmocoll	Protein Co	9.212296	70
GTF3C3	General Tr	Protein Co	9.212296	70
CCL13	C-C Motif (	Protein Co	9.16643	70
PTX3	Pentraxin 3	Protein Co	9.16643	70
ATP5MC2	ATP Syntha	Protein Co	8.080542	70
CALCOCO1	Calcium Bi	Protein Co	8.080542	70
DENND1B	DENN Dom	Protein Co	8.080542	70
ZWILCH	Zwilch Kine	Protein Co	8.080542	70
ACR	Acrosin	Protein Co	7.98948	70
MOV10L1	Mov10 Like	Protein Co	7.98948	70
PIWIL2	Piwi Like R	Protein Co	7.98948	70
SPO11	SPO11 Initi	Protein Co	7.98948	70
IMP3	IMP U3 Sm	Protein Co	5.694116	70
CLDN11	Claudin 11	Protein Co	5.644465	70
KAT14	Lysine Ace	Protein Co	5.597071	70
PNOC	Prepronoc	Protein Co	3.560168	70
GPC2	Glypican 2	Protein Co	1.723406	70
NUDT15	Nudix Hyd	Protein Co	1.723406	70
MIR199A1	MicroRNA	RNA Gene	68.4838	69
CALB2	Calbindin 2	Protein Co	45.01592	69
IL32	Interleukin	Protein Co	22.83849	69
BMP15	Bone Morp	Protein Co	22.18995	69
MIR34A	MicroRNA	RNA Gene	21.8526	69
HOXC8	Homeobox	Protein Co	21.27264	69
RERG	RAS Like Es	Protein Co	20.64163	69
HOXA7	Homeobox	Protein Co	18.69593	69
RNLS	Renalase, F	Protein Co	18.26042	69
RETN	Resistin	Protein Co	14.75605	69
CADM2	Cell Adhesi	Protein Co	14.21427	69
ISG20	Interferon	Protein Co	13.71363	69
SFXN1	Sideroflexi	Protein Co	13.70661	69
LOXL4	Lysyl Oxida	Protein Co	13.62252	69
HOXC10	Homeobox	Protein Co	13.36412	69
CLDN3	Claudin 3	Protein Co	13.28012	69
TFF1	Trefoil Fact	Protein Co	13.21224	69
APOBEC3A	Apolipopro	Protein Co	13.1921	69
MIR15B	MicroRNA	RNA Gene	12.80954	69
HAS3	Hyalurona	Protein Co	12.7882	69
CAB39	Calcium Bi	Protein Co	12.74674	69

HSD17B6	Hydroxyste	Protein Co	12.7266	69
EPX	Eosinophil	Protein Co	12.69709	69
IL24	Interleukin	Protein Co	12.69709	69
CCL1	C-C Motif (	Protein Co	12.64969	69
TNFAIP6	TNF Alpha	Protein Co	12.64969	69
ACOT9	Acyl-CoA T	Protein Co	12.3362	69
ERGIC1	Endoplasm	Protein Co	12.3362	69
GIGYF1	GRB10 Inte	Protein Co	12.3362	69
HMGB2	High Mobil	Protein Co	12.3362	69
ITIH2	Inter-Alpha	Protein Co	12.3362	69
MRPS23	Mitochond	Protein Co	12.3362	69
PDIA4	Protein Dis	Protein Co	12.3362	69
PSPC1	Paraspeckl	Protein Co	12.3362	69
RETSAT	Retinol Sat	Protein Co	12.3362	69
RRP9	Ribosomal	Protein Co	12.3362	69
SERPINA4	Serpin Fam	Protein Co	12.3362	69
SLIRP	SRA Stem-I	Protein Co	12.3362	69
VAT1	Vesicle Am	Protein Co	12.3362	69
ZGPAT	Zinc Finger	Protein Co	12.3362	69
LRP10	LDL Recept	Protein Co	10.61539	69
SEC14L1	SEC14 Like	Protein Co	10.61279	69
PRAME	PRAME Nu	Protein Co	9.228029	69
AKR7A2	Aldo-Keto	Protein Co	9.212296	69
ATG16L2	Autophagy	Protein Co	9.212296	69
H2AC20	H2A Cluste	Protein Co	9.212296	69
HACD3	3-Hydroxya	Protein Co	9.212296	69
ACTR1B	Actin Relat	Protein Co	8.080542	69
ARHGAP42	Rho GTPas	Protein Co	8.080542	69
DDX31	DEAD-Box	Protein Co	8.080542	69
FGD6	FYVE, RhoG	Protein Co	8.080542	69
HOXC13	Homeobox	Protein Co	8.080542	69
MCHR2	Melanin Co	Protein Co	8.080542	69
SLC9A2	Solute Carr	Protein Co	8.080542	69
TIPIN	TIMELESS I	Protein Co	8.080542	69
GJA4	Gap Juncti	Protein Co	7.98948	69
TLE6	TLE Family	Protein Co	7.98948	69
CRHBP	Corticotrop	Protein Co	5.708739	69
LAP3	Leucine An	Protein Co	3.560168	69
BCCIP	BRCA2 Anc	Protein Co	2.133948	69
CCDC6	Coiled-Coil	Protein Co	1.723406	69
DLX4	Distal-Less	Protein Co	1.723406	69
SP4	Sp4 Transc	Protein Co	1.723406	69
MIR126	MicroRNA	RNA Gene	177.7527	68
MIR200B	MicroRNA	RNA Gene	157.622	68
MIR29C	MicroRNA	RNA Gene	157.5815	68
MIR30B	MicroRNA	RNA Gene	54.18567	68
MIR9-1	MicroRNA	RNA Gene	26.89125	68
SRP14	Signal Reco	Protein Co	20.853	68
MIR140	MicroRNA	RNA Gene	19.81378	68
SGPP1	Sphingosin	Protein Co	15.47002	68

SNCG	Synuclein (	Protein Co	14.86799	68
MIR27B	MicroRNA	RNA Gene	14.75967	68
RASSF2	Ras Associa	Protein Co	14.51722	68
YTHDF2	YTH N6-Me	Protein Co	14.38392	68
HAS2	Hyaluronar	Protein Co	14.20768	68
S100P	S100 Calciu	Protein Co	13.67217	68
CD200R1	CD200 Rec	Protein Co	13.62252	68
CCL8	C-C Motif (	Protein Co	13.28012	68
LST1	Leukocyte	Protein Co	12.85376	68
MIR26A1	MicroRNA	RNA Gene	12.80954	68
CCL18	C-C Motif (	Protein Co	12.78118	68
CRISPLD2	Cysteine Ri	Protein Co	12.77246	68
HOXB4	Homeobox	Protein Co	12.77246	68
KIF25	Kinesin Far	Protein Co	12.77246	68
ZNF24	Zinc Finger	Protein Co	12.76136	68
BTG1	BTG Anti-P	Protein Co	12.74674	68
FOXD3	Forkhead E	Protein Co	12.74674	68
IL34	Interleukin	Protein Co	12.74674	68
OLFM1	Olfactome	Protein Co	12.7266	68
TRB	T Cell Rece	Protein Co	12.7266	68
DNAJC11	DnaJ Heat	Protein Co	12.3362	68
FRG1	FSHD Regio	Protein Co	12.3362	68
HNRNPA0	Heterogen	Protein Co	12.3362	68
LSM5	LSM5 Hom	Protein Co	12.3362	68
MRPS9	Mitochond	Protein Co	12.3362	68
MYL12A	Myosin Lig	Protein Co	12.3362	68
ORMDL3	ORMDL Sp	Protein Co	12.3362	68
RER1	Retention	Protein Co	12.3362	68
RPL34	Ribosomal	Protein Co	12.3362	68
RTCB	RNA 2',3'-C	Protein Co	12.3362	68
MEPE	Matrix Extr	Protein Co	10.61539	68
HECTD4	HECT Dom	Protein Co	10.61279	68
EHD4	EH Domain	Protein Co	9.228029	68
GCNT1	Glucosami	Protein Co	9.228029	68
DNAJB4	DnaJ Heat	Protein Co	9.212296	68
HECTD3	HECT Dom	Protein Co	9.212296	68
HRNR	Hornerin	Protein Co	9.212296	68
RCN2	Reticulocal	Protein Co	9.212296	68
TRA2A	Transform	Protein Co	9.212296	68
POLR2M	RNA Polym	Protein Co	9.16643	68
ASPN	Asporin	Protein Co	9.136921	68
LAMB4	Laminin Su	Protein Co	9.089527	68
CLIC4	Chloride In	Protein Co	8.080542	68
CYP2W1	Cytochrom	Protein Co	8.080542	68
DIPK1A	Divergent I	Protein Co	8.080542	68
DNAJC1	DnaJ Heat	Protein Co	8.080542	68
HOXC11	Homeobox	Protein Co	8.080542	68
HOXC9	Homeobox	Protein Co	8.080542	68
IL36G	Interleukin	Protein Co	8.080542	68
DPY19L2	Dpy-19 Lik	Protein Co	7.98948	68

CLDN2	Claudin 2	Protein Co	6.194056	68
VTCN1	V-Set Dom	Protein Co	1.723406	68
MIR20A	MicroRNA	RNA Gene	157.5906	67
MIR143	MicroRNA	RNA Gene	156.8087	67
GREB1	Growth Re	Protein Co	87.47234	67
MIR125B1	MicroRNA	RNA Gene	66.73515	67
MIR200C	MicroRNA	RNA Gene	58.04862	67
JAZF1	JAZF Zinc F	Protein Co	43.03012	67
RARRES2	Retinoic Ac	Protein Co	38.18996	67
MEG3	Maternally	RNA Gene	37.74719	67
S100A13	S100 Calciu	Protein Co	35.91045	67
MIR130A	MicroRNA	RNA Gene	24.58673	67
WFDC2	WAP Four-	Protein Co	23.60377	67
MIR210	MicroRNA	RNA Gene	21.99889	67
MIR23A	MicroRNA	RNA Gene	21.83325	67
MIR138-1	MicroRNA	RNA Gene	16.23347	67
KRT20	Keratin 20	Protein Co	15.03918	67
CLMP	CXADR Like	Protein Co	14.57047	67
TFF3	Trefoil Fact	Protein Co	14.20047	67
CHTOP	Chromatin	Protein Co	13.70661	67
ITGBL1	Integrin Su	Protein Co	13.6979	67
CYRIB	CYFIP Relat	Protein Co	12.78118	67
GNLY	Granulysin	Protein Co	12.77246	67
ELL2	Elongation	Protein Co	12.7266	67
IL17RE	Interleukin	Protein Co	12.7266	67
IL25	Interleukin	Protein Co	12.7266	67
MIR34B	MicroRNA	RNA Gene	12.7266	67
PROKR1	Prokinetici	Protein Co	12.7266	67
OGN	Osteoglycin	Protein Co	12.64969	67
BUD31	BUD31 Spl	Protein Co	12.3362	67
DNAJC9	DnaJ Heat	Protein Co	12.3362	67
INTS3	Integrator	Protein Co	12.3362	67
LYAR	Ly1 Antibo	Protein Co	12.3362	67
MFAP4	Microfibril	Protein Co	12.3362	67
RTRAF	RNA Transc	Protein Co	12.3362	67
SUB1	SUB1 Regu	Protein Co	12.3362	67
UBAP2	Ubiquitin A	Protein Co	12.3362	67
BLOC1S5	Biogenesis	Protein Co	10.61539	67
HSPA4L	Heat Shock	Protein Co	9.212296	67
HSPBP1	HSPA (Hsp7	Protein Co	9.212296	67
MRPL4	Mitochond	Protein Co	9.212296	67
NIPSNAP2	Nipsnap Ho	Protein Co	9.212296	67
PCDH8	Protocadhe	Protein Co	9.186571	67
OSR1	Odd-Skippe	Protein Co	8.77603	67
CDK2AP1	Cyclin Dep	Protein Co	8.080542	67
HOXA4	Homeobox	Protein Co	8.080542	67
JADE2	Jade Family	Protein Co	8.080542	67
KLF3	KLF Transc	Protein Co	8.080542	67
LSM11	LSM11, U7	Protein Co	8.080542	67
PRAG1	PEAK1 Rela	Protein Co	8.080542	67

PSMD5	Proteasom	Protein Co	8.080542	67
ZSCAN10	Zinc Finger	Protein Co	8.080542	67
MIR133A1	MicroRNA	RNA Gene	7.067037	67
MED9	Mediator (	Protein Co	5.283573	67
IBSP	Integrin Bi	Protein Co	3.560168	67
MIR223	MicroRNA	RNA Gene	181.1261	66
H19	H19 Imprir	RNA Gene	179.7579	66
PAEP	Progestage	Protein Co	70.95918	66
VEZT	Vezatin, Ac	Protein Co	50.45379	66
MIR17	MicroRNA	RNA Gene	43.88675	66
EBAG9	Estrogen R	Protein Co	42.86874	66
MIRLET7B	MicroRNA	RNA Gene	41.35786	66
MIR221	MicroRNA	RNA Gene	39.45234	66
MIR33B	MicroRNA	RNA Gene	28.01108	66
SLTM	SAFB Like T	Protein Co	27.78245	66
MIR181B1	MicroRNA	RNA Gene	24.37561	66
MIR146A	MicroRNA	RNA Gene	21.9264	66
MIR214	MicroRNA	RNA Gene	21.26751	66
ZBTB40	Zinc Finger	Protein Co	20.86874	66
MIR101-1	MicroRNA	RNA Gene	19.89037	66
MIR185	MicroRNA	RNA Gene	19.8395	66
MIR222	MicroRNA	RNA Gene	19.8395	66
MIR27A	MicroRNA	RNA Gene	19.8284	66
MIR15A	MicroRNA	RNA Gene	19.79363	66
MIR26B	MicroRNA	RNA Gene	14.76984	66
ALKBH5	AlkB Homo	Protein Co	14.76264	66
KLF12	KLF Transc	Protein Co	14.7275	66
UCN	Urocortin	Protein Co	14.55038	66
LRRC59	Leucine Ric	Protein Co	13.70661	66
YTHDF1	YTH N6-Me	Protein Co	13.0266	66
PCDH10	Protocadh	Protein Co	12.77246	66
HSPA7	Heat Shock	Pseudogen	12.76136	66
UGT2B28	UDP Glucu	Protein Co	12.76136	66
LEFTY1	Left-Right I	Protein Co	12.74674	66
MT1E	Metallothi	Protein Co	12.7266	66
API5	Apoptosis	Protein Co	12.3362	66
DDX27	DEAD-Box	Protein Co	12.3362	66
DNAJC8	DnaJ Heat	Protein Co	12.3362	66
MRPL46	Mitochond	Protein Co	12.3362	66
NOL11	Nucleolar I	Protein Co	12.3362	66
PDE12	Phosphodi	Protein Co	12.3362	66
PIN4	Peptidylpr	Protein Co	12.3362	66
PYCR3	Pyrroline-5	Protein Co	12.3362	66
SCAMP3	Secretory (	Protein Co	12.3362	66
TTLL12	Tubulin Ty	Protein Co	12.3362	66
UFL1	UFM1 Spec	Protein Co	12.3362	66
ZNF622	Zinc Finger	Protein Co	12.3362	66
ANXA2P2	Annexin A2	Pseudogen	9.212296	66
DCD	Dermcidin	Protein Co	9.212296	66
PDCL	Phosducin	Protein Co	9.212296	66

FAM3C	FAM3 Met	Protein Co	9.16643	66
ARL4C	ARF Like G	Protein Co	8.77603	66
AAGAB	Alpha And	Protein Co	8.080542	66
CHST8	Carbohydr	Protein Co	8.080542	66
IP6K1	Inositol He	Protein Co	8.080542	66
KCTD15	Potassium	Protein Co	8.080542	66
MRPS14	Mitochond	Protein Co	8.080542	66
RABL2A	RAB, Mem	Protein Co	8.080542	66
SPECC1	Sperm Ant	Protein Co	8.080542	66
XPOT	Exportin Fo	Protein Co	8.080542	66
YIPF5	Yip1 Doma	Protein Co	8.080542	66
ZBTB2	Zinc Finger	Protein Co	8.080542	66
SPAG6	Sperm Ass	Protein Co	7.98948	66
SYCP1	Synaptone	Protein Co	7.98948	66
TBPL1	TATA-Box I	Protein Co	7.98948	66
MIR30C1	MicroRNA	RNA Gene	7.067037	66
MIR590	MicroRNA	RNA Gene	7.067037	66
FOXO6	Forkhead E	Protein Co	6.927439	66
STC2	Stanniocal	Protein Co	6.927439	66
FBXO45	F-Box Prot	Protein Co	5.283573	66
HOXD9	Homeobox	Protein Co	5.283573	66
PRR12	Proline Ric	Protein Co	5.283573	66
DEFB1	Defensin B	Protein Co	1.723406	66
MIR34C	MicroRNA	RNA Gene	182.6494	65
NEAT1	Nuclear Pa	RNA Gene	149.3468	65
MIR98	MicroRNA	RNA Gene	39.36866	65
MIR148A	MicroRNA	RNA Gene	30.55248	65
MIR182	MicroRNA	RNA Gene	22.12557	65
MIR133B	MicroRNA	RNA Gene	22.03756	65
HOXC6	Homeobox	Protein Co	20.8419	65
MIR31	MicroRNA	RNA Gene	20.8047	65
MIR106B	MicroRNA	RNA Gene	19.8395	65
MIR149	MicroRNA	RNA Gene	19.81378	65
MIR30D	MicroRNA	RNA Gene	19.65404	65
CGB3	Chorionic (	Protein Co	12.85376	65
ULBP2	UL16 Bindi	Protein Co	12.80293	65
SERPINA12	Serpin Fam	Protein Co	12.7882	65
SORCS1	Sortilin Rel	Protein Co	12.7882	65
PCDH17	Protocadh	Protein Co	12.76136	65
CPA3	Carboxype	Protein Co	12.7266	65
MEST	Mesoderm	Protein Co	12.7266	65
MIR195	MicroRNA	RNA Gene	12.66411	65
MIR375	MicroRNA	RNA Gene	12.64969	65
MYCBP	MYC Bindi	Protein Co	12.56109	65
AGMAT	Agmatinas	Protein Co	12.3362	65
ARF4-AS1	ARF4 Antis	RNA Gene	12.3362	65
CENPV	Centromer	Protein Co	12.3362	65
DEFB4A	Defensin B	Protein Co	12.3362	65
FUBP3	Far Upstre	Protein Co	12.3362	65
MANF	Mesencepl	Protein Co	12.3362	65

METTL16	Methyltrar	Protein Co	12.3362	65
PDXDC1	Pyridoxal D	Protein Co	12.3362	65
PRPSAP1	Phosphorib	Protein Co	12.3362	65
SNRPGP15	Small Nucl	Pseudogen	12.3362	65
TMED9	Transmem	Protein Co	12.3362	65
ZNF384	Zinc Finger	Protein Co	12.3362	65
NGF-AS1	NGF Antise	RNA Gene	9.803947	65
KAZN	Kazrin, Per	Protein Co	8.77603	65
SIPA1L2	Signal Indu	Protein Co	8.77603	65
AEN	Apoptosis	Protein Co	8.080542	65
C12orf43	Chromosoi	Protein Co	8.080542	65
CDH20	Cadherin 2	Protein Co	8.080542	65
DM1-AS	DM1 Locus	RNA Gene	8.080542	65
DMWD	DM1 Locus	Protein Co	8.080542	65
KLHDC8B	Kelch Dom	Protein Co	8.080542	65
LINC02456	Long Interg	RNA Gene	8.080542	65
SKAP2	Src Kinase	Protein Co	8.080542	65
TSKU	Tsukushi, S	Protein Co	8.080542	65
PRM2	Protamine	Protein Co	7.98948	65
YBX2	Y-Box Bind	Protein Co	7.98948	65
MIR204	MicroRNA	RNA Gene	7.067037	65
TRO	Trophinin	Protein Co	1.723406	65
MIR142	MicroRNA	RNA Gene	157.3226	64
MIR150	MicroRNA	RNA Gene	156.853	64
MIRLET7G	MicroRNA	RNA Gene	36.61099	64
IPO13	Importin 1	Protein Co	31.26494	64
NFE2L3	NFE2 Like I	Protein Co	23.04641	64
MIR24-1	MicroRNA	RNA Gene	21.2826	64
MIR10A	MicroRNA	RNA Gene	19.87657	64
MIR93	MicroRNA	RNA Gene	16.23347	64
GNRHR2	Gonadotro	Pseudogen	15.96771	64
KLF16	KLF Transc	Protein Co	14.1634	64
MIR103A1	MicroRNA	RNA Gene	12.80954	64
DEFA3	Defensin A	Protein Co	12.77246	64
NUCKS1	Nuclear Ca	Protein Co	12.77246	64
TRIM59	Tripartite M	Protein Co	12.76136	64
ILKAP	ILK Associa	Protein Co	12.74674	64
CAPN6	Calpain 6	Protein Co	12.7266	64
GREM2	Gremlin 2,	Protein Co	12.7266	64
MT1G	Metallothi	Protein Co	12.7266	64
MT1X	Metallothi	Protein Co	12.7266	64
RGCC	Regulator (	Protein Co	12.7266	64
SOX15	SRY-Box Tr	Protein Co	12.64969	64
FKBP3	FKBP Proly	Protein Co	12.3362	64
GLTP	Glycolipid	Protein Co	12.3362	64
GNL1	G Protein N	Protein Co	12.3362	64
HNRNPUL2	Heterogen	Protein Co	12.3362	64
PDIA5	Protein Dis	Protein Co	12.3362	64
AAR2	AAR2 Splic	Protein Co	9.212296	64
POM121C	POM121 T	Protein Co	9.212296	64

HOXD1	Homeobox	Protein Co	9.186571	64
GIMAP5	GTPase, IM	Protein Co	8.77603	64
HOXB2	Homeobox	Protein Co	8.77603	64
CDCA2	Cell Divisio	Protein Co	8.080542	64
NPFF	Neuropept	Protein Co	8.080542	64
TMTC1	Transmem	Protein Co	8.080542	64
TOR2A	Torsin Fam	Protein Co	3.560168	64
FNDC5	Fibronectir	Protein Co	1.723406	64
MIR125A	MicroRNA	RNA Gene	183.4394	63
MIR141	MicroRNA	RNA Gene	178.9072	63
MIR424	MicroRNA	RNA Gene	157.8254	63
MIR200A	MicroRNA	RNA Gene	156.8087	63
MIR483	MicroRNA	RNA Gene	47.3545	63
MIRLET7A1	MicroRNA	RNA Gene	43.6634	63
MIR320A	MicroRNA	RNA Gene	41.96474	63
MIR199B	MicroRNA	RNA Gene	38.65807	63
MIR19B1	MicroRNA	RNA Gene	26.89125	63
MIR92A1	MicroRNA	RNA Gene	21.26751	63
MIR342	MicroRNA	RNA Gene	20.34208	63
MIR30A	MicroRNA	RNA Gene	19.87927	63
GSTT1	Glutathion	Protein Co	15.89636	63
BDNF-AS	BDNF Antis	RNA Gene	14.33575	63
DIRAS3	DIRAS Fam	Protein Co	14.20958	63
MIR203A	MicroRNA	RNA Gene	14.17913	63
HSDL2	Hydroxyste	Protein Co	13.65203	63
PF4V1	Platelet Fa	Protein Co	12.79398	63
OR1D2	Olfactory F	Protein Co	12.7882	63
NINJ1	Ninjurin 1	Protein Co	12.78118	63
SLITRK3	SLIT And N	Protein Co	12.77246	63
MIR499A	MicroRNA	RNA Gene	12.74674	63
MT1H	Metallothi	Protein Co	12.7266	63
TIMP4	TIMP Meta	Protein Co	12.7266	63
ANKHD1	Ankyrin Re	Protein Co	12.3362	63
ERMP1	Endoplasm	Protein Co	12.3362	63
MRPS35	Mitochond	Protein Co	12.3362	63
POTEE	POTE Anky	Protein Co	12.3362	63
WDR75	WD Repeat	Protein Co	12.3362	63
IGKC	Immunoglo	Protein Co	9.212296	63
ISL2	ISL LIM Ho	Protein Co	9.186571	63
SH3GLB2	SH3 Domai	Protein Co	9.16643	63
FAM120B	Family Wit	Protein Co	8.080542	63
FSTL4	Follistatin I	Protein Co	8.080542	63
KCTD9	Potassium	Protein Co	8.080542	63
MEGF11	Multiple EG	Protein Co	8.080542	63
PMCH	Pro-Melan	Protein Co	8.080542	63
SPPL3	Signal Pept	Protein Co	8.080542	63
UTP23	UTP23 Sma	Protein Co	8.080542	63
VWA3B	Von Willeb	Protein Co	8.080542	63
ZNF438	Zinc Finger	Protein Co	8.080542	63
MIR152	MicroRNA	RNA Gene	7.067037	63

TSGA10	Testis Spec	Protein Co	6.927439	63
VASH1	Vasohibin :	Protein Co	5.597071	63
TOB1	Transducer	Protein Co	5.508469	63
MMP27	Matrix Me	Protein Co	5.283573	63
OVGP1	Oviductal (	Protein Co	5.283573	63
SPA17	Sperm Aut	Protein Co	1.723406	63
MIR100	MicroRNA	RNA Gene	157.2198	62
ZSWIM7	Zinc Finger	Protein Co	43.36673	62
MIR205	MicroRNA	RNA Gene	43.11014	62
MIRLET7C	MicroRNA	RNA Gene	41.64495	62
MIRLET7F1	MicroRNA	RNA Gene	38.0501	62
MIR10B	MicroRNA	RNA Gene	27.01191	62
MIR302A	MicroRNA	RNA Gene	22.27928	62
MIR22	MicroRNA	RNA Gene	21.93139	62
XIST	X Inactive S	RNA Gene	21.11719	62
MIR106A	MicroRNA	RNA Gene	20.34477	62
MIR503	MicroRNA	RNA Gene	20.33135	62
MIR18A	MicroRNA	RNA Gene	19.87657	62
MIR361	MicroRNA	RNA Gene	19.87657	62
MIR144	MicroRNA	RNA Gene	19.85523	62
MIR155	MicroRNA	RNA Gene	19.79363	62
HOXC5	Homeobox	Protein Co	17.26711	62
CGB5	Chorionic (	Protein Co	15.22979	62
MRGPRX2	MAS Relate	Protein Co	14.38901	62
OLFM4	Olfactome	Protein Co	13.74217	62
AFM	Afamin	Protein Co	13.1921	62
MIR16-2	MicroRNA	RNA Gene	12.80954	62
ZBTB10	Zinc Finger	Protein Co	12.79881	62
PAX8-AS1	PAX8 Antis	RNA Gene	12.77246	62
MIR135A1	MicroRNA	RNA Gene	12.76136	62
PIGF	Phosphatic	Protein Co	12.74674	62
MT1F	Metallothi	Protein Co	12.7266	62
UCN3	Urocortin :	Protein Co	12.69709	62
MIR455	MicroRNA	RNA Gene	12.57551	62
CDKN2AIP	CDKN2A In	Protein Co	12.3362	62
CRIP2	Cysteine Ri	Protein Co	12.3362	62
ERH	ERH MRNA	Protein Co	12.3362	62
FASTKD5	FAST Kinas	Protein Co	12.3362	62
HEPHL1	Hephaestir	Protein Co	12.3362	62
OCIAD1	OCIA Doma	Protein Co	12.3362	62
ZC3H15	Zinc Finger	Protein Co	12.3362	62
AMBN	Ameloblas	Protein Co	10.61539	62
ILRUN	Inflammati	Protein Co	9.228029	62
NIT2	Nitrilase Fa	Protein Co	9.212296	62
CDC20B	Cell Divisio	Protein Co	9.136921	62
SMAP2	Small ArfG	Protein Co	9.089527	62
CISD3	CDGSH Iron	Protein Co	8.77603	62
CAPN14	Calpain 14	Protein Co	8.080542	62
CEP112	Centrosom	Protein Co	8.080542	62
DEFA4	Defensin A	Protein Co	8.080542	62

MPHOSPH!	M-Phase P	Protein Co	8.080542	62
ST7	Suppressio	Protein Co	8.080542	62
TFAP2D	Transcripti	Protein Co	8.080542	62
ZNF205	Zinc Finger	Protein Co	8.080542	62
DAZ1	Deleted In	Protein Co	7.98948	62
MIR193A	MicroRNA	RNA Gene	7.067037	62
MIR378A	MicroRNA	RNA Gene	7.067037	62
CHRAC1	Chromatin	Protein Co	6.927439	62
SGPP2	Sphingosin	Protein Co	5.694116	62
CAPS	Calcyphosi	Protein Co	5.673974	62
CDKN2B-A!	CDKN2B Ar	RNA Gene	206.1729	61
MIR146B	MicroRNA	RNA Gene	46.4603	61
MIR183	MicroRNA	RNA Gene	30.90517	61
MIRLET7I	MicroRNA	RNA Gene	23.84963	61
NAPSA	Napsin A A	Protein Co	22.54577	61
MIR486-1	MicroRNA	RNA Gene	21.20471	61
STC1	Stanniocal	Protein Co	21.12484	61
ETAA1	ETAA1 Acti	Protein Co	20.87452	61
MIR675	MicroRNA	RNA Gene	19.8395	61
MIR127	MicroRNA	RNA Gene	19.40323	61
AHSP	Alpha Hem	Protein Co	15.34098	61
ST7-OT3	ST7 Overla	RNA Gene	14.77032	61
MIR519D	MicroRNA	RNA Gene	14.4364	61
MYDGF	Myeloid De	Protein Co	14.18975	61
CCDC80	Coiled-Coil	Protein Co	12.7882	61
MIR885	MicroRNA	RNA Gene	12.77246	61
MIR493	MicroRNA	RNA Gene	12.76136	61
SPOCK2	SPARC (Ost	Protein Co	12.76136	61
KIR2DS4	Killer Cell I	Protein Co	12.74674	61
NRN1	Neuritin 1	Protein Co	12.7266	61
ABT1	Activator C	Protein Co	12.3362	61
ACTBL2	Actin Beta	Protein Co	12.3362	61
H1-6	H1.6 Linker	Protein Co	12.3362	61
NIPSNAP1	Nipsnap Ho	Protein Co	12.3362	61
ZNF703	Zinc Finger	Protein Co	9.803947	61
MIR181D	MicroRNA	RNA Gene	9.233808	61
ARGLU1	Arginine Ar	Protein Co	9.212296	61
LDHAL6B	Lactate De	Protein Co	9.212296	61
RBAK	RB Associa	Protein Co	9.212296	61
EGFL6	EGF Like D	Protein Co	9.089527	61
PTOV1	PTOV1 Ext	Protein Co	9.089527	61
HAND2-AS	HAND2 An	RNA Gene	9.000925	61
ABHD17C	Abhydrolas	Protein Co	8.080542	61
ARID3B	AT-Rich Int	Protein Co	8.080542	61
COX19	Cytochrom	Protein Co	8.080542	61
HOXA6	Homeobox	Protein Co	8.080542	61
SKOR1	SKI Family	Protein Co	8.080542	61
DAZ2	Deleted In	Protein Co	7.98948	61
DAZ4	Deleted In	Protein Co	7.98948	61
EPPIN	Epididymal	Protein Co	7.98948	61

MIR130B	MicroRNA	RNA Gene	7.067037	61
MIR324	MicroRNA	RNA Gene	7.067037	61
MSMB	Microsemi	Protein Co	5.694116	61
FGL2	Fibrinogen	Protein Co	5.597071	61
INS-IGF2	INS-IGF2 R	Protein Co	5.597071	61
CTHRC1	Collagen Tr	Protein Co	2.113806	61
ST20	Suppressor	RNA Gene	2.113806	61
CRB3	Crumbs Ce	Protein Co	1.723406	61
MIR337	MicroRNA	RNA Gene	1.723406	61
UCA1	Urothelial	RNA Gene	178.9424	60
MALAT1	Metastasis	RNA Gene	173.1878	60
MIR99B	MicroRNA	RNA Gene	148.0919	60
MIR451A	MicroRNA	RNA Gene	43.667	60
SMAD5-AS	SMAD5 An	RNA Gene	30.32395	60
MIR320B1	MicroRNA	RNA Gene	23.84963	60
MIR320D1	MicroRNA	RNA Gene	23.84963	60
MIR328	MicroRNA	RNA Gene	23.84963	60
MIR23B	MicroRNA	RNA Gene	21.81588	60
MOAP1	Modulator	Protein Co	20.86874	60
DNM3OS	DNM3 Opp	RNA Gene	20.80714	60
MIR196A1	MicroRNA	RNA Gene	19.85523	60
MON1A	MON1 Ves	Protein Co	17.40387	60
QPCTL	Glutaminyl	Protein Co	17.29284	60
MIR191	MicroRNA	RNA Gene	15.0691	60
RNU6-1	RNA, U6 Sr	RNA Gene	14.18975	60
CCN5	Cellular Co	Protein Co	14.17211	60
MIR186	MicroRNA	RNA Gene	13.99448	60
FAM98B	Family Wit	Protein Co	13.70661	60
MIR449A	MicroRNA	RNA Gene	13.23797	60
MIR26A2	MicroRNA	RNA Gene	12.80954	60
MLLT11	MLLT11 Tr	Protein Co	12.74674	60
ADGRG7	Adhesion (	Protein Co	12.7266	60
DDX60L	DExD/H-Bo	Protein Co	12.3362	60
RPL7L1	Ribosomal	Protein Co	12.3362	60
TMEM214	Transmem	Protein Co	12.3362	60
SP6	Sp6 Transc	Protein Co	10.61539	60
ZFYVE21	Zinc Finger	Protein Co	10.61279	60
ELL3	Elongation	Protein Co	9.212296	60
LOC12686	BRD4-Inde	Functional	9.201195	60
DPT	Dermatopo	Protein Co	9.136921	60
CCDC61	Coiled-Coil	Protein Co	8.080542	60
CHLSN	Cholesin	Protein Co	8.080542	60
FHL5	Four And A	Protein Co	8.080542	60
NICN1	Nicolin 1, T	Protein Co	8.080542	60
OR1E3	Olfactory F	Pseudogen	8.080542	60
SERPINB12	Serpin Fam	Protein Co	8.080542	60
TSPAN9	Tetraspani	Protein Co	8.080542	60
DND1	DND Micro	Protein Co	7.98948	60
ODF1	Outer Dens	Protein Co	7.98948	60
MIR877	MicroRNA	RNA Gene	7.067037	60

MIR218-1	MicroRNA	RNA Gene	2.036903	60
EFHB	EF-Hand D	Protein Co	1.723406	60
