# Supplementary material for: Multi-Target Anti-Endometriosis Mechanisms of Camellia sinensis Polyphenols: An Integrated Computational and Transcriptomic Study
Source: Life (Basel). 2026 Sep 26;16(10):1613. doi: 10.3390/life16101613 (PMC13705937; doi:10.3390/life16101613)
Supplement: Supplementary file 1 [file life-16-01613-s001.zip › Table S9.pdf]

Table S9

Enrichment statistics (gene ratio, fold enrichment, p-value and Benjamini–Hochberg adjusted p)

Category	ID	Description	GeneRatio	BgRatio	ldEnrichme	pvalue	p.adjust	qvalue	Count	geneID
GO	GO:0042445	hormone metabolic process	11/65	241/18903	13.27	5.62E-10	1.5E-06	9.36E-07	11	ALDH1A2/ESR1/MME/DPP4/ALDH1A3/HSD17B2/NR3C1/AKR1C2/AKR1C1/ACE/ENPEP
GO	GO:1901653	cellular response to peptide	12/65	373/18903	9.36	4.59E-09	6.2E-06	3.82E-06	12	NR4A3/NR4
GO	GO:0043434	response to peptide hormone	12/65	421/18903	8.29	1.77E-08	1.3E-05	7.82E-06	12	NR4A3/NR4
GO	GO:0030522	intracellular receptor signaling pathway	10/65	266/18903	10.93	2.33E-08	1.3E-05	7.82E-06	10	ALDH1A2/ES
GO	GO:0009410	response to xenobiotic stimulus	12/65	432/18903	8.08	2.35E-08	1.3E-05	7.82E-06	12	TYMS/CDK1/
GO	GO:0046777	protein autophosphorylation	9/65	226/18903	11.58	7.61E-08	3E-05	1.83E-05	9	NEK2/DAPK1
GO	GO:0048511	rhythmic process	10/65	302/18903	9.63	7.69E-08	3E-05	1.83E-05	10	TOP2A/TYMS
GO	GO:0050727	regulation of inflammatory response	11/65	414/18903	7.73	1.51E-07	5.1E-05	3.14E-05	11	PLA2G2A/ES
GO	GO:0097305	response to alcohol	9/65	253/18903	10.35	1.99E-07	5.9E-05	3.68E-05	9	TYMS/CDK1/
GO	GO:0048545	response to steroid hormone	10/65	339/18903	8.58	2.25E-07	6E-05	3.74E-05	10	TYMS/ESR1/
GO	GO:0051988	regulation of attachment of spindle microtubules to kinetochore	4/65	18/18903	64.63	3.76E-07	9.2E-05	5.68E-05	4	CCNB1/NEK2
GO	GO:0032103	positive regulation of response to external stimulus	11/65	464/18903	6.89	4.69E-07	0.0001	6.35E-05	11	PLA2G2A/ME
GO	GO:1901654	response to ketone	8/65	205/18903	11.35	4.96E-07	0.0001	6.35E-05	8	TYMS/PTGER
GO	GO:0050729	positive regulation of inflammatory response	7/65	154/18903	13.22	9.74E-07	0.00018	0.00011	7	PLA2G2A/SE
GO	GO:0071375	cellular response to peptide hormone stimulus	9/65	306/18903	8.55	9.8E-07	0.00018	0.00011	9	NR4A3/NR4
GO	GO:0010518	positive regulation of phospholipase activity	5/65	55/18903	26.44	1.25E-06	0.00021	0.00013	5	ESR1/AGTR1
GO	GO:0060326	cell chemotaxis	9/65	319/18903	8.2	1.38E-06	0.00022	0.00014	9	MET/SERPIN
GO	GO:0008202	steroid metabolic process	9/65	323/18903	8.1	1.53E-06	0.00023	0.00014	9	HSD11B1/ES
GO	GO:1901655	cellular response to ketone	6/65	105/18903	16.62	1.61E-06	0.00023	0.00014	6	PTGER2/NR3
GO	GO:0070482	response to oxygen levels	9/65	337/18903	7.77	2.17E-06	0.00029	0.00018	9	NR4A2/HSD1
GO	GO:0062012	regulation of small molecule metabolic process	9/65	339/18903	7.72	2.28E-06	0.00029	0.00018	9	NR4A3/NR1H
GO	GO:0062197	cellular response to chemical stress	9/65	345/18903	7.59	2.63E-06	0.00032	0.0002	9	CDK1/MYLK/
GO	GO:0031100	animal organ regeneration	5/65	65/18903	22.37	2.9E-06	0.00034	0.00021	5	TYMS/CDK1/
GO	GO:0010517	regulation of phospholipase activity	5/65	66/18903	22.03	3.12E-06	0.00035	0.00022	5	ESR1/AGTR1
GO	GO:0060193	positive regulation of lipase activity	5/65	68/18903	21.38	3.63E-06	0.00039	0.00024	5	ESR1/AGTR1
GO	GO:0031667	response to nutrient levels	10/65	474/18903	6.14	4.65E-06	0.00048	0.0003	10	ALDH1A2/TY
GO	GO:0050673	epithelial cell proliferation	10/65	481/18903	6.05	5.3E-06	0.00052	0.00033	10	ALDH1A2/ES
GO	GO:0033238	regulation of cellular amine metabolic process	4/65	34/18903	34.21	5.46E-06	0.00052	0.00033	4	NR4A2/NR1H
GO	GO:0018108	peptidyl-tyrosine phosphorylation	9/65	380/18903	6.89	5.77E-06	0.00053	0.00033	9	MET/JAK3/IN
GO	GO:0018212	peptidyl-tyrosine modification	9/65	382/18903	6.85	6.02E-06	0.00054	0.00033	9	MET/JAK3/IN
GO	GO:0051987	positive regulation of attachment of spindle microtubules to kinetoch	3/65	11/18903	79.31	6.28E-06	0.00054	0.00034	3	CCNB1/AURK
GO	GO:0007204	positive regulation of cytosolic calcium ion concentration	7/65	205/18903	9.93	6.51E-06	0.00055	0.00034	7	ESR1/JAK3/P
GO	GO:0007623	circadian rhythm	7/65	210/18903	9.69	7.62E-06	0.00062	0.00038	7	TOP2A/TYMS
GO	GO:0140448	signaling receptor ligand precursor processing	4/65	38/18903	30.61	8.61E-06	0.00068	0.00042	4	MME/DPP4/
GO	GO:0034754	cellular hormone metabolic process	6/65	141/18903	12.38	8.91E-06	0.00068	0.00042	6	ALDH1A2/ES
GO	GO:0001990	regulation of systemic arterial blood pressure by hormone	4/65	39/18903	29.83	9.57E-06	0.00071	0.00044	4	HSD11B2/AG
GO	GO:0042180	cellular ketone metabolic process	7/65	218/18903	9.34	9.73E-06	0.00071	0.00044	7	NR4A3/NR1H
GO	GO:0031349	positive regulation of defense response	8/65	307/18903	7.58	9.98E-06	0.00071	0.00044	8	PLA2G2A/SE
GO	GO:0036293	response to decreased oxygen levels	8/65	309/18903	7.53	1.05E-05	0.00071	0.00044	8	NR4A2/HSD1
GO	GO:0010863	positive regulation of phospholipase C activity	4/65	40/18903	29.08	1.06E-05	0.00071	0.00044	4	ESR1/KIT/FLT
GO	GO:0042417	dopamine metabolic process	4/65	41/18903	28.37	1.17E-05	0.00077	0.00048	4	NR4A2/SLC1
GO	GO:1900274	regulation of phospholipase C activity	4/65	42/18903	27.7	1.29E-05	0.00083	0.00051	4	ESR1/KIT/FLT
GO	GO:0008608	attachment of spindle microtubules to kinetochore	4/65	43/18903	27.05	1.42E-05	0.00088	0.00055	4	CCNB1/NEK2
GO	GO:0060191	regulation of lipase activity	5/65	90/18903	16.16	1.44E-05	0.00088	0.00055	5	ESR1/AGTR1
GO	GO:0007584	response to nutrient	6/65	154/18903	11.33	1.47E-05	0.00088	0.00055	6	ALDH1A2/TY
GO	GO:0014075	response to amine	4/65	44/18903	26.44	1.56E-05	0.0009	0.00056	4	CDK1/NR4A2
GO	GO:0072593	reactive oxygen species metabolic process	7/65	235/18903	8.66	1.58E-05	0.0009	0.00056	7	ARG2/SLC1A
GO	GO:0050886	endocrine process	5/65	93/18903	15.64	1.69E-05	0.00095	0.00059	5	HSD11B2/AG
GO	GO:0120254	olefinic compound metabolic process	6/65	160/18903	10.91	1.83E-05	0.001	0.00062	6	ALDH1A2/HS
GO	GO:0048732	gland development	9/65	441/18903	5.94	0.000019	0.00102	0.00063	9	ALDH1A2/TY
GO	GO:0032496	response to lipopolysaccharide	8/65	339/18903	6.86	2.04E-05	0.00107	0.00067	8	NR1H4/SERP
GO	GO:0031960	response to corticosteroid	6/65	165/18903	10.58	2.18E-05	0.00112	0.0007	6	TYMS/HSD11
GO	GO:0003044	regulation of systemic arterial blood pressure mediated by a chemical	4/65	49/18903	23.74	0.000024	0.00119	0.00074	4	HSD11B2/AG
GO	GO:0097306	cellular response to alcohol	5/65	100/18903	14.54	2.41E-05	0.00119	0.00074	5	PTGER2/AKR
GO	GO:0033044	regulation of chromosome organization	7/65	252/18903	8.08	2.48E-05	0.00119	0.00074	7	TOP2A/CCNE
GO	GO:0007568	aging	6/65	169/18903	10.32	2.49E-05	0.00119	0.00074	6	TYMS/MME/
GO	GO:0090399	replicative senescence	3/65	17/18903	51.32	2.55E-05	0.0012	0.00074	3	MME/SERPIN
GO	GO:0101023	vascular endothelial cell proliferation	4/65	51/18903	22.81	2.82E-05	0.00128	0.0008	4	ALDH1A2/PP
GO	GO:1905562	regulation of vascular endothelial cell proliferation	4/65	51/18903	22.81	2.82E-05	0.00128	0.0008	4	ALDH1A2/PP
GO	GO:0090068	positive regulation of cell cycle process	7/65	260/18903	7.83	3.03E-05	0.00135	0.00084	7	CDK1/CCNB1
GO	GO:0002237	response to molecule of bacterial origin	8/65	360/18903	6.46	3.14E-05	0.00138	0.00086	8	NR1H4/SERP
GO	GO:0034308	primary alcohol metabolic process	5/65	108/18903	13.46	3.49E-05	0.00148	0.00092	5	ALDH1A2/HS
GO	GO:0044106	cellular amine metabolic process	5/65	108/18903	13.46	3.49E-05	0.00148	0.00092	5	NR4A2/NR1H
GO	GO:0042574	retinal metabolic process	3/65	19/18903	45.92	3.62E-05	0.0015	0.00093	3	ALDH1A2/AL
GO	GO:0032868	response to insulin	7/65	269/18903	7.57	3.76E-05	0.0015	0.00093	7	NR1H4/HSD1
GO	GO:0006584	catecholamine metabolic process	4/65	55/18903	21.15	3.81E-05	0.0015	0.00093	4	NR4A2/SLC1
GO	GO:0009712	catechol-containing compound metabolic process	4/65	55/18903	21.15	3.81E-05	0.0015	0.00093	4	NR4A2/SLC1
GO	GO:1902749	regulation of cell cycle G2/M phase transition	5/65	110/18903	13.22	3.81E-05	0.0015	0.00093	5	CDK1/CCNB1
GO	GO:0001936	regulation of endothelial cell proliferation	6/65	183/18903	9.53	0.000039	0.00152	0.00094	6	ALDH1A2/AG
GO	GO:0018958	phenol-containing compound metabolic process	5/65	111/18903	13.1	3.98E-05	0.00152	0.00095	5	NR4A2/SLC1
GO	GO:0042053	regulation of dopamine metabolic process	3/65	20/18903	43.62	4.24E-05	0.00156	0.00097	3	NR4A2/ITGB
GO	GO:0042069	regulation of catecholamine metabolic process	3/65	20/18903	43.62	4.24E-05	0.00156	0.00097	3	NR4A2/ITGB
GO	GO:0071379	cellular response to prostaglandin stimulus	3/65	20/18903	43.62	4.24E-05	0.00156	0.00097	3	PTGER2/AKR
GO	GO:1904659	glucose transmembrane transport	5/65	113/18903	12.87	4.33E-05	0.00157	0.00098	5	NR4A3/SLC1
GO	GO:0007565	female pregnancy	6/65	189/18903	9.23	4.67E-05	0.00167	0.00104	6	ESR1/PGR/H
GO	GO:0008645	hexose transmembrane transport	5/65	116/18903	12.54	4.91E-05	0.00173	0.00107	5	NR4A3/SLC1
GO	GO:0008217	regulation of blood pressure	6/65	191/18903	9.14	4.95E-05	0.00173	0.00107	6	HSD11B2/CN
GO	GO:0009308	amine metabolic process	5/65	117/18903	12.43	5.12E-05	0.00176	0.00109	5	NR4A2/NR1H
GO	GO:0007088	regulation of mitotic nuclear division	5/65	118/18903	12.32	5.33E-05	0.00181	0.00112	5	CCNB1/NEK2
GO	GO:0015749	monosaccharide transmembrane transport	5/65	119/18903	12.22	5.55E-05	0.00186	0.00115	5	NR4A3/SLC1
GO	GO:1902644	tertiary alcohol metabolic process	3/65	22/18903	39.66	0.000057	0.00189	0.00117	3	HSD11B2/AK
GO	GO:0034599	cellular response to oxidative stress	7/65	288/18903	7.07	0.000058	0.0019	0.00118	7	CDK1/NR4A3

GO	GO:0070228	regulation of lymphocyte apoptotic process	4/65	62/18903	18.76	6.11E-05	0.00198	0.00123	4	ARG2/JAK3/AURKB/TP53
GO	GO:0001935	endothelial cell proliferation	6/65	199/18903	8.77	6.22E-05	0.00199	0.00123	6	ALDH1A2/AGTR1/PPARG/NR4A1/FLT1/FGFR1
GO	GO:0002366	leukocyte activation involved in immune response	7/65	293/18903	6.95	6.46E-05	0.00204	0.00126	7	NR4A3/JAK3
GO	GO:0060965	negative regulation of miRNA-mediated gene silencing	3/65	23/18903	37.93	6.54E-05	0.00204	0.00127	3	ESR1/TP53/P
GO	GO:0001666	response to hypoxia	7/65	296/18903	6.88	6.88E-05	0.00212	0.00132	7	NR4A2/HSD11B2/DPP4/TP53/PPARG/SLC2A1/ACE
GO	GO:0002263	cell activation involved in immune response	7/65	297/18903	6.85	7.03E-05	0.00214	0.00133	7	NR4A3/JAK3
GO	GO:0060149	negative regulation of post-transcriptional gene silencing	3/65	24/18903	36.35	7.46E-05	0.0022	0.00136	3	ESR1/TP53/P
GO	GO:0060967	negative regulation of gene silencing by RNA	3/65	24/18903	36.35	7.46E-05	0.0022	0.00136	3	ESR1/TP53/P
GO	GO:1900369	negative regulation of post-transcriptional gene silencing by RNA	3/65	24/18903	36.35	7.46E-05	0.0022	0.00136	3	ESR1/TP53/P
GO	GO:0046683	response to organophosphorus	5/65	128/18903	11.36	7.85E-05	0.00229	0.00142	5	TYMS/AKR1C
GO	GO:0044703	multi-organism reproductive process	6/65	209/18903	8.35	8.16E-05	0.00234	0.00145	6	ESR1/PGR/H
GO	GO:0015909	long-chain fatty acid transport	4/65	67/18903	17.36	8.29E-05	0.00234	0.00145	4	PLA2G2A/PP
GO	GO:0050678	regulation of epithelial cell proliferation	8/65	414/18903	5.62	8.38E-05	0.00234	0.00145	8	ALDH1A2/PG
GO	GO:0003081	regulation of systemic arterial blood pressure by renin-angiotensin	3/65	25/18903	34.9	8.46E-05	0.00234	0.00145	3	AGTR1/ACE/
GO	GO:0051984	positive regulation of chromosome segregation	3/65	25/18903	34.9	8.46E-05	0.00234	0.00145	3	CCNB1/AURK
GO	GO:0060541	respiratory system development	6/65	211/18903	8.27	0.000086	0.00235	0.00146	6	HSD11B1/AL
GO	GO:0034219	carbohydrate transmembrane transport	5/65	132/18903	11.02	9.08E-05	0.00246	0.00153	5	NR4A3/SLC1
GO	GO:0002244	hematopoietic progenitor cell differentiation	5/65	133/18903	10.93	9.41E-05	0.00253	0.00157	5	TOP2A/TP53
GO	GO:0044706	multi-multicellular organism process	6/65	218/18903	8	0.000103	0.00273	0.0017	6	ESR1/PGR/H
GO	GO:2000379	positive regulation of reactive oxygen species metabolic process	4/65	71/18903	16.38	0.000104	0.00274	0.0017	4	AGTR1/TP53
GO	GO:0006979	response to oxidative stress	8/65	434/18903	5.36	0.000116	0.00303	0.00188	8	CDK1/NR4A3
GO	GO:0015849	organic acid transport	7/65	324/18903	6.28	0.000121	0.00312	0.00194	7	PLA2G2A/SL
GO	GO:0051384	response to glucocorticoid	5/65	142/18903	10.24	0.000128	0.00326	0.00202	5	TYMS/HSD11
GO	GO:0046323	glucose import	4/65	75/18903	15.51	0.000129	0.00326	0.00202	4	SLC1A2/INSR
GO	GO:0071496	cellular response to external stimulus	7/65	328/18903	6.21	0.000131	0.00327	0.00203	7	NR4A2/NR1H
GO	GO:0051783	regulation of nuclear division	5/65	145/18903	10.03	0.000141	0.0035	0.00217	5	CCNB1/NEK2
GO	GO:0010827	regulation of glucose transmembrane transport	4/65	77/18903	15.11	0.000143	0.0035	0.00217	4	NR4A3/SLC1
GO	GO:0001975	response to amphetamine	3/65	30/18903	29.08	0.000147	0.0035	0.00217	3	NR4A2/SLC1
GO	GO:0034368	protein-lipid complex remodeling	3/65	30/18903	29.08	0.000147	0.0035	0.00217	3	PLA2G2A/NR
GO	GO:0034369	plasma lipoprotein particle remodeling	3/65	30/18903	29.08	0.000147	0.0035	0.00217	3	PLA2G2A/NR
GO	GO:0071214	cellular response to abiotic stimulus	7/65	335/18903	6.08	0.000149	0.0035	0.00217	7	MME/MYLK/
GO	GO:0104004	cellular response to environmental stimulus	7/65	335/18903	6.08	0.000149	0.0035	0.00217	7	MME/MYLK/
GO	GO:2000377	regulation of reactive oxygen species metabolic process	5/65	149/18903	9.76	0.000161	0.0037	0.0023	5	ARG2/AGTR1
GO	GO:0034694	response to prostaglandin	3/65	31/18903	28.14	0.000163	0.0037	0.0023	3	PTGER2/AKR
GO	GO:0035767	endothelial cell chemotaxis	3/65	31/18903	28.14	0.000163	0.0037	0.0023	3	MET/NR4A1/
GO	GO:0060603	mammary gland duct morphogenesis	3/65	31/18903	28.14	0.000163	0.0037	0.0023	3	ESR1/PGR/N
GO	GO:0014068	positive regulation of phosphatidylinositol 3-kinase signaling	4/65	80/18903	14.54	0.000166	0.0037	0.0023	4	INSR/KIT/FLT
GO	GO:0070227	lymphocyte apoptotic process	4/65	80/18903	14.54	0.000166	0.0037	0.0023	4	ARG2/JAK3/
GO	GO:0000819	sister chromatid segregation	6/65	239/18903	7.3	0.00017	0.00376	0.00233	6	TOP2A/CCNB
GO	GO:0046718	viral entry into host cell	5/65	151/18903	9.63	0.000171	0.00376	0.00233	5	CDK1/DPP4/
GO	GO:1901657	glycosyl compound metabolic process	4/65	81/18903	14.36	0.000174	0.00379	0.00235	4	TK1/AKR1C2
GO	GO:0062013	positive regulation of small molecule metabolic process	5/65	152/18903	9.57	0.000176	0.00381	0.00237	5	NR4A3/NR1H
GO	GO:0010743	regulation of macrophage derived foam cell differentiation	3/65	32/18903	27.26	0.000179	0.00382	0.00237	3	PLA2G2A/AG
GO	GO:0034367	protein-containing complex remodeling	3/65	32/18903	27.26	0.000179	0.00382	0.00237	3	PLA2G2A/NR
GO	GO:0097529	myeloid leukocyte migration	6/65	242/18903	7.21	0.000182	0.00384	0.00238	6	SERPINE1/DP
GO	GO:0019216	regulation of lipid metabolic process	7/65	347/18903	5.87	0.000185	0.00387	0.0024	7	NR4A3/NR1H
GO	GO:0045834	positive regulation of lipid metabolic process	5/65	154/18903	9.44	0.000187	0.0039	0.00242	5	NR4A3/NR1H
GO	GO:0043171	peptide catabolic process	3/65	33/18903	26.44	0.000197	0.00406	0.00252	3	MME/ACE/E
GO	GO:0008643	carbohydrate transport	5/65	156/18903	9.32	0.000199	0.00407	0.00253	5	NR4A3/SLC1
GO	GO:0044839	cell cycle G2/M phase transition	5/65	157/18903	9.26	0.000205	0.00416	0.00258	5	CDK1/CCNB1
GO	GO:0042593	glucose homeostasis	6/65	248/18903	7.04	0.000208	0.00419	0.0026	6	NR1H4/CNR1
GO	GO:0044409	entry into host	5/65	158/18903	9.2	0.000211	0.00421	0.00262	5	CDK1/DPP4/
GO	GO:0033500	carbohydrate homeostasis	6/65	249/18903	7.01	0.000212	0.00421	0.00262	6	NR1H4/CNR1
GO	GO:0061081	positive regulation of myeloid leukocyte cytokine production involved	3/65	34/18903	25.66	0.000215	0.00424	0.00263	3	NR4A3/KIT/N
GO	GO:0071695	anatomical structure maturation	6/65	250/18903	6.98	0.000217	0.00424	0.00263	6	ALDH1A2/TY
GO	GO:0010232	vascular transport	4/65	87/18903	13.37	0.000229	0.00441	0.00274	4	SLC1A2/INSR
GO	GO:0150104	transport across blood-brain barrier	4/65	87/18903	13.37	0.000229	0.00441	0.00274	4	SLC1A2/INSR
GO	GO:0001655	urogenital system development	7/65	360/18903	5.65	0.000231	0.00443	0.00275	7	ALDH1A2/ES
GO	GO:0071398	cellular response to fatty acid	3/65	35/18903	24.93	0.000235	0.00447	0.00277	3	NR1H4/AKR1
GO	GO:0045787	positive regulation of cell cycle	7/65	362/18903	5.62	0.000239	0.00448	0.00278	7	CDK1/CCNB1
GO	GO:0033627	cell adhesion mediated by integrin	4/65	88/18903	13.22	0.000239	0.00448	0.00278	4	SERPINE1/DP
GO	GO:0022411	cellular component disassembly	8/65	483/18903	4.82	0.000241	0.00449	0.00279	8	TOP2A/CDK1
GO	GO:0016486	peptide hormone processing	3/65	36/18903	24.23	0.000255	0.00466	0.00289	3	DPP4/ACE/E
GO	GO:0045777	positive regulation of blood pressure	3/65	36/18903	24.23	0.000255	0.00466	0.00289	3	HSD11B2/CN
GO	GO:0010639	negative regulation of organelle organization	7/65	366/18903	5.56	0.000256	0.00466	0.00289	7	TOP2A/CCNB
GO	GO:0006066	alcohol metabolic process	7/65	367/18903	5.55	0.00026	0.00469	0.00291	7	ALDH1A2/NR1H4/HSD11B2/ALDH1A3/AKR1C2/IP6K2/AKR1C1
GO	GO:0051262	protein tetramerization	4/65	90/18903	12.93	0.000261	0.00469	0.00291	4	ALDH1A2/TK
GO	GO:0070229	negative regulation of lymphocyte apoptotic process	3/65	37/18903	23.58	0.000277	0.00495	0.00307	3	ARG2/JAK3/
GO	GO:0031668	cellular response to extracellular stimulus	6/65	262/18903	6.66	0.000279	0.00495	0.00307	6	NR4A2/NR1H
GO	GO:0033674	positive regulation of kinase activity	8/65	496/18903	4.69	0.000288	0.00509	0.00316	8	CCNB1/MET/
GO	GO:2000106	regulation of leukocyte apoptotic process	4/65	93/18903	12.51	0.000295	0.00518	0.00321	4	ARG2/JAK3/
GO	GO:0010742	macrophage derived foam cell differentiation	3/65	38/18903	22.96	0.0003	0.00523	0.00325	3	PLA2G2A/AG
GO	GO:0002697	regulation of immune effector process	7/65	379/18903	5.37	0.000316	0.00546	0.00339	7	NR4A3/JAK3
GO	GO:0090077	foam cell differentiation	3/65	39/18903	22.37	0.000325	0.00555	0.00344	3	PLA2G2A/AG
GO	GO:0097242	amyloid-beta clearance	3/65	39/18903	22.37	0.000325	0.00555	0.00344	3	MME/INSR/I
GO	GO:0003018	vascular process in circulatory system	6/65	270/18903	6.46	0.000328	0.00556	0.00345	6	SLC1A2/INSR
GO	GO:0007059	chromosome segregation	7/65	382/18903	5.33	0.000331	0.00558	0.00346	7	TOP2A/CCNB
GO	GO:0003073	regulation of systemic arterial blood pressure	4/65	96/18903	12.12	0.000334	0.00559	0.00347	4	HSD11B2/AG
GO	GO:0006576	cellular biogenic amine metabolic process	4/65	97/18903	11.99	0.000347	0.00575	0.00357	4	NR4A2/SLC1
GO	GO:0007611	learning or memory	6/65	273/18903	6.39	0.000347	0.00575	0.00357	6	MME/CNR1/
GO	GO:0010389	regulation of G2/M transition of mitotic cell cycle	4/65	99/18903	11.75	0.000375	0.00616	0.00382	4	CDK1/CCNB1
GO	GO:0070232	regulation of T cell apoptotic process	3/65	41/18903	21.28	0.000377	0.00616	0.00382	3	ARG2/JAK3/T
GO	GO:0071900	regulation of protein serine/threonine kinase activity	7/65	395/18903	5.15	0.000405	0.00654	0.00406	7	CCNB1/INSR
GO	GO:0015908	fatty acid transport	4/65	101/18903	11.52	0.000405	0.00654	0.00406	4	PLA2G2A/PP
GO	GO:0044000	movement in host	5/65	183/18903	7.95	0.000416	0.00667	0.00414	5	CDK1/DPP4/
GO	GO:0050900	leukocyte migration	7/65	398/18903	5.11	0.000424	0.00676	0.0042	7	SERPINE1/DP

GO	GO:0048144	fibroblast proliferation	4/65	103/18903	11.29	0.000436	0.00692	0.00429	4	ESR1/CDK1/CCNB1/TP53
GO	GO:0043405	regulation of MAP kinase activity	5/65	186/18903	7.82	0.000448	0.00706	0.00438	5	INSR/PPARG/
GO	GO:0030324	lung development	5/65	187/18903	7.78	0.000459	0.00715	0.00444	5	HSD11B1/ALDH1A2/MME/PGR/ACE
GO	GO:0048469	cell maturation	5/65	187/18903	7.78	0.000459	0.00715	0.00444	5	TYMS/PGR/N
GO	GO:0010828	positive regulation of glucose transmembrane transport	3/65	44/18903	19.83	0.000465	0.0072	0.00447	3	NR4A3/SLC1/
GO	GO:0008585	female gonad development	4/65	105/18903	11.08	0.000469	0.0072	0.00447	4	ESR1/PGR/IN
GO	GO:0045786	negative regulation of cell cycle	7/65	405/18903	5.03	0.00047	0.0072	0.00447	7	CDK1/CCNB1/NEK2/AURKB/TP53/KAT2B/NR4A1
GO	GO:2001252	positive regulation of chromosome organization	4/65	106/18903	10.97	0.000486	0.00734	0.00456	4	NEK2/AURKE
GO	GO:0060964	regulation of miRNA-mediated gene silencing	3/65	45/18903	19.39	0.000497	0.00734	0.00456	3	ESR1/TP53/F
GO	GO:0071827	plasma lipoprotein particle organization	3/65	45/18903	19.39	0.000497	0.00734	0.00456	3	PLA2G2A/NR
GO	GO:0002444	myeloid leukocyte mediated immunity	4/65	107/18903	10.87	0.000504	0.00734	0.00456	4	NR4A3/KIT/I
GO	GO:0010906	regulation of glucose metabolic process	4/65	107/18903	10.87	0.000504	0.00734	0.00456	4	INSR/NR3C1/
GO	GO:0030323	respiratory tube development	5/65	191/18903	7.61	0.000505	0.00734	0.00456	5	HSD11B1/AL
GO	GO:0002002	regulation of angiotensin levels in blood	2/65	10/18903	58.16	0.000515	0.00734	0.00456	2	ACE/ENPEP
GO	GO:0002138	retinoic acid biosynthetic process	2/65	10/18903	58.16	0.000515	0.00734	0.00456	2	ALDH1A2/AL
GO	GO:0009157	deoxyribonucleoside monophosphate biosynthetic process	2/65	10/18903	58.16	0.000515	0.00734	0.00456	2	TYMS/TK1
GO	GO:0030638	polyketide metabolic process	2/65	10/18903	58.16	0.000515	0.00734	0.00456	2	AKR1C2/AKR
GO	GO:0030647	aminoglycoside antibiotic metabolic process	2/65	10/18903	58.16	0.000515	0.00734	0.00456	2	AKR1C2/AKR
GO	GO:0044598	doxorubicin metabolic process	2/65	10/18903	58.16	0.000515	0.00734	0.00456	2	AKR1C2/AKR
GO	GO:0009755	hormone-mediated signaling pathway	5/65	192/18903	7.57	0.000517	0.00734	0.00456	5	ESR1/PGR/N
GO	GO:0031099	regeneration	5/65	192/18903	7.57	0.000517	0.00734	0.00456	5	TYMS/CDK1/
GO	GO:0060443	mammary gland morphogenesis	3/65	46/18903	18.97	0.00053	0.00748	0.00464	3	ESR1/PGR/N
GO	GO:0007159	leukocyte cell-cell adhesion	7/65	414/18903	4.92	0.000536	0.00752	0.00467	7	NR4A3/ARG2
GO	GO:0046545	development of primary female sexual characteristics	4/65	109/18903	10.67	0.00054	0.00755	0.00468	4	ESR1/PGR/IN
GO	GO:0051972	regulation of telomerase activity	3/65	47/18903	18.56	0.000565	0.00777	0.00482	3	NEK2/AURKE
GO	GO:1900368	regulation of post-transcriptional gene silencing by RNA	3/65	47/18903	18.56	0.000565	0.00777	0.00482	3	ESR1/TP53/F
GO	GO:1902895	positive regulation of miRNA transcription	3/65	47/18903	18.56	0.000565	0.00777	0.00482	3	NR3C1/TP53
GO	GO:0014066	regulation of phosphatidylinositol 3-kinase signaling	4/65	111/18903	10.48	0.000579	0.00792	0.00491	4	INSR/KIT/FLT
GO	GO:0045860	positive regulation of protein kinase activity	7/65	420/18903	4.85	0.000583	0.00794	0.00493	7	CCNB1/INSR/
GO	GO:0016032	viral process	7/65	421/18903	4.84	0.000592	0.00798	0.00495	7	TOP2A/CDK1
GO	GO:0071897	DNA biosynthetic process	5/65	198/18903	7.34	0.000595	0.00798	0.00495	5	TYMS/NEK2/
GO	GO:0042220	response to cocaine	3/65	48/18903	18.18	0.000601	0.00798	0.00495	3	SLC1A2/CNR
GO	GO:0060147	regulation of post-transcriptional gene silencing	3/65	48/18903	18.18	0.000601	0.00798	0.00495	3	ESR1/TP53/F
GO	GO:0071825	protein-lipid complex subunit organization	3/65	48/18903	18.18	0.000601	0.00798	0.00495	3	PLA2G2A/NR
GO	GO:0021700	developmental maturation	6/65	304/18903	5.74	0.000613	0.00805	0.005	6	ALDH1A2/TY
GO	GO:0001660	fever generation	2/65	11/18903	52.88	0.000628	0.00805	0.005	2	PTGER3/CNR
GO	GO:0002069	columnar/cuboidal epithelial cell maturation	2/65	11/18903	52.88	0.000628	0.00805	0.005	2	TYMS/PGR
GO	GO:0014831	gastro-intestinal system smooth muscle contraction	2/65	11/18903	52.88	0.000628	0.00805	0.005	2	PTGER3/KIT
GO	GO:0016102	diterpenoid biosynthetic process	2/65	11/18903	52.88	0.000628	0.00805	0.005	2	ALDH1A2/AL
GO	GO:0019062	virion attachment to host cell	2/65	11/18903	52.88	0.000628	0.00805	0.005	2	DPP4/ICAM1
GO	GO:0070778	L-aspartate transmembrane transport	2/65	11/18903	52.88	0.000628	0.00805	0.005	2	SLC1A2/SLC1
GO	GO:0008206	bile acid metabolic process	3/65	49/18903	17.81	0.000639	0.00816	0.00506	3	NR1H4/KIT/
GO	GO:0051701	biological process involved in interaction with host	5/65	203/18903	7.16	0.000666	0.00845	0.00524	5	CDK1/DPP4/
GO	GO:0001822	kidney development	6/65	309/18903	5.65	0.000668	0.00845	0.00524	6	ALDH1A2/M
GO	GO:0060966	regulation of gene silencing by RNA	3/65	50/18903	17.45	0.000678	0.00849	0.00527	3	ESR1/TP53/F
GO	GO:0061082	myeloid leukocyte cytokine production	3/65	50/18903	17.45	0.000678	0.00849	0.00527	3	NR4A3/KIT/N
GO	GO:0043281	regulation of cysteine-type endopeptidase activity involved in apopto	5/65	205/18903	7.09	0.000696	0.00868	0.00539	5	DAPK1/SLC1/
GO	GO:0043406	positive regulation of MAP kinase activity	4/65	117/18903	9.94	0.000705	0.00872	0.00541	4	INSR/KIT/FLT
GO	GO:0071887	leukocyte apoptotic process	4/65	117/18903	9.94	0.000705	0.00872	0.00541	4	ARG2/JAK3/
GO	GO:2000241	regulation of reproductive process	5/65	206/18903	7.06	0.000712	0.00875	0.00543	5	ESR1/CNR1/I
GO	GO:0060324	face development	3/65	51/18903	17.11	0.000718	0.00876	0.00544	3	ALDH1A2/AL
GO	GO:0050890	cognition	6/65	314/18903	5.56	0.000726	0.00876	0.00544	6	MME/CNR1/
GO	GO:0032102	negative regulation of response to external stimulus	7/65	436/18903	4.67	0.000727	0.00876	0.00544	7	ARG2/NR1H
GO	GO:0002286	T cell activation involved in immune response	4/65	118/18903	9.86	0.000728	0.00876	0.00544	4	JAK3/TP53/P
GO	GO:0002718	regulation of cytokine production involved in immune response	4/65	118/18903	9.86	0.000728	0.00876	0.00544	4	NR4A3/JAK3/
GO	GO:0019627	urea metabolic process	2/65	12/18903	48.47	0.000752	0.00884	0.00549	2	ARG2/NR1H
GO	GO:0031650	regulation of heat generation	2/65	12/18903	48.47	0.000752	0.00884	0.00549	2	PTGER3/CNR
GO	GO:0042447	hormone catabolic process	2/65	12/18903	48.47	0.000752	0.00884	0.00549	2	MME/ACE
GO	GO:0071941	nitrogen cycle metabolic process	2/65	12/18903	48.47	0.000752	0.00884	0.00549	2	ARG2/NR1H
GO	GO:1904177	regulation of adipose tissue development	2/65	12/18903	48.47	0.000752	0.00884	0.00549	2	NR1H4/PPAR
GO	GO:0071383	cellular response to steroid hormone stimulus	5/65	209/18903	6.96	0.000759	0.00885	0.00549	5	ESR1/PGR/N
GO	GO:0002367	cytokine production involved in immune response	4/65	120/18903	9.69	0.000776	0.00885	0.00549	4	NR4A3/JAK3/
GO	GO:0007613	memory	4/65	120/18903	9.69	0.000776	0.00885	0.00549	4	CNR1/INSR/S
GO	GO:0042752	regulation of circadian rhythm	4/65	120/18903	9.69	0.000776	0.00885	0.00549	4	TOP2A/CDK1
GO	GO:0051897	positive regulation of protein kinase B signaling	4/65	120/18903	9.69	0.000776	0.00885	0.00549	4	MET/INSR/A
GO	GO:0010948	negative regulation of cell cycle process	6/65	318/18903	5.49	0.000776	0.00885	0.00549	6	CDK1/CCNB1
GO	GO:0072001	renal system development	6/65	318/18903	5.49	0.000776	0.00885	0.00549	6	ALDH1A2/M
GO	GO:0019058	viral life cycle	6/65	319/18903	5.47	0.000789	0.00896	0.00556	6	TOP2A/CDK1
GO	GO:0009416	response to light stimulus	6/65	321/18903	5.44	0.000815	0.00918	0.0057	6	MME/AURKE
GO	GO:0098813	nuclear chromosome segregation	6/65	321/18903	5.44	0.000815	0.00918	0.0057	6	TOP2A/CCNE
GO	GO:0002064	epithelial cell development	5/65	213/18903	6.83	0.000827	0.00928	0.00576	5	TYMS/ESR1/I
GO	GO:0002683	negative regulation of immune system process	7/65	449/18903	4.53	0.000864	0.00962	0.00597	7	ARG2/JAK3/D
GO	GO:0140014	mitotic nuclear division	6/65	325/18903	5.37	0.000869	0.00962	0.00597	6	CCNB1/NEK2
GO	GO:1901214	regulation of neuron death	6/65	325/18903	5.37	0.000869	0.00962	0.00597	6	NR4A3/NR4A
GO	GO:0046660	female sex differentiation	4/65	124/18903	9.38	0.000877	0.00962	0.00597	4	ESR1/PGR/IN
GO	GO:0003084	positive regulation of systemic arterial blood pressure	2/65	13/18903	44.74	0.000886	0.00962	0.00597	2	HSD11B2/AC
GO	GO:0044650	adhesion of symbiont to host cell	2/65	13/18903	44.74	0.000886	0.00962	0.00597	2	DPP4/ICAM1
GO	GO:0070243	regulation of thymocyte apoptotic process	2/65	13/18903	44.74	0.000886	0.00962	0.00597	2	JAK3/TP53
GO	GO:0090235	regulation of metaphase plate congression	2/65	13/18903	44.74	0.000886	0.00962	0.00597	2	AURKB/KAT2
GO	GO:2000107	negative regulation of leukocyte apoptotic process	3/65	55/18903	15.86	0.000896	0.00969	0.00601	3	ARG2/JAK3/
GO	GO:0043280	positive regulation of cysteine-type endopeptidase activity involved ir	4/65	125/18903	9.31	0.000903	0.00973	0.00604	4	DAPK1/SLC1/
GO	GO:0003012	muscle system process	7/65	455/18903	4.47	0.000934	0.01	0.00622	7	MYLK/NR4A3
GO	GO:0033047	regulation of mitotic sister chromatid segregation	3/65	56/18903	15.58	0.000944	0.0101	0.00624	3	CCNB1/AURK
GO	GO:2000630	positive regulation of miRNA metabolic process	3/65	56/18903	15.58	0.000944	0.0101	0.00624	3	NR3C1/TP53
GO	GO:0045471	response to ethanol	4/65	128/18903	9.09	0.000987	0.0105	0.00649	4	TYMS/CDK1/
GO	GO:0007080	mitotic metaphase plate congression	3/65	57/18903	15.31	0.000995	0.0105	0.00649	3	CCNB1/AURK

GO	GO:0070231	T cell apoptotic process	3/65	57/18903	15.31	0.000995	0.0105	0.00649	3	ARG2/JAK3/TP53
GO	GO:0051604	protein maturation	6/65	336/18903	5.19	0.00103	0.0107	0.00666	6	MME/SERPINE1/DPP4/KAT2B/ACE/ENPEP
GO	GO:0071100	mitotic centrosome separation	2/65	14/18903	41.55	0.00103	0.0107	0.00666	2	NEK2/AURKA
GO	GO:0002443	leukocyte mediated immunity	7/65	463/18903	4.4	0.00103	0.0107	0.00666	7	NR4A3/JAK3/
GO	GO:0042542	response to hydrogen peroxide	4/65	130/18903	8.95	0.00105	0.0108	0.00672	4	CDK1/NR4A3
GO	GO:0046324	regulation of glucose import	3/65	59/18903	14.79	0.0011	0.0113	0.00701	3	SLC1A2/INSR
GO	GO:0051289	protein homotetramerization	3/65	59/18903	14.79	0.0011	0.0113	0.00701	3	ALDH1A2/TK
GO	GO:0007052	mitotic spindle organization	4/65	133/18903	8.75	0.00114	0.0116	0.00723	4	CCNB1/NEK2
GO	GO:0071902	positive regulation of protein serine/threonine kinase activity	5/65	229/18903	6.35	0.00114	0.0117	0.00723	5	CCNB1/INSR/
GO	GO:0019748	secondary metabolic process	3/65	60/18903	14.54	0.00115	0.0117	0.00728	3	TRPC1/AKR1
GO	GO:0043467	regulation of generation of precursor metabolites and energy	4/65	134/18903	8.68	0.00117	0.0118	0.00729	4	CDK1/CCNB1
GO	GO:0051299	centrosome separation	2/65	15/18903	38.78	0.00119	0.0118	0.00729	2	NEK2/AURKA
GO	GO:0061744	motor behavior	2/65	15/18903	38.78	0.00119	0.0118	0.00729	2	DPP4/SLC1A
GO	GO:0071380	cellular response to prostaglandin E stimulus	2/65	15/18903	38.78	0.00119	0.0118	0.00729	2	PTGER2/PTG
GO	GO:1902563	regulation of neutrophil activation	2/65	15/18903	38.78	0.00119	0.0118	0.00729	2	PLA2G2A/ITG
GO	GO:1904251	regulation of bile acid metabolic process	2/65	15/18903	38.78	0.00119	0.0118	0.00729	2	NR1H4/KIT
GO	GO:2001028	positive regulation of endothelial cell chemotaxis	2/65	15/18903	38.78	0.00119	0.0118	0.00729	2	MET/FGFR1
GO	GO:0007098	centrosome cycle	4/65	135/18903	8.62	0.0012	0.0119	0.00736	4	CDK1/NEK2/
GO	GO:0060135	maternal process involved in female pregnancy	3/65	61/18903	14.3	0.00121	0.0119	0.00736	3	ESR1/PGR/C
GO	GO:1902893	regulation of miRNA transcription	3/65	61/18903	14.3	0.00121	0.0119	0.00736	3	NR3C1/TP53
GO	GO:0008406	gonad development	5/65	233/18903	6.24	0.00123	0.0119	0.0074	5	ESR1/PGR/IN
GO	GO:0070661	leukocyte proliferation	6/65	348/18903	5.01	0.00124	0.0119	0.0074	6	ARG2/JAK3/
GO	GO:0010565	regulation of cellular ketone metabolic process	4/65	136/18903	8.55	0.00124	0.0119	0.0074	4	NR4A3/NR1H
GO	GO:0043401	steroid hormone mediated signaling pathway	4/65	136/18903	8.55	0.00124	0.0119	0.0074	4	ESR1/PGR/N
GO	GO:0022617	extracellular matrix disassembly	3/65	62/18903	14.07	0.00127	0.0122	0.00755	3	MMP7/ADAM
GO	GO:0061614	miRNA transcription	3/65	62/18903	14.07	0.00127	0.0122	0.00755	3	NR3C1/TP53
GO	GO:2000116	regulation of cysteine-type endopeptidase activity	5/65	235/18903	6.19	0.00128	0.0122	0.00759	5	DAPK1/SLC1A
GO	GO:0000280	nuclear division	7/65	481/18903	4.23	0.00129	0.0122	0.0076	7	TOP2A/CCNB1/NEK2/AURKB/INSR/KAT2B/AURKA
GO	GO:0070542	response to fatty acid	3/65	63/18903	13.85	0.00133	0.0124	0.0077	3	NR1H4/AKR1
GO	GO:0002274	myeloid leukocyte activation	5/65	237/18903	6.14	0.00133	0.0124	0.0077	5	PLA2G2A/NR
GO	GO:0007586	digestion	4/65	139/18903	8.37	0.00134	0.0124	0.0077	4	PTGER3/AKR
GO	GO:0002730	regulation of dendritic cell cytokine production	2/65	16/18903	36.35	0.00135	0.0124	0.0077	2	JAK3/KIT
GO	GO:0006750	glutathione biosynthetic process	2/65	16/18903	36.35	0.00135	0.0124	0.0077	2	SLC1A2/SLC1
GO	GO:0034374	low-density lipoprotein particle remodeling	2/65	16/18903	36.35	0.00135	0.0124	0.0077	2	PLA2G2A/AG
GO	GO:0042448	progesterone metabolic process	2/65	16/18903	36.35	0.00135	0.0124	0.0077	2	AKR1C2/AKR
GO	GO:0044406	adhesion of symbiont to host	2/65	16/18903	36.35	0.00135	0.0124	0.0077	2	DPP4/ICAM1
GO	GO:0061469	regulation of type B pancreatic cell proliferation	2/65	16/18903	36.35	0.00135	0.0124	0.0077	2	NR4A3/NR4A
GO	GO:1903799	negative regulation of miRNA maturation	2/65	16/18903	36.35	0.00135	0.0124	0.0077	2	ESR1/TP53
GO	GO:0045137	development of primary sexual characteristics	5/65	238/18903	6.11	0.00136	0.0124	0.0077	5	ESR1/PGR/IN
GO	GO:0030595	leukocyte chemotaxis	5/65	240/18903	6.06	0.00141	0.0128	0.00794	5	SERPINE1/DP
GO	GO:0001889	liver development	4/65	141/18903	8.25	0.00141	0.0128	0.00794	4	ALDH1A2/TY
GO	GO:0007093	mitotic cell cycle checkpoint signaling	4/65	141/18903	8.25	0.00141	0.0128	0.00794	4	CDK1/CCNB1
GO	GO:0002703	regulation of leukocyte mediated immunity	5/65	241/18903	6.03	0.00143	0.0129	0.00803	5	JAK3/KIT/ITG
GO	GO:0000086	G2/M transition of mitotic cell cycle	4/65	142/18903	8.19	0.00145	0.013	0.00809	4	CDK1/CCNB1
GO	GO:0055123	digestive system development	4/65	143/18903	8.13	0.00149	0.0132	0.00822	4	ALDH1A2/TY
GO	GO:2001056	positive regulation of cysteine-type endopeptidase activity	4/65	143/18903	8.13	0.00149	0.0132	0.00822	4	DAPK1/SLC1A
GO	GO:0061008	hepaticobiliary system development	4/65	144/18903	8.08	0.00153	0.0132	0.00822	4	ALDH1A2/TY
GO	GO:0001991	regulation of systemic arterial blood pressure by circulatory renin-ang	2/65	17/18903	34.21	0.00153	0.0132	0.00822	2	ACE/ENPEP
GO	GO:0002371	dendritic cell cytokine production	2/65	17/18903	34.21	0.00153	0.0132	0.00822	2	JAK3/KIT
GO	GO:0016114	terpenoid biosynthetic process	2/65	17/18903	34.21	0.00153	0.0132	0.00822	2	ALDH1A2/AL
GO	GO:0031649	heat generation	2/65	17/18903	34.21	0.00153	0.0132	0.00822	2	PTGER3/CNR
GO	GO:0033189	response to vitamin A	2/65	17/18903	34.21	0.00153	0.0132	0.00822	2	ALDH1A2/TY
GO	GO:0098712	L-glutamate import across plasma membrane	2/65	17/18903	34.21	0.00153	0.0132	0.00822	2	SLC1A2/SLC1
GO	GO:1904355	positive regulation of telomere capping	2/65	17/18903	34.21	0.00153	0.0132	0.00822	2	NEK2/AURKE
GO	GO:1905288	vascular associated smooth muscle cell apoptotic process	2/65	17/18903	34.21	0.00153	0.0132	0.00822	2	PPARG/DNM
GO	GO:1905459	regulation of vascular associated smooth muscle cell apoptotic proces	2/65	17/18903	34.21	0.00153	0.0132	0.00822	2	PPARG/DNM
GO	GO:0043410	positive regulation of MAPK cascade	7/65	496/18903	4.1	0.00154	0.0132	0.00822	7	PLA2G2A/INS
GO	GO:0014065	phosphatidylinositol 3-kinase signaling	4/65	145/18903	8.02	0.00156	0.0134	0.00835	4	INSR/KIT/FLT
GO	GO:0045576	mast cell activation	3/65	67/18903	13.02	0.00159	0.0136	0.00844	3	NR4A3/CNR1
GO	GO:0051259	protein complex oligomerization	5/65	247/18903	5.89	0.0016	0.0136	0.00844	5	ALDH1A2/TK
GO	GO:0010631	epithelial cell migration	6/65	366/18903	4.77	0.0016	0.0136	0.00844	6	MET/DPP4/P
GO	GO:0005996	monosaccharide metabolic process	5/65	248/18903	5.86	0.00162	0.0137	0.00853	5	INSR/NR3C1/
GO	GO:0048863	stem cell differentiation	5/65	248/18903	5.86	0.00162	0.0137	0.00853	5	ALDH1A2/ES
GO	GO:0070997	neuron death	6/65	368/18903	4.74	0.00164	0.0138	0.00858	6	NR4A3/NR4A
GO	GO:0008584	male gonad development	4/65	147/18903	7.91	0.00165	0.0138	0.00858	4	ESR1/INSR/K
GO	GO:0090132	epithelium migration	6/65	369/18903	4.73	0.00166	0.0139	0.00865	6	MET/DPP4/P
GO	GO:0009636	response to toxic substance	5/65	250/18903	5.82	0.00168	0.014	0.00869	5	TYMS/CDK1/
GO	GO:0016485	protein processing	5/65	250/18903	5.82	0.00168	0.014	0.00869	5	MME/SERPIN
GO	GO:0046546	development of primary male sexual characteristics	4/65	148/18903	7.86	0.00169	0.014	0.00869	4	ESR1/INSR/K
GO	GO:0019184	nonribosomal peptide biosynthetic process	2/65	18/18903	32.31	0.00172	0.014	0.00872	2	SLC1A2/SLC1
GO	GO:0034501	protein localization to kinetochore	2/65	18/18903	32.31	0.00172	0.014	0.00872	2	CDK1/AURKE
GO	GO:0051770	positive regulation of nitric-oxide synthase biosynthetic process	2/65	18/18903	32.31	0.00172	0.014	0.00872	2	JAK3/NAMPT
GO	GO:1903083	protein localization to condensed chromosome	2/65	18/18903	32.31	0.00172	0.014	0.00872	2	CDK1/AURKE
GO	GO:0009411	response to UV	4/65	149/18903	7.81	0.00173	0.014	0.00872	4	MME/AURKE
GO	GO:0061180	mammary gland epithelium development	3/65	69/18903	12.64	0.00173	0.014	0.00872	3	ESR1/PGR/N
GO	GO:0015711	organic anion transport	6/65	372/18903	4.69	0.00173	0.014	0.00872	6	PLA2G2A/SL
GO	GO:0051098	regulation of binding	6/65	372/18903	4.69	0.00173	0.014	0.00872	6	NEK2/MET/A
GO	GO:0031023	microtubule organizing center organization	4/65	150/18903	7.76	0.00177	0.0143	0.00888	4	CDK1/NEK2/
GO	GO:0090130	tissue migration	6/65	374/18903	4.67	0.00178	0.0143	0.0089	6	MET/DPP4/P
GO	GO:0006081	cellular aldehyde metabolic process	3/65	70/18903	12.46	0.0018	0.0145	0.00898	3	ALDH1A2/AL
GO	GO:0051310	metaphase plate congression	3/65	71/18903	12.29	0.00188	0.015	0.00933	3	CCNB1/AURK
GO	GO:0006837	serotonin transport	2/65	19/18903	30.61	0.00192	0.0151	0.00938	2	CNR1/MAOB
GO	GO:0010744	positive regulation of macrophage derived foam cell differentiation	2/65	19/18903	30.61	0.00192	0.0151	0.00938	2	PLA2G2A/AG
GO	GO:0035813	regulation of renal sodium excretion	2/65	19/18903	30.61	0.00192	0.0151	0.00938	2	AGTR1/ACE
GO	GO:0070242	thymocyte apoptotic process	2/65	19/18903	30.61	0.00192	0.0151	0.00938	2	JAK3/TP53
GO	GO:2000774	positive regulation of cellular senescence	2/65	19/18903	30.61	0.00192	0.0151	0.00938	2	ARG2/TP53

GO	GO:0042742	defense response to bacterium	6/65	382/18903	4.57	0.00198	0.0156	0.00967	6	PLA2G2A/ARG2/NR1H4/ADAMTS5/SERPINE1/NOD1
GO	GO:0010675	regulation of cellular carbohydrate metabolic process	4/65	155/18903	7.5	0.002	0.0157	0.00972	4	INSR/NR3C1/
GO	GO:0071260	cellular response to mechanical stimulus	3/65	73/18903	11.95	0.00203	0.0159	0.00986	3	SLC2A1/PTG
GO	GO:0007189	adenylate cyclase-activating G protein-coupled receptor signaling pat	4/65	157/18903	7.41	0.00209	0.0163	0.0101	4	PTGER3/CNR
GO	GO:2000628	regulation of miRNA metabolic process	3/65	74/18903	11.79	0.00211	0.0164	0.0102	3	NR3C1/TP53
GO	GO:0060004	reflex	2/65	20/18903	29.08	0.00213	0.0165	0.0102	2	ALDH1A3/SL
GO	GO:0002834	regulation of response to tumor cell	2/65	21/18903	27.7	0.00234	0.0177	0.011	2	DAPK1/AHR
GO	GO:0016137	glycoside metabolic process	2/65	21/18903	27.7	0.00234	0.0177	0.011	2	AKR1C2/AKR
GO	GO:0035812	renal sodium excretion	2/65	21/18903	27.7	0.00234	0.0177	0.011	2	AGTR1/ACE
GO	GO:0038083	peptidyl-tyrosine autophosphorylation	2/65	21/18903	27.7	0.00234	0.0177	0.011	2	INSR/ACE
GO	GO:0051315	attachment of mitotic spindle microtubules to kinetochore	2/65	21/18903	27.7	0.00234	0.0177	0.011	2	AURKB/KAT2
GO	GO:0090231	regulation of spindle checkpoint	2/65	21/18903	27.7	0.00234	0.0177	0.011	2	CCNB1/AUR
GO	GO:0090266	regulation of mitotic cell cycle spindle assembly checkpoint	2/65	21/18903	27.7	0.00234	0.0177	0.011	2	CCNB1/AUR
GO	GO:1903206	negative regulation of hydrogen peroxide-induced cell death	2/65	21/18903	27.7	0.00234	0.0177	0.011	2	NR4A3/MET
GO	GO:1903504	regulation of mitotic spindle checkpoint	2/65	21/18903	27.7	0.00234	0.0177	0.011	2	CCNB1/AUR
GO	GO:0006919	activation of cysteine-type endopeptidase activity involved in apoptot	3/65	77/18903	11.33	0.00237	0.0178	0.011	3	SLC1A1/PPA
GO	GO:0009123	nucleoside monophosphate metabolic process	3/65	77/18903	11.33	0.00237	0.0178	0.011	3	TYMS/TK1/N
GO	GO:1902850	microtubule cytoskeleton organization involved in mitosis	4/65	163/18903	7.14	0.0024	0.018	0.0111	4	CCNB1/NEK2
GO	GO:0031348	negative regulation of defense response	5/65	272/18903	5.35	0.00243	0.0182	0.0113	5	ARG2/NR1H
GO	GO:0051100	negative regulation of binding	4/65	164/18903	7.09	0.00245	0.0183	0.0113	4	NEK2/AURK
GO	GO:0006631	fatty acid metabolic process	6/65	400/18903	4.36	0.00249	0.0185	0.0115	6	NR4A3/CNR1
GO	GO:0002720	positive regulation of cytokine production involved in immune respon	3/65	79/18903	11.04	0.00255	0.0187	0.0116	3	NR4A3/KIT/
GO	GO:0043299	leukocyte degranulation	3/65	79/18903	11.04	0.00255	0.0187	0.0116	3	NR4A3/KIT/
GO	GO:0002070	epithelial cell maturation	2/65	22/18903	26.44	0.00257	0.0187	0.0116	2	TYMS/PGR
GO	GO:0044062	regulation of excretion	2/65	22/18903	26.44	0.00257	0.0187	0.0116	2	AGTR1/ACE
GO	GO:0051767	nitric-oxide synthase biosynthetic process	2/65	22/18903	26.44	0.00257	0.0187	0.0116	2	JAK3/NAMP
GO	GO:0051769	regulation of nitric-oxide synthase biosynthetic process	2/65	22/18903	26.44	0.00257	0.0187	0.0116	2	JAK3/NAMP
GO	GO:0071404	cellular response to low-density lipoprotein particle stimulus	2/65	22/18903	26.44	0.00257	0.0187	0.0116	2	PPARG/ITGB
GO	GO:0033555	multicellular organismal response to stress	3/65	81/18903	10.77	0.00273	0.0199	0.0123	3	NR4A2/DPP4
GO	GO:0043542	endothelial cell migration	5/65	281/18903	5.17	0.0028	0.0202	0.0125	5	MET/DPP4/P
GO	GO:0014821	phasic smooth muscle contraction	2/65	23/18903	25.29	0.00281	0.0202	0.0125	2	PTGER3/KIT
GO	GO:0034695	response to prostaglandin E	2/65	23/18903	25.29	0.00281	0.0202	0.0125	2	PTGER2/PTG
GO	GO:0060444	branching involved in mammary gland duct morphogenesis	2/65	23/18903	25.29	0.00281	0.0202	0.0125	2	ESR1/PGR
GO	GO:1901032	negative regulation of response to reactive oxygen species	2/65	23/18903	25.29	0.00281	0.0202	0.0125	2	NR4A3/MET
GO	GO:0031016	pancreas development	3/65	82/18903	10.64	0.00283	0.0202	0.0125	3	ALDH1A2/M
GO	GO:0051303	establishment of chromosome localization	3/65	82/18903	10.64	0.00283	0.0202	0.0125	3	CCNB1/AUR
GO	GO:0046661	male sex differentiation	4/65	171/18903	6.8	0.00285	0.0203	0.0126	4	ESR1/INSR/K
GO	GO:0097006	regulation of plasma lipoprotein particle levels	3/65	84/18903	10.39	0.00303	0.0215	0.0133	3	PLA2G2A/NR
GO	GO:0010950	positive regulation of endopeptidase activity	4/65	174/18903	6.69	0.00303	0.0215	0.0133	4	DAPK1/SLC1
GO	GO:0070233	negative regulation of T cell apoptotic process	2/65	24/18903	24.23	0.00306	0.0215	0.0134	2	ARG2/JAK3
GO	GO:2001026	regulation of endothelial cell chemotaxis	2/65	24/18903	24.23	0.00306	0.0215	0.0134	2	MET/FGFR1
GO	GO:0051048	negative regulation of secretion	4/65	175/18903	6.65	0.0031	0.0217	0.0135	4	PTGER3/CNR
GO	GO:0007548	sex differentiation	5/65	288/18903	5.05	0.00311	0.0218	0.0135	5	ESR1/PGR/IN
GO	GO:0008625	extrinsic apoptotic signaling pathway via death domain receptors	3/65	85/18903	10.26	0.00314	0.0219	0.0136	3	DAPK1/SERP
GO	GO:0046942	carboxylic acid transport	5/65	290/18903	5.01	0.0032	0.0223	0.0138	5	PLA2G2A/SL
GO	GO:0006970	response to osmotic stress	3/65	86/18903	10.14	0.00324	0.0225	0.0139	3	MYLK/TP53/
GO	GO:0033273	response to vitamin	3/65	86/18903	10.14	0.00324	0.0225	0.0139	3	ALDH1A2/TY
GO	GO:0048015	phosphatidylinositol-mediated signaling	4/65	178/18903	6.54	0.00329	0.0226	0.014	4	INSR/KIT/FLT
GO	GO:0015850	organic hydroxy compound transport	5/65	292/18903	4.98	0.0033	0.0226	0.014	5	CNR1/AGTR1
GO	GO:0003071	renal system process involved in regulation of systemic arterial blood	2/65	25/18903	23.27	0.00332	0.0226	0.014	2	HSD11B2/AG
GO	GO:0008211	glucocorticoid metabolic process	2/65	25/18903	23.27	0.00332	0.0226	0.014	2	HSD11B2/NR
GO	GO:0009162	deoxyribonucleoside monophosphate metabolic process	2/65	25/18903	23.27	0.00332	0.0226	0.014	2	TYMS/TK1
GO	GO:1905063	regulation of vascular associated smooth muscle cell differentiation	2/65	25/18903	23.27	0.00332	0.0226	0.014	2	KIT/DNMT1
GO	GO:1905563	negative regulation of vascular endothelial cell proliferation	2/65	25/18903	23.27	0.00332	0.0226	0.014	2	PPARG/FLT1
GO	GO:0001523	retinoid metabolic process	3/65	87/18903	10.03	0.00335	0.0226	0.014	3	ALDH1A2/AL
GO	GO:0010660	regulation of muscle cell apoptotic process	3/65	87/18903	10.03	0.00335	0.0226	0.014	3	TP53/PPARG
GO	GO:0014910	regulation of smooth muscle cell migration	3/65	87/18903	10.03	0.00335	0.0226	0.014	3	NR4A3/SERP
GO	GO:0048145	regulation of fibroblast proliferation	3/65	87/18903	10.03	0.00335	0.0226	0.014	3	ESR1/CCNB1
GO	GO:0052548	regulation of endopeptidase activity	6/65	428/18903	4.08	0.00348	0.0234	0.0145	6	DAPK1/SERP
GO	GO:0048017	inositol lipid-mediated signaling	4/65	182/18903	6.39	0.00356	0.0238	0.0147	4	INSR/KIT/FLT
GO	GO:0034644	cellular response to UV	3/65	89/18903	9.8	0.00357	0.0238	0.0147	3	MME/AURK
GO	GO:0050000	chromosome localization	3/65	89/18903	9.8	0.00357	0.0238	0.0147	3	CCNB1/AUR
GO	GO:0060333	interferon-gamma-mediated signaling pathway	2/65	26/18903	22.37	0.00359	0.0238	0.0147	2	TP53/PPARG
GO	GO:1903798	regulation of miRNA maturation	2/65	26/18903	22.37	0.00359	0.0238	0.0147	2	ESR1/TP53
GO	GO:1904353	regulation of telomere capping	2/65	26/18903	22.37	0.00359	0.0238	0.0147	2	NEK2/AURK
GO	GO:0071466	cellular response to xenobiotic stimulus	4/65	183/18903	6.36	0.00363	0.024	0.0149	4	TP53/AKR1C
GO	GO:0010586	miRNA metabolic process	3/65	90/18903	9.69	0.00368	0.0241	0.015	3	NR3C1/TP53
GO	GO:0016101	diterpenoid metabolic process	3/65	90/18903	9.69	0.00368	0.0241	0.015	3	ALDH1A2/AL
GO	GO:0045995	regulation of embryonic development	3/65	90/18903	9.69	0.00368	0.0241	0.015	3	CDK1/INSR/
GO	GO:0006869	lipid transport	6/65	433/18903	4.03	0.00369	0.0241	0.015	6	PLA2G2A/AG
GO	GO:0050728	negative regulation of inflammatory response	4/65	184/18903	6.32	0.0037	0.0242	0.015	4	NR1H4/PPA
GO	GO:1901216	positive regulation of neuron death	3/65	91/18903	9.59	0.0038	0.0247	0.0154	3	NR3C1/TP53
GO	GO:0000075	cell cycle checkpoint signaling	4/65	186/18903	6.25	0.00385	0.0248	0.0154	4	CDK1/CCNB1
GO	GO:0006109	regulation of carbohydrate metabolic process	4/65	186/18903	6.25	0.00385	0.0248	0.0154	4	INSR/NR3C1/
GO	GO:0030325	adrenal gland development	2/65	27/18903	21.54	0.00387	0.0248	0.0154	2	INSR/NR3C1
GO	GO:0032682	negative regulation of chemokine production	2/65	27/18903	21.54	0.00387	0.0248	0.0154	2	ARG2/NR1H
GO	GO:0044342	type B pancreatic cell proliferation	2/65	27/18903	21.54	0.00387	0.0248	0.0154	2	NR4A3/NR4
GO	GO:0060259	regulation of feeding behavior	2/65	27/18903	21.54	0.00387	0.0248	0.0154	2	NR4A3/CNR1
GO	GO:0010657	muscle cell apoptotic process	3/65	92/18903	9.48	0.00392	0.0251	0.0156	3	TP53/PPARG
GO	GO:0002700	regulation of production of molecular mediator of immune response	4/65	187/18903	6.22	0.00392	0.0251	0.0156	4	NR4A3/JAK3
GO	GO:0006006	glucose metabolic process	4/65	188/18903	6.19	0.004	0.0255	0.0158	4	INSR/NR3C1/
GO	GO:0048608	reproductive structure development	5/65	306/18903	4.75	0.00402	0.0256	0.0159	5	ESR1/PGR/IN
GO	GO:0070301	cellular response to hydrogen peroxide	3/65	93/18903	9.38	0.00404	0.0256	0.0159	3	CDK1/NR4A3
GO	GO:1904705	regulation of vascular associated smooth muscle cell proliferation	3/65	93/18903	9.38	0.00404	0.0256	0.0159	3	NR4A3/PPA
GO	GO:0051896	regulation of protein kinase B signaling	4/65	189/18903	6.15	0.00408	0.0257	0.0159	4	MET/INSR/A
GO	GO:0044403	biological process involved in symbiotic interaction	5/65	307/18903	4.74	0.00408	0.0257	0.0159	5	CDK1/DPP4/

GO	GO:0050777	negative regulation of immune response	4/65	190/18903	6.12	0.00415	0.0257	0.0159	4	ARG2/JAK3/PPARG/AHR
GO	GO:0051260	protein homooligomerization	4/65	190/18903	6.12	0.00415	0.0257	0.0159	4	ALDH1A2/TK
GO	GO:0002675	positive regulation of acute inflammatory response	2/65	28/18903	20.77	0.00415	0.0257	0.0159	2	PTGER3/CNR
GO	GO:0010971	positive regulation of G2/M transition of mitotic cell cycle	2/65	28/18903	20.77	0.00415	0.0257	0.0159	2	CDK1/CCNB1
GO	GO:0014072	response to isoquinoline alkaloid	2/65	28/18903	20.77	0.00415	0.0257	0.0159	2	CNR1/SLC1A
GO	GO:0043278	response to morphine	2/65	28/18903	20.77	0.00415	0.0257	0.0159	2	CNR1/SLC1A
GO	GO:0070920	regulation of production of small RNA involved in gene silencing by RN	2/65	28/18903	20.77	0.00415	0.0257	0.0159	2	ESR1/TP53
GO	GO:1902883	negative regulation of response to oxidative stress	2/65	28/18903	20.77	0.00415	0.0257	0.0159	2	NR4A3/MET
GO	GO:0061458	reproductive system development	5/65	310/18903	4.69	0.00425	0.0262	0.0163	5	ESR1/PGR/IN
GO	GO:0014909	smooth muscle cell migration	3/65	95/18903	9.18	0.00429	0.0263	0.0163	3	NR4A3/SERP
GO	GO:1990874	vascular associated smooth muscle cell proliferation	3/65	95/18903	9.18	0.00429	0.0263	0.0163	3	NR4A3/PPAR
GO	GO:0032943	mononuclear cell proliferation	5/65	312/18903	4.66	0.00437	0.0267	0.0166	5	ARG2/JAK3/T
GO	GO:0002275	myeloid cell activation involved in immune response	3/65	96/18903	9.09	0.00442	0.0267	0.0166	3	NR4A3/KIT/I
GO	GO:0019217	regulation of fatty acid metabolic process	3/65	96/18903	9.09	0.00442	0.0267	0.0166	3	NR4A3/CNR1
GO	GO:0032091	negative regulation of protein binding	3/65	96/18903	9.09	0.00442	0.0267	0.0166	3	AURKB/AURK
GO	GO:0015740	C4-dicarboxylate transport	2/65	29/18903	20.06	0.00445	0.0267	0.0166	2	SLC1A2/SLC1
GO	GO:0015813	L-glutamate transmembrane transport	2/65	29/18903	20.06	0.00445	0.0267	0.0166	2	SLC1A2/SLC1
GO	GO:0051938	L-glutamate import	2/65	29/18903	20.06	0.00445	0.0267	0.0166	2	SLC1A2/SLC1
GO	GO:0097066	response to thyroid hormone	2/65	29/18903	20.06	0.00445	0.0267	0.0166	2	KIT/ACE
GO	GO:0097254	renal tubular secretion	2/65	29/18903	20.06	0.00445	0.0267	0.0166	2	AGTR1/ACE
GO	GO:0097421	liver regeneration	2/65	29/18903	20.06	0.00445	0.0267	0.0166	2	TYMS/AURK
GO	GO:0010952	positive regulation of peptidase activity	4/65	194/18903	6	0.00447	0.0267	0.0166	4	DAPK1/SLC1
GO	GO:1901991	negative regulation of mitotic cell cycle phase transition	4/65	194/18903	6	0.00447	0.0267	0.0166	4	CDK1/CCNB1
GO	GO:0009314	response to radiation	6/65	452/18903	3.86	0.00454	0.027	0.0168	6	MME/AURKB/SLC1A2/FEN1/TP53/KIT
GO	GO:2001251	negative regulation of chromosome organization	3/65	97/18903	8.99	0.00455	0.027	0.0168	3	TOP2A/CCNE
GO	GO:0007051	spindle organization	4/65	195/18903	5.97	0.00455	0.027	0.0168	4	CCNB1/NEK2
GO	GO:0007626	locomotory behavior	4/65	197/18903	5.9	0.00472	0.0279	0.0173	4	NR4A2/DPP4
GO	GO:0008209	androgen metabolic process	2/65	30/18903	19.39	0.00476	0.0279	0.0173	2	ESR1/HSD17
GO	GO:0008299	isoprenoid biosynthetic process	2/65	30/18903	19.39	0.00476	0.0279	0.0173	2	ALDH1A2/AL
GO	GO:0090140	regulation of mitochondrial fission	2/65	30/18903	19.39	0.00476	0.0279	0.0173	2	PPARG/AURK
GO	GO:1903205	regulation of hydrogen peroxide-induced cell death	2/65	30/18903	19.39	0.00476	0.0279	0.0173	2	NR4A3/MET
GO	GO:0043279	response to alkaloid	3/65	99/18903	8.81	0.00481	0.0282	0.0175	3	SLC1A2/CNR
GO	GO:0052547	regulation of peptidase activity	6/65	459/18903	3.8	0.00489	0.0286	0.0177	6	DAPK1/SERPINE1/SLC1A1/PPARG/NR4A1/NOD1
GO	GO:0036473	cell death in response to oxidative stress	3/65	100/18903	8.72	0.00495	0.0289	0.0179	3	NR4A3/MET
GO	GO:0032148	activation of protein kinase B activity	2/65	31/18903	18.76	0.00508	0.0293	0.0182	2	INSR/SLC1A1
GO	GO:0048384	retinoic acid receptor signaling pathway	2/65	31/18903	18.76	0.00508	0.0293	0.0182	2	ALDH1A2/PP
GO	GO:1902042	negative regulation of extrinsic apoptotic signaling pathway via death	2/65	31/18903	18.76	0.00508	0.0293	0.0182	2	SERPINE1/IC
GO	GO:1902751	positive regulation of cell cycle G2/M phase transition	2/65	31/18903	18.76	0.00508	0.0293	0.0182	2	CDK1/CCNB1
GO	GO:0006721	terpenoid metabolic process	3/65	101/18903	8.64	0.00509	0.0293	0.0182	3	ALDH1A2/AL
GO	GO:0019218	regulation of steroid metabolic process	3/65	102/18903	8.55	0.00523	0.0301	0.0187	3	NR1H4/NR3
GO	GO:0000070	mitotic sister chromatid segregation	4/65	204/18903	5.7	0.00534	0.0305	0.0189	4	CCNB1/NEK2
GO	GO:0000302	response to reactive oxygen species	4/65	204/18903	5.7	0.00534	0.0305	0.0189	4	CDK1/NR4A3
GO	GO:0019233	sensory perception of pain	3/65	103/18903	8.47	0.00537	0.0305	0.0189	3	MME/CNR1/
GO	GO:0043255	regulation of carbohydrate biosynthetic process	3/65	103/18903	8.47	0.00537	0.0305	0.0189	3	INSR/NR3C1
GO	GO:0034390	smooth muscle cell apoptotic process	2/65	32/18903	18.18	0.0054	0.0305	0.0189	2	PPARG/DNM
GO	GO:0034391	regulation of smooth muscle cell apoptotic process	2/65	32/18903	18.18	0.0054	0.0305	0.0189	2	PPARG/DNM
GO	GO:0043552	positive regulation of phosphatidylinositol 3-kinase activity	2/65	32/18903	18.18	0.0054	0.0305	0.0189	2	KIT/FLT1
GO	GO:0050482	arachidonic acid secretion	2/65	32/18903	18.18	0.0054	0.0305	0.0189	2	PLA2G2A/AC
GO	GO:1903963	arachidonate transport	2/65	32/18903	18.18	0.0054	0.0305	0.0189	2	PLA2G2A/AC
GO	GO:0023061	signal release	6/65	470/18903	3.71	0.00548	0.0309	0.0192	6	NR1H4/CNR1
GO	GO:0002285	lymphocyte activation involved in immune response	4/65	207/18903	5.62	0.00562	0.0315	0.0196	4	JAK3/TP53/P
GO	GO:0048167	regulation of synaptic plasticity	4/65	207/18903	5.62	0.00562	0.0315	0.0196	4	MME/SLC1A
GO	GO:0007200	phospholipase C-activating G protein-coupled receptor signaling path	3/65	105/18903	8.31	0.00567	0.0317	0.0196	3	ESR1/PTGER
GO	GO:1901796	regulation of signal transduction by p53 class mediator	3/65	105/18903	8.31	0.00567	0.0317	0.0196	3	AURKB/TP53
GO	GO:0032869	cellular response to insulin stimulus	4/65	208/18903	5.59	0.00571	0.0317	0.0197	4	NR1H4/INSR
GO	GO:0015980	energy derivation by oxidation of organic compounds	5/65	333/18903	4.37	0.00574	0.0317	0.0197	5	CDK1/CCNB1
GO	GO:0007143	female meiotic nuclear division	2/65	33/18903	17.63	0.00574	0.0317	0.0197	2	TOP2A/AURK
GO	GO:0036475	neuron death in response to oxidative stress	2/65	33/18903	17.63	0.00574	0.0317	0.0197	2	NR4A3/SLC1
GO	GO:0070633	transepithelial transport	2/65	33/18903	17.63	0.00574	0.0317	0.0197	2	SLC1A2/SLC1
GO	GO:0098801	regulation of renal system process	2/65	33/18903	17.63	0.00574	0.0317	0.0197	2	AGTR1/ACE
GO	GO:0033045	regulation of sister chromatid segregation	3/65	107/18903	8.15	0.00597	0.0329	0.0204	3	CCNB1/AURK
GO	GO:0009612	response to mechanical stimulus	4/65	211/18903	5.51	0.00601	0.0329	0.0204	4	SLC2A1/KIT/
GO	GO:0032212	positive regulation of telomere maintenance via telomerase	2/65	34/18903	17.11	0.00608	0.0329	0.0204	2	NEK2/AURKE
GO	GO:0036474	cell death in response to hydrogen peroxide	2/65	34/18903	17.11	0.00608	0.0329	0.0204	2	NR4A3/MET
GO	GO:0042573	retinoic acid metabolic process	2/65	34/18903	17.11	0.00608	0.0329	0.0204	2	ALDH1A2/AL
GO	GO:0051973	positive regulation of telomerase activity	2/65	34/18903	17.11	0.00608	0.0329	0.0204	2	NEK2/AURKE
GO	GO:0055094	response to lipoprotein particle	2/65	34/18903	17.11	0.00608	0.0329	0.0204	2	PPARG/ITGB
GO	GO:0090313	regulation of protein targeting to membrane	2/65	34/18903	17.11	0.00608	0.0329	0.0204	2	SLC1A1/ITGB
GO	GO:0006836	neurotransmitter transport	4/65	212/18903	5.49	0.00611	0.0329	0.0204	4	SLC1A2/CNR
GO	GO:0007631	feeding behavior	3/65	108/18903	8.08	0.00613	0.0329	0.0204	3	NR4A3/CNR1
GO	GO:0014812	muscle cell migration	3/65	108/18903	8.08	0.00613	0.0329	0.0204	3	NR4A3/SERP
GO	GO:0019882	antigen processing and presentation	3/65	108/18903	8.08	0.00613	0.0329	0.0204	3	ACE/ICAM1/
GO	GO:0032526	response to retinoic acid	3/65	108/18903	8.08	0.00613	0.0329	0.0204	3	ALDH1A2/HS
GO	GO:0051341	regulation of oxidoreductase activity	3/65	108/18903	8.08	0.00613	0.0329	0.0204	3	CNR1/AGTR1
GO	GO:0010876	lipid localization	6/65	483/18903	3.61	0.00624	0.0334	0.0207	6	PLA2G2A/AG
GO	GO:0070371	ERK1 and ERK2 cascade	5/65	340/18903	4.28	0.00625	0.0334	0.0207	5	PLA2G2A/CD
GO	GO:0001819	positive regulation of cytokine production	6/65	486/18903	3.59	0.00643	0.034	0.0211	6	NR4A3/NR1H
GO	GO:0002446	neutrophil mediated immunity	2/65	35/18903	16.62	0.00644	0.034	0.0211	2	ITGB2/ACE
GO	GO:0010661	positive regulation of muscle cell apoptotic process	2/65	35/18903	16.62	0.00644	0.034	0.0211	2	TP53/PPARG
GO	GO:0060218	hematopoietic stem cell differentiation	2/65	35/18903	16.62	0.00644	0.034	0.0211	2	TP53/ACE
GO	GO:2000352	negative regulation of endothelial cell apoptotic process	2/65	35/18903	16.62	0.00644	0.034	0.0211	2	SERPINE1/IC
GO	GO:2000515	negative regulation of CD4-positive, alpha-beta T cell activation	2/65	35/18903	16.62	0.00644	0.034	0.0211	2	ARG2/JAK3
GO	GO:1904035	regulation of epithelial cell apoptotic process	3/65	110/18903	7.93	0.00645	0.034	0.0211	3	ESR1/SERPIN
GO	GO:0043491	protein kinase B signaling	4/65	218/18903	5.34	0.00673	0.0353	0.0219	4	MET/INSR/A
GO	GO:0043523	regulation of neuron apoptotic process	4/65	218/18903	5.34	0.00673	0.0353	0.0219	4	NR4A2/SLC1
GO	GO:0046320	regulation of fatty acid oxidation	2/65	36/18903	16.16	0.0068	0.0355	0.0221	2	NR4A3/CNR1

GO	GO:0090218	positive regulation of lipid kinase activity	2/65	36/18903	16.16	0.0068	0.0355	0.0221	2KIT/FLT1
GO	GO:0001667	ameboidal-type cell migration	6/65	492/18903	3.55	0.00681	0.0355	0.0221	6MET/DPP4/PPARG/KIT/NR4A1/FGFR1
GO	GO:0001505	regulation of neurotransmitter levels	4/65	219/18903	5.31	0.00684	0.0356	0.0221	4SLC1A2/CNR
GO	GO:0006939	smooth muscle contraction	3/65	113/18903	7.72	0.00694	0.0361	0.0224	3MYLK/PTGER
GO	GO:0019221	cytokine-mediated signaling pathway	6/65	496/18903	3.52	0.00708	0.0364	0.0226	6NR1H4/JAK3
GO	GO:0044344	cellular response to fibroblast growth factor stimulus	3/65	114/18903	7.65	0.00711	0.0364	0.0226	3NR4A1/FGFR
GO	GO:0006936	muscle contraction	5/65	351/18903	4.14	0.00713	0.0364	0.0226	5MYLK/ARG2/
GO	GO:0001662	behavioral fear response	2/65	37/18903	15.72	0.00718	0.0364	0.0226	2DPP4/SLC1A
GO	GO:0007588	excretion	2/65	37/18903	15.72	0.00718	0.0364	0.0226	2AGTR1/ACE
GO	GO:0008207	C21-steroid hormone metabolic process	2/65	37/18903	15.72	0.00718	0.0364	0.0226	2AKR1C2/AKR
GO	GO:0031076	embryonic camera-type eye development	2/65	37/18903	15.72	0.00718	0.0364	0.0226	2ALDH1A2/AL
GO	GO:0046326	positive regulation of glucose import	2/65	37/18903	15.72	0.00718	0.0364	0.0226	2SLC1A2/INSR
GO	GO:0046473	phosphatidic acid metabolic process	2/65	37/18903	15.72	0.00718	0.0364	0.0226	2PLA2G2A/NR
GO	GO:0051385	response to mineralocorticoid	2/65	37/18903	15.72	0.00718	0.0364	0.0226	2ACE/MAOB
GO	GO:0071312	cellular response to alkaloid	2/65	37/18903	15.72	0.00718	0.0364	0.0226	2SLC1A2/SLC1
GO	GO:0071402	cellular response to lipoprotein particle stimulus	2/65	37/18903	15.72	0.00718	0.0364	0.0226	2PPARG/ITGB
GO	GO:1904358	positive regulation of telomere maintenance via telomere lengthening	2/65	37/18903	15.72	0.00718	0.0364	0.0226	2NEK2/AURKE
GO	GO:0001938	positive regulation of endothelial cell proliferation	3/65	115/18903	7.59	0.00729	0.0366	0.0227	3AGTR1/NR4
GO	GO:0033559	unsaturated fatty acid metabolic process	3/65	115/18903	7.59	0.00729	0.0366	0.0227	3AKR1C2/AKR
GO	GO:0034502	protein localization to chromosome	3/65	115/18903	7.59	0.00729	0.0366	0.0227	3ESR1/CDK1/
GO	GO:0035710	CD4-positive, alpha-beta T cell activation	3/65	115/18903	7.59	0.00729	0.0366	0.0227	3ARG2/JAK3/
GO	GO:1904892	regulation of receptor signaling pathway via STAT	3/65	115/18903	7.59	0.00729	0.0366	0.0227	3JAK3/PPARG
GO	GO:1901990	regulation of mitotic cell cycle phase transition	5/65	354/18903	4.11	0.00738	0.0369	0.0229	5CDK1/CCNB1
GO	GO:0050679	positive regulation of epithelial cell proliferation	4/65	224/18903	5.19	0.00739	0.0369	0.0229	4NR4A3/AGT
GO	GO:0097191	extrinsic apoptotic signaling pathway	4/65	224/18903	5.19	0.00739	0.0369	0.0229	4DAPK1/SERP
GO	GO:0002065	columnar/cuboidal epithelial cell differentiation	3/65	116/18903	7.52	0.00746	0.0371	0.023	3TYMS/PGR/S
GO	GO:0002832	negative regulation of response to biotic stimulus	3/65	116/18903	7.52	0.00746	0.0371	0.023	3ARG2/PPARG
GO	GO:0030518	intracellular steroid hormone receptor signaling pathway	3/65	116/18903	7.52	0.00746	0.0371	0.023	3ESR1/PGR/N
GO	GO:0002209	behavioral defense response	2/65	38/18903	15.31	0.00756	0.0375	0.0232	2DPP4/SLC1A
GO	GO:0006805	xenobiotic metabolic process	3/65	118/18903	7.39	0.00782	0.0385	0.0239	3AKR1C1/EPH
GO	GO:0043200	response to amino acid	3/65	118/18903	7.39	0.00782	0.0385	0.0239	3SLC1A2/DNM
GO	GO:0071346	cellular response to interferon-gamma	3/65	118/18903	7.39	0.00782	0.0385	0.0239	3DAPK1/TP53
GO	GO:0010038	response to metal ion	5/65	360/18903	4.04	0.00791	0.0389	0.0242	5CDK1/SLC1A
GO	GO:0032570	response to progesterone	2/65	39/18903	14.91	0.00795	0.039	0.0242	2TYMS/PTGER
GO	GO:1901661	quinone metabolic process	2/65	39/18903	14.91	0.00795	0.039	0.0242	2AKR1C2/AKR
GO	GO:0019318	hexose metabolic process	4/65	229/18903	5.08	0.00798	0.039	0.0242	4INSR/NR3C1/
GO	GO:0002831	regulation of response to biotic stimulus	5/65	361/18903	4.03	0.008	0.039	0.0242	5ARG2/DAPK1
GO	GO:0015718	monocarboxylic acid transport	3/65	119/18903	7.33	0.008	0.039	0.0242	3PLA2G2A/AK
GO	GO:0050920	regulation of chemotaxis	4/65	230/18903	5.06	0.0081	0.0394	0.0245	4MET/SERPIN
GO	GO:0071774	response to fibroblast growth factor	3/65	120/18903	7.27	0.00819	0.0398	0.0247	3NR4A1/FGFR
GO	GO:0002685	regulation of leukocyte migration	4/65	231/18903	5.04	0.00822	0.0398	0.0247	4SERPINE1/D
GO	GO:0031669	cellular response to nutrient levels	4/65	231/18903	5.04	0.00822	0.0398	0.0247	4NR1H4/TP53
GO	GO:0000266	mitochondrial fission	2/65	40/18903	14.54	0.00835	0.04	0.0248	2PPARG/AUR
GO	GO:0016233	telomere capping	2/65	40/18903	14.54	0.00835	0.04	0.0248	2NEK2/AURKE
GO	GO:0035886	vascular associated smooth muscle cell differentiation	2/65	40/18903	14.54	0.00835	0.04	0.0248	2KIT/DNMT1
GO	GO:0045923	positive regulation of fatty acid metabolic process	2/65	40/18903	14.54	0.00835	0.04	0.0248	2NR4A3/PPAR
GO	GO:0006720	isoprenoid metabolic process	3/65	121/18903	7.21	0.00837	0.04	0.0248	3ALDH1A2/AL
GO	GO:0032355	response to estradiol	3/65	121/18903	7.21	0.00837	0.04	0.0248	3ALDH1A2/ES
GO	GO:2000278	regulation of DNA biosynthetic process	3/65	121/18903	7.21	0.00837	0.04	0.0248	3NEK2/AURKE
GO	GO:0060348	bone development	4/65	233/18903	4.99	0.00847	0.0404	0.0251	4TP53/KIT/PT
GO	GO:0046883	regulation of hormone secretion	4/65	234/18903	4.97	0.00859	0.0409	0.0254	4NR1H4/CNR1
GO	GO:0002691	regulation of cellular extravasation	2/65	41/18903	14.19	0.00876	0.0414	0.0257	2PTGER4/ICA
GO	GO:0042119	neutrophil activation	2/65	41/18903	14.19	0.00876	0.0414	0.0257	2PLA2G2A/IT
GO	GO:1901031	regulation of response to reactive oxygen species	2/65	41/18903	14.19	0.00876	0.0414	0.0257	2NR4A3/MET
GO	GO:0022612	gland morphogenesis	3/65	123/18903	7.09	0.00876	0.0414	0.0257	3ESR1/PGR/N
GO	GO:0071482	cellular response to light stimulus	3/65	123/18903	7.09	0.00876	0.0414	0.0257	3MME/AURKE
GO	GO:0034764	positive regulation of transmembrane transport	4/65	237/18903	4.91	0.00898	0.0423	0.0263	4NR4A3/SLC1
GO	GO:0045862	positive regulation of proteolysis	5/65	372/18903	3.91	0.00904	0.0425	0.0264	5DAPK1/SLC1
GO	GO:0003014	renal system process	3/65	125/18903	6.98	0.00915	0.0429	0.0266	3HSD11B2/AG
GO	GO:0010907	positive regulation of glucose metabolic process	2/65	42/18903	13.85	0.00917	0.0429	0.0266	2INSR/KAT2B
GO	GO:0031670	cellular response to nutrient	2/65	42/18903	13.85	0.00917	0.0429	0.0266	2NR1H4/COL1
GO	GO:0050999	regulation of nitric-oxide synthase activity	2/65	42/18903	13.85	0.00917	0.0429	0.0266	2CNR1/NOD1
GO	GO:2001233	regulation of apoptotic signaling pathway	5/65	374/18903	3.89	0.00924	0.0431	0.0267	5NR4A2/SERP
GO	GO:0006690	icosanoid metabolic process	3/65	126/18903	6.92	0.00935	0.0435	0.027	3AKR1C2/AKR
GO	GO:1903037	regulation of leukocyte cell-cell adhesion	5/65	377/18903	3.86	0.00954	0.0442	0.0274	5NR4A3/ARG
GO	GO:0002347	response to tumor cell	2/65	43/18903	13.53	0.0096	0.0442	0.0274	2DAPK1/AHR
GO	GO:0009124	nucleoside monophosphate biosynthetic process	2/65	43/18903	13.53	0.0096	0.0442	0.0274	2TYMS/TK1
GO	GO:0042596	fear response	2/65	43/18903	13.53	0.0096	0.0442	0.0274	2DPP4/SLC1A
GO	GO:0051150	regulation of smooth muscle cell differentiation	2/65	43/18903	13.53	0.0096	0.0442	0.0274	2KIT/DNMT1
GO	GO:0071459	protein localization to chromosome, centromeric region	2/65	43/18903	13.53	0.0096	0.0442	0.0274	2CDK1/AURKE
GO	GO:0071548	response to dexamethasone	2/65	43/18903	13.53	0.0096	0.0442	0.0274	2NR3C1/ACE
GO	GO:0045055	regulated exocytosis	4/65	242/18903	4.81	0.00964	0.0443	0.0275	4NR4A3/CNR1
GO	GO:0051225	spindle assembly	3/65	128/18903	6.82	0.00976	0.0447	0.0278	3NEK2/AURKE
GO	GO:0045444	fat cell differentiation	4/65	244/18903	4.77	0.00991	0.0454	0.0282	4NR4A3/NR4
GO	GO:0051928	positive regulation of calcium ion transport	3/65	129/18903	6.76	0.00997	0.0455	0.0283	3MYLK/JAK3/
GO	GO:0045840	positive regulation of mitotic nuclear division	2/65	44/18903	13.22	0.01	0.0458	0.0284	2INSR/AURKA
GO	GO:1901617	organic hydroxy compound biosynthetic process	4/65	245/18903	4.75	0.0101	0.0458	0.0284	4NR4A2/NR1
GO	GO:0030900	forebrain development	5/65	383/18903	3.8	0.0102	0.0461	0.0286	5ALDH1A2/N
GO	GO:0032612	interleukin-1 production	3/65	130/18903	6.71	0.0102	0.0461	0.0286	3ARG2/NR1H
GO	GO:0032652	regulation of interleukin-1 production	3/65	130/18903	6.71	0.0102	0.0461	0.0286	3ARG2/NR1H
GO	GO:0045930	negative regulation of mitotic cell cycle	4/65	246/18903	4.73	0.0102	0.0461	0.0286	4CDK1/CCNB1
GO	GO:0033002	muscle cell proliferation	4/65	247/18903	4.71	0.0103	0.0467	0.029	4CDK1/NR4A
GO	GO:0007188	adenylate cyclase-modulating G protein-coupled receptor signaling pa	4/65	248/18903	4.69	0.0105	0.047	0.0292	4PTGER3/CNR
GO	GO:0019098	reproductive behavior	2/65	45/18903	12.93	0.0105	0.047	0.0292	2CNR1/NR3C1
GO	GO:0033003	regulation of mast cell activation	2/65	45/18903	12.93	0.0105	0.047	0.0292	2NR4A3/CNR1
GO	GO:0046636	negative regulation of alpha-beta T cell activation	2/65	45/18903	12.93	0.0105	0.047	0.0292	2ARG2/JAK3

GO	GO:0048565	digestive tract development	3/65	132/18903	6.61	0.0106	0.0474	0.0294	3	ALDH1A2/TYMS/KIT
GO	GO:0051983	regulation of chromosome segregation	3/65	132/18903	6.61	0.0106	0.0474	0.0294	3	CCNB1/AURK
GO	GO:0001101	response to acid chemical	3/65	133/18903	6.56	0.0108	0.0482	0.0299	3	SLC1A2/DNMT1/COL1A1
GO	GO:0002702	positive regulation of production of molecular mediator of immune re	3/65	133/18903	6.56	0.0108	0.0482	0.0299	3	NR4A3/KIT/N
GO	GO:0007094	mitotic spindle assembly checkpoint signaling	2/65	46/18903	12.64	0.0109	0.0484	0.03	2	CCNB1/AURK
GO	GO:0042398	cellular modified amino acid biosynthetic process	2/65	46/18903	12.64	0.0109	0.0484	0.03	2	SLC1A2/SLC1
GO	GO:0071173	spindle assembly checkpoint signaling	2/65	46/18903	12.64	0.0109	0.0484	0.03	2	CCNB1/AURK
GO	GO:0071174	mitotic spindle checkpoint signaling	2/65	46/18903	12.64	0.0109	0.0484	0.03	2	CCNB1/AURK
GO	GO:0035270	endocrine system development	3/65	135/18903	6.46	0.0113	0.0496	0.0308	3	ALDH1A2/IN
GO	GO:0042476	odontogenesis	3/65	135/18903	6.46	0.0113	0.0496	0.0308	3	ADAMTS5/SE
GO	GO:0051304	chromosome separation	3/65	135/18903	6.46	0.0113	0.0496	0.0308	3	TOP2A/CCNE
GO	GO:0031577	spindle checkpoint signaling	2/65	47/18903	12.38	0.0114	0.0498	0.0309	2	CCNB1/AURK
GO	GO:0036230	granulocyte activation	2/65	47/18903	12.38	0.0114	0.0498	0.0309	2	PLA2G2A/ITC
GO	GO:0048286	lung alveolus development	2/65	47/18903	12.38	0.0114	0.0498	0.0309	2	PGR/ACE
GO	GO:0061647	histone H3-K9 modification	2/65	47/18903	12.38	0.0114	0.0498	0.0309	2	KAT2B/DNM
GO	GO:0071470	cellular response to osmotic stress	2/65	47/18903	12.38	0.0114	0.0498	0.0309	2	MYLK/SLC2A
KEGG	hsa04933	AGE-RAGE signaling pathway in diabetic complications	30/271	101/9423	10.33	8E-23	1.63E-20	5.45E-21	30	1432/5290/6772/596/4313/5054/1277/5600/3383/207/50507/7124/5295/1019/595/5970/5580/5581/5594/5579/6774/185/2152/3717/5601/5599/4846/7046/836/4790
KEGG	hsa05417	Lipid and atherosclerosis	41/271	216/9423	6.6	1.08E-22	1.63E-20	5.45E-21	41	1432/2932/5290/3329/596/4312/4318/6714/572/598/7157/3326/5600/3309/3383/207/3551/7124/5468/1543/5747/5295/4314/6256/5970/7099/5594/3320/114548/4780/6774/2081/1559/1558/3717/5601/5599/4846/836/4790/3304
KEGG	hsa05215	Prostate cancer	30/271	106/9423	9.84	3.87E-22	3.91E-20	1.3E-20	30	2932/2475/5
KEGG	hsa05208	Chemical carcinogenesis - reactive oxygen species	40/271	227/9423	6.13	6.65E-21	5.04E-19	1.68E-19	40	1432/5290/4
KEGG	hsa04151	PI3K-Akt signaling pathway	48/271	362/9423	4.61	1.56E-19	9.47E-18	3.16E-18	48	2932/2475/5290/596/4233/3791/5228/2260/3815/572/598/4170/7157/3326/1277/2064/2321/1956/207/3551/3558/5747/5294/5295/3480/3643/1019/6256/595/1021/5970/7099/5594/3320/3164/898/3718/3717/4846/3690/3688/3685/3678/5105/4790/5159/2324/7010
KEGG	hsa05161	Hepatitis B	32/271	163/9423	6.83	2.52E-18	1.27E-16	4.24E-17	32	1432/5290/6
KEGG	hsa04066	HIF-1 signaling pathway	27/271	110/9423	8.53	2.97E-18	1.29E-16	4.29E-17	27	2475/5290/5
KEGG	hsa05205	Proteoglycans in cancer	35/271	204/9423	5.97	5.46E-18	2.07E-16	6.89E-17	35	1432/2475/5
KEGG	hsa01522	Endocrine resistance	25/271	99/9423	8.78	2.67E-17	8.98E-16	2.99E-16	25	1432/2475/5
KEGG	hsa05145	Toxoplasmosis	26/271	112/9423	8.07	5.73E-17	1.74E-15	5.79E-16	26	1432/6772/5
KEGG	hsa05167	Kaposi sarcoma-associated herpesvirus infection	33/271	196/9423	5.85	9.38E-17	2.58E-15	8.62E-16	33	1432/2932/2
KEGG	hsa05212	Pancreatic cancer	22/271	77/9423	9.93	1.31E-16	3.31E-15	1.1E-15	22	2475/5290/6
KEGG	hsa01521	EGFR tyrosine kinase inhibitor resistance	22/271	80/9423	9.56	3.23E-16	7.52E-15	2.51E-15	22	2932/2475/5
KEGG	hsa05163	Human cytomegalovirus infection	34/271	227/9423	5.21	1.24E-15	2.68E-14	8.94E-15	34	1432/2932/2
KEGG	hsa04510	Focal adhesion	32/271	203/9423	5.48	2.03E-15	4.1E-14	1.37E-14	32	2932/5290/5
KEGG	hsa05418	Fluid shear stress and atherosclerosis	27/271	142/9423	6.61	3E-15	5.69E-14	1.9E-14	27	1432/5290/5
KEGG	hsa05223	Non-small cell lung cancer	20/271	73/9423	9.53	8.17E-15	1.46E-13	4.86E-14	20	5290/4233/5
KEGG	hsa05162	Measles	26/271	136/9423	6.65	8.77E-15	1.48E-13	4.92E-14	26	2932/5290/6
KEGG	hsa05222	Small cell lung cancer	22/271	93/9423	8.23	1.03E-14	1.65E-13	5.49E-14	22	5290/596/59
KEGG	hsa05207	Chemical carcinogenesis - receptor activation	32/271	217/9423	5.13	1.43E-14	2.17E-13	7.24E-14	32	2475/5290/5
KEGG	hsa05415	Diabetic cardiomyopathy	31/271	205/9423	5.26	1.88E-14	2.71E-13	9.03E-14	31	1432/2932/2
KEGG	hsa04919	Thyroid hormone signaling pathway	24/271	122/9423	6.84	5.07E-14	6.98E-13	2.33E-13	24	2932/2475/5
KEGG	hsa04931	Insulin resistance	22/271	109/9423	7.02	3.44E-13	4.53E-12	1.51E-12	22	2932/2475/5
KEGG	hsa04917	Prolactin signaling pathway	18/271	71/9423	8.82	8.17E-13	1.03E-11	3.44E-12	18	1432/2932/5
KEGG	hsa04518	Integrin signaling	25/271	154/9423	5.64	1.43E-12	1.74E-11	5.79E-12	25	2932/2475/5
KEGG	hsa04668	TNF signaling pathway	22/271	119/9423	6.43	2.25E-12	2.62E-11	8.74E-12	22	1432/4323/5
KEGG	hsa00140	Steroid hormone biosynthesis	17/271	66/9423	8.96	2.71E-12	3.05E-11	1.02E-11	17	1545/1588/3
KEGG	hsa04068	FoxO signaling pathway	23/271	133/9423	6.01	3.01E-12	3.26E-11	1.09E-11	23	1432/1454/5
KEGG	hsa04659	Th17 cell differentiation	21/271	109/9423	6.7	3.14E-12	3.28E-11	1.09E-11	21	1432/2475/6
KEGG	hsa05220	Chronic myeloid leukemia	18/271	77/9423	8.13	3.68E-12	3.72E-11	1.24E-11	18	5290/572/59
KEGG	hsa05235	PD-L1 expression and PD-1 checkpoint pathway in cancer	19/271	90/9423	7.34	6.39E-12	6.24E-11	2.08E-11	19	1432/2475/5
KEGG	hsa05166	Human T-cell leukemia virus 1 infection	29/271	224/9423	4.5	7.69E-12	7.28E-11	2.43E-11	29	5290/7015/5
KEGG	hsa05230	Central carbon metabolism in cancer	17/271	71/9423	8.33	9.82E-12	9.02E-11	3.01E-11	17	2475/5290/4
KEGG	hsa04015	Rap1 signaling pathway	28/271	212/9423	4.59	1.11E-11	9.89E-11	3.3E-11	28	1432/5290/4
KEGG	hsa04657	IL-17 signaling pathway	19/271	94/9423	7.03	1.45E-11	1.25E-10	4.17E-11	19	1432/2932/4
KEGG	hsa04148	Efferocytosis	24/271	157/9423	5.32	1.55E-11	1.3E-10	4.35E-11	24	1432/3091/5
KEGG	hsa04621	NOD-like receptor signaling pathway	26/271	187/9423	4.83	1.94E-11	1.59E-10	5.31E-11	26	1432/6772/5
KEGG	hsa05165	Human papillomavirus infection	35/271	333/9423	3.65	2.06E-11	1.64E-10	5.47E-11	35	2932/2475/5
KEGG	hsa05206	MicroRNAs in cancer	34/271	323/9423	3.66	3.93E-11	3.05E-10	1.02E-10	34	2475/5290/1
KEGG	hsa05219	Bladder cancer	13/271	41/9423	11.03	5.93E-11	4.49E-10	1.5E-10	13	4313/4312/4
KEGG	hsa04726	Serotonergic synapse	20/271	115/9423	6.05	7.48E-11	5.53E-10	1.84E-10	20	5742/240/25
KEGG	hsa05160	Hepatitis C	23/271	156/9423	5.13	8.92E-11	6.44E-10	2.15E-10	23	2932/5290/6
KEGG	hsa04932	Non-alcoholic fatty liver disease	23/271	157/9423	5.09	1.02E-10	7.18E-10	2.39E-10	23	1432/2932/5
KEGG	hsa04926	Relaxin signaling pathway	21/271	130/9423	5.62	1.05E-10	7.24E-10	2.41E-10	21	1432/5290/4
KEGG	hsa04625	C-type lectin receptor signaling pathway	19/271	105/9423	6.29	1.11E-10	7.46E-10	2.49E-10	19	1432/5290/6
KEGG	hsa04010	MAPK signaling pathway	32/271	301/9423	3.7	1.2E-10	7.89E-10	2.63E-10	32	1432/4233/3
KEGG	hsa05131	Shigellosis	29/271	253/9423	3.99	1.57E-10	1.01E-09	3.37E-10	29	1432/2932/2
KEGG	hsa04014	Ras signaling pathway	28/271	239/9423	4.07	1.98E-10	1.23E-09	4.09E-10	28	5290/4233/3
KEGG	hsa05171	Coronavirus disease	28/271	239/9423	4.07	1.98E-10	1.23E-09	4.09E-10	28	1432/5290/6
KEGG	hsa04723	Retrograde endocannabinoid signaling	22/271	149/9423	5.13	2.26E-10	1.37E-09	4.58E-10	22	1432/2554/2
KEGG	hsa04012	ErbB signaling pathway	17/271	86/9423	6.87	2.56E-10	1.52E-09	5.07E-10	17	2932/2475/5
KEGG	hsa05210	Colorectal cancer	17/271	87/9423	6.79	3.1E-10	1.81E-09	6.03E-10	17	2932/2475/5
KEGG	hsa05135	Yersinia infection	21/271	138/9423	5.29	3.33E-10	1.9E-09	6.34E-10	21	1432/2932/5
KEGG	hsa05170	Human immunodeficiency virus 1 infection	26/271	214/9423	4.22	4.12E-10	2.31E-09	7.7E-10	26	1432/2475/5
KEGG	hsa05133	Pertussis	16/271	78/9423	7.13	4.89E-10	2.69E-09	8.99E-10	16	1432/5600/7
KEGG	hsa05225	Hepatocellular carcinoma	23/271	170/9423	4.7	5.2E-10	2.81E-09	9.39E-10	23	2932/2475/5
KEGG	hsa05221	Acute myeloid leukemia	15/271	68/9423	7.67	5.86E-10	3.11E-09	1.04E-09	15	2475/5290/3
KEGG	hsa05142	Chagas disease	18/271	103/9423	6.08	6.31E-10	3.3E-09	1.1E-09	18	1432/5290/5
KEGG	hsa05164	Influenza A	23/271	173/9423	4.62	7.41E-10	3.8E-09	1.27E-09	23	5290/6772/2
KEGG	hsa04920	Adipocytokine signaling pathway	15/271	70/9423	7.45	9.04E-10	4.57E-09	1.52E-09	15	2475/5465/2
KEGG	hsa04370	VEGF signaling pathway	14/271	60/9423	8.11	1.01E-09	4.99E-09	1.67E-09	14	1432/5290/3
KEGG	hsa05120	Epithelial cell signaling in Helicobacter pylori infection	15/271	71/9423	7.35	1.12E-09	5.46E-09	1.82E-09	15	1432/4233/6
KEGG	hsa04722	Neurotrophin signaling pathway	19/271	120/9423	5.51	1.2E-09	5.75E-09	1.92E-09	19	1432/2932/5
KEGG	hsa05224	Breast cancer	21/271	148/9423	4.93	1.25E-09	5.92E-09	1.98E-09	21	2932/2475/5
KEGG	hsa05218	Melanoma	15/271	73/9423	7.14	1.69E-09	7.86E-09	2.62E-09	15	5290/4233/2
KEGG	hsa04210	Apoptosis	20/271	138/9423	5.04	2.15E-09	9.88E-09	3.3E-09	20	5290/596/57
KEGG	hsa05214	Glioma	15/271	76/9423	6.86	3.05E-09	1.38E-08	4.6E-09	15	2475/5290/7
KEGG	hsa05169	Epstein-Barr virus infection	24/271	205/9423	4.07	4.27E-09	1.9E-08	6.34E-09	24	1432/5290/6
KEGG	hsa04936	Alcoholic liver disease	20/271	144/9423	4.83	4.6E-09	2.02E-08	6.74E-09	20	1432/2932/2
KEGG	hsa04930	Type II diabetes mellitus	12/271	47/9423	8.88	5.35E-09	2.32E-08	7.73E-09	12	2475/5290/5

KEGG	hsa05010	Alzheimer disease	34/271	389/9423	3.04	5.58E-09	2.38E-08	7.94E-09	3423621/2932/1454/2475/5290/572/6868/5743/207/50507/3551/7124/1499/1457/5295/3643/5970/5594/2081/673/5601/5599/4843/4842/4311/2915/1139/836/4790/4720/4719/4723/4698/51079
KEGG	hsa04062	Chemokine signaling pathway	23/271	193/9423	4.14	6.46E-09	2.72E-08	9.06E-09	232932/5290/6
KEGG	hsa05152	Tuberculosis	22/271	181/9423	4.23	9.67E-09	4.01E-08	1.34E-08	221432/6772/3
KEGG	hsa04935	Growth hormone synthesis, secretion and action	18/271	122/9423	5.13	1.05E-08	4.31E-08	1.44E-08	181432/2932/2
KEGG	hsa04914	Progesterone-mediated oocyte maturation	17/271	110/9423	5.37	1.34E-08	5.39E-08	1.8E-08	171432/5290/5
KEGG	hsa04071	Sphingolipid signaling pathway	18/271	125/9423	5.01	1.56E-08	6.22E-08	2.07E-08	181432/5290/5
KEGG	hsa04750	Inflammatory mediator regulation of TRP channels	16/271	99/9423	5.62	1.87E-08	7.35E-08	2.45E-08	161432/5290/6
KEGG	hsa05203	Viral carcinogenesis	23/271	205/9423	3.9	2.06E-08	8.01E-08	2.67E-08	235290/5315/1
KEGG	hsa00590	Arachidonic acid metabolism	13/271	64/9423	7.06	2.45E-08	9.39E-08	3.13E-08	139536/5742/8
KEGG	hsa05140	Leishmaniasis	14/271	78/9423	6.24	3.73E-08	1.4E-07	4.71E-08	141432/6772/5
KEGG	hsa05168	Herpes simplex virus 1 infection	21/271	180/9423	4.06	4.4E-08	1.6E-07	5.48E-08	212475/5290/6
KEGG	hsa05226	Gastric cancer	19/271	150/9423	4.4	5.23E-08	1.9E-07	6.44E-08	192932/2475/5
KEGG	hsa04520	Adherens junction	15/271	93/9423	5.61	5.41E-08	2E-07	6.58E-08	154233/6714/2
KEGG	hsa04660	T cell receptor signaling pathway	17/271	122/9423	4.85	6.51E-08	2.4E-07	7.83E-08	171432/2932/5
KEGG	hsa04620	Toll-like receptor signaling pathway	16/271	109/9423	5.1	7.62E-08	2.7E-07	9.05E-08	161432/5290/6
KEGG	hsa04915	Estrogen signaling pathway	18/271	139/9423	4.5	8.41E-08	2.9E-07	9.79E-08	185290/596/43
KEGG	hsa05213	Endometrial cancer	12/271	59/9423	7.07	8.43E-08	2.9E-07	9.79E-08	122932/5290/5
KEGG	hsa05022	Pathways of neurodegeneration - multiple diseases	36/271	481/9423	2.6	1.09E-07	3.7E-07	1.25E-07	361432/2932/1454/2475/596/572/598/5743/5600/3309/50507/7124/1499/1457/5970/5594/5579/5582/2081/11315/673/7415/5601/5599/4843/4842/2915/1139/836/4790/81631/4720/4719/4723/4698/51079
KEGG	hsa04218	Cellular senescence	19/271	157/9423	4.21	1.1E-07	3.7E-07	1.25E-07	191432/2475/5
KEGG	hsa05146	Amoebiasis	15/271	103/9423	5.06	2.18E-07	7.4E-07	2.45E-07	155290/1277/7
KEGG	hsa04630	JAK-STAT signaling pathway	19/271	168/9423	3.93	3.23E-07	1.1E-06	3.58E-07	192475/5290/6
KEGG	hsa05204	Chemical carcinogenesis - DNA adducts	12/271	70/9423	5.96	6.04E-07	2E-06	6.55E-07	121545/873/32
KEGG	hsa05211	Renal cell carcinoma	12/271	70/9423	5.96	6.04E-07	2E-06	6.55E-07	125290/4233/3
KEGG	hsa04380	Osteoclast differentiation	17/271	142/9423	4.16	6.09E-07	2E-06	6.55E-07	171432/5290/6
KEGG	hsa04613	Neutrophil extracellular trap formation	20/271	195/9423	3.57	7.69E-07	2.5E-06	8.18E-07	201432/2475/5
KEGG	hsa04670	Leukocyte transendothelial migration	15/271	116/9423	4.5	1.05E-06	3.3E-06	1.11E-06	151432/5290/4
KEGG	hsa00330	Arginine and proline metabolism	10/271	50/9423	6.95	1.22E-06	3.8E-06	1.27E-06	104129/4128/2
KEGG	hsa01524	Platinum drug resistance	12/271	75/9423	5.56	1.31E-06	4E-06	1.35E-06	125290/596/57
KEGG	hsa04140	Autophagy - animal	18/271	169/9423	3.7	1.61E-06	4.9E-06	1.65E-06	182475/5290/5
KEGG	hsa04913	Ovarian steroidogenesis	10/271	52/9423	6.69	1.79E-06	5.4E-06	1.8E-06	101545/1588/3
KEGG	hsa04658	Th1 and Th2 cell differentiation	13/271	93/9423	4.86	2.29E-06	6.9E-06	2.3E-06	131432/6772/5
KEGG	hsa04611	Platelet activation	15/271	126/9423	4.14	3.04E-06	9E-06	3.01E-06	151432/5290/5
KEGG	hsa04082	Neuroactive ligand signaling	19/271	199/9423	3.32	4.28E-06	1.3E-05	4.2E-06	192554/2561/2
KEGG	hsa04517	IgSF CAM signaling	24/271	300/9423	2.78	5.38E-06	1.6E-05	5.23E-06	241432/2932/5
KEGG	hsa04072	Phospholipase D signaling pathway	16/271	149/9423	3.73	5.56E-06	1.6E-05	5.35E-06	162475/5290/3
KEGG	hsa04217	Necroptosis	17/271	168/9423	3.52	6.36E-06	1.8E-05	6.06E-06	176772/596/33
KEGG	hsa04115	p53 signaling pathway	11/271	75/9423	5.1	8.69E-06	2.5E-05	8.21E-06	11596/5054/59
KEGG	hsa04064	NF-kappa B signaling pathway	13/271	105/9423	4.31	9.07E-06	2.6E-05	8.49E-06	13596/598/574
KEGG	hsa04910	Insulin signaling pathway	15/271	140/9423	3.73	1.13E-05	3.1E-05	1.04E-05	152932/2475/5
KEGG	hsa04912	GnRH signaling pathway	12/271	93/9423	4.49	1.31E-05	3.6E-05	1.21E-05	121432/4323/4
KEGG	hsa04519	Cadherin signaling	28/271	404/9423	2.41	1.36E-05	3.7E-05	1.24E-05	281432/2932/4
KEGG	hsa01523	Antifolate resistance	7/271	30/9423	8.11	1.74E-05	4.7E-05	1.57E-05	79429/4363/3
KEGG	hsa00380	Tryptophan metabolism	8/271	42/9423	6.62	2.13E-05	5.7E-05	1.91E-05	81545/4129/4
KEGG	hsa00591	Linoleic acid metabolism	7/271	31/9423	7.85	2.19E-05	5.8E-05	1.94E-05	7246/5320/15
KEGG	hsa04664	Fc epsilon RI signaling pathway	10/271	69/9423	5.04	2.49E-05	6.5E-05	2.18E-05	101432/5290/2
KEGG	hsa05231	Choline metabolism in cancer	12/271	99/9423	4.21	0.000025	6.5E-05	2.18E-05	122475/5290/3
KEGG	hsa05134	Legionellosis	9/271	56/9423	5.59	2.73E-05	7.1E-05	2.36E-05	93329/7124/5
KEGG	hsa05132	Salmonella infection	20/271	249/9423	2.79	3.16E-05	8.1E-05	2.71E-05	201432/5290/5
KEGG	hsa04614	Renin-angiotensin system	6/271	23/9423	9.07	3.58E-05	9.1E-05	3.04E-05	63817/3816/1
KEGG	hsa04152	AMPK signaling pathway	13/271	122/9423	3.71	4.62E-05	0.00012	3.89E-05	132475/5290/2
KEGG	hsa04110	Cell cycle	15/271	158/9423	3.3	4.77E-05	0.00012	3.98E-05	152932/5347/7
KEGG	hsa04371	Apelin signaling pathway	14/271	140/9423	3.48	4.81E-05	0.00012	3.99E-05	142475/5054/2
KEGG	hsa04211	Longevity regulating pathway	11/271	90/9423	4.25	5.04E-05	0.00012	4.14E-05	112475/5290/7
KEGG	hsa04310	Wnt signaling pathway	16/271	178/9423	3.13	5.21E-05	0.00013	4.25E-05	162932/1454/4
KEGG	hsa04213	Longevity regulating pathway - multiple species	9/271	62/9423	5.05	6.29E-05	0.00015	5.08E-05	92475/5290/2
KEGG	hsa05130	Pathogenic Escherichia coli infection	17/271	201/9423	2.94	6.59E-05	0.00016	5.29E-05	171432/6714/5
KEGG	hsa05216	Thyroid cancer	7/271	37/9423	6.58	0.000074	0.00018	5.89E-05	77157/5468/1
KEGG	hsa04024	cAMP signaling pathway	18/271	226/9423	2.77	8.82E-05	0.00021	6.93E-05	185290/572/20
KEGG	hsa04960	Aldosterone-regulated sodium reabsorption	7/271	38/9423	6.41	8.85E-05	0.00021	6.93E-05	75290/3291/5
KEGG	hsa04020	Calcium signaling pathway	19/271	254/9423	2.6	0.00013	0.0003	0.0001	194233/3791/2
KEGG	hsa05020	Prion disease	20/271	276/9423	2.52	0.000133	0.00031	0.0001	201432/2932/5
KEGG	hsa05033	Nicotine addiction	7/271	41/9423	5.94	0.000146	0.00034	0.00011	72554/2561/2
KEGG	hsa04622	RIG-I-like receptor signaling pathway	9/271	72/9423	4.35	0.000205	0.00047	0.00016	91432/340061
KEGG	hsa05202	Transcriptional misregulation in cancer	16/271	201/9423	2.77	0.000219	0.0005	0.00017	164233/4318/5
KEGG	hsa04727	GABAergic synapse	10/271	89/9423	3.91	0.000225	0.00051	0.00017	102554/6714/2
KEGG	hsa04976	Bile secretion	10/271	90/9423	3.86	0.000247	0.00055	0.00018	105243/760/94
KEGG	hsa04550	Signaling pathways regulating pluripotency of stem cells	13/271	144/9423	3.14	0.000252	0.00056	0.00019	131432/2932/5
KEGG	hsa04215	Apoptosis - multiple species	6/271	32/9423	6.52	0.000258	0.00057	0.00019	6596/598/331
KEGG	hsa04810	Regulation of actin cytoskeleton	17/271	232/9423	2.55	0.000373	0.00081	0.00027	175290/6714/2
KEGG	hsa00980	Metabolism of xenobiotics by cytochrome P450	9/271	79/9423	3.96	0.000417	0.0009	0.0003	91545/873/32
KEGG	hsa00220	Arginine biosynthesis	5/271	23/9423	7.56	0.000417	0.0009	0.0003	54843/4842/4
KEGG	hsa04080	Neuroactive ligand-receptor interaction	23/271	371/9423	2.16	0.000425	0.00091	0.0003	232554/2561/2
KEGG	hsa04650	Natural killer cell mediated cytotoxicity	12/271	133/9423	3.14	0.000436	0.00092	0.00031	125290/3383/7
KEGG	hsa04928	Parathyroid hormone synthesis, secretion and action	11/271	115/9423	3.33	0.000457	0.00096	0.00032	114323/596/43
KEGG	hsa04929	GnRH secretion	8/271	65/9423	4.28	0.000514	0.00107	0.00036	85290/2100/2
KEGG	hsa04934	Cushing syndrome	13/271	155/9423	2.92	0.000516	0.00107	0.00036	132932/1956/1
KEGG	hsa05321	Inflammatory bowel disease	8/271	66/9423	4.21	0.000571	0.00118	0.00039	86772/3558/7
KEGG	hsa04150	mTOR signaling pathway	13/271	158/9423	2.86	0.000619	0.00127	0.00042	132932/2475/5
KEGG	hsa00830	Retinol metabolism	8/271	69/9423	4.03	0.000772	0.00157	0.00052	88854/1543/2
KEGG	hsa04137	Mitophagy - animal	10/271	105/9423	3.31	0.000853	0.00172	0.00058	103091/6714/5
KEGG	hsa05014	Amyotrophic lateral sclerosis	22/271	369/9423	2.07	0.000952	0.00191	0.00064	221432/2475/5
KEGG	hsa04540	Gap junction	9/271	90/9423	3.48	0.00109	0.00217	0.00072	96714/1956/9
KEGG	hsa04662	B cell receptor signaling pathway	9/271	91/9423	3.44	0.00118	0.00231	0.00077	92932/5290/2
KEGG	hsa05032	Morphine addiction	9/271	91/9423	3.44	0.00118	0.00231	0.00077	92554/2561/2
KEGG	hsa04728	Dopaminergic synapse	11/271	133/9423	2.88	0.00154	0.00301	0.001	111432/2932/5
KEGG	hsa04923	Regulation of lipolysis in adipocytes	7/271	60/9423	4.06	0.00158	0.00307	0.00102	75290/5742/5

KEGG	hsa04081	Hormone signaling	15/271	219/9423	2.38	0.00162	0.00313	0.00104	155290/6714/2100/2099/5295/3480/3643/1813/6774/185/4988/3717/4842/3690/3685
KEGG	hsa04725	Cholinergic synapse	10/271	115/9423	3.02	0.00172	0.00329	0.0011	105290/596/20
KEGG	hsa05100	Bacterial invasion of epithelial cells	8/271	78/9423	3.57	0.00173	0.0033	0.0011	85290/4233/6
KEGG	hsa05012	Parkinson disease	17/271	269/9423	2.2	0.00194	0.00368	0.00123	17598/7157/3309/4129/4128/1813/4780/2081/11315/5601/5599/836/4720/4719/4723/4698/51079
KEGG	hsa04114	Oocyte meiosis	11/271	138/9423	2.77	0.00207	0.00389	0.0013	111432/5347/5
KEGG	hsa04916	Melanogenesis	9/271	101/9423	3.1	0.00244	0.00457	0.00152	92932/7299/3
KEGG	hsa04720	Long-term potentiation	7/271	67/9423	3.63	0.003	0.00558	0.00186	72033/1387/5
KEGG	hsa00340	Histidine metabolism	4/271	22/9423	6.32	0.00325	0.00601	0.002	44129/4128/2
KEGG	hsa04145	Phagocytosis	16/271	260/9423	2.14	0.0034	0.00625	0.00208	165290/4318/1
KEGG	hsa04922	Glucagon signaling pathway	9/271	107/9423	2.92	0.00361	0.0066	0.0022	95315/2033/1
KEGG	hsa05143	African trypanosomiasis	5/271	37/9423	4.7	0.00389	0.00706	0.00235	53383/7124/3
KEGG	hsa00760	Nicotinate and nicotinamide metabolism	5/271	38/9423	4.58	0.00438	0.00789	0.00263	510135/5167/
KEGG	hsa00982	Drug metabolism - cytochrome P450	7/271	73/9423	3.33	0.00486	0.00872	0.00291	74129/4128/1
KEGG	hsa05323	Rheumatoid arthritis	8/271	95/9423	2.93	0.00592	0.0105	0.00352	84312/3383/2
KEGG	hsa04724	Glutamatergic synapse	9/271	116/9423	2.7	0.00615	0.0109	0.00363	95594/5579/5
KEGG	hsa04360	Axon guidance	12/271	184/9423	2.27	0.00681	0.012	0.004	122932/5290/4
KEGG	hsa04666	Fc gamma R-mediated phagosome formation	8/271	98/9423	2.84	0.00712	0.0125	0.00416	85290/207/52
KEGG	hsa04730	Long-term depression	6/271	60/9423	3.48	0.0073	0.0127	0.00424	63480/5594/5
KEGG	hsa05410	Hypertrophic cardiomyopathy	8/271	99/9423	2.81	0.00756	0.0131	0.00436	81636/7124/4
KEGG	hsa05016	Huntington disease	17/271	309/9423	1.91	0.00786	0.0135	0.00451	172475/7157/2
KEGG	hsa04612	Antigen processing and presentation	7/271	82/9423	2.97	0.00912	0.0156	0.00521	73326/3309/7
KEGG	hsa00360	Phenylalanine metabolism	3/271	16/9423	6.52	0.00998	0.017	0.00567	34129/4128/4
KEGG	hsa04981	Folate transport and metabolism	4/271	31/9423	4.49	0.0114	0.0193	0.00645	49429/4363/1
KEGG	hsa04141	Protein processing in endoplasmic reticulum	11/271	175/9423	2.19	0.0122	0.0205	0.00684	11596/3326/33
KEGG	hsa05030	Cocaine addiction	5/271	49/9423	3.55	0.0129	0.0216	0.00719	54129/4128/1
KEGG	hsa04921	Oxytocin signaling pathway	10/271	154/9423	2.26	0.0133	0.0222	0.00741	106714/5743/1
KEGG	hsa00620	Pyruvate metabolism	5/271	50/9423	3.48	0.014	0.023	0.00768	55315/219/21
KEGG	hsa05144	Malaria	5/271	50/9423	3.48	0.014	0.023	0.00768	54233/3383/7
KEGG	hsa05031	Amphetamine addiction	6/271	69/9423	3.02	0.0141	0.0231	0.00771	64129/4128/5
KEGG	hsa05416	Viral myocarditis	6/271	70/9423	2.98	0.0151	0.0246	0.0082	63383/595/25
KEGG	hsa04270	Vascular smooth muscle contraction	9/271	135/9423	2.32	0.0158	0.0256	0.00853	94638/5320/5
KEGG	hsa04961	Endocrine and other factor-regulated calcium reabsorption	5/271	53/9423	3.28	0.0177	0.0285	0.0095	53817/3816/2
KEGG	hsa00350	Tyrosine metabolism	4/271	37/9423	3.76	0.021	0.0336	0.0112	47299/4129/4
KEGG	hsa00770	Pantothenate and CoA biosynthesis	3/271	21/9423	4.97	0.0213	0.034	0.0113	3219/217/516
KEGG	hsa04022	cGMP-PKG signaling pathway	10/271	166/9423	2.09	0.0215	0.0341	0.0114	10572/207/865
KEGG	hsa05017	Spinocerebellar ataxia	9/271	144/9423	2.17	0.023	0.0363	0.0121	92475/5290/2
KEGG	hsa03320	PPAR signaling pathway	6/271	77/9423	2.71	0.0231	0.0363	0.0121	64312/5465/5
KEGG	hsa00240	Pyrimidine metabolism	5/271	59/9423	2.95	0.0269	0.042	0.014	55167/7298/7
KEGG	hsa04972	Pancreatic secretion	7/271	103/9423	2.36	0.0287	0.0446	0.0149	7760/5406/53
KEGG	hsa05414	Dilated cardiomyopathy	7/271	105/9423	2.32	0.0315	0.0487	0.0162	77124/4842/3
KEGG	hsa04623	Cytosolic DNA-sensing pathway	6/271	83/9423	2.51	0.032	0.0492	0.0164	6340061/3551
