# Supplementary material for: Multi-Target Anti-Endometriosis Mechanisms of Camellia sinensis Polyphenols: An Integrated Computational and Transcriptomic Study
Source: Life (Basel). 2026 Sep 26;16(10):1613. doi: 10.3390/life16101613 (PMC13705937; doi:10.3390/life16101613)
Supplement: Supplementary file 1 [file life-16-01613-s001.zip › Tables S1, S2, S3, S5, S6, S7, S8 Figures S1, S2.pdf]

# Table S1

## Identification of the 20 *C. sinensis* compounds selected for network analysis

| No. | Compound | Ion mode | RT (min) | Observed m/z | Mass error (ppm) | ID score | MS/MS lib score | Identification basis | MSI level | Rel. height (%)* | Notes |
|---|---|---|---|---|---|---|---|---|---|---|---|
| 1 | (–)-Epigallocatechin gallate (EGCG) | ESI- | 10.93 | 457.0773 | 0.75 | 98.3 | 97.5 | Accurate mass + MS/MS library | Level 2 | 3.922 | |
| 2 | Epicatechin | ESI+ | 10.62 | 291.0868 | -1.66 | 97.5 | 97.2 | Accurate mass + MS/MS library | Level 2 | 3.847 | also detected in ESI– |
| 3 | (±)-Catechin | ESI- | 9.68 | 289.0721 | -1.18 | 93.3 | 89.5 | Accurate mass + MS/MS library | Level 2 | 5.589 | also detected in ESI+ |
| 4 | Rutin | ESI- | 11.98 | 609.1457 | 0.8 | 98.6 | 98.6 | Accurate mass + MS/MS library | Level 2 | 4.638 | |
| 5 | Quercetin 3-galactoside | ESI- | 12.54 | 463.088 | 0.62 | 96.2 | 94.2 | Accurate mass + MS/MS library | Level 2 | 3.846 | also detected in ESI+ |
| 6 | Procyanidin B2 | ESI+ | 9.98 | 579.1492 | 0.99 | 94.6 | 89.9 | Accurate mass + MS/MS library | Level 2 | 0.464 | also detected in ESI– |
| 7 | (–)-Naringenin | ESI+ | 12.81 | 273.0761 | -1.21 | 93.9 | 89.3 | Accurate mass + MS/MS library | Level 2 | 0.786 | |
| 8 | Kaempferol | ESI+ | 13.46 | 287.0554 | -1.46 | 96.6 | 94.9 | Accurate mass + MS/MS library | Level 2 | 0.162 | |
| 9 | Chlorogenic acid | ESI- | 8.07 | 353.0875 | 1.01 | 94.4 | 90.1 | Accurate mass + MS/MS library | Level 2 | 0.961 | |
| 10 | Gallic acid | ESI- | 12.89 | 169.0142 | 0.24 | 93.9 | 88.2 | Accurate mass + MS/MS library | Level 2 | 0.456 | |
| 11 | Caffeic acid | ESI- | 9.57 | 179.0349 | 0.4 | 93.5 | 87.2 | Accurate mass + MS/MS library | Level 2 | 0.259 | |
| 12 | Herbacetin | ESI+ | 12.51 | 303.0503 | -1.25 | 93.3 | 89.4 | Accurate mass + MS/MS library | Level 2 | 0.624 | |

| | | | | | | | | | | | |
|---|---|---|---|---|---|---|---|---|---|---|---|
| 13 | Caffeine | ESI+ | 9.47 | 195.0902 | -13.26 | 48.3 | 96.7 | Accurate mass + MS/MS library | Level 2 | 15.093 | MS/MS library score 96.7; accurate-mass deviation reflects detector saturation of the dominant peak |
| 14 | Theanine | ESI- | 3.35 | 173.0932 | -0.22 | 88.5 | 89.8 | Accurate mass + MS/MS library | Level 2 | 1.552 | |
| 15 | Theophylline | ESI+ | 7.11 | 181.0723 | -1.89 | 86.9 | 87.5 | Accurate mass + MS/MS library | Level 2 | 0.206 | |
| 16 | Marmesin | ESI- | 10.65 | 245.0821 | -0.4 | 89.2 | 92 | Accurate mass + MS/MS library | Level 2 | 0.768 | |
| 17 | 4-Hydroxycoumarin | ESI+ | 8.06 | 163.0392 | -2.34 | 88.9 | 83.2 | Accurate mass + MS/MS library | Level 2 | 0.8 | |
| 18 | Aesculin | ESI- | 8.55 | 339.0718 | 1.28 | 97.3 | 96.2 | Accurate mass + MS/MS library | Level 2 | 0.076 | |
| 19 | Epicatechin gallate (ECG) | ESI- | 12.84 | 441.0825 | 0.36 | 49.2 | n.a. | Accurate mass + formula | Level 3 | 5.947 | accurate-mass/formula match; isomeric catechin not confirmed by MS/MS library |
| 20 | Epigallocatechin (EGC) | ESI+ | 7.54 | 307.0812 | 0.08 | 49.5 | n.a. | Accurate mass + formula | Level 3 | 0.664 | accurate-mass/formula match; isomeric catechin not confirmed by MS/MS library |

# Table S2

## Complete list of 159 accurate-mass features detected in *C. sinensis* extract

| Cpd # | Putative name | Formula | Ion mode | RT (min) | Observed m/z | Neutral mass | Mass error (ppm) | Overall score | DB score | MS/MS lib score | Peak height | Rel. height (%)* | ID source |
|---|---|---|---|---|---|---|---|---|---|---|---|---|---|
| 1 | Galactose-beta-1,4-xylose | C11H20O10 | ESI+ | 1.915 | 335.0953 | 312.106 | -1.05 | 49.55 | 99.1 | 0 | 401971 | 0.4969 | DBSearch |
| 2 | Choline | C5H14NO | ESI+ | 1.953 | 104.1073 | 104.1078 | -2.94 | 90.87 | 86.62 | 95.12 | 1873513 | 2.3161 | DBSearch-LibSearch |
| 3 | Betaine | C5H12NO2 | ESI+ | 1.99 | 118.0864 | 118.087 | -1.67 | 91.67 | 86.75 | 96.6 | 1828578 | 2.2606 | DBSearch-LibSearch |
| 4 | Castanospermine | C8H15NO4 | ESI+ | 2.066 | 190.1076 | 189.1003 | -1.01 | 95.21 | 99.53 | 90.9 | 317342 | 0.3923 | DBSearch-LibSearch |
| 5 | Valiolone | C7H12O6 | ESI+ | 2.241 | 215.053 | 192.0637 | -1.88 | 49.4 | 98.8 | 0 | 498387 | 0.6161 | DBSearch |
| 6 | Apiumoside | C29H30O12 | ESI+ | 2.266 | 308.0763 | 570.1739 | -0.32 | 45.17 | 90.33 | 0 | 2214506 | 2.7377 | DBSearch |
| 7 | 2-(beta-D-Glucosyl)-sn-glycerol | C9H18O8 | ESI+ | 2.341 | 277.0896 | 254.1004 | -0.81 | 49.45 | 98.89 | 0 | 358833 | 0.4436 | DBSearch |
| 8 | Sucrose | C12H22O11 | ESI+ | 2.643 | 365.1059 | 342.1167 | -1.31 | 99.06 | 98.76 | 99.35 | 5531291 | 6.8381 | DBSearch-LibSearch |
| 9 | Biotin amide | C10H17N3O2S | ESI+ | 2.743 | 266.0932 | 243.104 | 0.51 | 49.29 | 98.58 | 0 | 76840 | 0.095 | DBSearch |
| 10 | (-)-Dioxibrassinin | C11H12N2O2S2 | ESI+ | 2.818 | 306.9972 | 268.0343 | -0.87 | 36.37 | 72.75 | 0 | 134889 | 0.1668 | DBSearch |
| 11 | 3-Galactosyllactose | C18H32O16 | ESI+ | 3.144 | 527.1578 | 504.1684 | 1.32 | 49.05 | 98.11 | 0 | 32534 | 0.0402 | DBSearch |
| 12 | Glycyl-Valine | C7H14N2O3 | ESI+ | 3.32 | 175.108 | 174.1007 | -1.6 | 43.58 | 87.16 | 0 | 400372 | 0.495 | DBSearch |
| 13 | N-Methacrylylglycine methyl ester | C7H11NO3 | ESI+ | 3.358 | 158.0818 | 157.0746 | -4.19 | 42.57 | 85.14 | 0 | 3797262 | 4.6944 | DBSearch |
| 14 | Adenine | C5H5N5 | ESI+ | 3.446 | 136.0617 | 135.0545 | 0.11 | 43.94 | 87.89 | 0 | 399247 | 0.4936 | DBSearch |
| 15 | Cytosine | C4H5N3O | ESI+ | 3.747 | 112.0507 | 111.0435 | -1.92 | 43.72 | 87.44 | 0 | 60075 | 0.0743 | DBSearch |
| 16 | L-isoleucyl-L-proline | C11H20N2O3 | ESI+ | 4.199 | 229.1547 | 228.1475 | -0.44 | 49.81 | 99.62 | 0 | 339555 | 0.4198 | DBSearch |
| 17 | 12alpha-Fluoro-11beta,17beta-dihydroxyandrosta-1,4-dien-3-one | C19H25FO3 | ESI+ | 4.55 | 359.1425 | 320.1794 | -1.82 | 41.58 | 83.16 | 0 | 155684 | 0.1925 | DBSearch |
| 18 | (-)-trans-Carveol glucoside | C16H26O6 | ESI+ | 4.55 | 337.161 | 314.1716 | 4.15 | 45.59 | 91.17 | 0 | 1118899 | 1.3832 | DBSearch |

| | | | | | | | | | | | | | |
|---|---|---|---|---|---|---|---|---|---|---|---|---|---|
| 19 | Pirbuterol | C12H20N2O3 | ESI+ | 4.676 | 241.1547 | 240.1474 | 0.11 | 49.99 | 99.99 | 0 | 261968 | 0.3239 | DBSearch |
| 20 | Guanine | C5H5N5O | ESI+ | 4.902 | 152.0566 | 151.0494 | 0.22 | 87.35 | 87.41 | 87.28 | 132420 | 0.1637 | DBSearch-LibSearch |
| 21 | Butamben | C11H15NO2 | ESI+ | 4.902 | 194.1178 | 193.1105 | -0.94 | 49.74 | 99.49 | 0 | 307511 | 0.3802 | DBSearch |
| 22 | (S)-5'-Deoxy-5'-(methylsulfinyl) adenosine | C11H15N5O4S | ESI+ | 4.927 | 336.0736 | 313.0844 | 0.12 | 49.6 | 99.2 | 0 | 98640 | 0.1219 | DBSearch |
| 23 | Adenosine | C10H13N5O4 | ESI+ | 5.127 | 268.1046 | 267.0973 | -1.99 | 98.74 | 98.55 | 98.93 | 129212 | 0.1597 | DBSearch-LibSearch |
| 24 | 2-Hydroxyadenine | C5H5N5O | ESI+ | 5.453 | 152.0564 | 151.0492 | 1.58 | 43.64 | 87.29 | 0 | 39594 | 0.0489 | DBSearch |
| 25 | N-(1-Deoxy-1-fructosyl)leucine | C12H23NO7 | ESI+ | 5.504 | 316.1368 | 293.1476 | -0.46 | 49.9 | 99.8 | 0 | 398724 | 0.4929 | DBSearch |
| 26 | D-Pipecolic acid | C6H11NO2 | ESI+ | 5.855 | 130.0861 | 129.0789 | 0.92 | 49.04 | 98.08 | 0 | 37867 | 0.0468 | DBSearch |
| 27 | Theogallin | C14H16O10 | ESI+ | 5.906 | 367.0639 | 344.0747 | -1.03 | 49.7 | 99.4 | 0 | 3986115 | 4.9278 | DBSearch |
| 28 | Cyanidin-3-O-(6''-O-malonyl-2''-O-glucuronyl)glucoside | C30H31O20 | ESI+ | 5.955 | 711.1381 | 711.138 | 4.06 | 42.63 | 85.25 | 0 | 112995 | 0.1397 | DBSearch |
| 29 | DL-Phenylalanine | C9H11NO2 | ESI+ | 6.457 | 166.0865 | 165.0793 | -1.65 | 92.35 | 87.32 | 97.38 | 137871 | 0.1704 | DBSearch-LibSearch |
| 30 | Theophylline | C7H8N4O2 | ESI+ | 7.11 | 181.0723 | 180.0651 | -1.89 | 86.91 | 86.29 | 87.52 | 166893 | 0.2063 | DBSearch-LibSearch |
| 31 | (+)-Gallocatechin (EGC) | C15H14O7 | ESI+ | 7.537 | 307.0812 | 306.0739 | 0.08 | 49.48 | 98.96 | 0 | 536828 | 0.6637 | DBSearch |
| 32 | 4-Hydroxycoumarin | C9H6O3 | ESI+ | 8.064 | 163.0392 | 162.0321 | -2.34 | 88.87 | 94.59 | 83.15 | 647048 | 0.7999 | DBSearch-LibSearch |
| 33 | N-(2,5-Dihydroxyphenyl)pyridinium(1+) | C11H10NO2 | ESI+ | 8.415 | 188.0711 | 188.0716 | -2.61 | 49.29 | 98.57 | 0 | 285273 | 0.3527 | DBSearch |
| 34 | Oxyquinoline | C9H7NO | ESI+ | 8.44 | 146.0602 | 145.0529 | -1.07 | 43.69 | 87.38 | 0 | 99515 | 0.123 | DBSearch |
| 35 | 3-Hydroxy-3-(3,4-dihydroxy-4-methylpentanoyl)-5-(3-methylbutyl)-1,2,4-cyclopentanetrione | C16H22O8 | ESI+ | 8.566 | 365.1211 | 342.1319 | -1.32 | 49.18 | 98.36 | 0 | 4818263 | 5.9566 | DBSearch |

| | | | | | | | | | | | | | |
|---|---|---|---|---|---|---|---|---|---|---|---|---|---|
| 36 | Catechin-4beta-ol | C15H14O7 | ESI+ | 8.892 | 307.0818 | 306.0745 | -1.8 | 49.29 | 98.58 | 0 | 1768595 | 2.1864 | DBSearch |
| 37 | Phenylethyl primeveroside | C19H28O10 | ESI+ | 9.118 | 439.1578 | 416.1685 | -0.59 | 49.11 | 98.22 | 0 | 942788 | 1.1655 | DBSearch |
| 38 | Cis-5-Caffeoylquinic acid | C16H18O9 | ESI+ | 9.344 | 377.0844 | 354.0951 | 0.01 | 49.66 | 99.33 | 0 | 86163 | 0.1065 | DBSearch |
| 39 | 2-[4-(3-Hydroxypropyl)-2-methoxyphenoxy]-1,3-propanediol 1-glucoside | C19H30O10 | ESI+ | 9.369 | 441.173 | 418.1837 | 0.47 | 49.72 | 99.44 | 0 | 53157 | 0.0657 | DBSearch |
| 40 | Caffeine | C8H10N4O2 | ESI+ | 9.47 | 195.0902 | 194.0829 | -13.26 | 48.33 | | 96.65 | 12208784 | 15.0932 | LibSearch |
| 41 | trans-p-Menthane-1,7,8-triol 8-glucoside | C16H30O8 | ESI+ | 9.507 | 373.1839 | 350.1946 | -1.55 | 49.28 | 98.55 | 0 | 632730 | 0.7822 | DBSearch |
| 42 | Isoniazid | C6H7N3O | ESI+ | 9.545 | 138.0664 | 137.0592 | -1.76 | 43.15 | 86.3 | 0 | 529661 | 0.6548 | DBSearch |
| 43 | (±)-Catechin | C15H14O6 | ESI+ | 9.645 | 291.0871 | 290.0798 | -2.57 | 48.31 | 96.62 | | 482117 | 0.596 | DBSearch |
| 44 | Procyanidin B2 | C30H26O12 | ESI+ | 9.984 | 579.1492 | 578.1419 | 0.99 | 94.58 | 99.31 | 89.86 | 375091 | 0.4637 | DBSearch-LibSearch |
| 45 | Procyanidin B3 | C30H26O12 | ESI+ | 10.021 | 601.1309 | 578.1416 | 1.41 | 48.94 | 97.87 | 0 | 201700 | 0.2494 | DBSearch |
| 46 | Phenylpropiolic acid | C9H6O2 | ESI+ | 10.423 | 147.0442 | 146.037 | -1.64 | 49.05 | 98.1 | 0 | 220791 | 0.273 | DBSearch |
| 47 | Epicatechin | C15H14O6 | ESI+ | 10.624 | 291.0868 | 290.0795 | -1.66 | 97.52 | 97.82 | 97.23 | 3112086 | 3.8473 | DBSearch-LibSearch |
| 48 | cis-3,4-Leucopelargonidin | C15H14O6 | ESI+ | 10.674 | 313.0688 | 290.0795 | -1.74 | 49.17 | 98.34 | 0 | 260429 | 0.322 | DBSearch |
| 49 | Lucuminic acid | C19H26O12 | ESI+ | 10.724 | 469.1321 | 446.1428 | -0.75 | 49.59 | 99.19 | 0 | 2307033 | 2.8521 | DBSearch |
| 50 | 2-Methoxy-1,4-benzoquinone | C7H6O3 | ESI+ | 10.749 | 139.0391 | 138.0318 | -1.02 | 43.77 | 87.54 | 0 | 1669427 | 2.0638 | DBSearch |
| 51 | Porric acid B | C15H12O6 | ESI+ | 10.875 | 289.071 | 288.0639 | -1.71 | 48.2 | 96.4 | 0 | 723327 | 0.8942 | DBSearch |
| 52 | 2-Methoxycarbonylphenyl beta-D-glucopyranoside | C14H18O8 | ESI+ | 11.126 | 337.0898 | 314.1005 | -1.09 | 49.3 | 98.59 | 0 | 210878 | 0.2607 | DBSearch |
| 53 | ent-Epicatechin-(4alpha->8)-ent-epicatechin 3'-gallate | C37H30O16 | ESI+ | 11.327 | 731.1599 | 730.1526 | 1.11 | 49.35 | 98.7 | 0 | 196460 | 0.2429 | DBSearch |

| | | | | | | | | | | | | | |
|---|---|---|---|---|---|---|---|---|---|---|---|---|---|
| 54 | Margrapine A | C21H23NO7 | ESI+ | 11.678 | 424.1365 | 401.1475 | -0.01 | 49.62 | 99.24 | 0 | 50877 | 0.0629 | DBSearch |
| 55 | Malvidin 3-(6-acetylglucoside) | C25H27O13 | ESI+ | 11.854 | 535.1445 | 535.1448 | 0.7 | 49.37 | 98.74 | 0 | 57605 | 0.0712 | DBSearch |
| 56 | Luteolin 6-C-glucoside 8-C-arabinoside | C27H30O16 | ESI+ | 12.004 | 633.1418 | 610.1524 | 1.65 | 48.76 | 97.52 | 0 | 1019328 | 1.2601 | DBSearch |
| 57 | Delphinidin | C15H11O7 | ESI+ | 12.079 | 303.0502 | 303.0507 | -0.74 | 49.28 | 98.56 | 0 | 56725 | 0.0701 | DBSearch |
| 58 | Carissanol | C20H24O7 | ESI+ | 12.431 | 399.1422 | 376.1528 | -1.72 | 48.99 | 97.99 | 0 | 1140822 | 1.4103 | DBSearch |
| 59 | Herbacetin | C15H10O7 | ESI+ | 12.506 | 303.0503 | 302.043 | -1.25 | 93.29 | 97.18 | 89.4 | 504931 | 0.6242 | DBSearch-LibSearch |
| 60 | Quercetin 3-galactoside | C21H20O12 | ESI+ | 12.531 | 487.0852 | 464.0958 | -0.71 | 48.62 | 97.23 | 0 | 1168531 | 1.4446 | DBSearch |
| 61 | Carinol | C20H26O7 | ESI+ | 12.657 | 401.1569 | 378.1676 | 0.54 | 49.65 | 99.29 | 0 | 77129 | 0.0954 | DBSearch |
| 62 | Linalool oxide D 3-[apiosyl-(1->6)-glucoside] | C21H36O11 | ESI+ | 12.682 | 487.2155 | 464.2261 | -0.74 | 49.1 | 98.21 | 0 | 813995 | 1.0063 | DBSearch |
| 63 | 7-Galloylcatechin (ECG) | C22H18O10 | ESI+ | 12.782 | 443.0974 | 442.0901 | -0.25 | 49.83 | 99.66 | 0 | 916981 | 1.1336 | DBSearch |
| 64 | (-)-Naringenin | C15H12O5 | ESI+ | 12.807 | 273.0761 | 272.0688 | -1.21 | 93.87 | 98.41 | 89.34 | 636019 | 0.7863 | DBSearch-LibSearch |
| 65 | Isovitexin 2''-O-glucoside | C27H30O15 | ESI+ | 12.833 | 617.1479 | 594.1585 | 0.02 | 49.21 | 98.41 | 0 | 593897 | 0.7342 | DBSearch |
| 66 | (7'S,8'S)-4,7'-Epoxy-3,8'-bilign-7-ene-3',5-dimethoxy-4',9,9'-triol 4'-glucoside | C26H32O11 | ESI+ | 13.058 | 543.1833 | 520.1942 | 0.41 | 48.08 | 96.16 | 0 | 46635 | 0.0577 | DBSearch |
| 67 | Unshuoside A | C16H28O7 | ESI+ | 13.084 | 355.173 | 332.1838 | -0.75 | 49.37 | 98.74 | 0 | 291076 | 0.3598 | DBSearch |
| 68 | 3-Methylene-indolenine | C9H7N | ESI+ | 13.159 | 130.0654 | 129.0581 | -2.25 | 43.41 | 86.82 | 0 | 152370 | 0.1884 | DBSearch |
| 69 | Isolariciresinol 9-O-beta-D-glucoside | C26H34O11 | ESI+ | 13.259 | 545.1987 | 522.2094 | 1.28 | 49.19 | 98.38 | 0 | 64957 | 0.0803 | DBSearch |
| 70 | Quercitrin | C21H20O11 | ESI+ | 13.435 | 471.0902 | 448.1009 | -0.7 | 48.49 | 96.99 | 0 | 394481 | 0.4877 | DBSearch |
| 71 | Kaempferol | C15H10O6 | ESI+ | 13.46 | 287.0554 | 286.0482 | -1.46 | 96.64 | 98.43 | 94.86 | 130990 | 0.1619 | DBSearch-LibSearch |
| 72 | 2,6-Dimethoxy-4-propylphenol | C11H16O3 | ESI+ | 13.837 | 219.0996 | 196.1103 | -1.9 | 42.95 | 85.89 | 0 | 176659 | 0.2184 | DBSearch |
| 73 | SB 243213 | C22H19F3N4O2 | ESI+ | 13.912 | 429.152 | 428.1452 | 1.84 | 38.09 | 76.18 | 0 | 785246 | 0.9708 | DBSearch |
| 74 | Tetrahydroaldosterone-3-glucuronide | C27H40O11 | ESI+ | 14.64 | 579.2199 | 540.257 | 0.05 | 43.15 | 86.3 | 0 | 206785 | 0.2556 | DBSearch |

| | | | | | | | | | | | | | |
|---|---|---|---|---|---|---|---|---|---|---|---|---|---|
| 75 | 1-O-Feruloylglucose | C16H20O9 | ESI+ | 14.665 | 379.1001 | 356.1108 | -0.26 | 49.5 | 99.01 | 0 | 75083 | 0.0928 | DBSearch |
| 76 | Valerosidatum | C21H34O11 | ESI+ | 14.966 | 501.1728 | 462.2098 | 0.64 | 43.53 | 87.05 | 0 | 40255 | 0.0498 | DBSearch |
| 77 | Acuminoside | C21H36O10 | ESI+ | 15.844 | 471.2203 | 448.2311 | -0.54 | 49.18 | 98.37 | 0 | 434334 | 0.5369 | DBSearch |
| 78 | Phenazine-1,6-dicarboxylic acid | C14H8N2O4 | ESI+ | 15.894 | 291.0388 | 268.0496 | -4.3 | 43.69 | 87.38 | 0 | 123380 | 0.1525 | DBSearch |
| 79 | Lappaol C | C30H34O10 | ESI+ | 16.346 | 577.2039 | 554.2146 | 1.15 | 49.46 | 98.91 | 0 | 91481 | 0.1131 | DBSearch |
| 80 | Musabalbisiane C | C28H40O12 | ESI+ | 17.652 | 607.2146 | 568.2516 | 0.63 | 43.73 | 87.45 | 0 | 78531 | 0.0971 | DBSearch |
| 81 | Palmitic amide | C16H33NO | ESI+ | 20.713 | 256.2635 | 255.2563 | -0.16 | 49.71 | 99.41 | 0 | 128659 | 0.1591 | DBSearch |
| 82 | C16 Sphinganine | C16H35NO2 | ESI+ | 20.713 | 274.275 | 273.2678 | -3.58 | 47.42 | 94.83 | 0 | 12779480 | 15.7987 | DBSearch |
| 83 | Octadecanedioic acid | C18H34O4 | ESI+ | 25.708 | 337.2354 | 314.246 | -0.92 | 48.65 | 97.31 | 0 | 56231 | 0.0695 | DBSearch |
| 84 | Alpha-CEHC | C16H22O4 | ESI+ | 32.71 | 301.1411 | 278.1519 | -0.2 | 49.86 | 99.71 | 0 | 78360 | 0.0969 | DBSearch |
| 85 | Galactose-beta-1,4-xylose | C11H20O10 | ESI- | 1.857 | 311.0982 | 312.1055 | 0.46 | 49.69 | 99.37 | 0 | 408963 | 0.3699 | DBSearch |
| 86 | Galactonic acid | C6H12O7 | ESI- | 1.983 | 195.0508 | 196.0581 | 1 | 94.51 | 99.25 | 89.78 | 144659 | 0.1308 | DBSearch-LibSearch |
| 87 | L-Xylonate | C5H10O6 | ESI- | 1.995 | 165.0402 | 166.0475 | 1.39 | 49.77 | 99.54 | 0 | 173383 | 0.1568 | DBSearch |
| 88 | Threonate | C4H8O5 | ESI- | 2.096 | 135.0299 | 136.0372 | -0.04 | 49.98 | 99.96 | 0 | 114957 | 0.104 | DBSearch |
| 89 | UDP-glucose | C15H24N2O17P2 | ESI- | 2.146 | 565.0467 | 566.054 | 1.82 | 94.87 | 97.18 | 92.55 | 59170 | 0.0535 | DBSearch-LibSearch |
| 90 | Acetyl-maltose | C14H24O12 | ESI- | 2.296 | 383.119 | 384.1264 | 1.07 | 49.52 | 99.04 | 0 | 612329 | 0.5539 | DBSearch |
| 91 | Quinic acid | C7H12O6 | ESI- | 2.297 | 191.057 | 192.0643 | -4.5 | 93.3 | 95.74 | 90.87 | 19851552 | 17.9565 | DBSearch-LibSearch |
| 92 | Citbismine C | C37H36N2O11 | ESI- | 2.623 | 683.2253 | 684.2324 | -0.79 | 40.36 | 80.72 | 0 | 421270 | 0.3811 | DBSearch |
| 93 | Sucrose | C12H22O11 | ESI- | 2.648 | 341.1091 | 342.1164 | -0.42 | 98.65 | 99.26 | 98.03 | 2609598 | 2.3605 | DBSearch-LibSearch |
| 94 | Malic acid | C4H6O5 | ESI- | 2.799 | 133.0143 | 134.0215 | -0.18 | 92.68 | 86.92 | 98.45 | 123600 | 0.1118 | DBSearch-LibSearch |
| 95 | Maleic acid | C4H4O4 | ESI- | 2.823 | 112.9853 | 113.9925 | 17706.56 | 49.08 | | 98.16 | 29252 | 0.0265 | LibSearch |
| 96 | Shikimic acid | C7H10O5 | ESI- | 2.999 | 173.0456 | 174.0529 | -0.27 | 94.65 | 99.9 | 89.39 | 262023 | 0.237 | DBSearch-LibSearch |
| 97 | D-Galactopyranosyl-(1->3)-D-galactopyranosyl-(1->3)-L-arabinose | C17H30O15 | ESI- | 3.1 | 473.1508 | 474.1581 | 0.7 | 49.56 | 99.11 | 0 | 154219 | 0.1395 | DBSearch |
| 98 | Raffinose | C18H32O16 | ESI- | 3.125 | 503.1608 | 504.1681 | 1.86 | 93.39 | 97.23 | 89.55 | 41472 | 0.0375 | DBSearch-LibSearch |

| | | | | | | | | | | | | | |
|---|---|---|---|---|---|---|---|---|---|---|---|---|---|
| 99 | Theanine | C7H14N2O3 | ESI- | 3.351 | 173.0932 | 174.1005 | -0.22 | 88.46 | 87.18 | 89.75 | 1715692 | 1.5519 | DBSearch-LibSearch |
| 100 | L-Ascorbic acid-2-glucoside | C12H18O11 | ESI- | 4.104 | 337.0774 | 338.0847 | 0.66 | 49.87 | 99.74 | 0 | 156258 | 0.1413 | DBSearch |
| 101 | N-Acryloylglycine | C5H7NO3 | ESI- | 4.606 | 128.0353 | 129.0425 | 0.52 | 43.87 | 87.75 | 0 | 81701 | 0.0739 | DBSearch |
| 102 | threo-Isocitric acid | C6H8O7 | ESI- | 4.63 | 191.0199 | 192.0271 | -0.47 | 49.82 | 99.64 | 0 | 51588 | 0.0467 | DBSearch |
| 103 | 3-Furoic acid | C5H4O3 | ESI- | 4.655 | 111.0088 | 112.0161 | -0.74 | 43.89 | 87.77 | 0 | 47538 | 0.043 | DBSearch |
| 104 | Aminocaproic acid | C6H13NO2 | ESI- | 4.932 | 130.0873 | 131.0947 | -0.21 | 75.94 | 87.75 | 64.14 | 57379 | 0.0519 | DBSearch-LibSearch |
| 105 | Adenine | C5H5N5 | ESI- | 5.108 | 134.0472 | 135.0545 | -0.02 | 89.01 | 87.87 | 90.15 | 236801 | 0.2142 | DBSearch-LibSearch |
| 106 | 6-Galloylglucose | C13H16O10 | ESI- | 5.258 | 331.0668 | 332.0741 | 0.65 | 49.89 | 99.77 | 0 | 1173840 | 1.0618 | DBSearch |
| 107 | Theogallin | C14H16O10 | ESI- | 5.986 | 343.067 | 344.0743 | 0.13 | 49.9 | 99.8 | 0 | 3243650 | 2.934 | DBSearch |
| 108 | L-Phenylalanine | C9H11NO2 | ESI- | 6.438 | 164.0718 | 165.079 | -0.31 | 92.59 | 87.55 | 97.64 | 283676 | 0.2566 | DBSearch-LibSearch |
| 109 | Epicatechin-(2beta->7,4beta->6)-catechin | C30H24O12 | ESI- | 7.216 | 575.1185 | 576.1259 | 1.59 | 48.81 | 97.61 | 0 | 117533 | 0.1063 | DBSearch |
| 110 | (+)-Gallocatechin | C15H14O7 | ESI- | 7.567 | 305.067 | 306.0742 | -0.95 | 49.6 | 99.21 | 0 | 3041796 | 2.7514 | DBSearch |
| 111 | 6''-O-Caffeoylastragalin | C30H26O14 | ESI- | 7.919 | 609.1243 | 610.1314 | 1.41 | 49.03 | 98.06 | 0 | 139915 | 0.1266 | DBSearch |
| 112 | Chlorogenic Acid | C16H18O9 | ESI- | 8.069 | 353.0875 | 354.0947 | 1.01 | 94.38 | 98.62 | 90.14 | 1062402 | 0.961 | DBSearch-LibSearch |
| 113 | Epicatechin-(4beta->8)-gallocatechin | C30H26O13 | ESI- | 8.22 | 593.1288 | 594.1362 | 1.89 | 47.4 | 94.79 | 0 | 175849 | 0.1591 | DBSearch |
| 114 | DL-Tryptophan | C11H12N2O2 | ESI- | 8.421 | 203.0824 | 204.0897 | 0.87 | 90.2 | 85.27 | 95.13 | 58249 | 0.0527 | DBSearch-LibSearch |
| 115 | Verbenalin | C17H24O10 | ESI- | 8.521 | 387.1294 | 388.1366 | 0.79 | 49.65 | 99.3 | 0 | 89712 | 0.0811 | DBSearch |
| 116 | Aesculin | C15H16O9 | ESI- | 8.546 | 339.0718 | 340.079 | 1.28 | 97.32 | 98.49 | 96.15 | 83759 | 0.0758 | DBSearch-LibSearch |
| 117 | Hamamelitannin | C20H20O14 | ESI- | 8.747 | 483.0773 | 484.0845 | 1.62 | 49.11 | 98.22 | 0 | 716697 | 0.6483 | DBSearch |
| 118 | 2-Methoxy-1,4-benzoquinone | C7H6O3 | ESI- | 8.922 | 137.0241 | 138.0317 | 0.12 | 36.83 | 73.67 | 0 | 379647 | 0.3434 | DBSearch |
| 119 | Catechin-4beta-ol | C15H14O7 | ESI- | 8.935 | 305.0668 | 306.0741 | -0.48 | 49.65 | 99.31 | 0 | 7139669 | 6.4581 | DBSearch |
| 120 | m-Coumaric acid | C9H8O3 | ESI- | 9.173 | 163.0398 | 164.0471 | 1.52 | 98.89 | 98.75 | 99.03 | 137509 | 0.1244 | DBSearch-LibSearch |
| 121 | Sanguiin H4 | C27H22O18 | ESI- | 9.387 | 633.073 | 634.0801 | 0.75 | 49.67 | 99.34 | 0 | 1458842 | 1.3196 | DBSearch |

| | | | | | | | | | | | | | |
|---|---|---|---|---|---|---|---|---|---|---|---|---|---|
| 122 | Cis-5-Caffeoylquinic acid | C16H18O9 | ESI- | 9.449 | 353.0879 | 354.0952 | -0.32 | 49.32 | 98.65 | 0 | 3839046 | 3.4726 | DBSearch |
| 123 | Cinnamtannin A1 | C45H38O18 | ESI- | 9.474 | 865.1963 | 866.2036 | 2.57 | 46.98 | 93.96 | 0 | 141987 | 0.1284 | DBSearch |
| 124 | Caffeic Acid | C9H8O4 | ESI- | 9.574 | 179.0349 | 180.0422 | 0.4 | 93.48 | 99.77 | 87.18 | 286575 | 0.2592 | DBSearch-LibSearch |
| 125 | (±)-Catechin | C15H14O6 | ESI- | 9.675 | 289.0721 | 290.0794 | -1.18 | 93.32 | 97.17 | 89.48 | 6179242 | 5.5894 | DBSearch-LibSearch |
| 126 | Loquatoside | C20H22O11 | ESI- | 9.75 | 437.1085 | 438.1157 | 1.14 | 49.02 | 98.05 | 0 | 125676 | 0.1137 | DBSearch |
| 127 | (+)-Gallocatechin (EGC) | C15H14O7 | ESI- | 9.775 | 305.0663 | 306.0737 | 0.83 | 49.38 | 98.76 | 0 | 76723 | 0.0694 | DBSearch |
| 128 | Procyanidin B2 | C30H26O12 | ESI- | 9.926 | 577.1351 | 578.1422 | 0.31 | 90.09 | 99.45 | 80.72 | 1842543 | 1.6666 | DBSearch-LibSearch |
| 129 | beta-D-Glucopyranosyl-11-hydroxyjasmonic acid | C18H28O9 | ESI- | 10.152 | 387.1658 | 388.173 | 0.87 | 47.16 | 94.32 | 0 | 62504 | 0.0565 | DBSearch |
| 130 | Epicatechin-(4beta->8)-epigallocatechin 3-O-gallate | C37H30O17 | ESI- | 10.29 | 745.1399 | 746.147 | 1.77 | 48.48 | 96.96 | 0 | 229957 | 0.208 | DBSearch |
| 131 | Marmesin | C14H14O4 | ESI- | 10.654 | 245.0821 | 246.0893 | -0.4 | 89.24 | 86.48 | 92 | 848677 | 0.7677 | DBSearch-LibSearch |
| 132 | Epicatechin | C15H14O6 | ESI- | 10.654 | 289.0731 | 290.0801 | -3.69 | 92.09 | 93.33 | 90.84 | 15640127 | 14.1471 | DBSearch-LibSearch |
| 133 | 6''-p-Coumaroylprunin | C30H28O12 | ESI- | 10.679 | 579.1508 | 580.1578 | 0.43 | 49.39 | 98.78 | 0 | 296936 | 0.2686 | DBSearch |
| 134 | 3-O-p-Coumaroylquinic acid | C16H18O8 | ESI- | 10.729 | 337.0928 | 338.1 | 0.4 | 49.69 | 99.38 | 0 | 1762519 | 1.5943 | DBSearch |
| 135 | Assamicain C | C44H36O22 | ESI- | 10.855 | 915.1621 | 916.1693 | 0.55 | 49.56 | 99.12 | 0 | 5929397 | 5.3634 | DBSearch |
| 136 | Epigallocatechin Gallate (EGCG) | C22H18O11 | ESI- | 10.93 | 457.0773 | 458.0846 | 0.75 | 98.27 | 99.06 | 97.47 | 4336442 | 3.9225 | DBSearch-LibSearch |
| 137 | 3-O-Feruloylquinic acid | C17H20O9 | ESI- | 11.131 | 367.103 | 368.1103 | 1.27 | 49.56 | 99.12 | 0 | 228227 | 0.2064 | DBSearch |
| 138 | resorcinol | C6H6O2 | ESI- | 11.156 | 109.0293 | 110.0368 | -0.35 | 87.88 | 93.5 | 82.26 | 47245 | 0.0427 | DBSearch-LibSearch |
| 139 | ent-Epicatechin-(4alpha->8)-ent-epicatechin 3-gallate | C37H30O16 | ESI- | 11.369 | 729.1458 | 730.1528 | 0.76 | 49.2 | 98.4 | 0 | 701579 | 0.6346 | DBSearch |

| | | | | | | | | | | | | | |
|---|---|---|---|---|---|---|---|---|---|---|---|---|---|
| 140 | Quercetin-3'-glucuronide | C21H20O13 | ESI- | 11.495 | 479.0828 | 480.09 | 0.9 | 49.49 | 98.99 | 0 | 877655 | 0.7939 | DBSearch |
| 141 | Xanthotoxol arabinoside | C16H14O8 | ESI- | 11.533 | 333.0612 | 334.0687 | 0.62 | 48.85 | 97.7 | 0 | 211526 | 0.1913 | DBSearch |
| 142 | Quercetin 3-O-(6''-acetyl-glucoside) | C23H22O13 | ESI- | 11.582 | 505.098 | 506.1053 | 1.48 | 48.77 | 97.54 | 0 | 62379 | 0.0564 | DBSearch |
| 143 | Kaempferol 3-gentiobioside 7-rhamnoside | C33H40O20 | ESI- | 11.633 | 755.2031 | 756.2101 | 1.55 | 48.31 | 96.61 | 0 | 121324 | 0.1097 | DBSearch |
| 144 | Margrapine A | C21H23NO7 | ESI- | 11.683 | 400.1395 | 401.1468 | 1.52 | 48.75 | 97.5 | 0 | 39847 | 0.036 | DBSearch |
| 145 | 3-Caffeoyl-1,5-quinolactone | C16H16O8 | ESI- | 11.959 | 335.0767 | 336.0839 | 1.77 | 48.16 | 96.32 | 0 | 146767 | 0.1328 | DBSearch |
| 146 | Rutin | C27H30O16 | ESI- | 11.984 | 609.1457 | 610.1529 | 0.8 | 98.61 | 98.58 | 98.64 | 5126937 | 4.6375 | DBSearch-LibSearch |
| 147 | 6''-O-Galloylquercimeritrin | C28H24O16 | ESI- | 12.059 | 615.0983 | 616.1055 | 1.53 | 49.16 | 98.33 | 0 | 101131 | 0.0915 | DBSearch |
| 148 | alpha-Rhamnorobin | C21H20O10 | ESI- | 12.135 | 431.0974 | 432.1047 | 2.16 | 39.99 | 79.97 | 0 | 171531 | 0.1552 | DBSearch |
| 149 | Geniposide pentaacetate | C27H34O15 | ESI- | 12.235 | 597.1817 | 598.1888 | 1.58 | 48.3 | 96.6 | 0 | 94276 | 0.0853 | DBSearch |
| 150 | Quercetin 3-galactoside | C21H20O12 | ESI- | 12.537 | 463.088 | 464.0952 | 0.62 | 96.16 | 98.16 | 94.17 | 4251674 | 3.8458 | DBSearch-LibSearch |
| 151 | SB 206553 | C17H16N4O | ESI- | 12.687 | 291.1265 | 292.1336 | -3.94 | 38.57 | 77.13 | 0 | 56486 | 0.0511 | DBSearch |
| 152 | 7-Galloylcatechin (ECG) | C22H18O10 | ESI- | 12.838 | 441.0825 | 442.0898 | 0.36 | 49.19 | 98.38 | 0 | 6574501 | 5.9469 | DBSearch |
| 153 | Isovitexin 2''-O-glucoside | C27H30O15 | ESI- | 12.887 | 593.1511 | 594.1582 | 0.42 | 49.64 | 99.28 | 0 | 204596 | 0.1851 | DBSearch |
| 154 | Gallic acid | C7H6O5 | ESI- | 12.887 | 169.0142 | 170.0215 | 0.24 | 93.92 | 99.59 | 88.24 | 503621 | 0.4555 | DBSearch-LibSearch |
| 155 | Naringenin-4'-O-glucuronide | C21H20O11 | ESI- | 13.515 | 447.093 | 448.1002 | 0.75 | 49.62 | 99.23 | 0 | 1824382 | 1.6502 | DBSearch |
| 156 | Isovitexin 2''-O-[4-hydroxy-(E)-cinnamoyl-(->6)-beta-D-glucopyranosyl] 4'-O-beta-D-glucopyranoside | C42H46O22 | ESI- | 14.645 | 901.2393 | 902.2464 | 1.81 | 48.51 | 97.02 | 0 | 280118 | 0.2534 | DBSearch |
| 157 | Hieracin | C15H10O7 | ESI- | 15.096 | 301.0347 | 302.0419 | 2.46 | 89.59 | 97.41 | 81.76 | 55201 | 0.0499 | DBSearch-LibSearch |
| 158 | Ustiloxin D | C23H34N4O8 | ESI- | 15.849 | 493.2287 | 494.236 | 3.45 | 44.83 | 89.66 | 0 | 508951 | 0.4604 | DBSearch |
| 159 | Acuminoside | C21H36O10 | ESI- | 15.874 | 447.2229 | 448.2302 | 1.44 | 49.31 | 98.63 | 0 | 109297 | 0.0989 | DBSearch |

# Table S3.

## Predicted Pharmacokinetic and Toxicological Properties of *Camellia sinensis* Bioactive Compounds

| | Compound | Chem CID | QED | PAINS alert | F30% | Bioavailability Score | HIA | PPB | Fu | hERG | DILI | Ames | NR-ER | NR-Aromatase |
|---|---|---|---|---|---|---|---|---|---|---|---|---|---|---|
| 1 | (-)-Epigallocatechin gallate (EGCG) | 65064 | 0.21 | 1 alert: catechol_A | 0.999778 | 0.17 | 0.00312 | 87.25 | 13.71 | 0.05 | 0.94 | 0.85 | 0.0094 | 0.0283374 |
| 2 | Epicatechin | 72276 | 0.51 | 1 | 0.98782 | 0.55 | 0.01601 | 92.65 | 14.88 | 0.11 | 0.37 | 0.60 | 0.2397 | 0.2905551 |
| 3 | (±)-Catechin | 9064 | 0.51 | 1 | 0.994156 | 0.55 | 0.0161 | 89.28 | 17.11 | 0.13 | 0.22 | 0.46 | 0.2003 | 0.2532855 |
| 4 | Rutin | 5280805 | 0.14 | 1 | 0.999751 | 0.17 | 0.63973 | 85.01 | 14.66 | 0.01 | 0.94 | 0.76 | 0.7747 | 0.1548417 |
| 5 | Quercetin 3-galactoside | 5281643 | 0.23 | 1 | 0.999758 | 0.17 | 0.72302 | 84.93 | 13.50 | 0.01 | 0.91 | 0.94 | 0.762 | 0.4483061 |
| 6 | Procyanidin B2 | 122738 | 0.16 | 1 | 0.999981 | 0.17 | 0.00037 | 90.34 | 17.46 | 0.05 | 0.74 | 0.71 | 0.0725 | 0.4499031 |
| 7 | (−)-Naringenin | 932 | 0.74 | 0 | 0.849586 | 0.55 | 0.00083 | 94.96 | 7.37 | 0.15 | 0.33 | 0.68 | 0.9867 | 0.2774405 |
| 8 | Kaempferol | 5280863 | 0.55 | 0 | 0.715565 | 0.55 | 0.01514 | 97.88 | 1.36 | 0.07 | 0.70 | 0.55 | 0.9982 | 0.9647095 |
| 9 | Chlorogenic acid | 1794427 | 0.23 | 1 | 0.995865 | 0.11 | 0.10626 | 64.83 | 31.62 | 0.03 | 0.29 | 0.39 | 0.5636 | 0.001294 |
| 10 | Gallic acid | 370 | 0.46 | 1 | 0.355294 | 0.56 | 0.01709 | 37.57 | 55.13 | 0.03 | 0.70 | 0.48 | 0.0108 | 0.0027893 |
| 11 | Caffeic acid | 689043 | 0.47 | 1 | 0.995638 | 0.56 | 0.30119 | 64.67 | 32.16 | 0.04 | 0.69 | 0.27 | 0.2801 | 0.0336224 |
| 12 | Herbacetin | 5280544 | 0.34 | 1 | 0.929294 | 0.55 | 0.04932 | 98.45 | 1.27 | 0.04 | 0.80 | 0.59 | 0.9935 | 0.9303793 |
| 13 | Caffeine | 2519 | 0.54 | 0 | 0.00011 | 0.55 | 0.00017 | 32.52 | 62.79 | 0.07 | 0.82 | 0.59 | 5E-07 | 4.38E-12 |
| 14 | Theanine | 439378 | 0.51 | 0 | 0.005599 | 0.55 | 0.03069 | 11.94 | 92.92 | 0.05 | 0.07 | 0.60 | 0.0535 | 0.0010653 |
| 15 | Theophylline | 2153 | 0.56 | 0 | 0.000324 | 0.55 | 0.00075 | 26.35 | 68.49 | 0.06 | 0.90 | 0.53 | 3E-06 | 9.29E-12 |
| 16 | Marmesin | 334704 | 0.78 | 0 | 0.600122 | 0.55 | 0.00145 | 72.36 | 23.84 | 0.15 | 0.77 | 0.70 | 0.2462 | 0.0567432 |
| 17 | 4-Hydroxycoumarin | 54682930 | 0.60 | 0 | 0.225649 | 0.55 | 0.01807 | 88.60 | 10.69 | 0.07 | 0.59 | 0.59 | 0.2075 | 0.0099813 |
| 18 | Aesculin | 5281417 | 0.43 | 0 | 0.936478 | 0.55 | 0.02654 | 65.34 | 36.13 | 0.02 | 0.93 | 0.86 | 0.6683 | 0.1558173 |
| 19 | Epicatechin Gallate (ECG) | 107905 | 0.24 | 1 | 0.999384 | 0.55 | 0.00144 | 90.56 | 10.97 | 0.06 | 0.85 | 0.81 | 0.0385 | 0.0718726 |

| | | | | | | | | | | | | | | |
|---|---|---|---|---|---|---|---|---|---|---|---|---|---|---|
| 20 | Epigallocatechin (EGC) | 72277 | 0.44 | 1 | 0.997253 | 0.55 | 0.02536 | 89.37 | 19.44 | 0.07 | 0.69 | 0.70 | 0.0528 | 0.1258281 |

# Table S5.

### Per-compound contribution to the 292 shared targets

| Compound | Predicted targets (n) | Targets in the 292 (n) | % of 292 |
|---|---|---|---|
| (-)-Epigallocatechin gallate (EGCG) | 146 | 84 | 28.8 |
| (−)-Naringenin | 103 | 65 | 22.3 |
| Caffeic acid | 101 | 62 | 21.2 |
| Herbacetin | 101 | 61 | 20.9 |
| Epigallocatechin (EGC) | 104 | 59 | 20.2 |
| (±)-Catechin | 104 | 59 | 20.2 |
| Epicatechin | 104 | 59 | 20.2 |
| Quercetin 3-galactoside | 99 | 59 | 20.2 |
| Epicatechin Gallate (ECG) | 104 | 58 | 19.9 |
| Kaempferol | 105 | 58 | 19.9 |
| Chlorogenic acid | 100 | 56 | 19.2 |
| Rutin | 98 | 55 | 18.8 |
| 4-Hydroxycoumarin | 103 | 55 | 18.8 |
| Procyanidin B2 | 95 | 54 | 18.5 |
| Aesculin | 151 | 54 | 18.5 |
| Marmesin | 98 | 53 | 18.2 |
| Theanine | 108 | 51 | 17.5 |
| Gallic acid | 100 | 50 | 17.1 |
| Theophylline | 103 | 48 | 16.4 |
| Caffeine | 101 | 47 | 16.1 |

# Table S6.

## 292 putative therapeutic target genes of CSE against Endometriosis

| | Genes | Abbreviation |
|---|---|---|
| 1 | ADAMTS5 | A disintegrin and metalloproteinase with thrombospondin motifs 5 |
| 2 | MAPK14 | Mitogen-activated protein kinase 14 |
| 3 | BACE1 | Beta-secretase 1 |
| 4 | MMP14 | Matrix metalloproteinase-14 |
| 5 | GSK3B | Glycogen synthase kinase-3 beta |
| 6 | CSNK1E | Casein kinase I isoform epsilon |
| 7 | MTOR | Serine/threonine-protein kinase mTOR |
| 8 | PIK3CA | Phosphatidylinositol 4,5-bisphosphate 3-kinase catalytic subunit alpha isoform |
| 9 | STAT1 | Signal transducer and activator of transcription 1-alpha/beta |
| 10 | DNMT1 | DNA (cytosine-5)-methyltransferase 1 |
| 11 | HSPD1 | 60 kDa heat shock protein, mitochondrial |
| 12 | BCL2 | Apoptosis regulator Bcl-2 |
| 13 | RPSA | 40S ribosomal protein SA |
| 14 | MET | Hepatocyte growth factor receptor |
| 15 | MMP2 | 72 kDa type IV collagenase |
| 16 | ABCB1 | ATP-dependent translocase ABCB1 |
| 17 | MMP1 | 22 kDa interstitial collagenase |
| 18 | TERT | Telomerase reverse transcriptase |
| 19 | PTGES | Prostaglandin E synthase |
| 20 | KCNH2 | Potassium voltage-gated channel subfamily H member 2 |
| 21 | PKM | Pyruvate kinase PKM |
| 22 | SYNJ2 | Synaptojanin-2 |
| 23 | CA2 | Carbonic anhydrase 2 |
| 24 | CA1 | Carbonic anhydrase 1 |
| 25 | GABRA1 | Gamma-aminobutyric acid receptor subunit alpha-1 |
| 26 | PTGS1 | Prostaglandin G/H synthase 1 |
| 27 | MMP13 | Collagenase 3 |
| 28 | MMP9 | 67 kDa matrix metalloproteinase-9 |
| 29 | STING1 | Stimulator of interferon genes protein |
| 30 | ABCG2 | Broad substrate specificity ATP-binding cassette transporter ABCG2 |
| 31 | ABCC1 | Multidrug resistance-associated protein 1 |
| 32 | DPP4 | Dipeptidyl peptidase 4 membrane form |
| 33 | CYP1B1 | Cytochrome P450 1B1 |
| 34 | PLK1 | Serine/threonine-protein kinase PLK1 |
| 35 | CYP19A1 | Aromatase |
| 36 | HDAC6 | Histone deacetylase 6 |
| 37 | HIF1A | Hypoxia-inducible factor 1-alpha |
| 38 | SERPINE1 | Plasminogen activator inhibitor 1 |

| | | |
|---|---|---|
| 39 | KDR | Vascular endothelial growth factor receptor 2 |
| 40 | SRC | Proto-oncogene tyrosine-protein kinase Src |
| 41 | AXL | Tyrosine-protein kinase receptor UFO |
| 42 | ESR2 | Estrogen receptor beta |
| 43 | KLK2 | Kallikrein-2 |
| 44 | KLK1 | Kallikrein-1 |
| 45 | PGF | Placenta growth factor |
| 46 | ESR1 | Estrogen receptor |
| 47 | MMP12 | Macrophage metalloelastase |
| 48 | CBR1 | Carbonyl reductase [NADPH] 1 |
| 49 | HSD17B1 | Estradiol 17-beta-dehydrogenase 1 |
| 50 | TYR | Tyrosinase |
| 51 | FGFR1 | Fibroblast growth factor receptor 1 |
| 52 | KIT | Mast/stem cell growth factor receptor Kit |
| 53 | KDM1A | Lysine-specific histone demethylase 1A |
| 54 | ALOX5 | Arachidonate 5-lipoxygenase |
| 55 | HSPE1 | 10 kDa heat shock protein, mitochondrial |
| 56 | GABRB2 | Gamma-aminobutyric acid receptor subunit beta-2 |
| 57 | GABRG2 | Gamma-aminobutyric acid receptor subunit gamma-2 |
| 58 | BAD | Bcl2-associated agonist of cell death |
| 59 | BCL2L1 | Bcl-2-like protein 1 |
| 60 | MCL1 | Induced myeloid leukemia cell differentiation protein Mcl-1 |
| 61 | ALOX12 | Arachidonate 12-lipoxygenase, 12S-type |
| 62 | MMP7 | Matrilysin |
| 63 | ELANE | Neutrophil elastase |
| 64 | HSD11B2 | Corticosteroid 11-beta-dehydrogenase isozyme 2 |
| 65 | ADAM17 | Disintegrin and metalloproteinase domain-containing protein 17 |
| 66 | TP53 | Cellular tumor antigen p53 |
| 67 | EP300 | Histone acetyltransferase p300 |
| 68 | CNR1 | Cannabinoid receptor 1 |
| 69 | CNR2 | Cannabinoid receptor 2 |
| 70 | CREBBP | CREB-binding protein |
| 71 | FASN | 3-hydroxyacyl-[acyl-carrier-protein] dehydratase |
| 72 | HSD11B1 | Corticosteroid 11-beta-dehydrogenase isozyme 1 |
| 73 | KAT2B | Histone acetyltransferase KAT2B |
| 74 | HSP90AB1 | Heat shock protein HSP 90-beta |
| 75 | ABCB11 | Bile salt export pump |
| 76 | COL1A1 | Collagen alpha-1(I) chain |
| 77 | PTGS2 | Prostaglandin G/H synthase 2 |
| 78 | MAPK11 | Mitogen-activated protein kinase 11 |
| 79 | HSPA5 | Endoplasmic reticulum chaperone BiP |
| 80 | PPARA | Peroxisome proliferator-activated receptor alpha |
| 81 | ACE | Angiotensin-converting enzyme, soluble form |

| | | |
|---|---|---|
| 82 | ICAM1 | Intercellular adhesion molecule 1 |
| 83 | ERBB2 | Receptor tyrosine-protein kinase erbB-2 |
| 84 | SLCO1B1 | Solute carrier organic anion transporter family member 1B1 |
| 85 | FLT1 | Vascular endothelial growth factor receptor 1 |
| 86 | EGFR | Epidermal growth factor receptor |
| 87 | MAOB | Amine oxidase [flavin-containing] B |
| 88 | AKT1 | RAC-alpha serine/threonine-protein kinase |
| 89 | PTPN1 | Tyrosine-protein phosphatase non-receptor type 1 |
| 90 | ALOX15 | Arachidonate 15-lipoxygenase |
| 91 | MAOA | Amine oxidase [flavin-containing] A |
| 92 | NOX4 | NADPH oxidase 4 |
| 93 | NAMPT | Nicotinamide phosphoribosyltransferase |
| 94 | AKR1B1 | Aldo-keto reductase family 1 member B1 |
| 95 | P4HB | Protein disulfide-isomerase |
| 96 | PDE5A | cGMP-specific 3',5'-cyclic phosphodiesterase |
| 97 | IKBKB | Inhibitor of nuclear factor kappa-B kinase subunit beta |
| 98 | IL2 | Interleukin-2 |
| 99 | TNF | Tumor necrosis factor, membrane form |
| 100 | PLG | Plasmin heavy chain A, short form |
| 101 | PPARG | Peroxisome proliferator-activated receptor gamma |
| 102 | ALDH1B1 | Aldehyde dehydrogenase X, mitochondrial |
| 103 | ALDH2 | Aldehyde dehydrogenase, mitochondrial |
| 104 | ALDH1A2 | Retinal dehydrogenase 2 |
| 105 | IP6K2 | Inositol hexakisphosphate kinase 2 |
| 106 | CYP1A1 | Cytochrome P450 1A1 |
| 107 | CTNNB1 | Catenin beta-1 |
| 108 | ALK | ALK tyrosine kinase receptor |
| 109 | AURKB | Aurora kinase B |
| 110 | MYLK | Myosin light chain kinase, smooth muscle, deglutamylated form |
| 111 | ELAVL1 | ELAV-like protein 1 |
| 112 | PTK2 | Focal adhesion kinase 1 |
| 113 | CSNK2A1 | Casein kinase II subunit alpha |
| 114 | DAPK1 | Death-associated protein kinase 1 |
| 115 | NEK2 | Serine/threonine-protein kinase Nek2 |
| 116 | PIK3CG | Phosphatidylinositol 4,5-bisphosphate 3-kinase catalytic subunit gamma isoform |
| 117 | HSD17B2 | Estradiol 17-beta-dehydrogenase 2 |
| 118 | PIK3R1 | Phosphatidylinositol 3-kinase regulatory subunit alpha |
| 119 | MMP3 | Stromelysin-1 |
| 120 | IGF1R | Insulin-like growth factor 1 receptor alpha chain |
| 121 | INSR | Insulin receptor subunit alpha |
| 122 | CDK1 | Cyclin-dependent kinase 1 |
| 123 | CXCR1 | C-X-C chemokine receptor type 1 |

| | | |
|---|---|---|
| 124 | TOP2A | DNA topoisomerase 2-alpha |
| 125 | MPO | Myeloperoxidase heavy chain |
| 126 | F2 | Activation peptide fragment 1 |
| 127 | APEX1 | DNA-(apurinic or apyrimidinic site) lyase, mitochondrial |
| 128 | PNLIP | Pancreatic lipase |
| 129 | PLA2G2A | Phospholipase A2, membrane associated |
| 130 | SHBG | Sex hormone-binding globulin |
| 131 | AR | Androgen receptor |
| 132 | IDO1 | Indoleamine 2,3-dioxygenase 1 |
| 133 | AKR1C3 | Aldo-keto reductase family 1 member C3 |
| 134 | ROR1 | Inactive tyrosine-protein kinase transmembrane receptor ROR1 |
| 135 | CTSB | Cathepsin B heavy chain |
| 136 | CDK4 | Cyclin-dependent kinase 4 |
| 137 | RXRA | Retinoic acid receptor RXR-alpha |
| 138 | DRD2 | D(2) dopamine receptor |
| 139 | TRPV1 | Transient receptor potential cation channel subfamily V member 1 |
| 140 | CCND1 | G1/S-specific cyclin-D1 |
| 141 | AHR | Aryl hydrocarbon receptor |
| 142 | ESRRA | Steroid hormone receptor ERR1 |
| 143 | SLC2A1 | Solute carrier family 2, facilitated glucose transporter member 1 |
| 144 | YES1 | Tyrosine-protein kinase Yes |
| 145 | METTL3 | N6-adenosine-methyltransferase catalytic subunit |
| 146 | CDK6 | Cyclin-dependent kinase 6 |
| 147 | TNKS | Poly [ADP-ribose] polymerase tankyrase-1 |
| 148 | FTO | Alpha-ketoglutarate-dependent dioxygenase FTO |
| 149 | ALKBH5 | RNA demethylase ALKBH5 |
| 150 | CCNB1 | G2/mitotic-specific cyclin-B1 |
| 151 | METTL14 | N6-adenosine-methyltransferase non-catalytic subunit |
| 152 | RELA | Transcription factor p65 |
| 153 | PRKCD | Protein kinase C delta type regulatory subunit |
| 154 | PRKCE | Protein kinase C epsilon type |
| 155 | TLR4 | Toll-like receptor 4 |
| 156 | ENPP1 | Ectonucleotide pyrophosphatase/phosphodiesterase family member 1, secreted form |
| 157 | METAP2 | Methionine aminopeptidase 2 |
| 158 | AKR1C2 | Aldo-keto reductase family 1 member C2 |
| 159 | MAPK1 | Mitogen-activated protein kinase 1 |
| 160 | AKR1C4 | Aldo-keto reductase family 1 member C4 |
| 161 | HSP90AA1 | Heat shock protein HSP 90-alpha |
| 162 | NLRP3 | NACHT, LRR and PYD domains-containing protein 3 |
| 163 | PRKCB | Protein kinase C beta type |
| 164 | PRKCG | Protein kinase C gamma type |
| 165 | NFE2L2 | Nuclear factor erythroid 2-related factor 2 |

| | | |
|---|---|---|
| 166 | PARP1 | Poly [ADP-ribose] polymerase 1 |
| 167 | FDPS | Farnesyl pyrophosphate synthase |
| 168 | FDFT1 | Squalene synthase |
| 169 | STAT3 | Signal transducer and activator of transcription 3 |
| 170 | HTR1A | 5-hydroxytryptamine receptor 1A |
| 171 | TRPC1 | Short transient receptor potential channel 1 |
| 172 | LDHA | Lactate dehydrogenase A |
| 173 | PTPN11 | Tyrosine-protein phosphatase non-receptor type 11 |
| 174 | HMGB1 | High mobility group protein B1 |
| 175 | TYMS | Thymidylate synthase |
| 176 | ALB | Serum albumin |
| 177 | HDAC1 | Histone deacetylase 1 |
| 178 | TRIM24 | Transcription intermediary factor 1-alpha |
| 179 | NR4A2 | Nuclear receptor subfamily 4 group A member 2 |
| 180 | NR4A1 | Nuclear receptor subfamily 4 group A member 1 |
| 181 | SRD5A2 | 3-oxo-5-alpha-steroid 4-dehydrogenase 2 |
| 182 | AKR1C1 | Aldo-keto reductase family 1 member C1 |
| 183 | ERN1 | Serine/threonine-protein kinase/endoribonuclease IRE1 |
| 184 | MMP8 | Neutrophil collagenase |
| 185 | ALDH1A3 | Aldehyde dehydrogenase family 1 member A3 |
| 186 | NR4A3 | Nuclear receptor subfamily 4 group A member 3 |
| 187 | AGTR1 | Type-1 angiotensin II receptor |
| 188 | MIF | Macrophage migration inhibitory factor |
| 189 | XIAP | E3 ubiquitin-protein ligase XIAP |
| 190 | TRPA1 | Transient receptor potential cation channel subfamily A member 1 |
| 191 | CYP2C19 | Cytochrome P450 2C19 |
| 192 | CYP2C9 | Cytochrome P450 2C9 |
| 193 | CYP3A4 | Cytochrome P450 3A4 |
| 194 | CYP1A2 | Cytochrome P450 1A2 |
| 195 | PTK2B | Protein-tyrosine kinase 2-beta |
| 196 | CCNE1 | G1/S-specific cyclin-E1 |
| 197 | PARK7 | Protein/nucleic acid deglycase DJ-1 |
| 198 | COPS5 | COP9 signalosome complex subunit 5 |
| 199 | F3 | Tissue factor |
| 200 | PFKFB3 | 6-phosphofructo-2-kinase/fructose-2,6-bisphosphatase 3 |
| 201 | BRAF | Serine/threonine-protein kinase B-raf |
| 202 | CYP2C8 | Cytochrome P450 2C8 |
| 203 | SIRT3 | NAD-dependent protein deacetylase sirtuin-3, mitochondrial |
| 204 | LGMN | Legumain |
| 205 | BRDT | Bromodomain testis-specific protein |
| 206 | CHIT1 | Chitotriosidase-1 |
| 207 | SIRT1 | NAD-dependent protein deacetylase sirtuin-1 |
| 208 | OPRM1 | Mu-type opioid receptor |

| | | |
|---|---|---|
| 209 | TK1 | Thymidine kinase, cytosolic |
| 210 | TYMP | Thymidine phosphorylase |
| 211 | NOD1 | Nucleotide-binding oligomerization domain-containing protein 1 |
| 212 | ALDH1A1 | Retinal dehydrogenase 1 |
| 213 | PCSK9 | Proprotein convertase subtilisin/kexin type 9 |
| 214 | NOD2 | Nucleotide-binding oligomerization domain-containing protein 2 |
| 215 | EPHB4 | Ephrin type-B receptor 4 |
| 216 | JAK3 | Tyrosine-protein kinase JAK3 |
| 217 | ABL1 | Tyrosine-protein kinase ABL1 |
| 218 | GABRA5 | Gamma-aminobutyric acid receptor subunit alpha-5 |
| 219 | JAK2 | Tyrosine-protein kinase JAK2 |
| 220 | PRKDC | DNA-dependent protein kinase catalytic subunit |
| 221 | VCP | Transitional endoplasmic reticulum ATPase |
| 222 | FEN1 | Flap endonuclease 1 |
| 223 | MAPK9 | Mitogen-activated protein kinase 9 |
| 224 | MAPK8 | Mitogen-activated protein kinase 8 |
| 225 | GPR18 | N-arachidonyl glycine receptor |
| 226 | NOS2 | Nitric oxide synthase, inducible |
| 227 | NOS1 | Nitric oxide synthase, brain |
| 228 | NOS3 | Nitric oxide synthase, endothelial |
| 229 | MME | Neprilysin |
| 230 | SLC6A11 | Sodium- and chloride-dependent GABA transporter 3 |
| 231 | CPB2 | Carboxypeptidase B2 |
| 232 | ARG1 | Arginase-1 |
| 233 | ITGB3 | Integrin beta-3 |
| 234 | SLC1A2 | Excitatory amino acid transporter 2 |
| 235 | ITGB1 | Integrin beta-1 |
| 236 | SLC1A1 | Excitatory amino acid transporter 3 |
| 237 | GRIK2 | Glutamate receptor ionotropic, kainate 2 |
| 238 | GRIK3 | Glutamate receptor ionotropic, kainate 3 |
| 239 | NR3C1 | Glucocorticoid receptor |
| 240 | ITGAV | Integrin alpha-V heavy chain |
| 241 | PEPD | Xaa-Pro dipeptidase |
| 242 | TGFBR1 | TGF-beta receptor type-1 |
| 243 | LAP3 | Cytosol aminopeptidase |
| 244 | PTGER2 | Prostaglandin E2 receptor EP2 subtype |
| 245 | LTA4H | Leukotriene A-4 hydrolase |
| 246 | PTGER3 | Prostaglandin E2 receptor EP3 subtype |
| 247 | ENPEP | Glutamyl aminopeptidase |
| 248 | PTGER4 | Prostaglandin E2 receptor EP4 subtype |
| 249 | GRM5 | Metabotropic glutamate receptor 5 |
| 250 | CHRNA7 | Neuronal acetylcholine receptor subunit alpha-7 |
| 251 | ARG2 | Arginase-2, mitochondrial |

| | | |
|---|---|---|
| 252 | EIF4A1 | Eukaryotic initiation factor 4A-I |
| 253 | NR1H4 | Bile acid receptor |
| 254 | CASP3 | Caspase-3 subunit p12 |
| 255 | EPHX1 | Epoxide hydrolase 1 |
| 256 | GABRA4 | Gamma-aminobutyric acid receptor subunit alpha-4 |
| 257 | ITGA5 | Integrin alpha-5 heavy chain |
| 258 | ITGB2 | Integrin beta-2 |
| 259 | TYK2 | Non-receptor tyrosine-protein kinase TYK2 |
| 260 | PCK1 | Phosphoenolpyruvate carboxykinase, cytosolic [GTP] |
| 261 | GABRB3 | Gamma-aminobutyric acid receptor subunit beta-3 |
| 262 | FHIT | Bis(5'-adenosyl)-triphosphatase |
| 263 | PPARD | Peroxisome proliferator-activated receptor delta |
| 264 | SRD5A1 | 3-oxo-5-alpha-steroid 4-dehydrogenase 1 |
| 265 | CYP11B1 | Cytochrome P450 11B1, mitochondrial |
| 266 | XPO1 | Exportin-1 |
| 267 | CCR5 | C-C chemokine receptor type 5 |
| 268 | CCR1 | C-C chemokine receptor type 1 |
| 269 | ACKR3 | Atypical chemokine receptor 3 |
| 270 | NQO1 | NAD(P)H dehydrogenase [quinone] 1 |
| 271 | PGR | Progesterone receptor |
| 272 | NFKB1 | Nuclear factor NF-kappa-B p105 subunit |
| 273 | FAAH | Fatty-acid amide hydrolase 1 |
| 274 | HSD17B3 | Testosterone 17-beta-dehydrogenase 3 |
| 275 | MAP1LC3B | Microtubule-associated proteins 1A/1B light chain 3B |
| 276 | AURKA | Aurora kinase A |
| 277 | ANTXR2 | Anthrax toxin receptor 2 |
| 278 | PDGFRB | Platelet-derived growth factor receptor beta |
| 279 | HSPA1B | Heat shock 70 kDa protein 1A |
| 280 | FLT4 | Vascular endothelial growth factor receptor 3 |
| 281 | TEK | Angiopoietin-1 receptor |
| 282 | MAP3K8 | Mitogen-activated protein kinase kinase kinase 8 |
| 283 | TOP1 | DNA topoisomerase 1 |
| 284 | NT5E | 5'-nucleotidase |
| 285 | TNNI3 | Troponin I, cardiac muscle |
| 286 | SLC6A4 | Sodium-dependent serotonin transporter |
| 287 | NDUFS2 | NADH dehydrogenase [ubiquinone] iron-sulfur protein 2, mitochondrial |
| 288 | MT-ND5 | NADH-ubiquinone oxidoreductase chain 5 |
| 289 | NDUFS1 | NADH-ubiquinone oxidoreductase 75 kDa subunit, mitochondrial |
| 290 | NDUFV1 | NADH dehydrogenase [ubiquinone] flavoprotein 1, mitochondrial |
| 291 | NDUFA5 | NADH dehydrogenase [ubiquinone] 1 alpha subcomplex subunit 5 |
| 292 | NDUFA13 | NADH dehydrogenase [ubiquinone] 1 alpha subcomplex subunit 13 |

# Table S7.

## 20 hub genes according to degree and betweenness

| Gene | degree | betweenness |
|---|---|---|
| TP53 | 56 | 7506.7 |
| SRC | 42 | 3825.3 |
| STAT3 | 37 | 2664 |
| HSP90AA1 | 36 | 3560.2 |
| AKT1 | 34 | 1398 |
| ESR1 | 32 | 2876 |
| CTNNB1 | 29 | 1084.2 |
| HSP90AB1 | 28 | 1214.8 |
| PIK3CA | 28 | 298 |
| PIK3R1 | 26 | 999 |
| EGFR | 25 | 438.2 |
| PTK2 | 25 | 463 |
| EP300 | 23 | 507.3 |
| MAPK1 | 22 | 612 |
| CYP3A4 | 20 | 567 |
| NFKB1 | 20 | 430 |
| PTPN11 | 20 | 57 |
| CREBBP | 19 | 968 |
| HIF1A | 19 | 351.3 |
| CYP1A1 | 18 | 1535 |

# Table S8.

## Top 10 hub genes of the 292-target PPI network ranked independently by three cytoHubba centrality algorithms.

| Rank | Degree | MCC | MNC |
|---|---|---|---|
| 1 | **TP53** | **SRC *** | **TP53** |
| 2 | **SRC *** | PTPN11 | **SRC *** |
| 3 | **STAT3 *** | JAK2 | **STAT3 *** |
| 4 | **HSP90AA1** | **PIK3R1 *** | **AKT1** |
| 5 | **AKT1** | EGFR | **HSP90AA1** |
| 6 | **ESR1** | PTK2 | **ESR1** |
| 7 | **CTNNB1** | ERBB2 | **CTNNB1** |
| 8 | **HSP90AB1** | IGF1R | **HSP90AB1** |
| 9 | **PIK3CA *** | **STAT3 *** | **PIK3CA *** |
| 10 | **PIK3R1 *** | **PIK3CA *** | **PIK3R1 *** |

Rankings were derived using Degree (global connectivity), MCC (Maximal Clique Centrality; dense-clique membership) and MNC (Maximum Neighbourhood Component; neighbourhood size). Genes ranked in the top 10 by ≥ 2 of the three methods (bold) were retained as consensus hubs; * denotes genes shared by all three methods (SRC, STAT3, PIK3CA and PIK3R1).

# Figure S1.

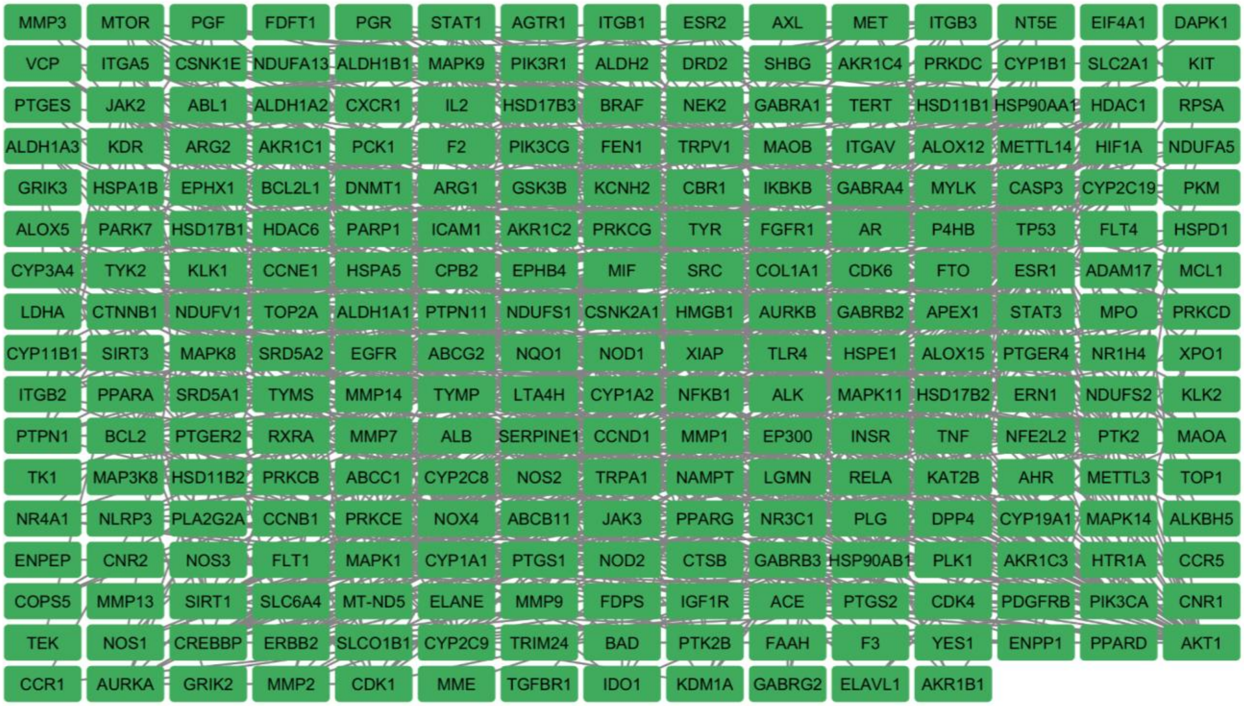


**Figure S1: A protein interaction network diagram of active components and disease targets (292) of CSE was constructed**

# Figure S2.

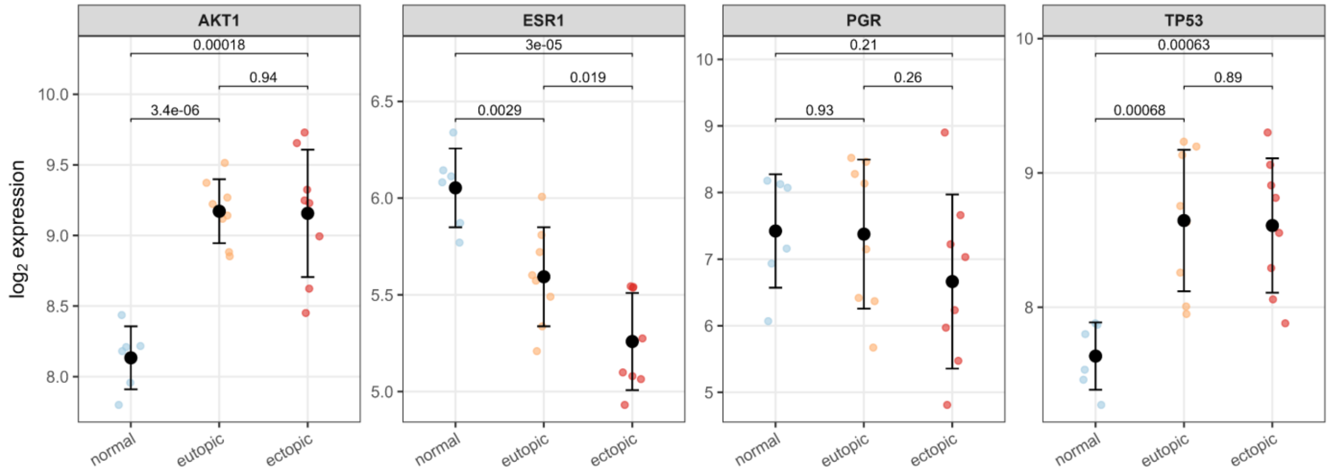


**Figure S2: Expression of key hub genes across the three GSE25628 groups (normal, n = 6; eutopic, n = 8; ectopic, n = 8). Black points with error bars show mean ± SD; coloured points are individual samples; pairwise p-values are from two-sample t-tests. AKT1 and TP53 are up-regulated already in eutopic endometrium with little further change in ectopic tissue (a systemic field effect), whereas ESR1 declines progressively from normal to eutopic to ectopic tissue; PGR shows a non-significant downward trend.**
